# Supplementary figures and images for: simona: a comprehensive R package for semantic similarity analysis on bio-ontologies (part 1 of 3)
Source: BMC Genomics. 2024 Sep 16;25:869. doi: 10.1186/s12864-024-10759-4 (PMC11406866; doi:10.1186/s12864-024-10759-4)

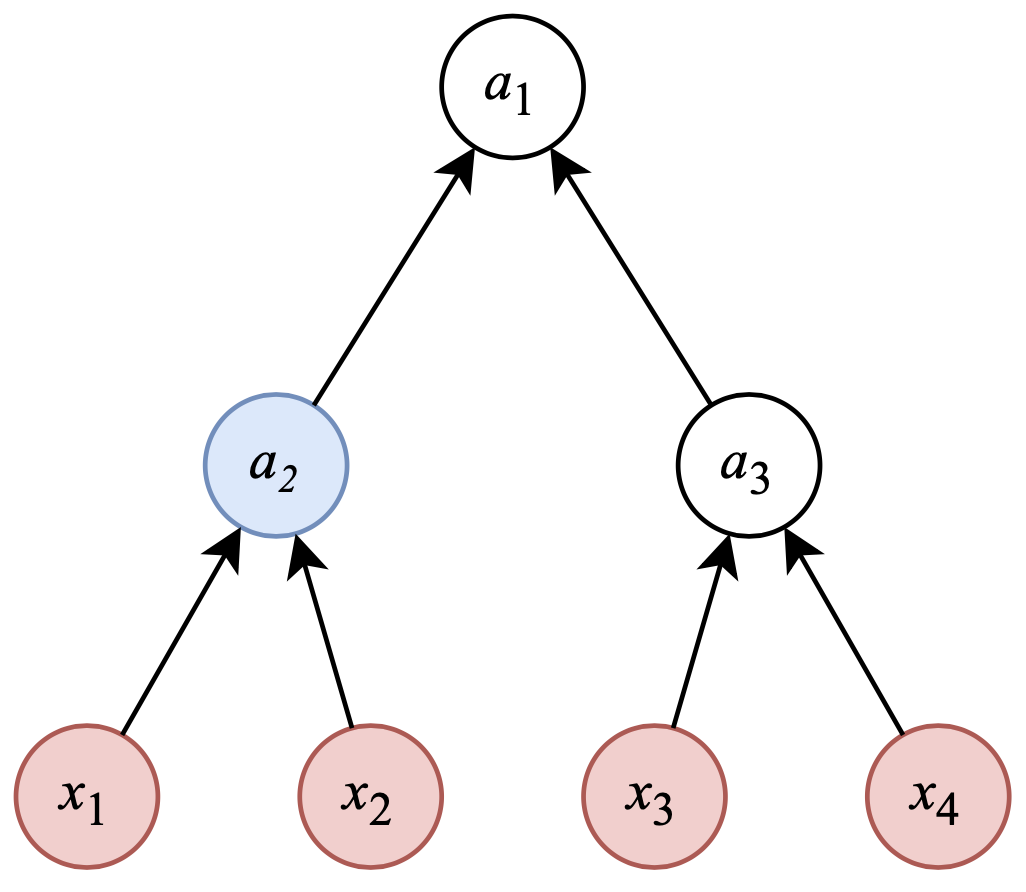

Supplement: Supplementary file 2 — Supplementary Material 2. The algorithm [file 12864_2024_10759_MOESM2_ESM.zip › suppl2_algorithm/f1.png]

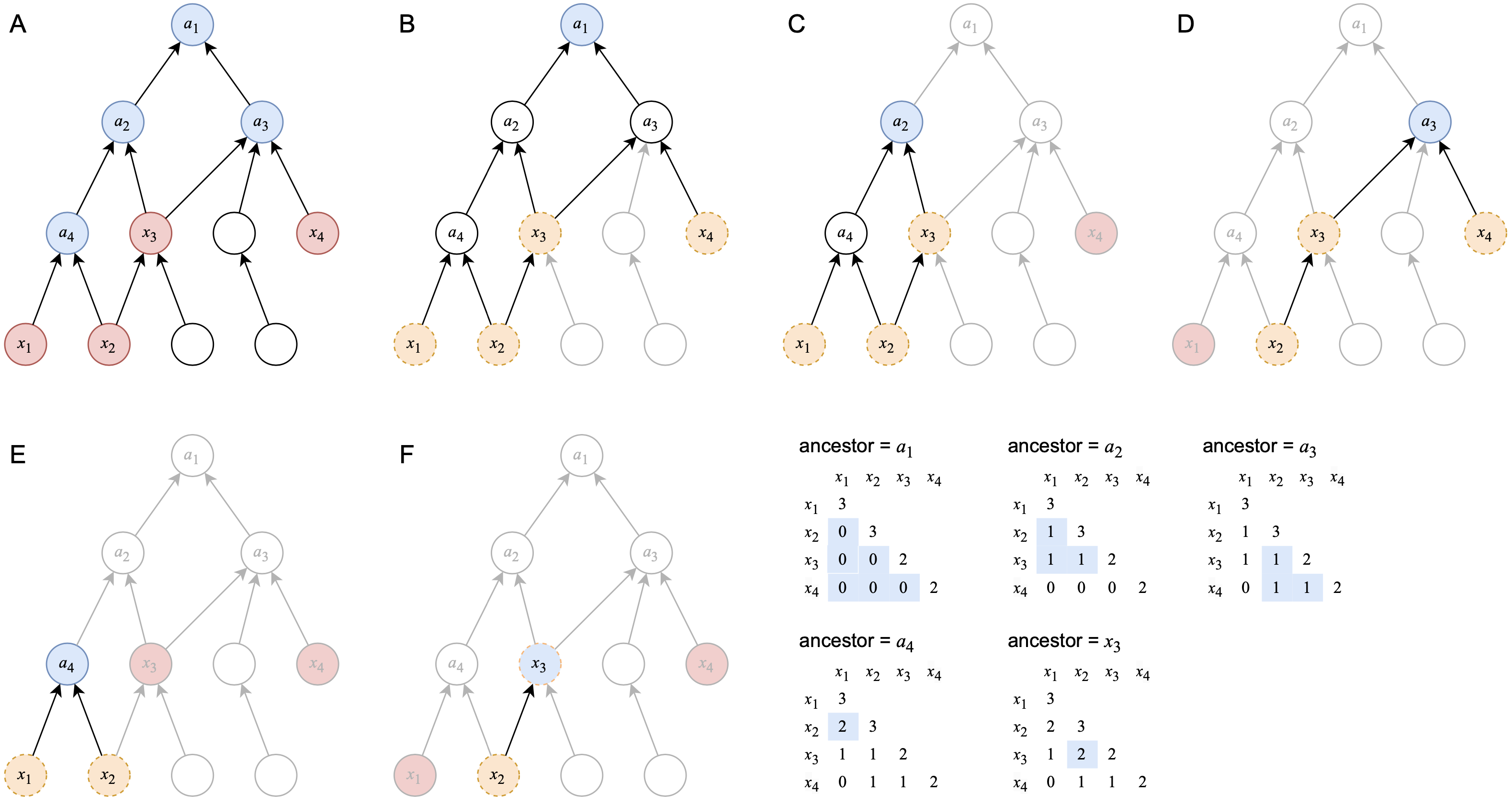

Supplement: Supplementary file 2 — Supplementary Material 2. The algorithm [file 12864_2024_10759_MOESM2_ESM.zip › suppl2_algorithm/f2.png]

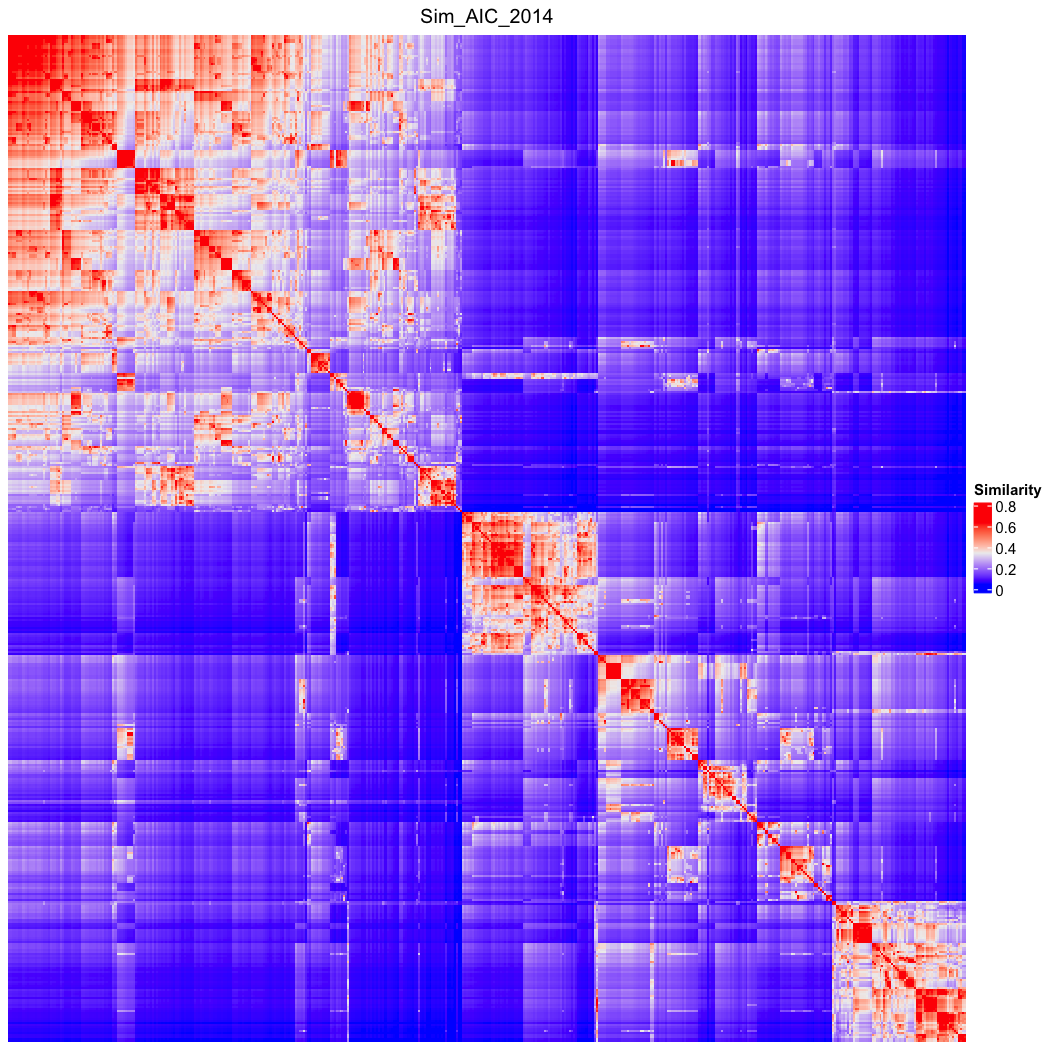

Supplement: Supplementary file 4 — Supplementary Material 4. Compare semantic similarity methods [file 12864_2024_10759_MOESM4_ESM.zip › suppl4_compare_sim_methods/image/go_bp_random_500_sim_Sim_AIC_2014.png]

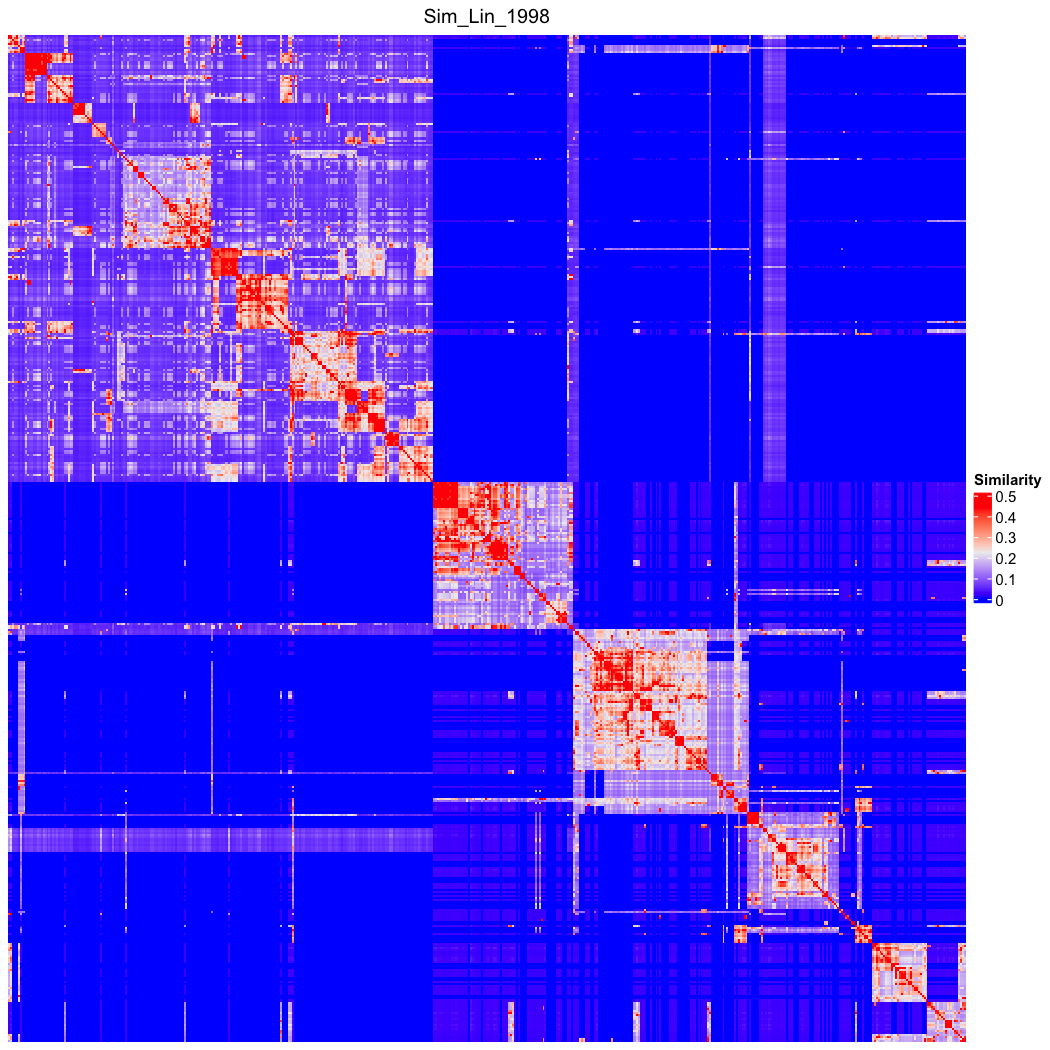

Supplement: Supplementary file 4 — Supplementary Material 4. Compare semantic similarity methods [file 12864_2024_10759_MOESM4_ESM.zip › suppl4_compare_sim_methods/image/go_bp_random_500_sim_Sim_Lin_1998_Lin_order.png]

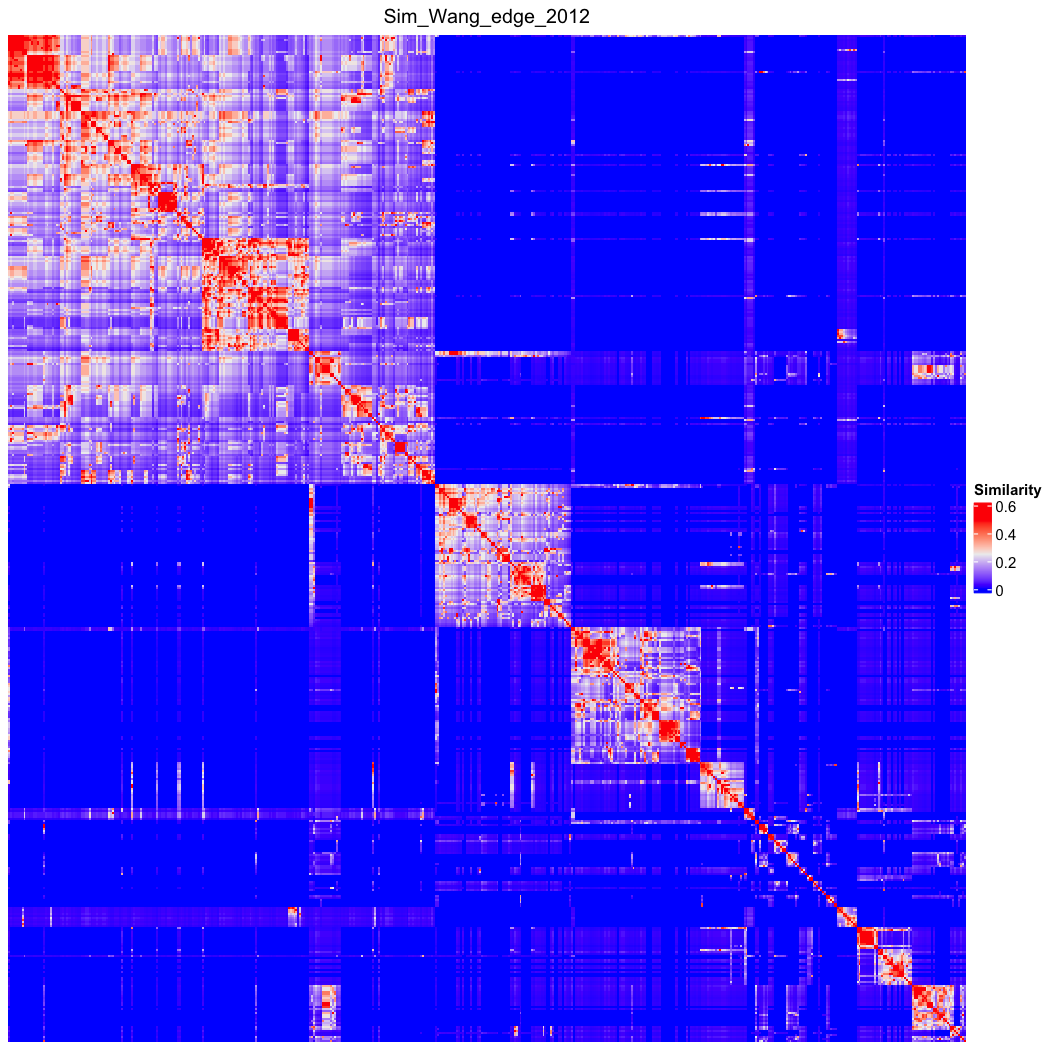

Supplement: Supplementary file 4 — Supplementary Material 4. Compare semantic similarity methods [file 12864_2024_10759_MOESM4_ESM.zip › suppl4_compare_sim_methods/image/go_bp_random_500_sim_Sim_Wang_edge_2012.png]

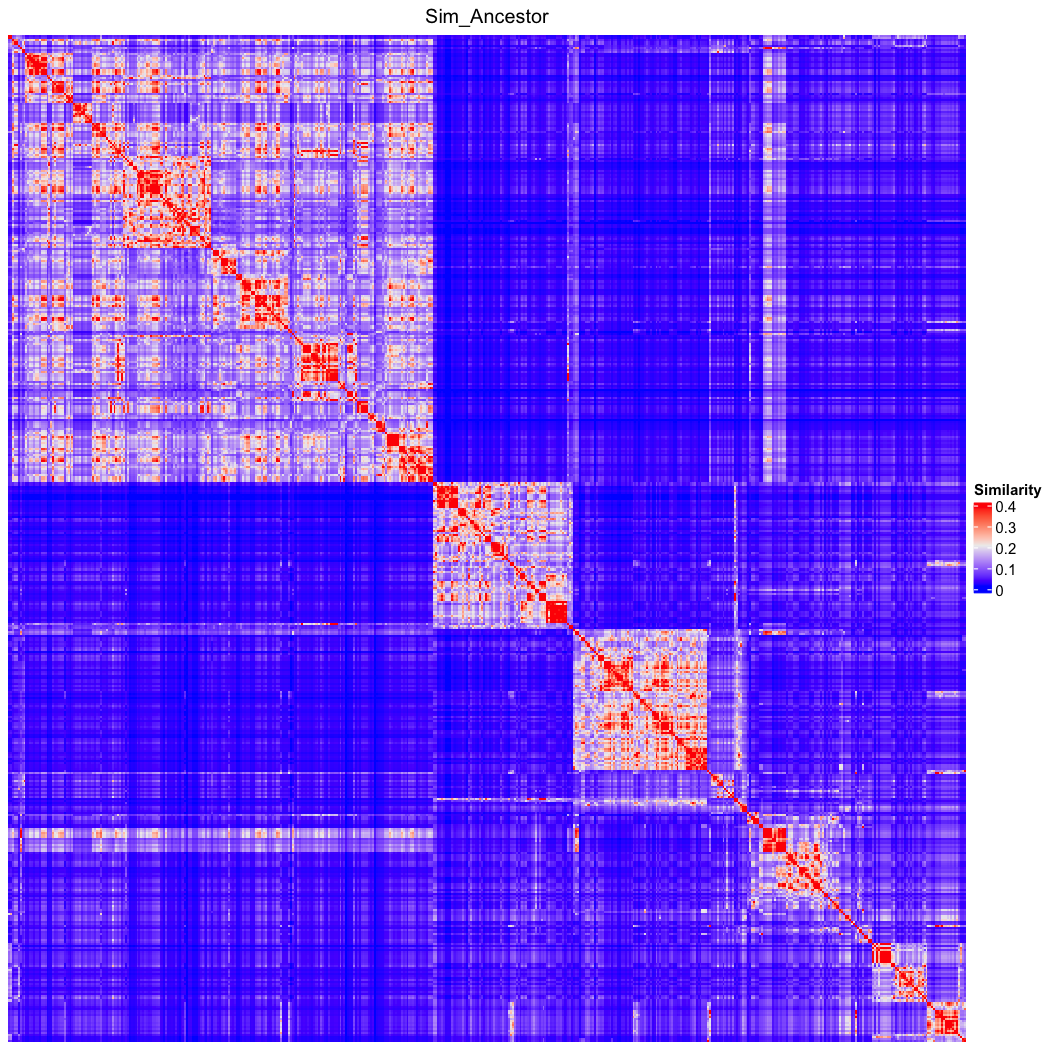

Supplement: Supplementary file 4 — Supplementary Material 4. Compare semantic similarity methods [file 12864_2024_10759_MOESM4_ESM.zip › suppl4_compare_sim_methods/image/go_bp_random_500_sim_Sim_Ancestor_Lin_order.png]

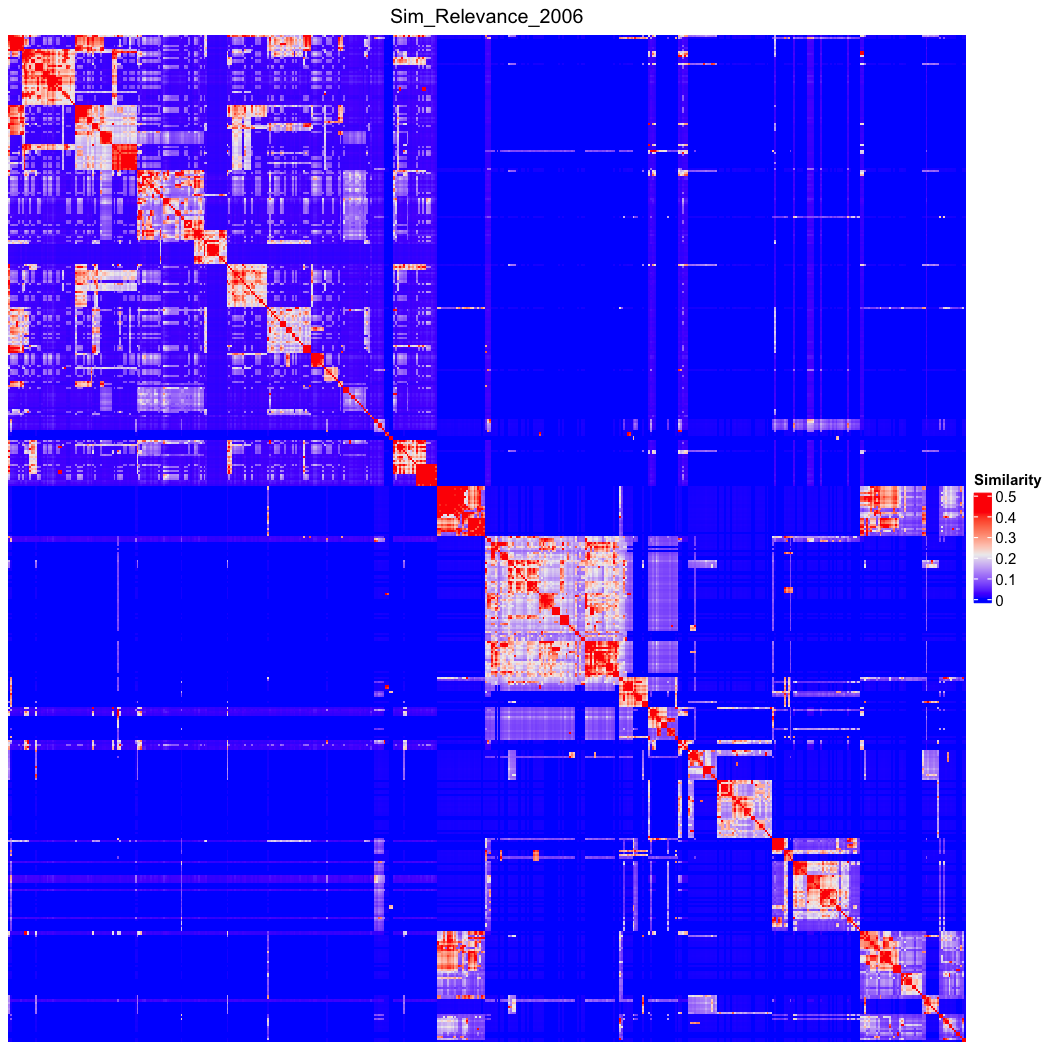

Supplement: Supplementary file 4 — Supplementary Material 4. Compare semantic similarity methods [file 12864_2024_10759_MOESM4_ESM.zip › suppl4_compare_sim_methods/image/go_bp_random_500_sim_Sim_Relevance_2006.png]

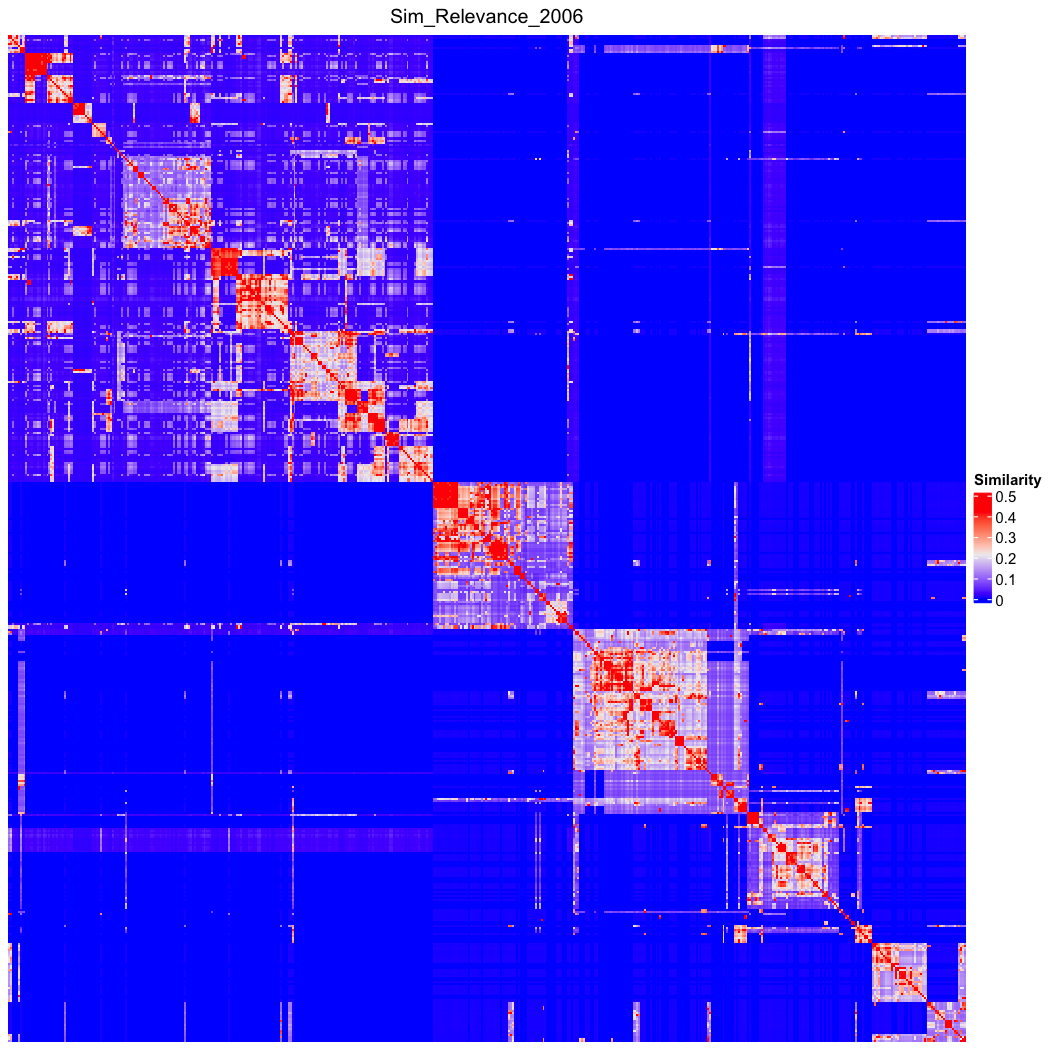

Supplement: Supplementary file 4 — Supplementary Material 4. Compare semantic similarity methods [file 12864_2024_10759_MOESM4_ESM.zip › suppl4_compare_sim_methods/image/go_bp_random_500_sim_Sim_Relevance_2006_Lin_order.png]

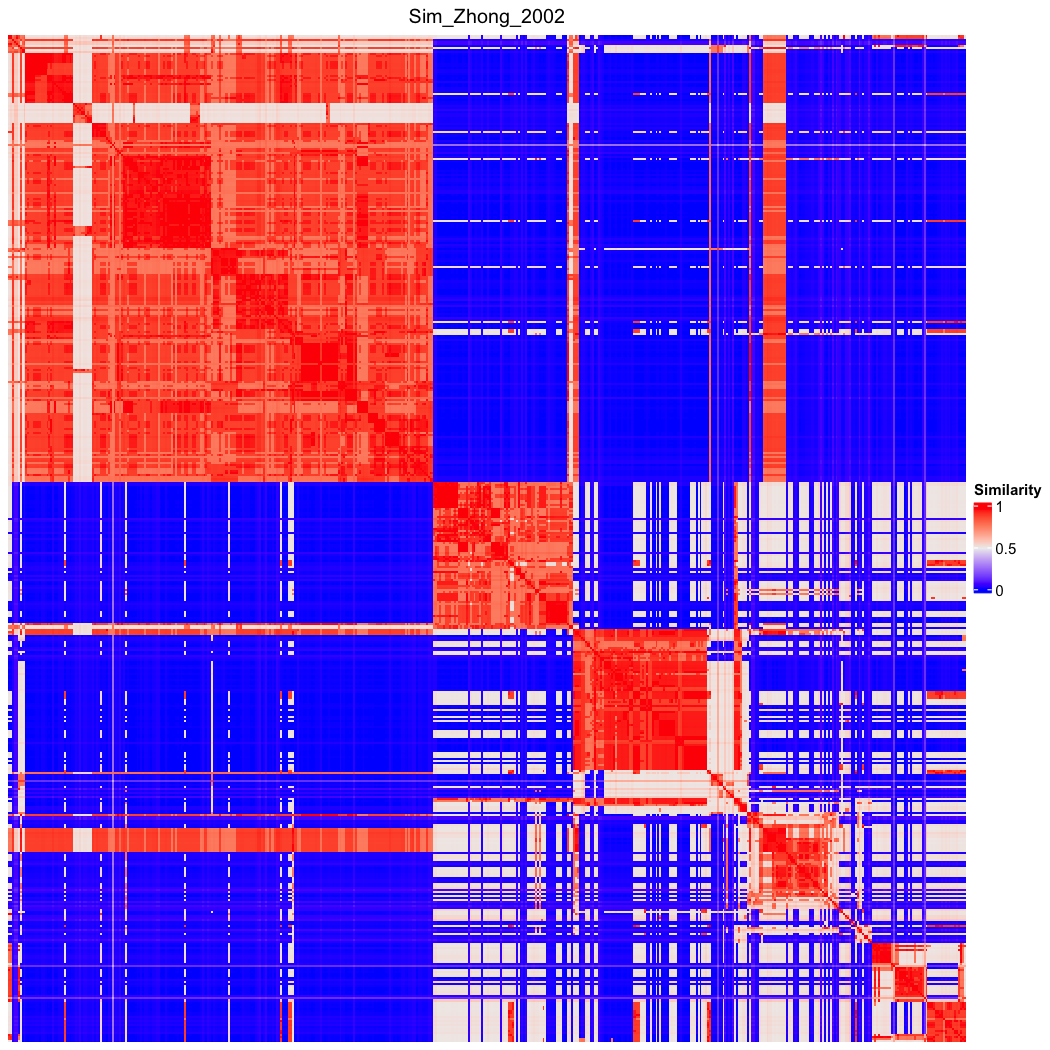

Supplement: Supplementary file 4 — Supplementary Material 4. Compare semantic similarity methods [file 12864_2024_10759_MOESM4_ESM.zip › suppl4_compare_sim_methods/image/go_bp_random_500_sim_Sim_Zhong_2002_Lin_order.png]

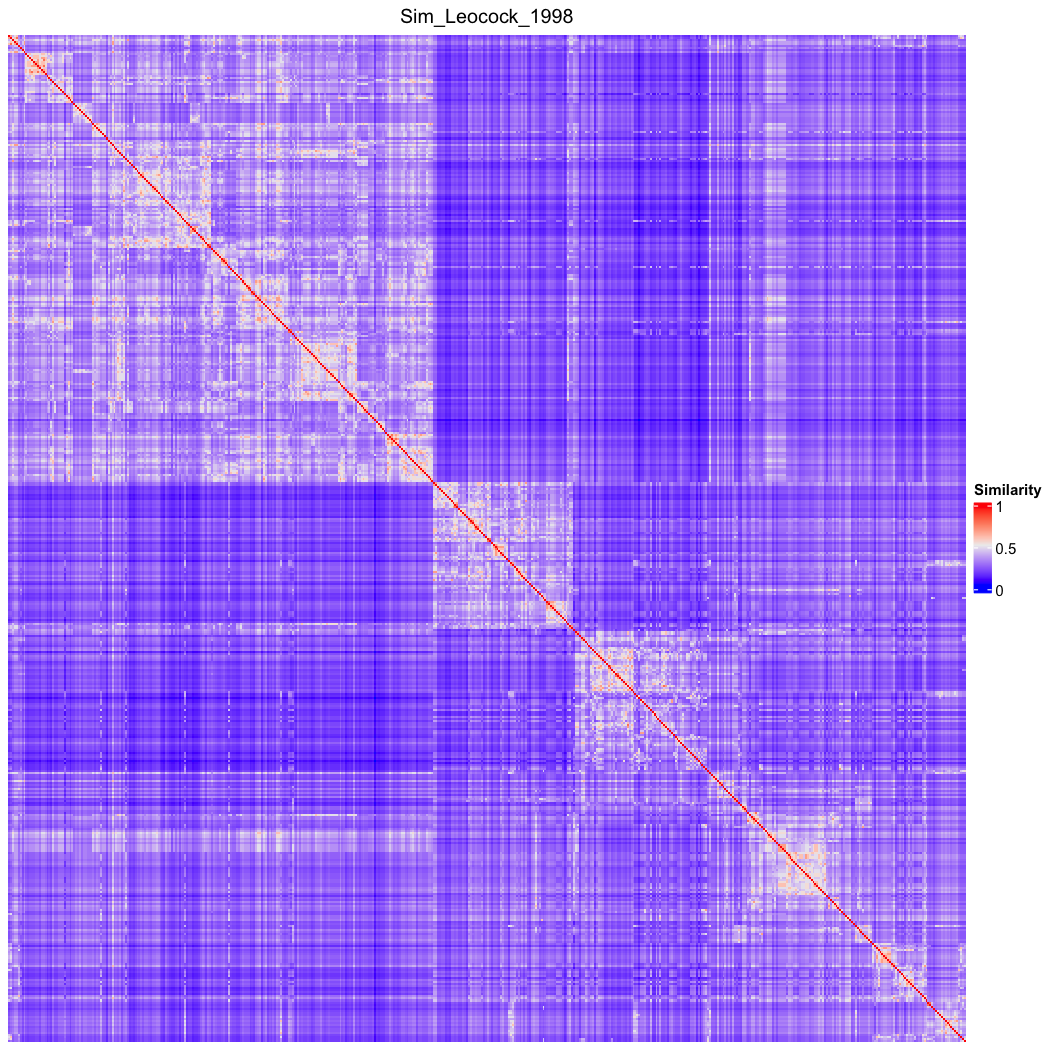

Supplement: Supplementary file 4 — Supplementary Material 4. Compare semantic similarity methods [file 12864_2024_10759_MOESM4_ESM.zip › suppl4_compare_sim_methods/image/go_bp_random_500_sim_Sim_Leocock_1998_Lin_order.png]

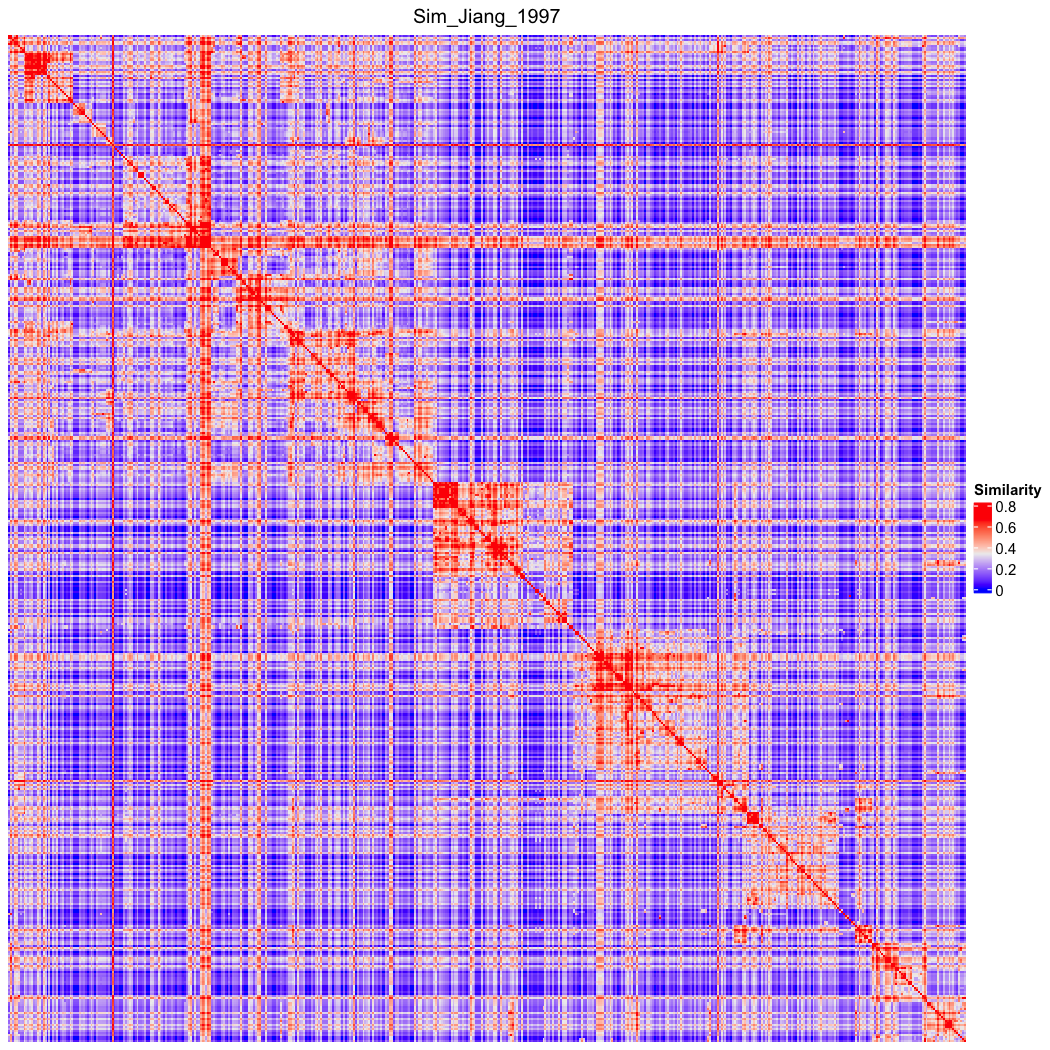

Supplement: Supplementary file 4 — Supplementary Material 4. Compare semantic similarity methods [file 12864_2024_10759_MOESM4_ESM.zip › suppl4_compare_sim_methods/image/go_bp_random_500_sim_Sim_Jiang_1997_Lin_order.png]

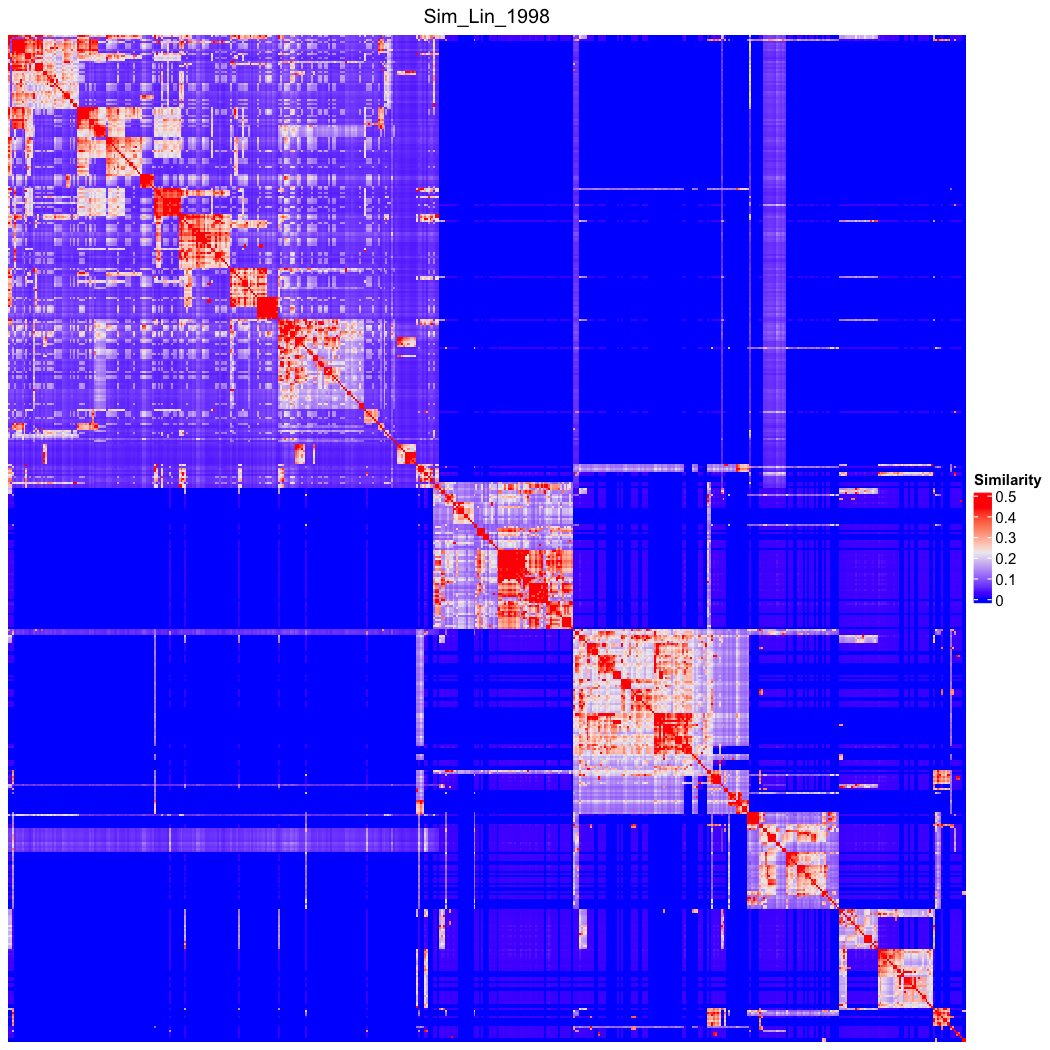

Supplement: Supplementary file 4 — Supplementary Material 4. Compare semantic similarity methods [file 12864_2024_10759_MOESM4_ESM.zip › suppl4_compare_sim_methods/image/go_bp_random_500_sim_Sim_Lin_1998.png]

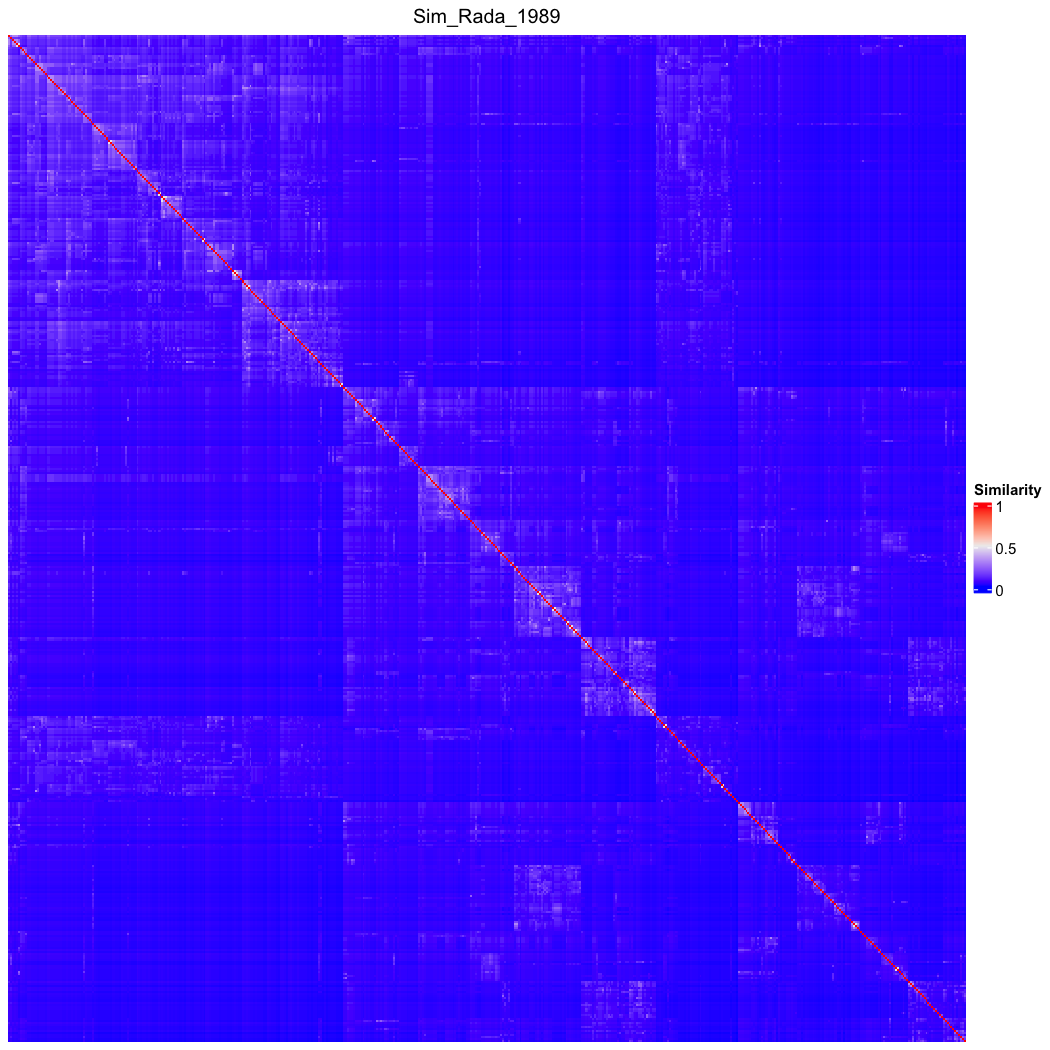

Supplement: Supplementary file 4 — Supplementary Material 4. Compare semantic similarity methods [file 12864_2024_10759_MOESM4_ESM.zip › suppl4_compare_sim_methods/image/go_bp_random_500_sim_Sim_Rada_1989.png]

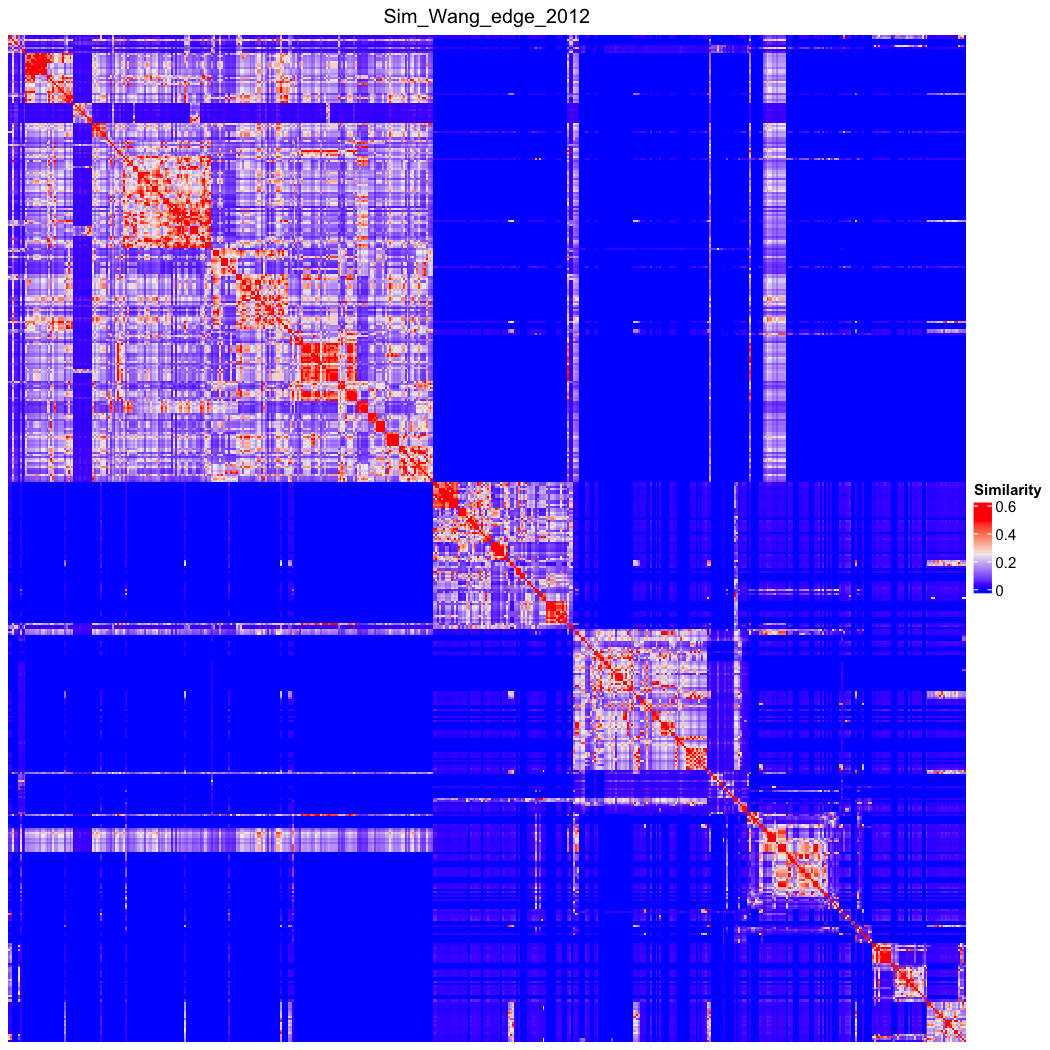

Supplement: Supplementary file 4 — Supplementary Material 4. Compare semantic similarity methods [file 12864_2024_10759_MOESM4_ESM.zip › suppl4_compare_sim_methods/image/go_bp_random_500_sim_Sim_Wang_edge_2012_Lin_order.png]

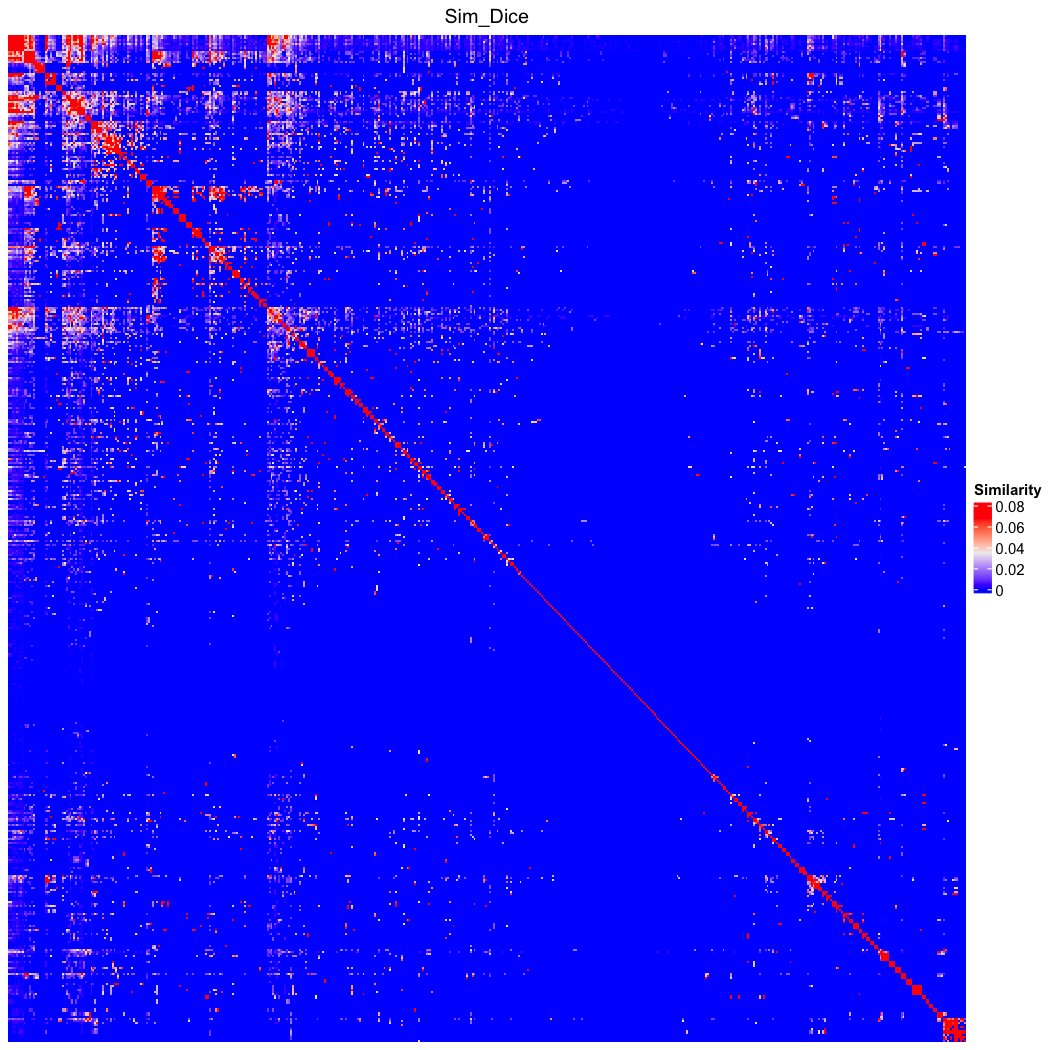

Supplement: Supplementary file 4 — Supplementary Material 4. Compare semantic similarity methods [file 12864_2024_10759_MOESM4_ESM.zip › suppl4_compare_sim_methods/image/go_bp_random_500_sim_Sim_Dice.png]

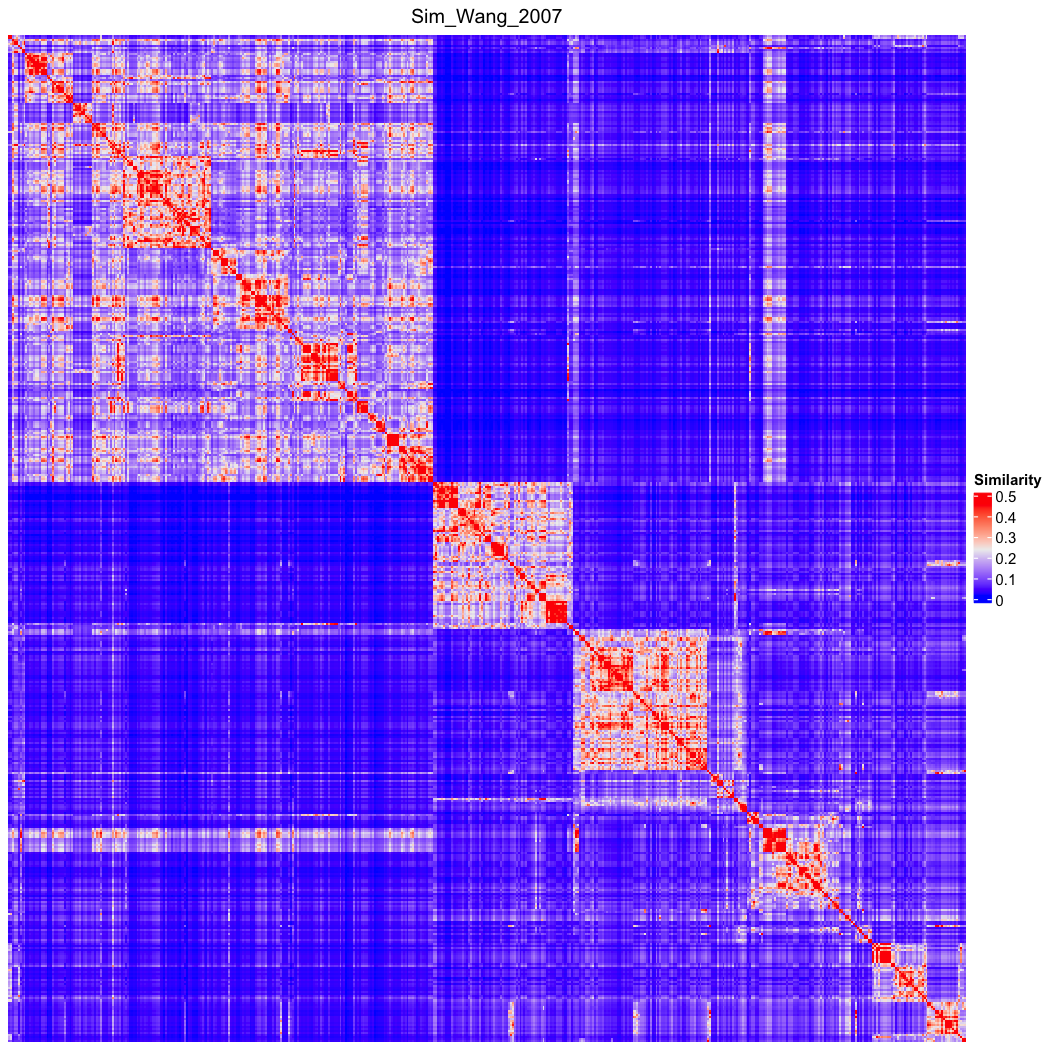

Supplement: Supplementary file 4 — Supplementary Material 4. Compare semantic similarity methods [file 12864_2024_10759_MOESM4_ESM.zip › suppl4_compare_sim_methods/image/go_bp_random_500_sim_Sim_Wang_2007_Lin_order.png]

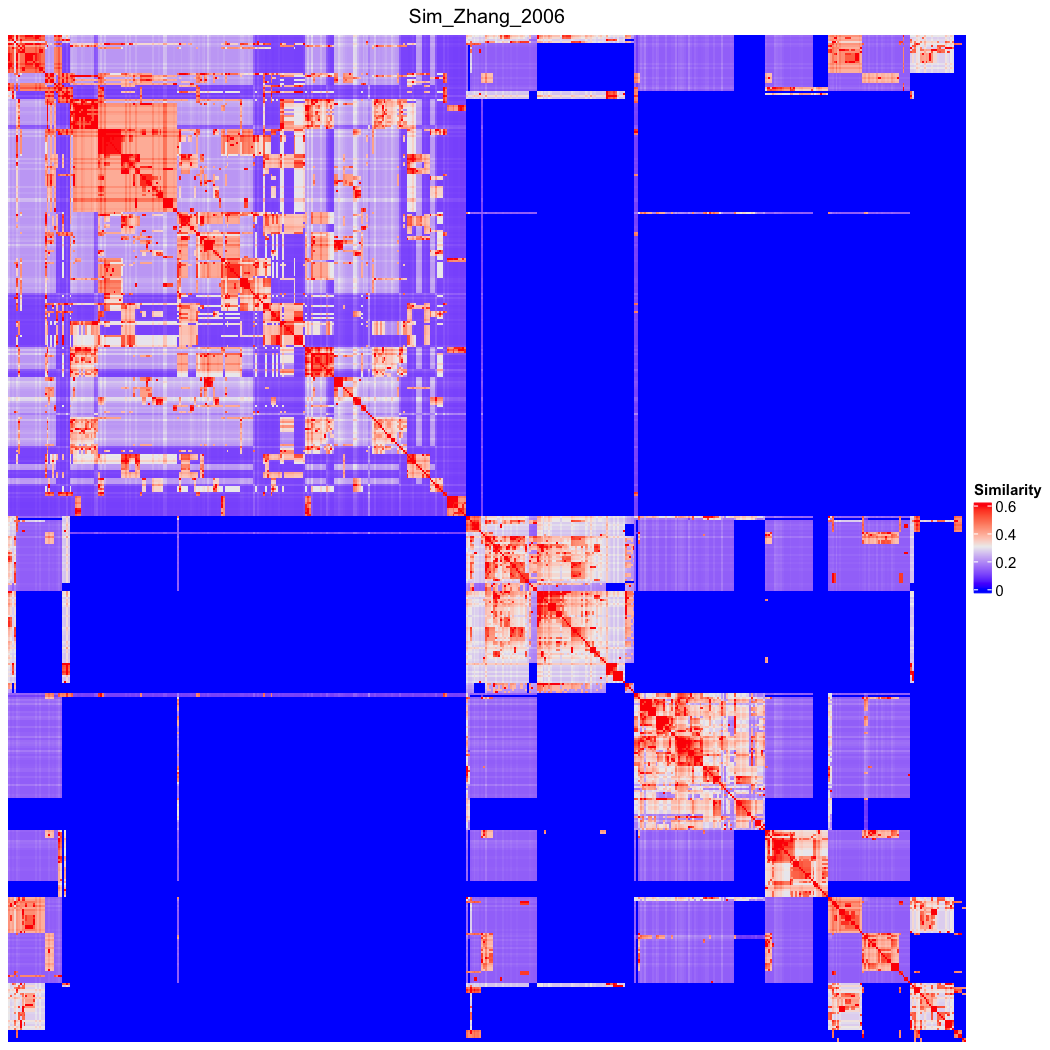

Supplement: Supplementary file 4 — Supplementary Material 4. Compare semantic similarity methods [file 12864_2024_10759_MOESM4_ESM.zip › suppl4_compare_sim_methods/image/go_bp_random_500_sim_Sim_Zhang_2006.png]

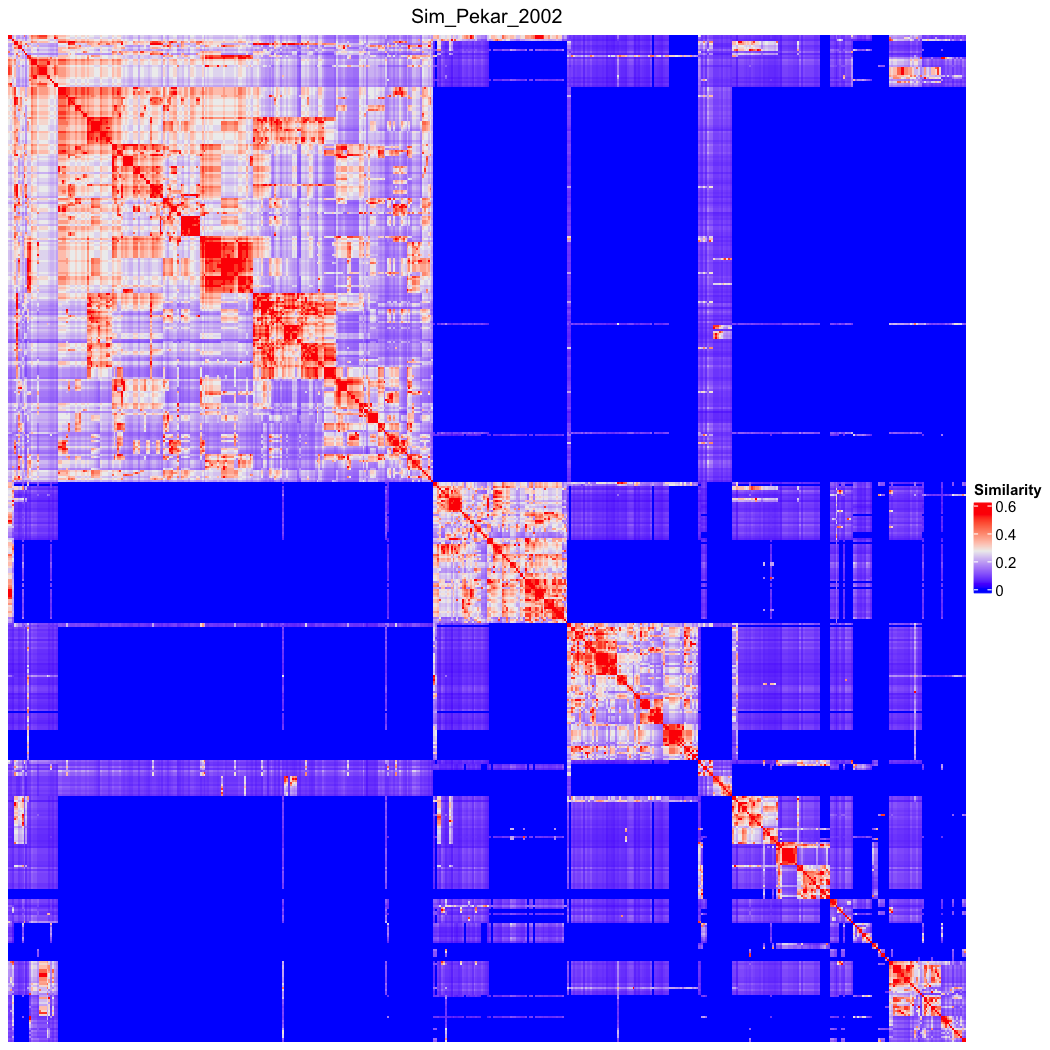

Supplement: Supplementary file 4 — Supplementary Material 4. Compare semantic similarity methods [file 12864_2024_10759_MOESM4_ESM.zip › suppl4_compare_sim_methods/image/go_bp_random_500_sim_Sim_Pekar_2002.png]

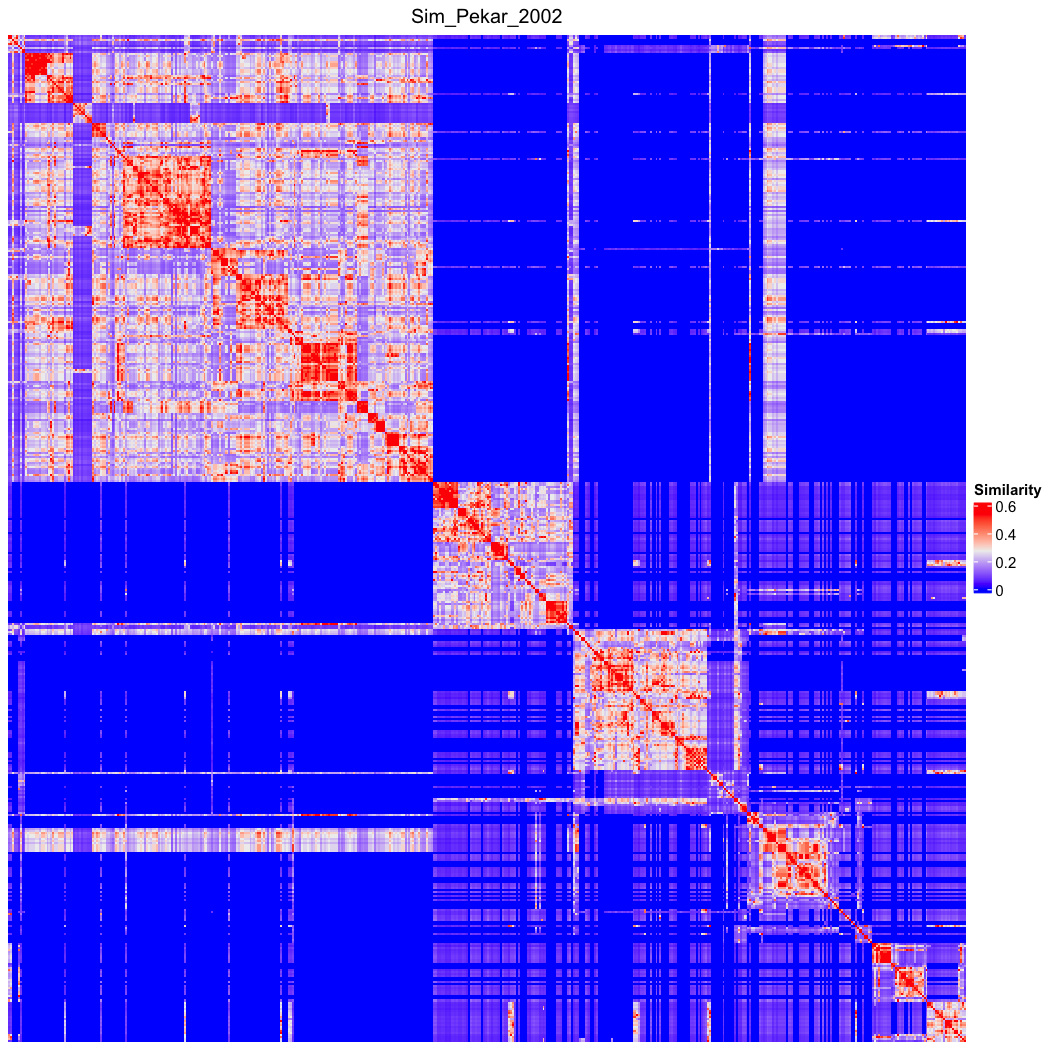

Supplement: Supplementary file 4 — Supplementary Material 4. Compare semantic similarity methods [file 12864_2024_10759_MOESM4_ESM.zip › suppl4_compare_sim_methods/image/go_bp_random_500_sim_Sim_Pekar_2002_Lin_order.png]

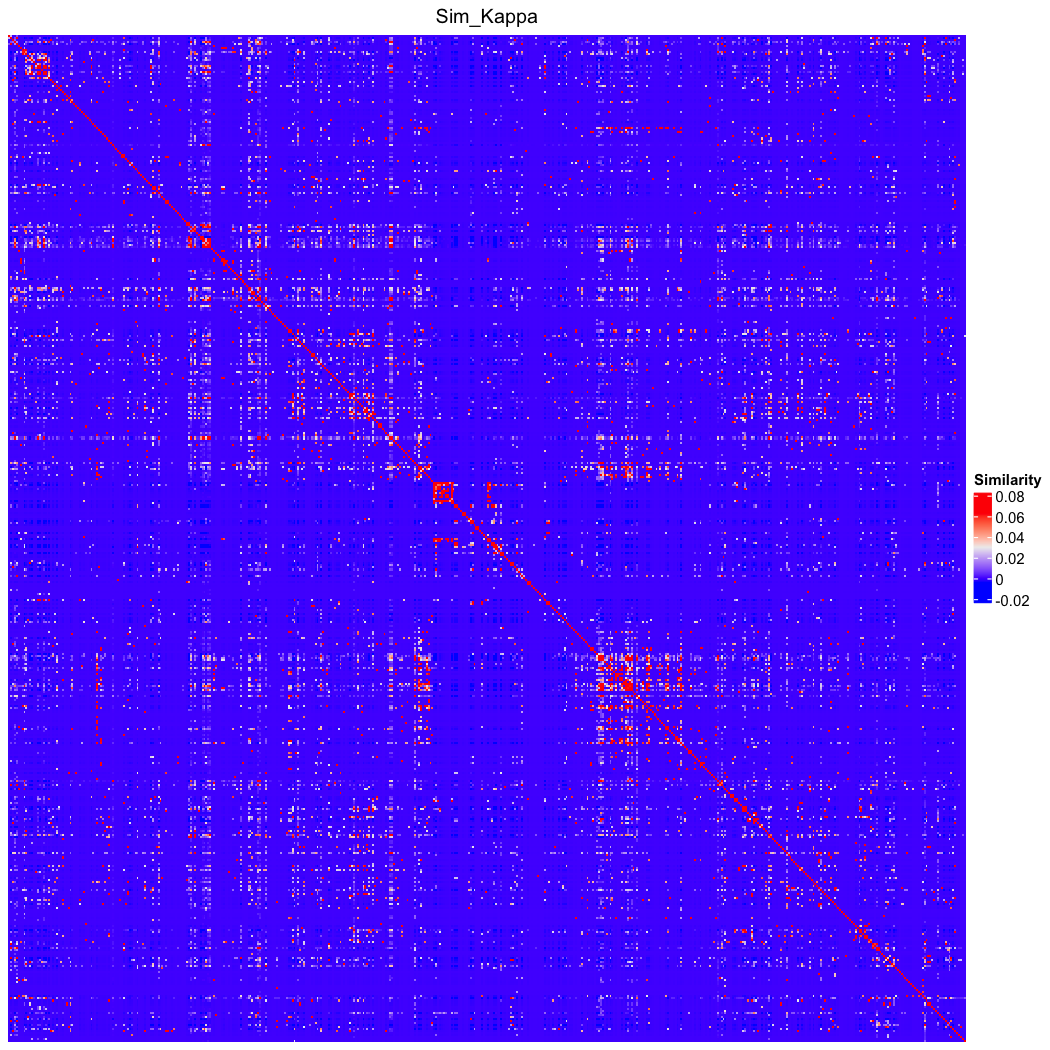

Supplement: Supplementary file 4 — Supplementary Material 4. Compare semantic similarity methods [file 12864_2024_10759_MOESM4_ESM.zip › suppl4_compare_sim_methods/image/go_bp_random_500_sim_Sim_Kappa_Lin_order.png]

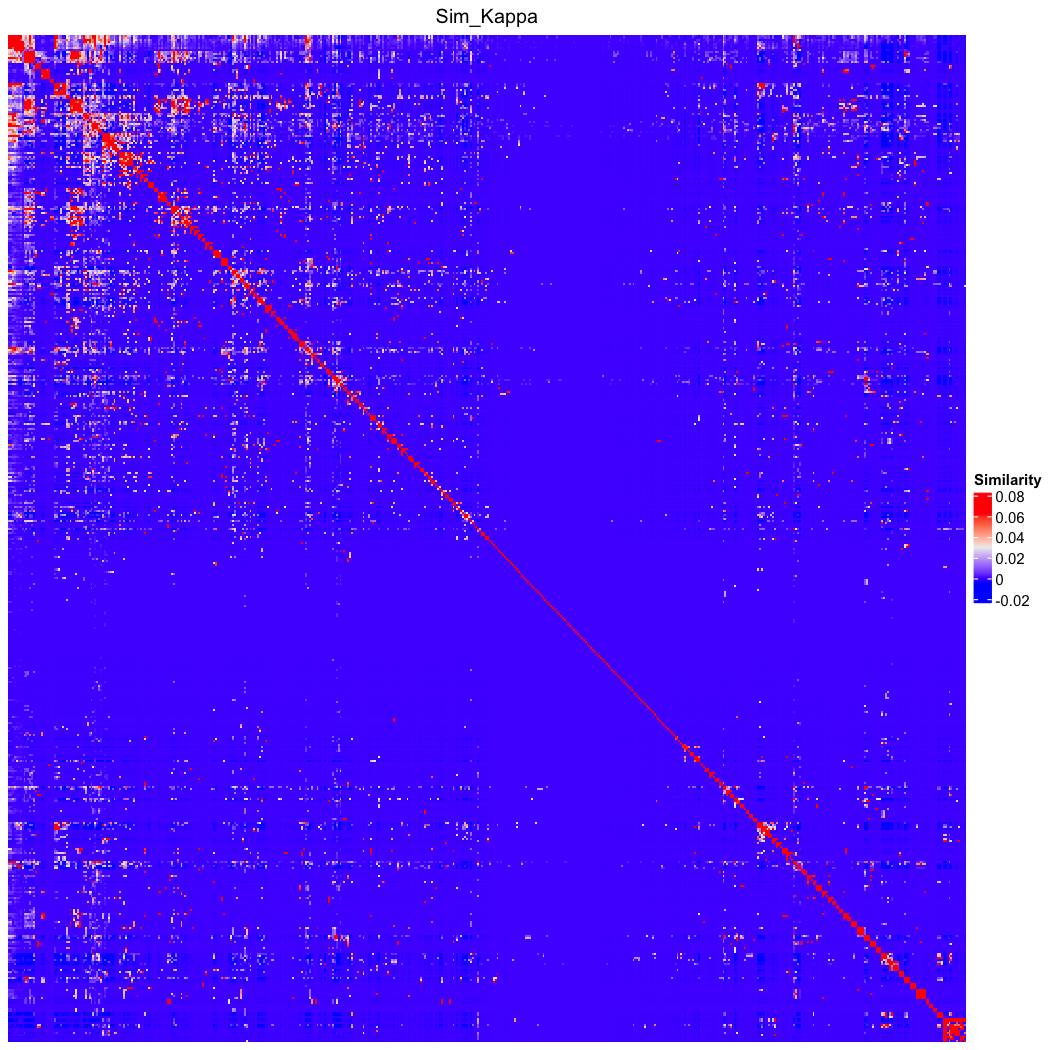

Supplement: Supplementary file 4 — Supplementary Material 4. Compare semantic similarity methods [file 12864_2024_10759_MOESM4_ESM.zip › suppl4_compare_sim_methods/image/go_bp_random_500_sim_Sim_Kappa.png]

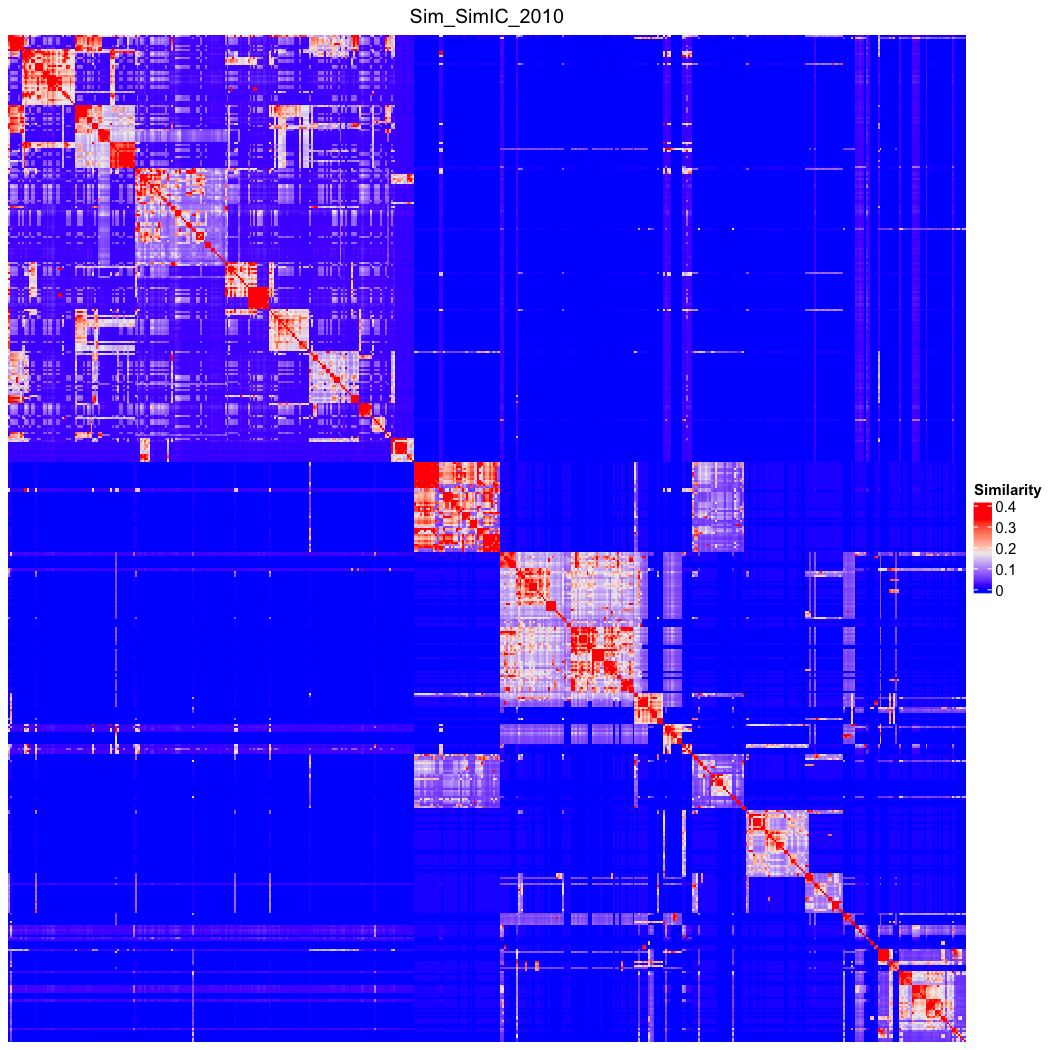

Supplement: Supplementary file 4 — Supplementary Material 4. Compare semantic similarity methods [file 12864_2024_10759_MOESM4_ESM.zip › suppl4_compare_sim_methods/image/go_bp_random_500_sim_Sim_SimIC_2010.png]

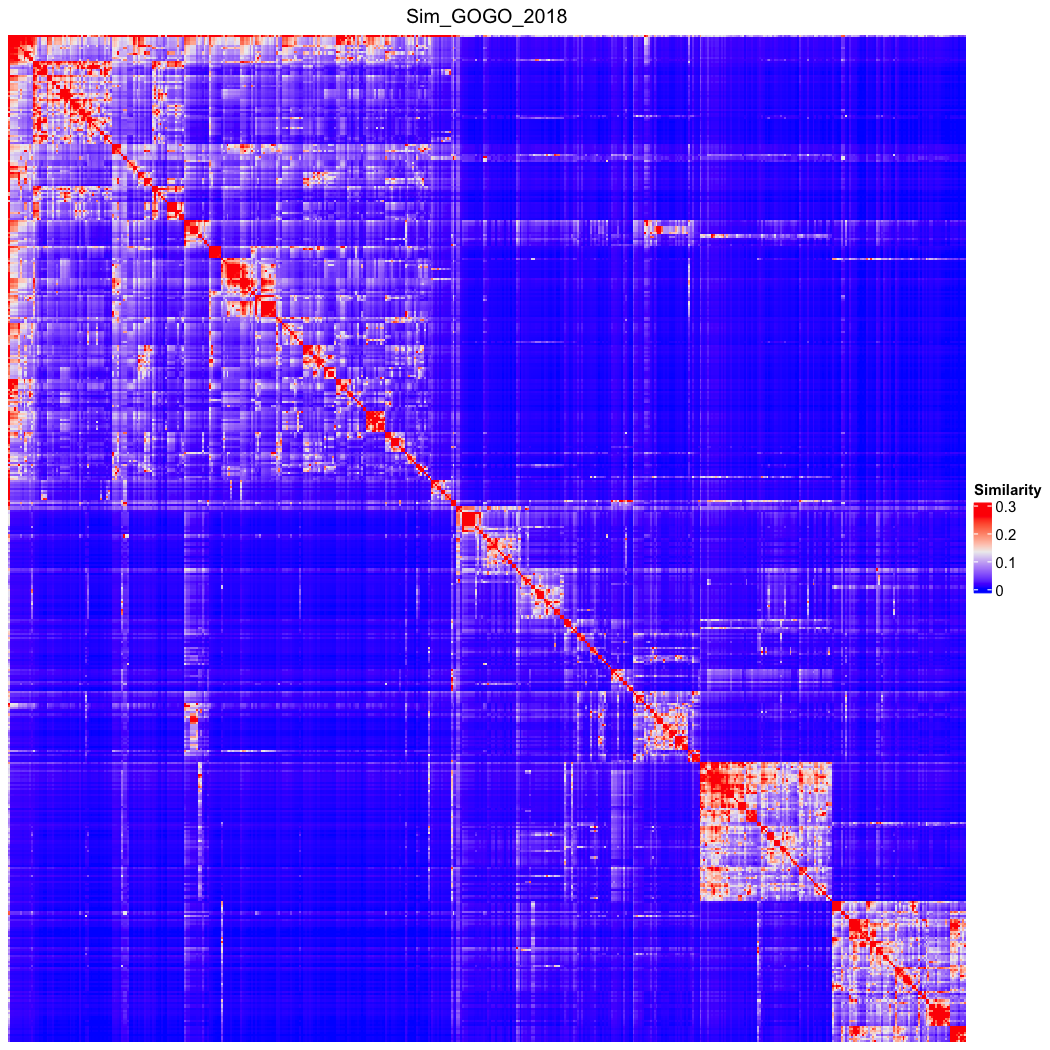

Supplement: Supplementary file 4 — Supplementary Material 4. Compare semantic similarity methods [file 12864_2024_10759_MOESM4_ESM.zip › suppl4_compare_sim_methods/image/go_bp_random_500_sim_Sim_GOGO_2018.png]

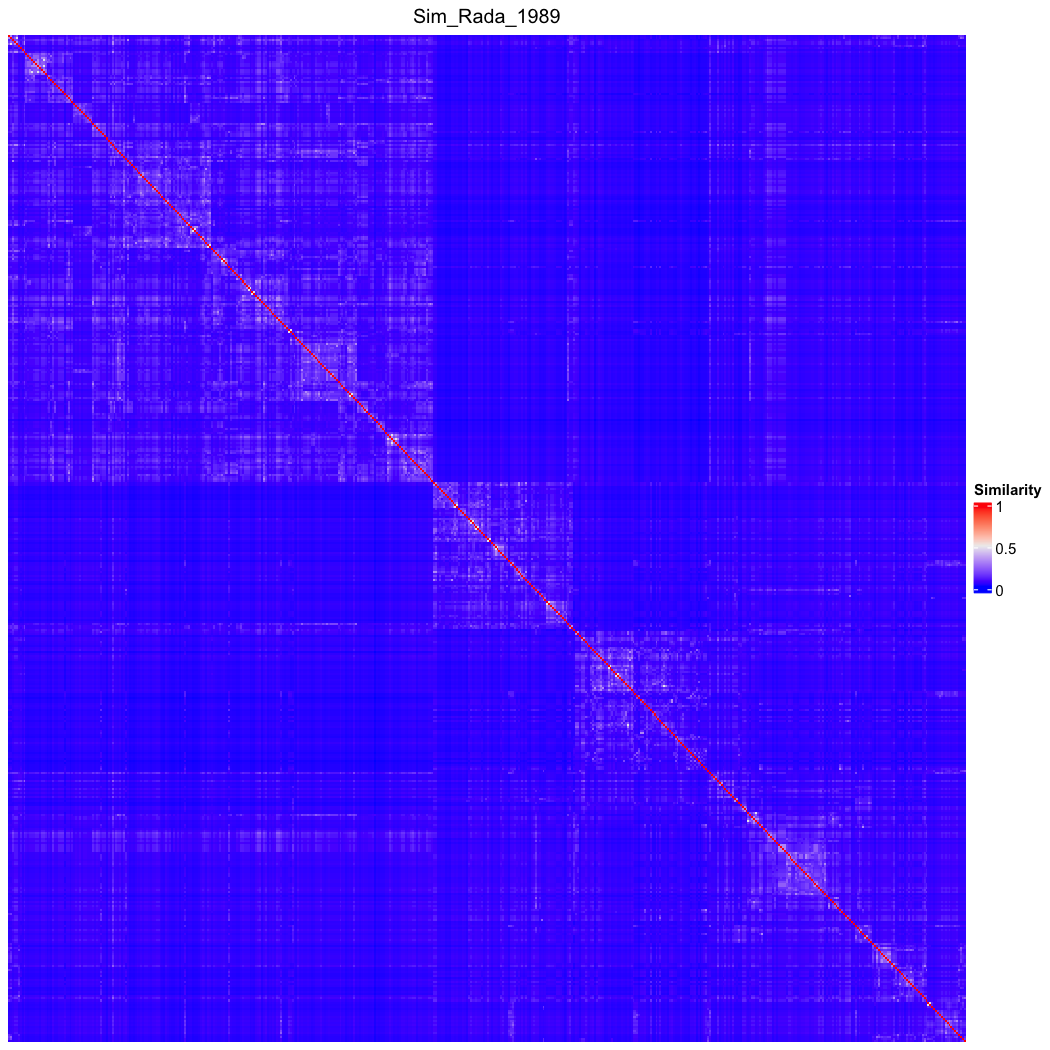

Supplement: Supplementary file 4 — Supplementary Material 4. Compare semantic similarity methods [file 12864_2024_10759_MOESM4_ESM.zip › suppl4_compare_sim_methods/image/go_bp_random_500_sim_Sim_Rada_1989_Lin_order.png]

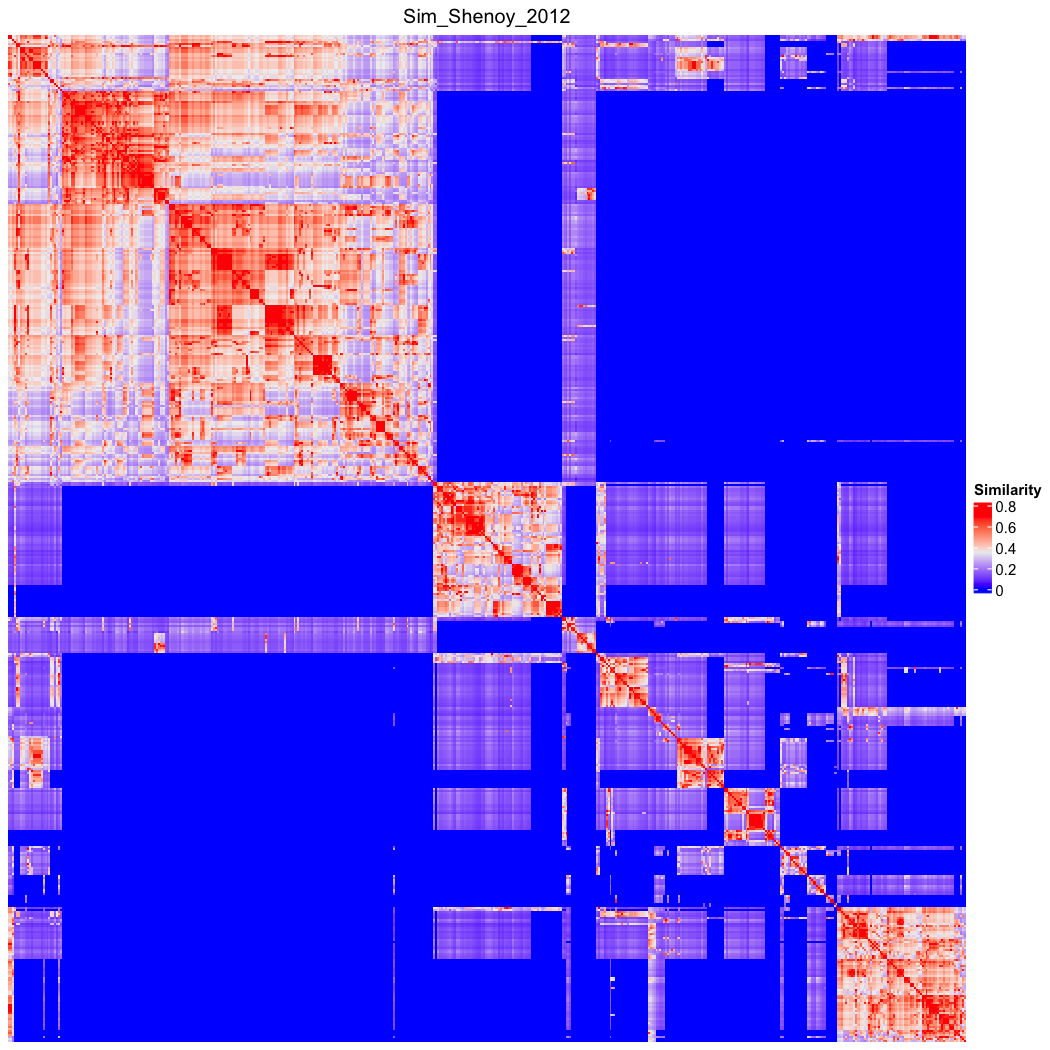

Supplement: Supplementary file 4 — Supplementary Material 4. Compare semantic similarity methods [file 12864_2024_10759_MOESM4_ESM.zip › suppl4_compare_sim_methods/image/go_bp_random_500_sim_Sim_Shenoy_2012.png]

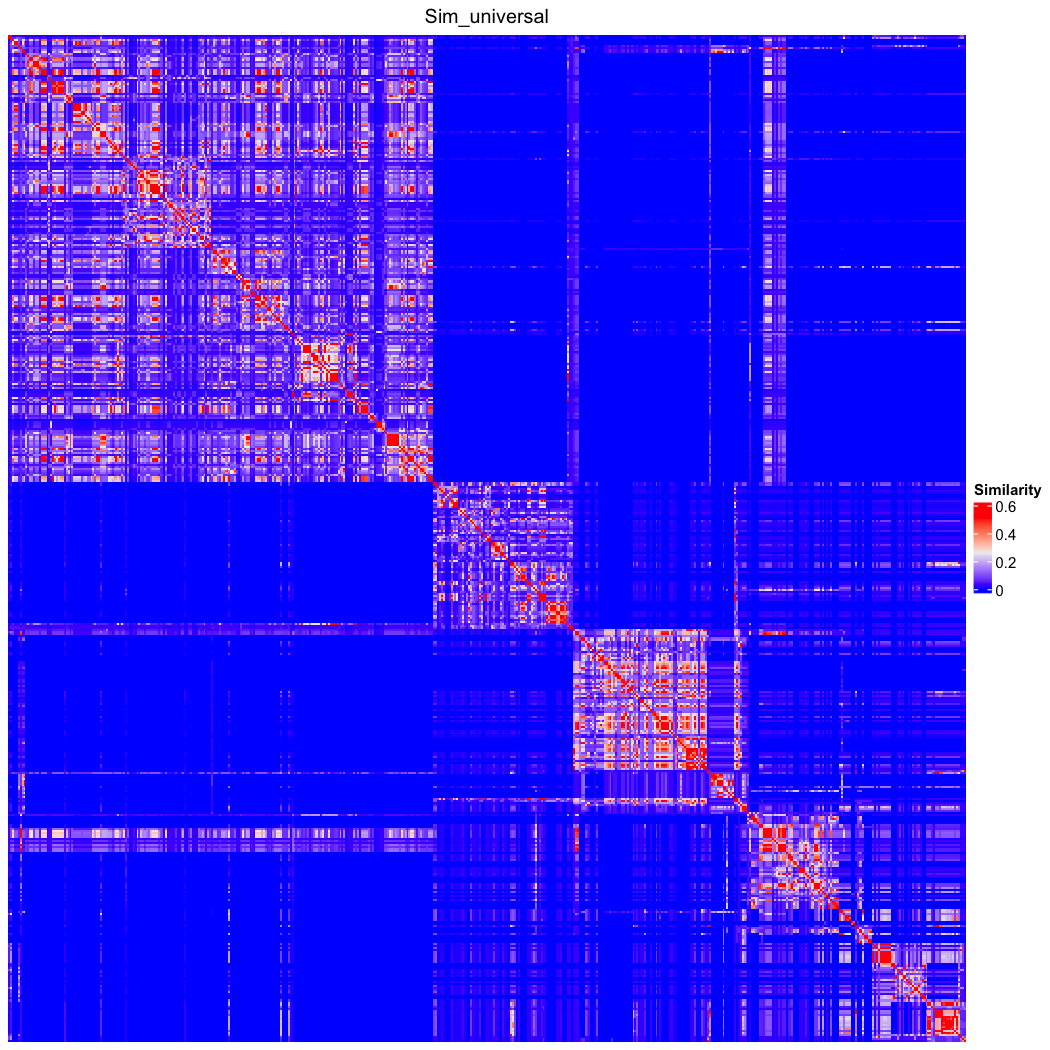

Supplement: Supplementary file 4 — Supplementary Material 4. Compare semantic similarity methods [file 12864_2024_10759_MOESM4_ESM.zip › suppl4_compare_sim_methods/image/go_bp_random_500_sim_Sim_universal_Lin_order.png]

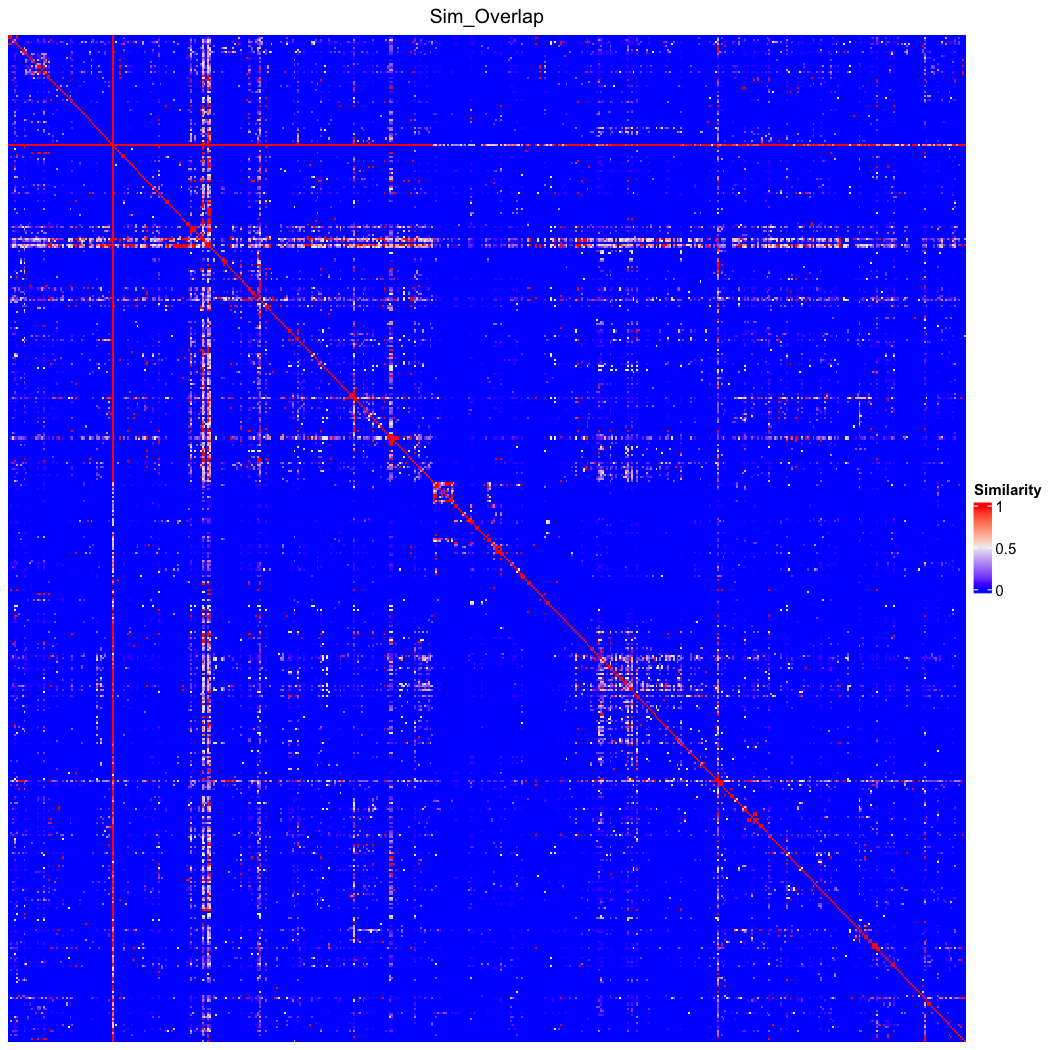

Supplement: Supplementary file 4 — Supplementary Material 4. Compare semantic similarity methods [file 12864_2024_10759_MOESM4_ESM.zip › suppl4_compare_sim_methods/image/go_bp_random_500_sim_Sim_Overlap_Lin_order.png]

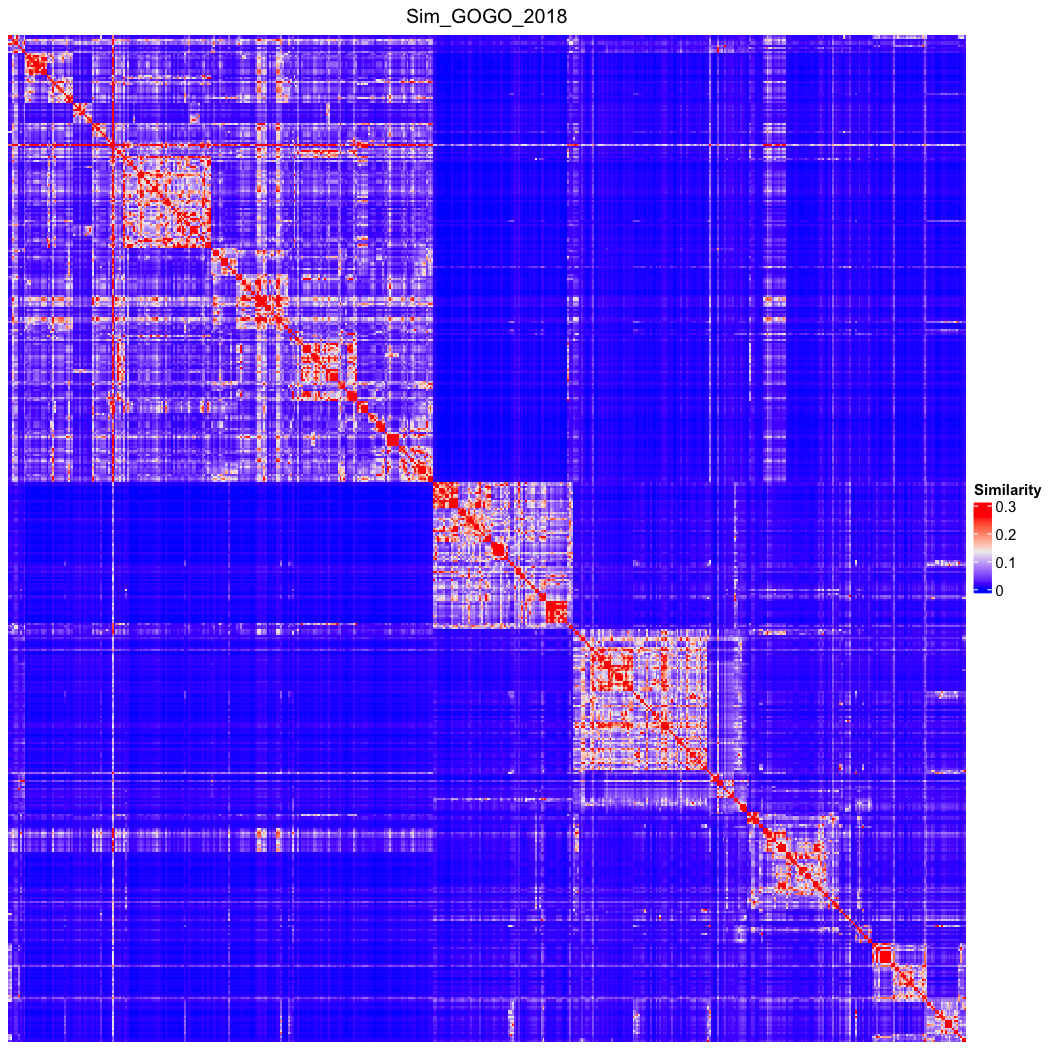

Supplement: Supplementary file 4 — Supplementary Material 4. Compare semantic similarity methods [file 12864_2024_10759_MOESM4_ESM.zip › suppl4_compare_sim_methods/image/go_bp_random_500_sim_Sim_GOGO_2018_Lin_order.png]

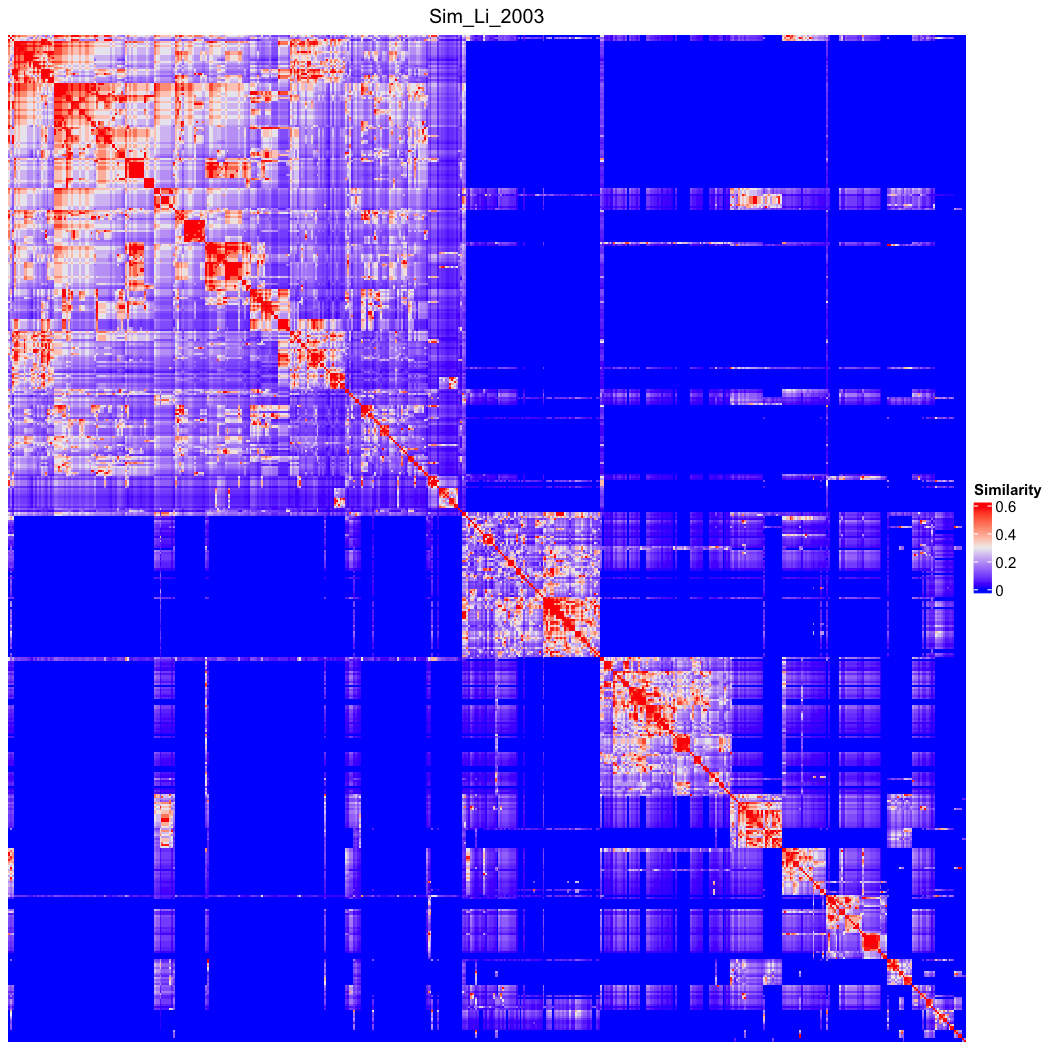

Supplement: Supplementary file 4 — Supplementary Material 4. Compare semantic similarity methods [file 12864_2024_10759_MOESM4_ESM.zip › suppl4_compare_sim_methods/image/go_bp_random_500_sim_Sim_Li_2003.png]

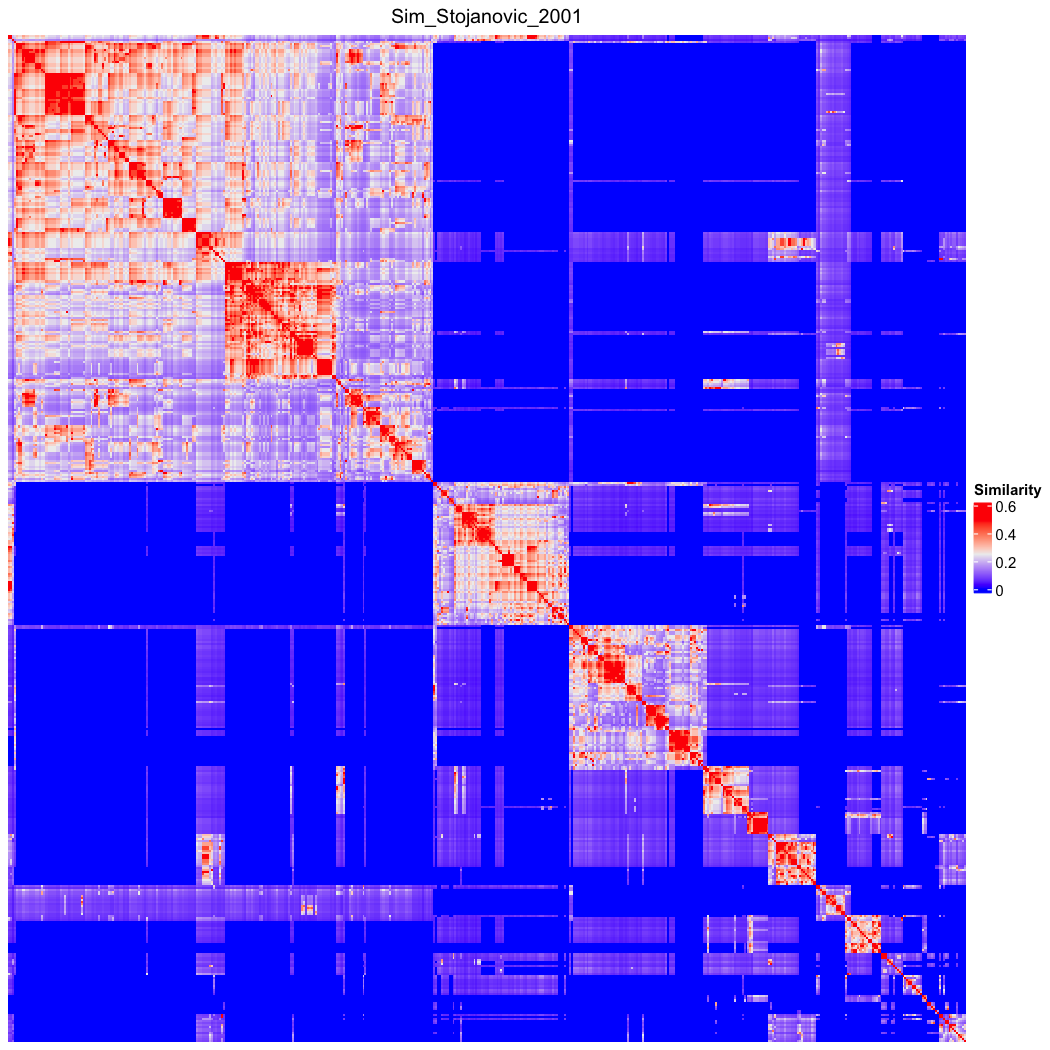

Supplement: Supplementary file 4 — Supplementary Material 4. Compare semantic similarity methods [file 12864_2024_10759_MOESM4_ESM.zip › suppl4_compare_sim_methods/image/go_bp_random_500_sim_Sim_Stojanovic_2001.png]

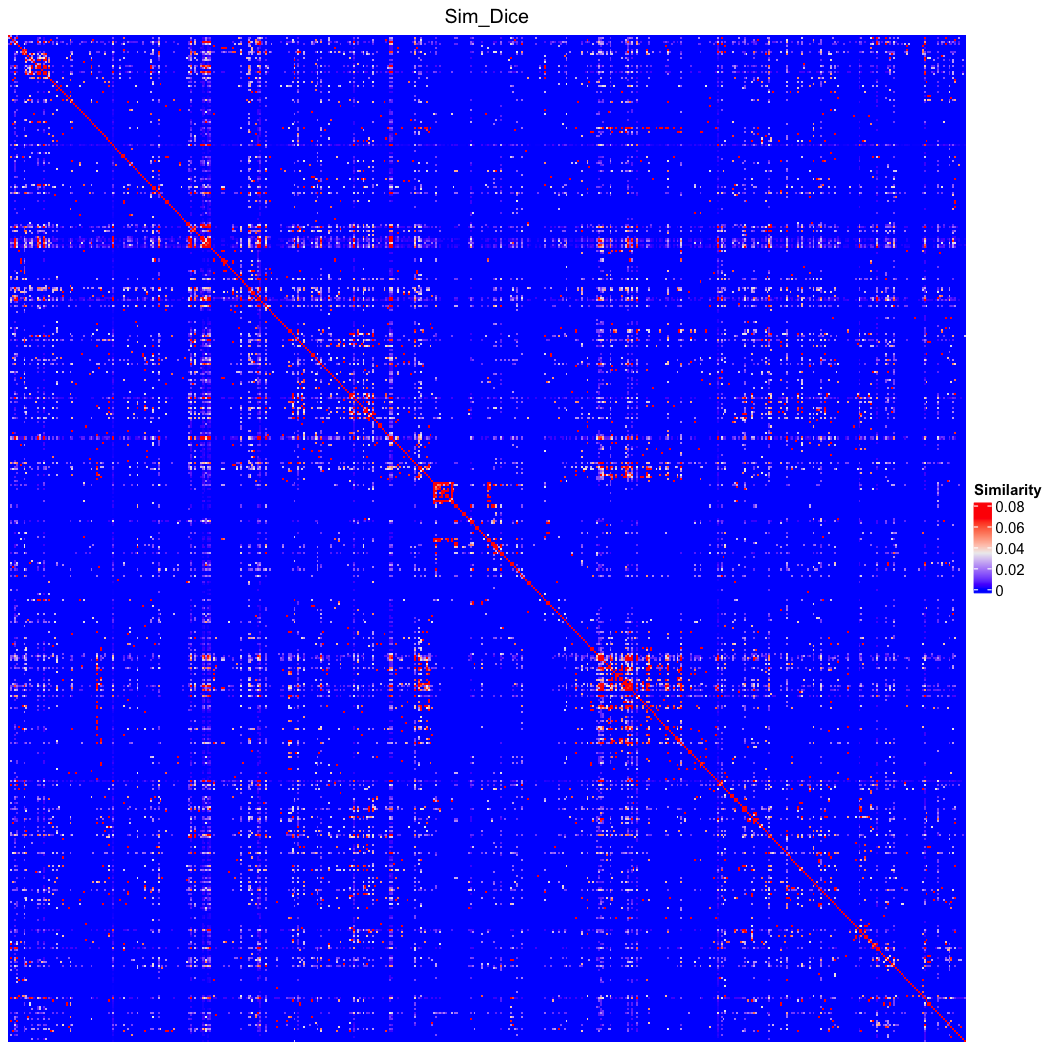

Supplement: Supplementary file 4 — Supplementary Material 4. Compare semantic similarity methods [file 12864_2024_10759_MOESM4_ESM.zip › suppl4_compare_sim_methods/image/go_bp_random_500_sim_Sim_Dice_Lin_order.png]

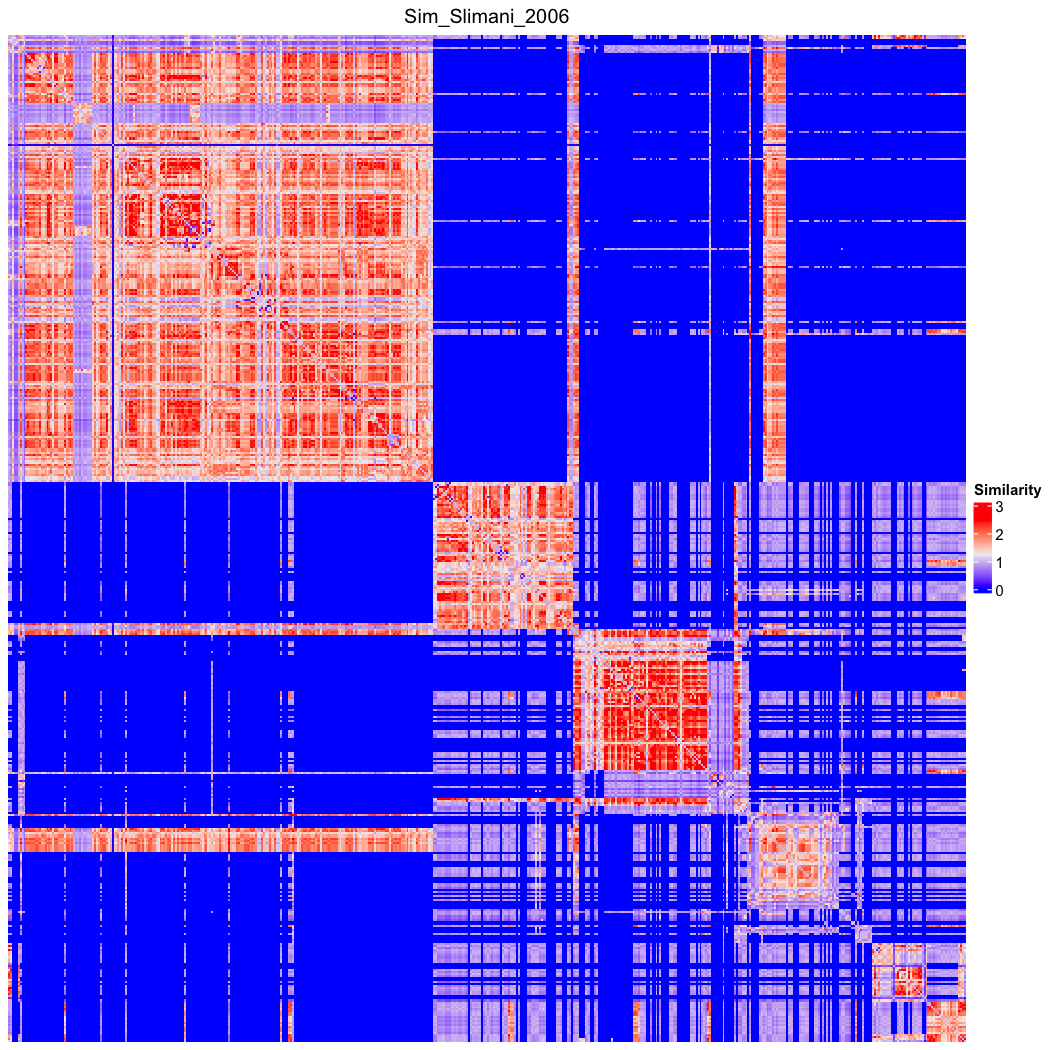

Supplement: Supplementary file 4 — Supplementary Material 4. Compare semantic similarity methods [file 12864_2024_10759_MOESM4_ESM.zip › suppl4_compare_sim_methods/image/go_bp_random_500_sim_Sim_Slimani_2006_Lin_order.png]

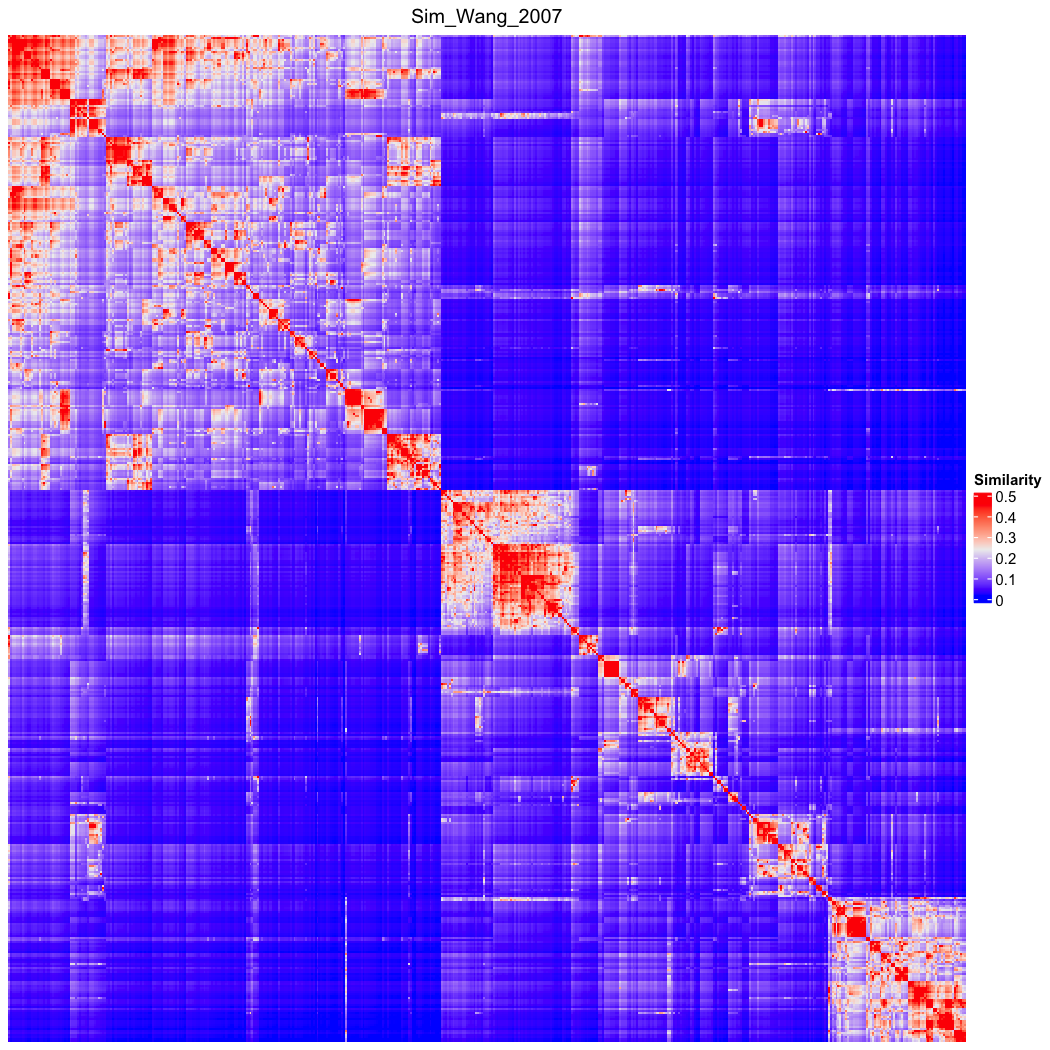

Supplement: Supplementary file 4 — Supplementary Material 4. Compare semantic similarity methods [file 12864_2024_10759_MOESM4_ESM.zip › suppl4_compare_sim_methods/image/go_bp_random_500_sim_Sim_Wang_2007.png]

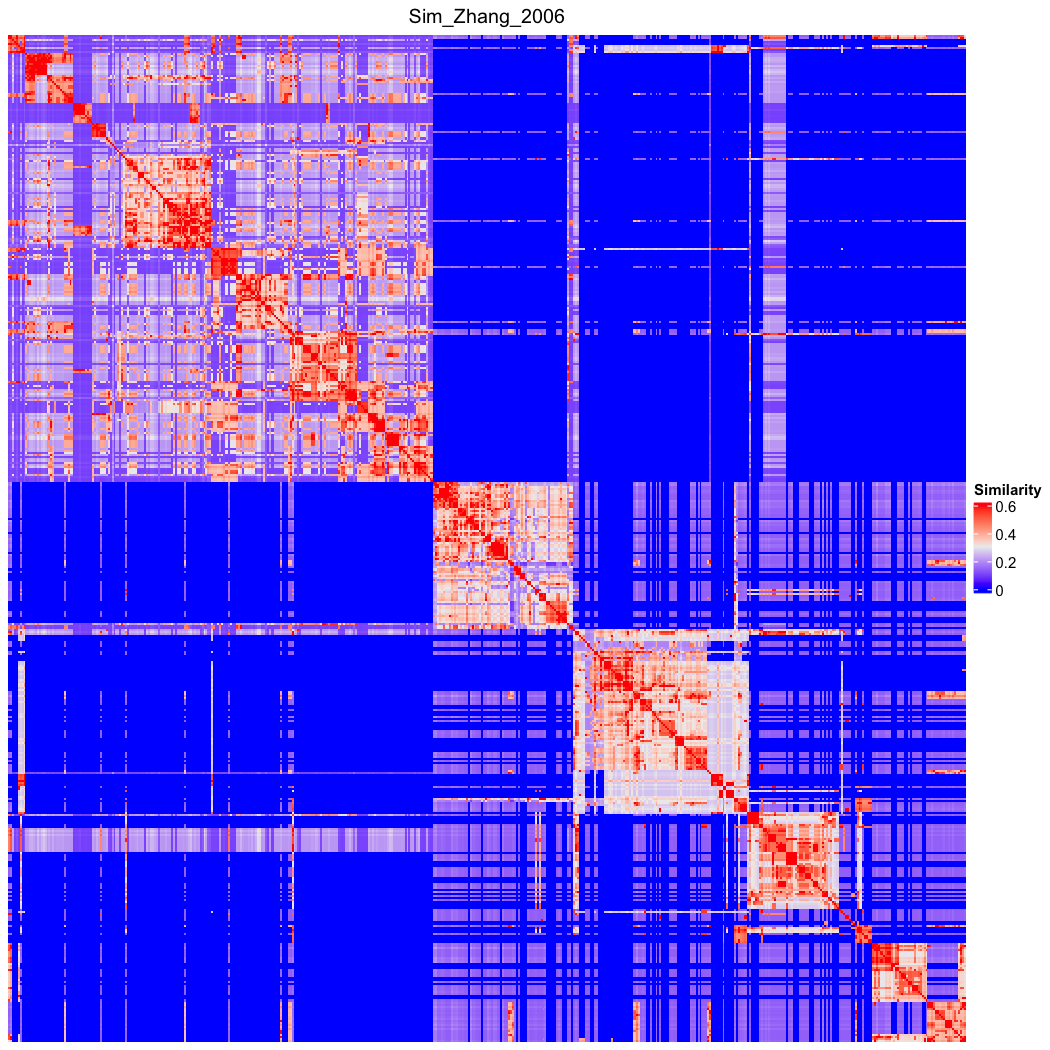

Supplement: Supplementary file 4 — Supplementary Material 4. Compare semantic similarity methods [file 12864_2024_10759_MOESM4_ESM.zip › suppl4_compare_sim_methods/image/go_bp_random_500_sim_Sim_Zhang_2006_Lin_order.png]

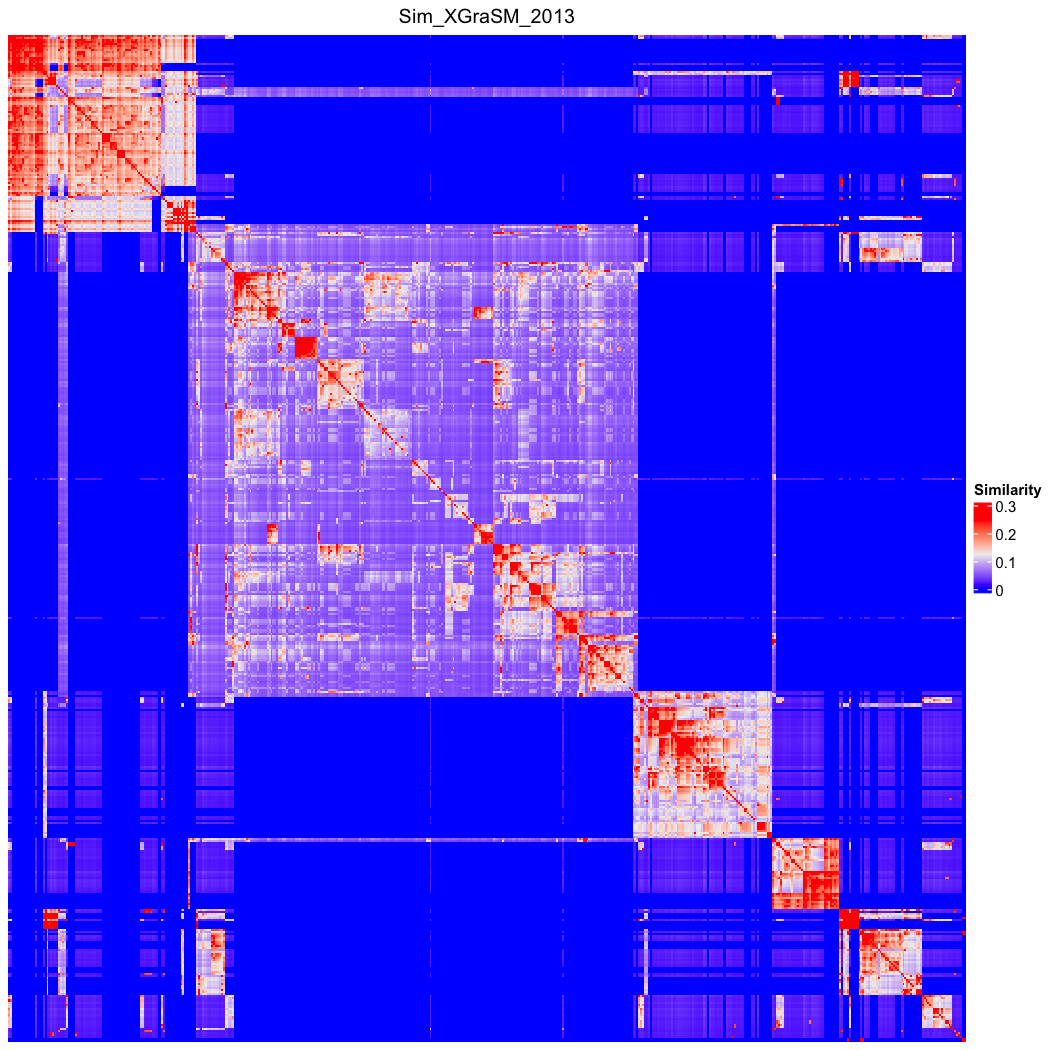

Supplement: Supplementary file 4 — Supplementary Material 4. Compare semantic similarity methods [file 12864_2024_10759_MOESM4_ESM.zip › suppl4_compare_sim_methods/image/go_bp_random_500_sim_Sim_XGraSM_2013.png]

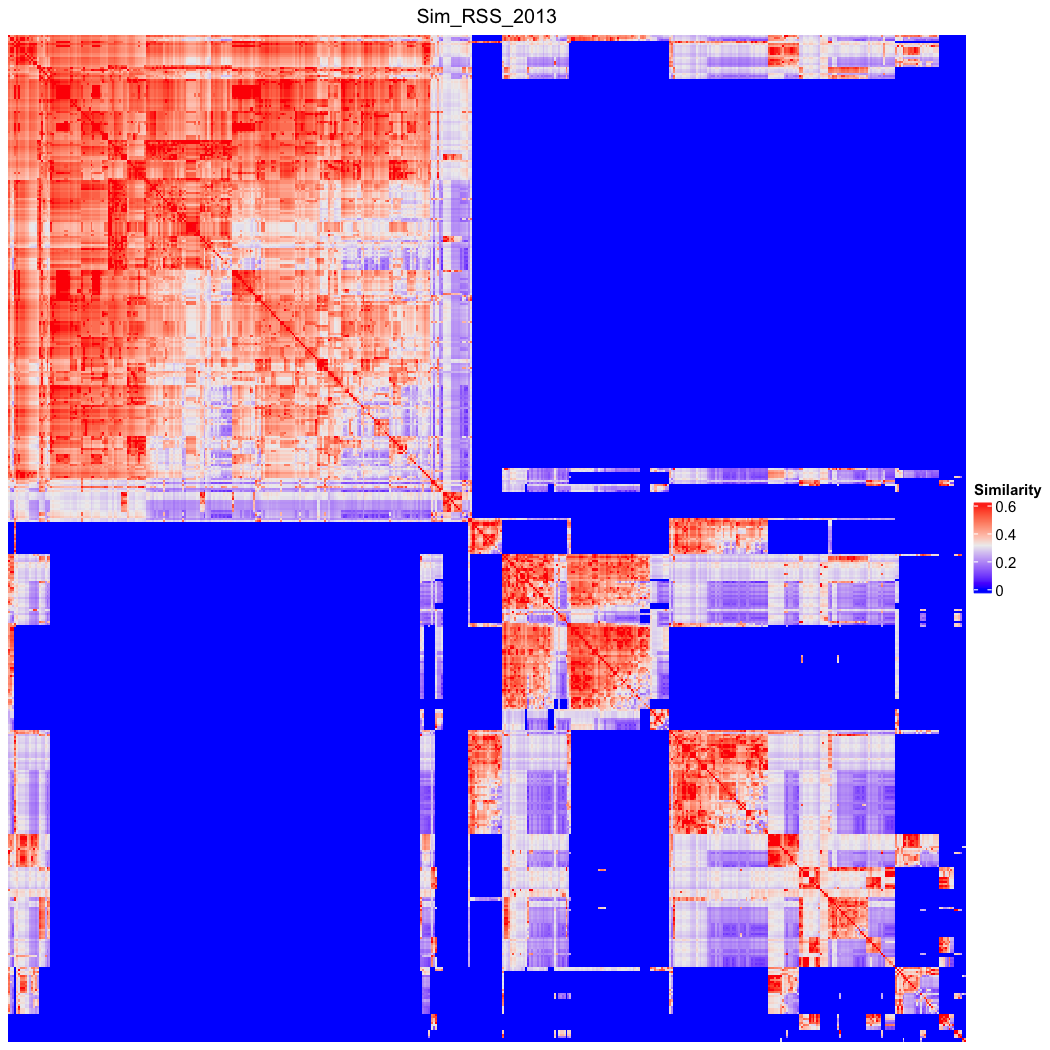

Supplement: Supplementary file 4 — Supplementary Material 4. Compare semantic similarity methods [file 12864_2024_10759_MOESM4_ESM.zip › suppl4_compare_sim_methods/image/go_bp_random_500_sim_Sim_RSS_2013.png]

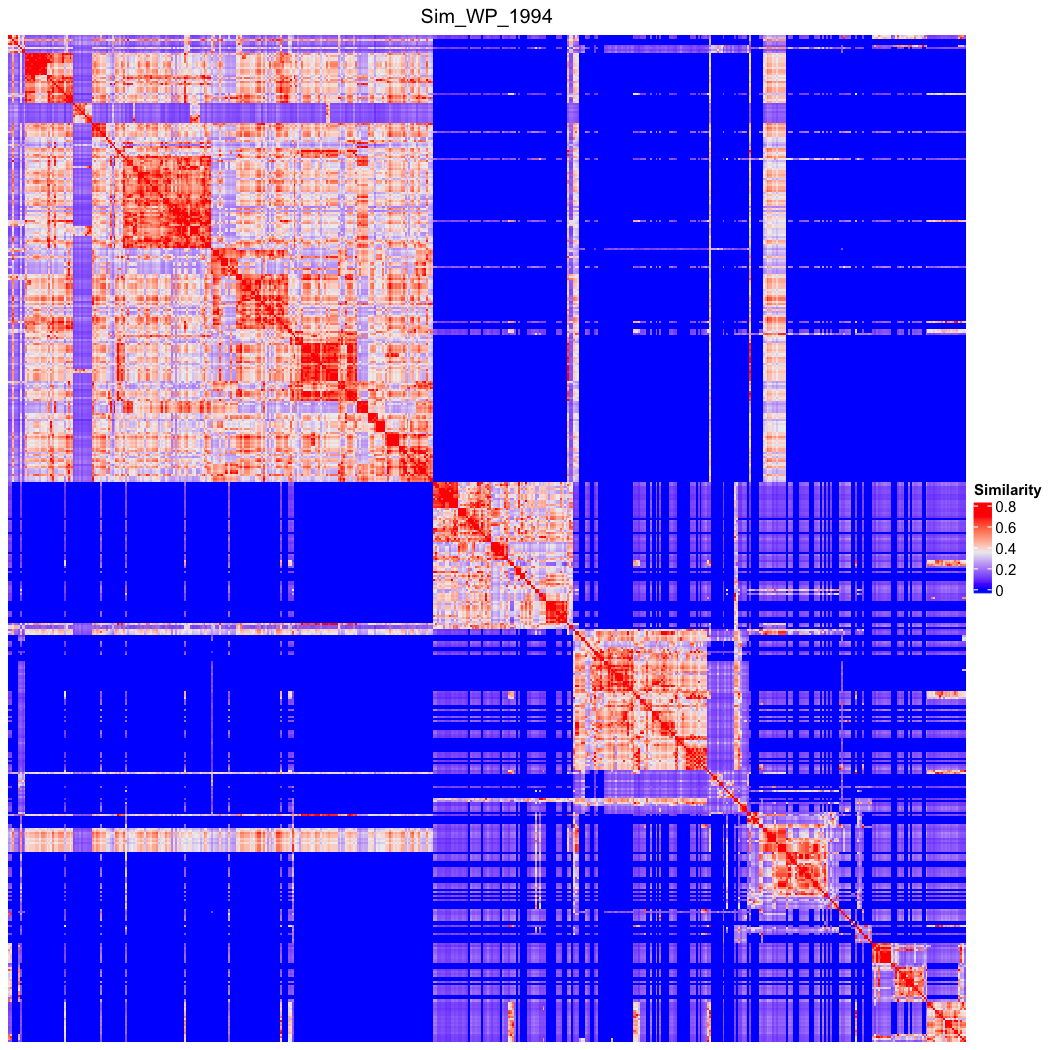

Supplement: Supplementary file 4 — Supplementary Material 4. Compare semantic similarity methods [file 12864_2024_10759_MOESM4_ESM.zip › suppl4_compare_sim_methods/image/go_bp_random_500_sim_Sim_WP_1994_Lin_order.png]

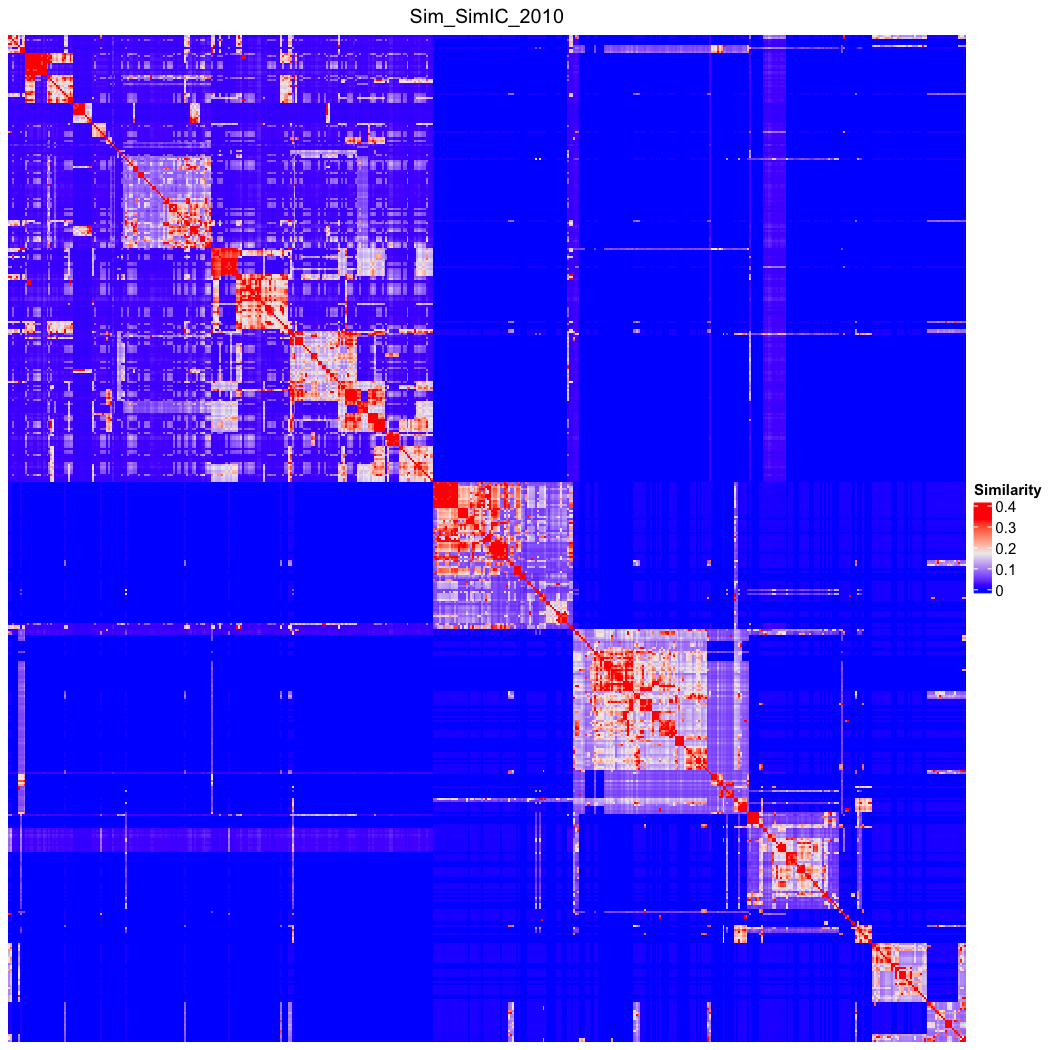

Supplement: Supplementary file 4 — Supplementary Material 4. Compare semantic similarity methods [file 12864_2024_10759_MOESM4_ESM.zip › suppl4_compare_sim_methods/image/go_bp_random_500_sim_Sim_SimIC_2010_Lin_order.png]

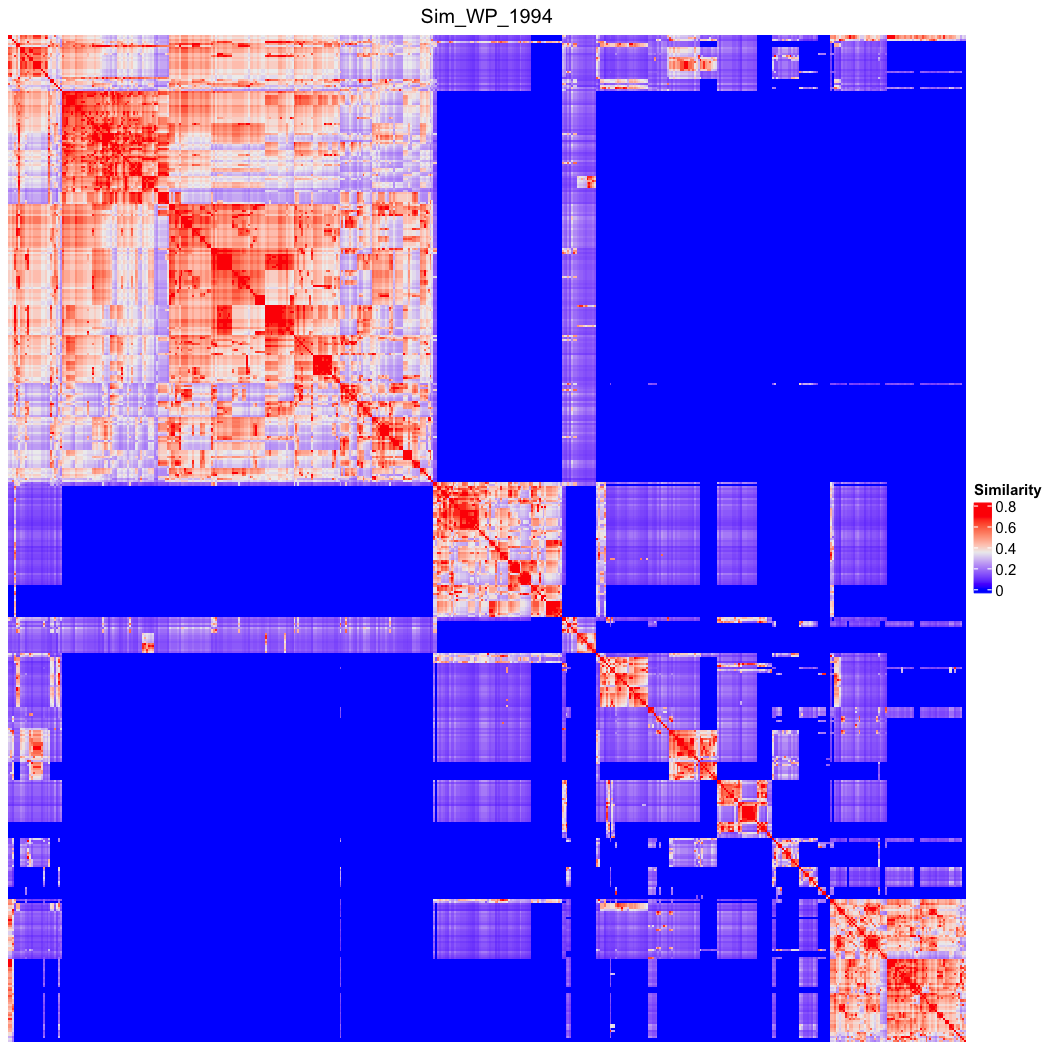

Supplement: Supplementary file 4 — Supplementary Material 4. Compare semantic similarity methods [file 12864_2024_10759_MOESM4_ESM.zip › suppl4_compare_sim_methods/image/go_bp_random_500_sim_Sim_WP_1994.png]

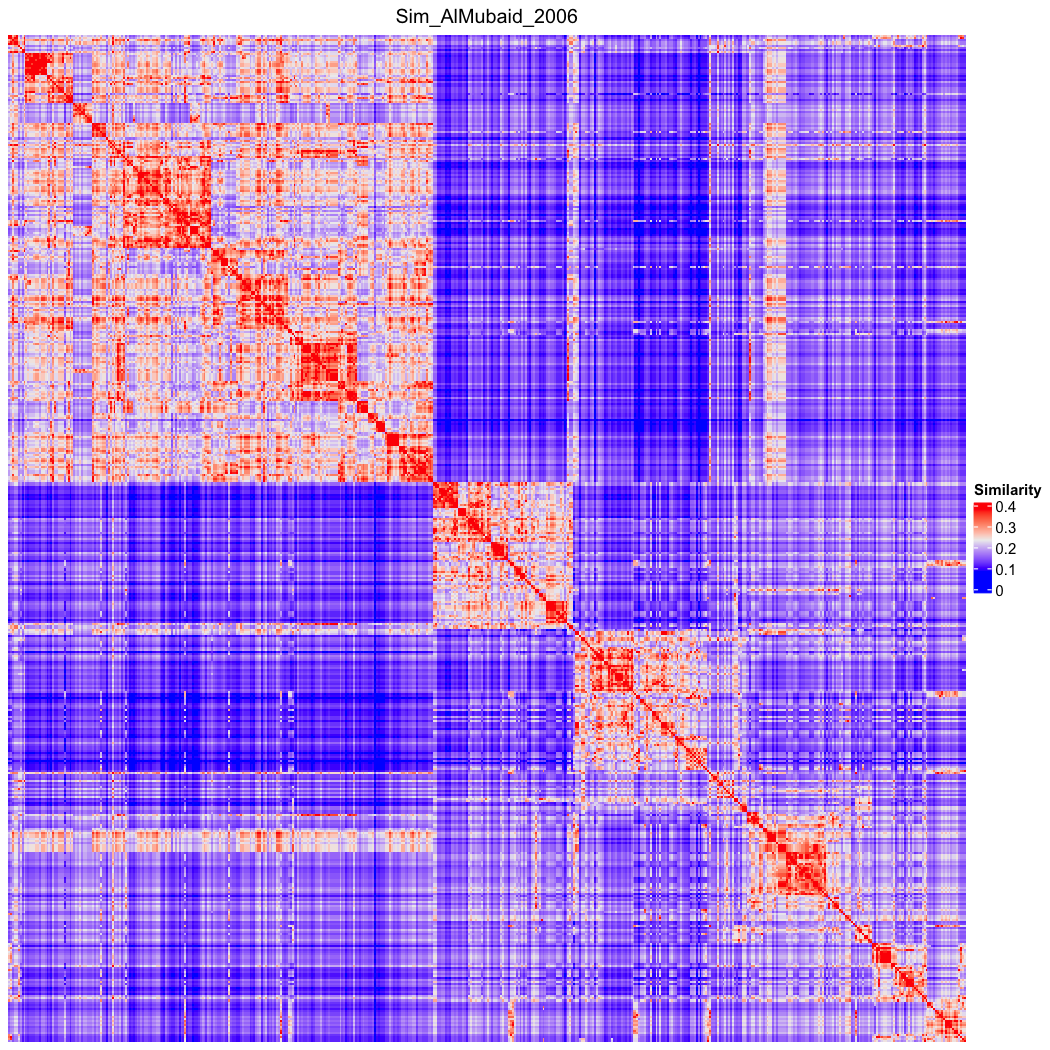

Supplement: Supplementary file 4 — Supplementary Material 4. Compare semantic similarity methods [file 12864_2024_10759_MOESM4_ESM.zip › suppl4_compare_sim_methods/image/go_bp_random_500_sim_Sim_AlMubaid_2006_Lin_order.png]

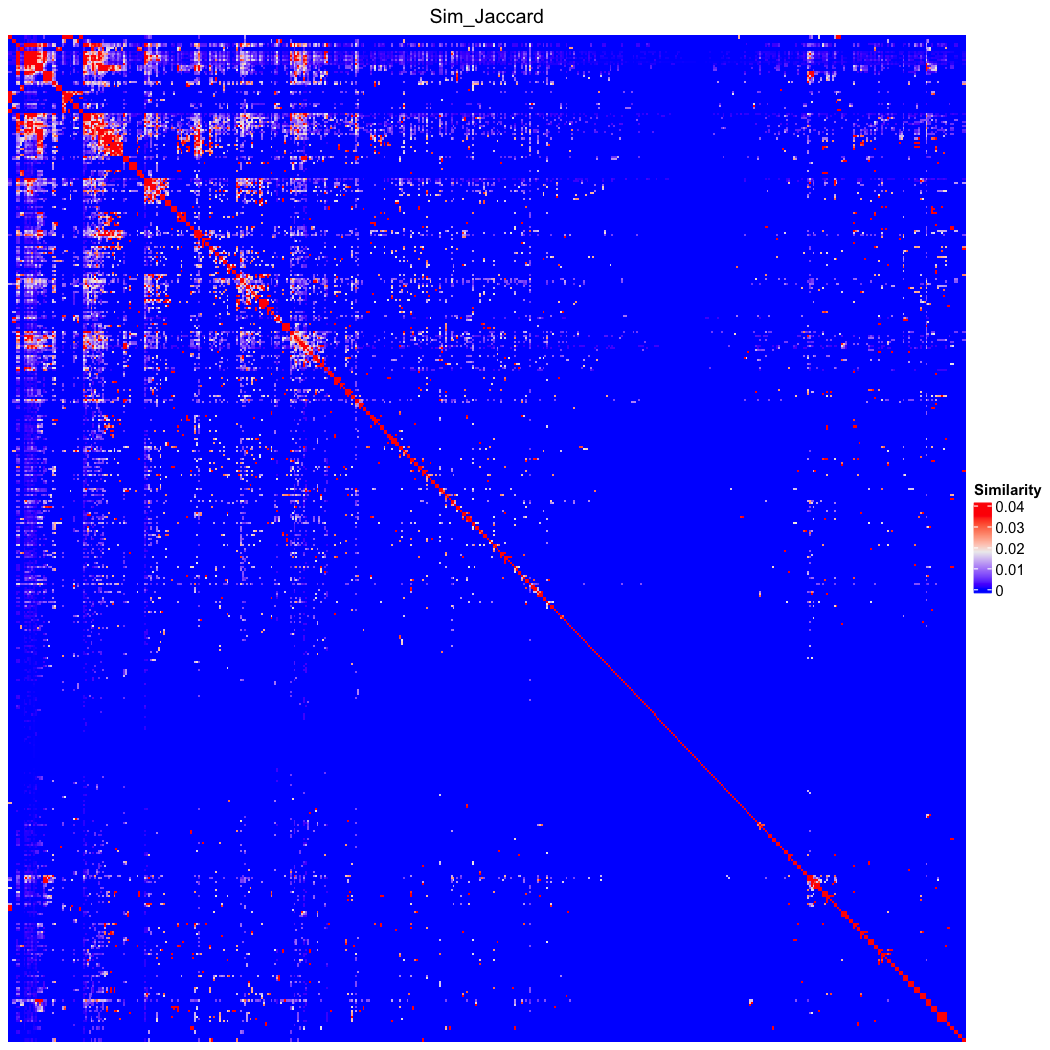

Supplement: Supplementary file 4 — Supplementary Material 4. Compare semantic similarity methods [file 12864_2024_10759_MOESM4_ESM.zip › suppl4_compare_sim_methods/image/go_bp_random_500_sim_Sim_Jaccard.png]

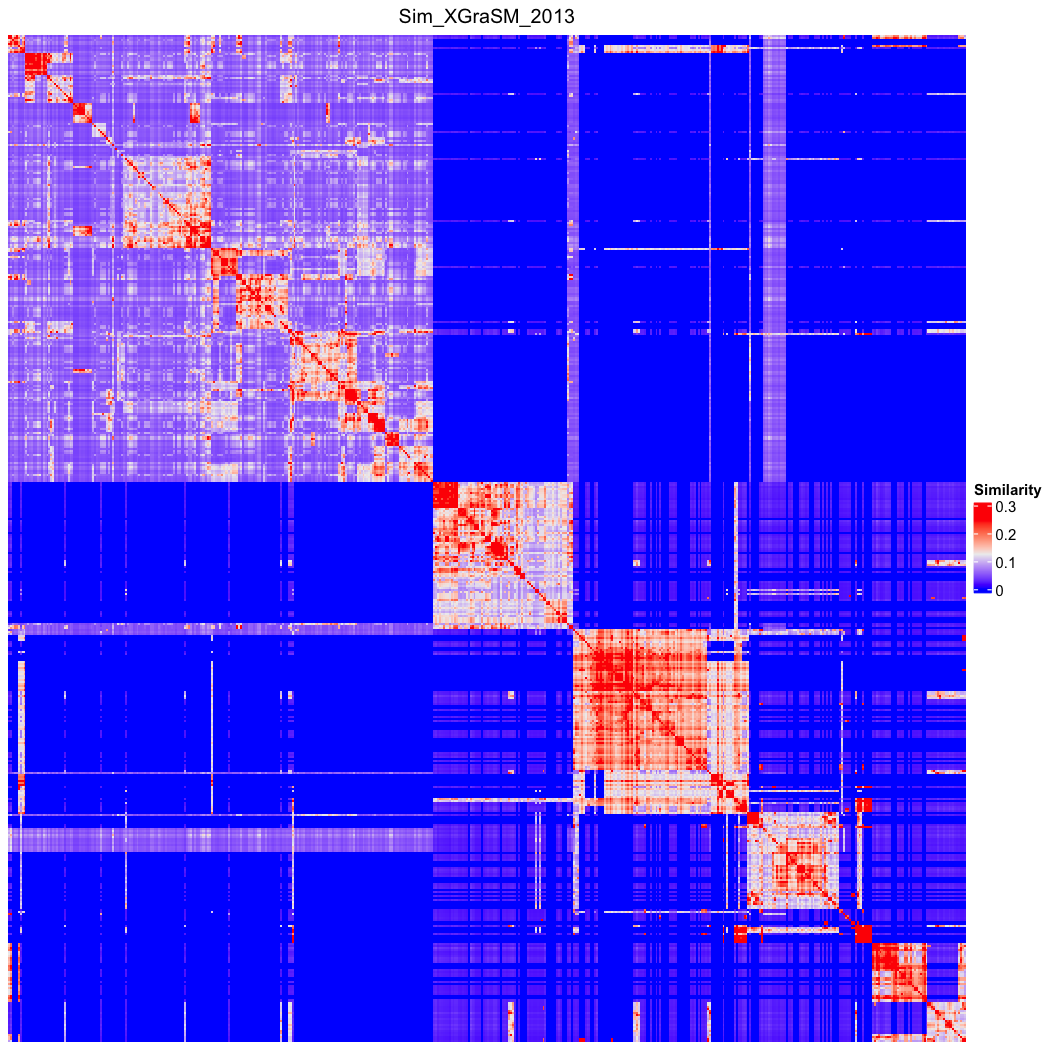

Supplement: Supplementary file 4 — Supplementary Material 4. Compare semantic similarity methods [file 12864_2024_10759_MOESM4_ESM.zip › suppl4_compare_sim_methods/image/go_bp_random_500_sim_Sim_XGraSM_2013_Lin_order.png]

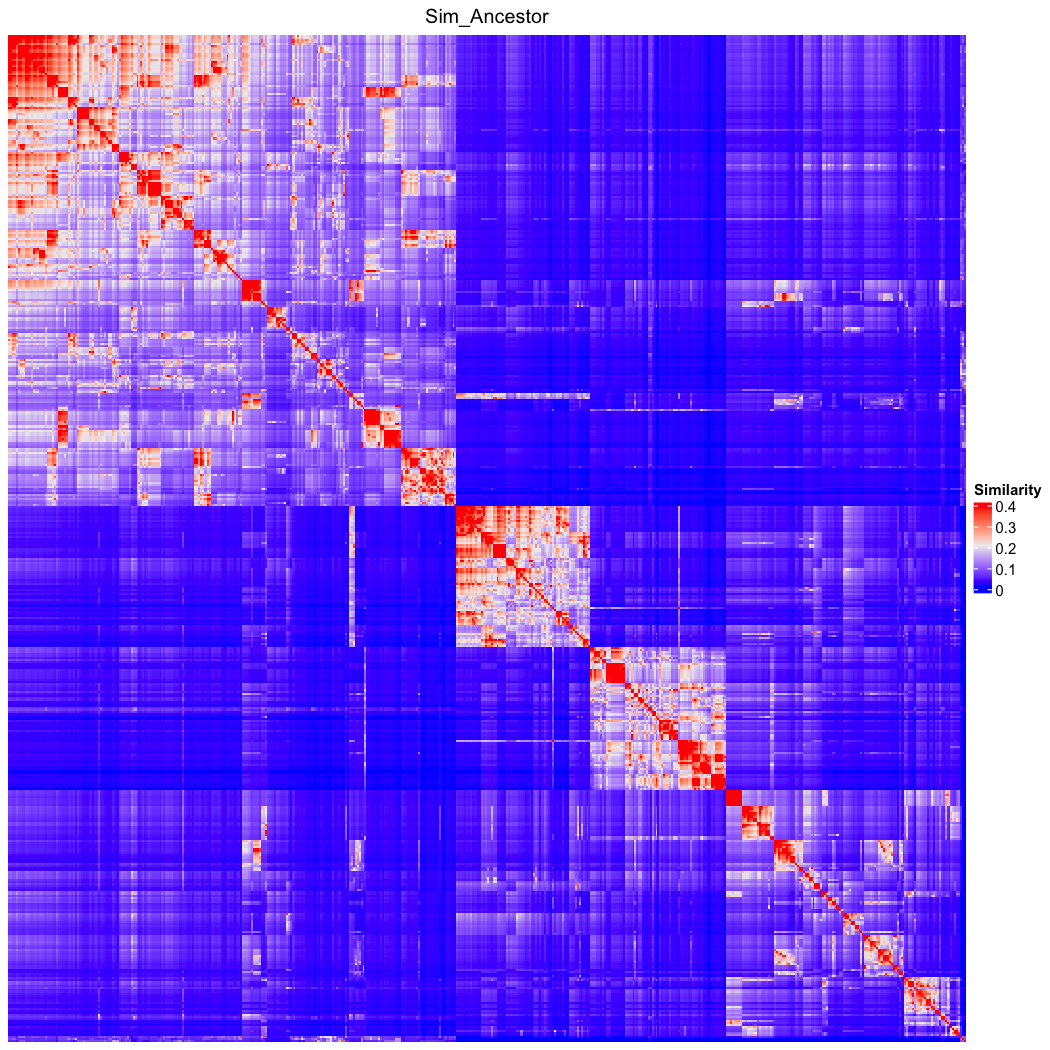

Supplement: Supplementary file 4 — Supplementary Material 4. Compare semantic similarity methods [file 12864_2024_10759_MOESM4_ESM.zip › suppl4_compare_sim_methods/image/go_bp_random_500_sim_Sim_Ancestor.png]

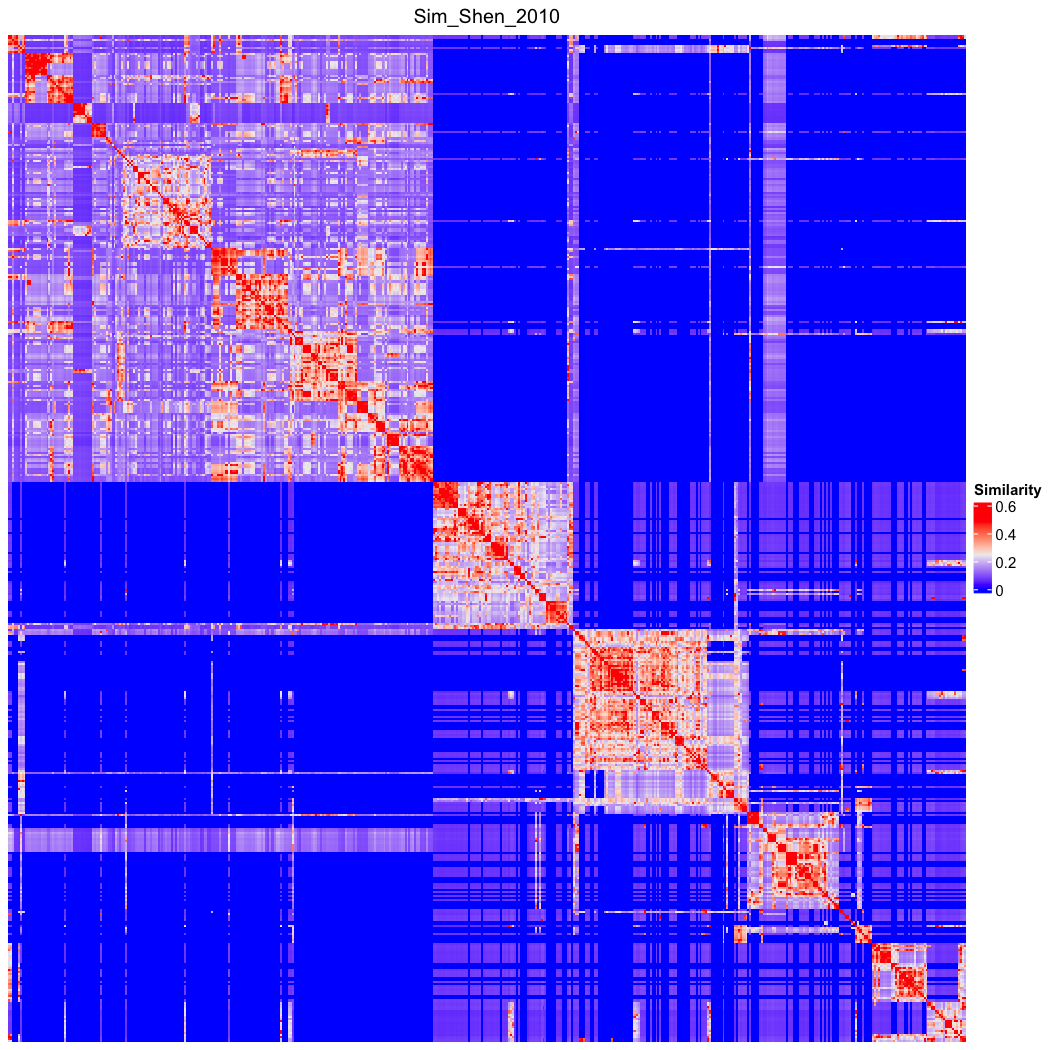

Supplement: Supplementary file 4 — Supplementary Material 4. Compare semantic similarity methods [file 12864_2024_10759_MOESM4_ESM.zip › suppl4_compare_sim_methods/image/go_bp_random_500_sim_Sim_Shen_2010_Lin_order.png]

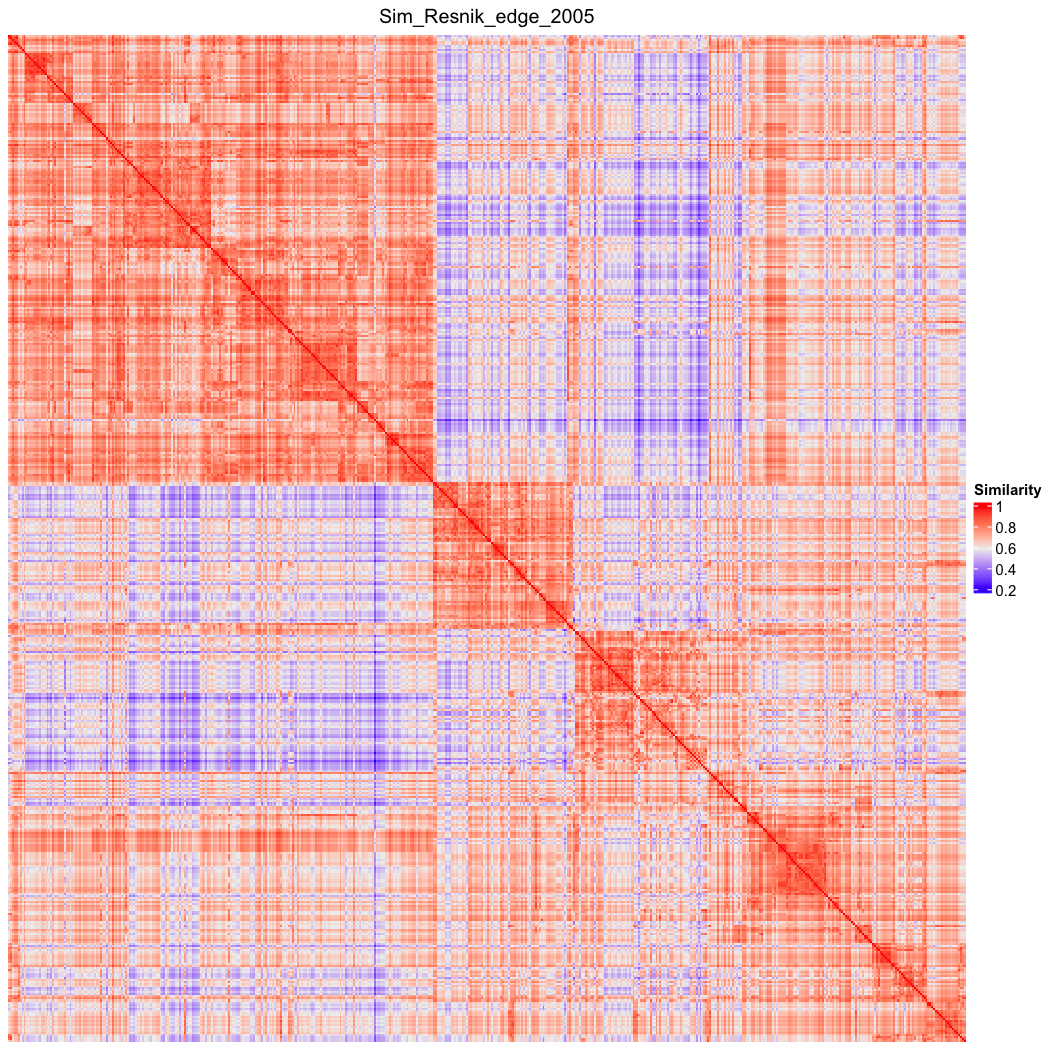

Supplement: Supplementary file 4 — Supplementary Material 4. Compare semantic similarity methods [file 12864_2024_10759_MOESM4_ESM.zip › suppl4_compare_sim_methods/image/go_bp_random_500_sim_Sim_Resnik_edge_2005_Lin_order.png]

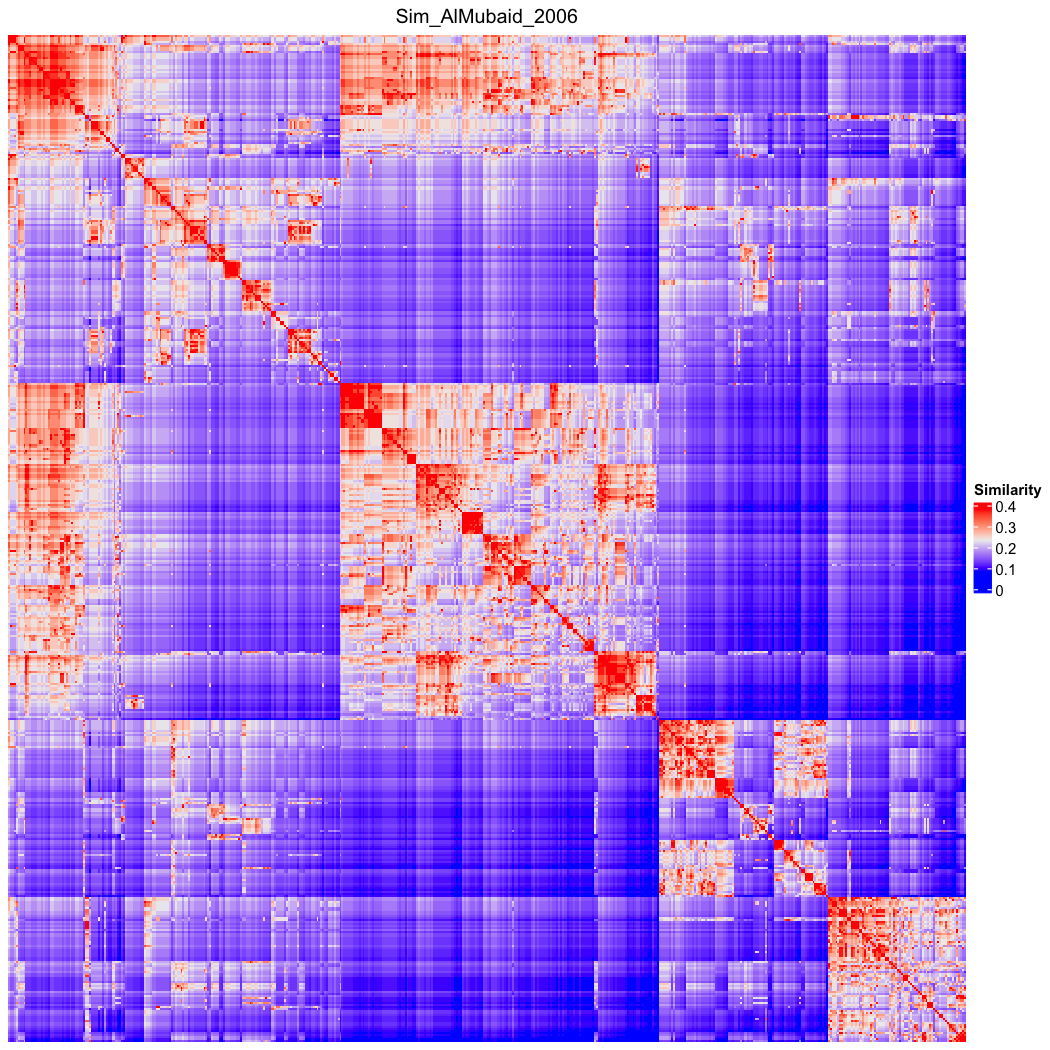

Supplement: Supplementary file 4 — Supplementary Material 4. Compare semantic similarity methods [file 12864_2024_10759_MOESM4_ESM.zip › suppl4_compare_sim_methods/image/go_bp_random_500_sim_Sim_AlMubaid_2006.png]

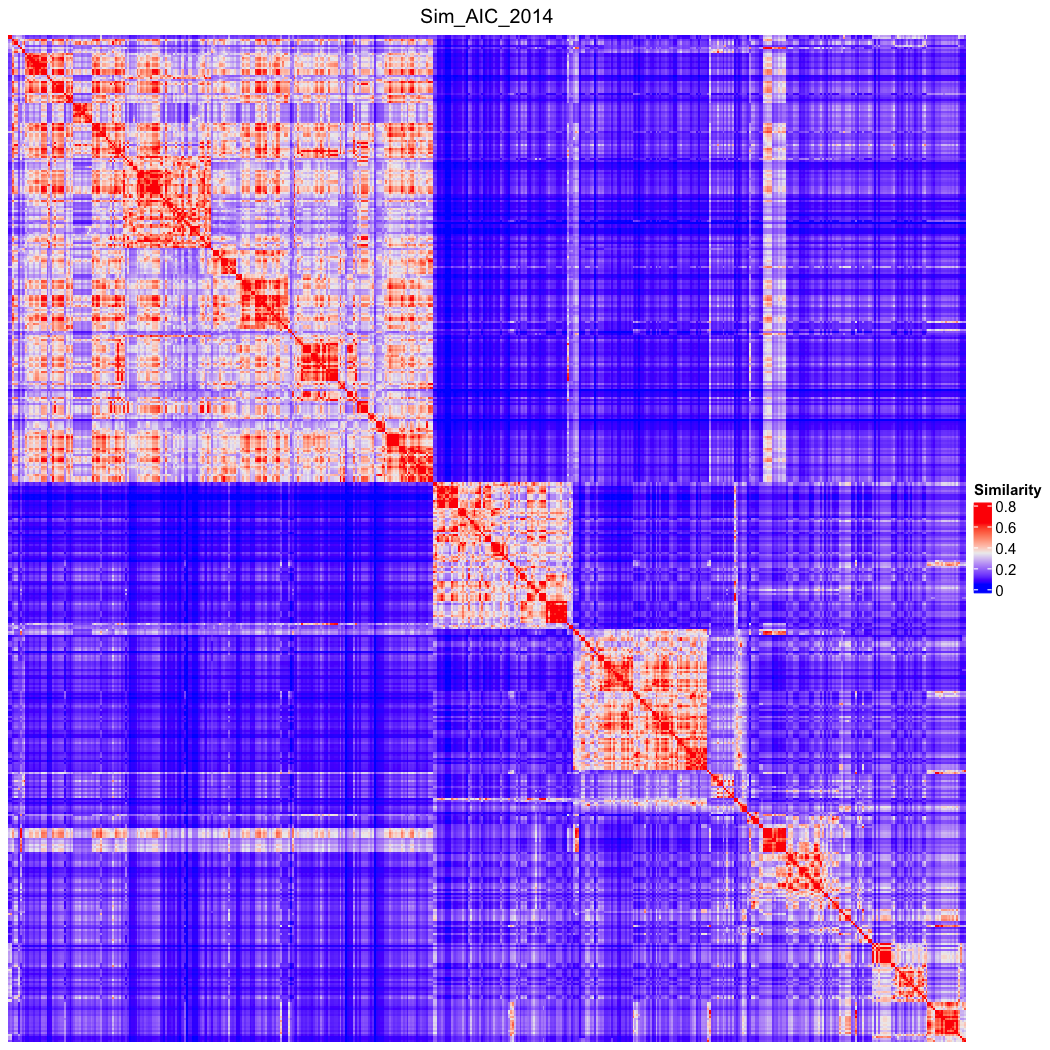

Supplement: Supplementary file 4 — Supplementary Material 4. Compare semantic similarity methods [file 12864_2024_10759_MOESM4_ESM.zip › suppl4_compare_sim_methods/image/go_bp_random_500_sim_Sim_AIC_2014_Lin_order.png]

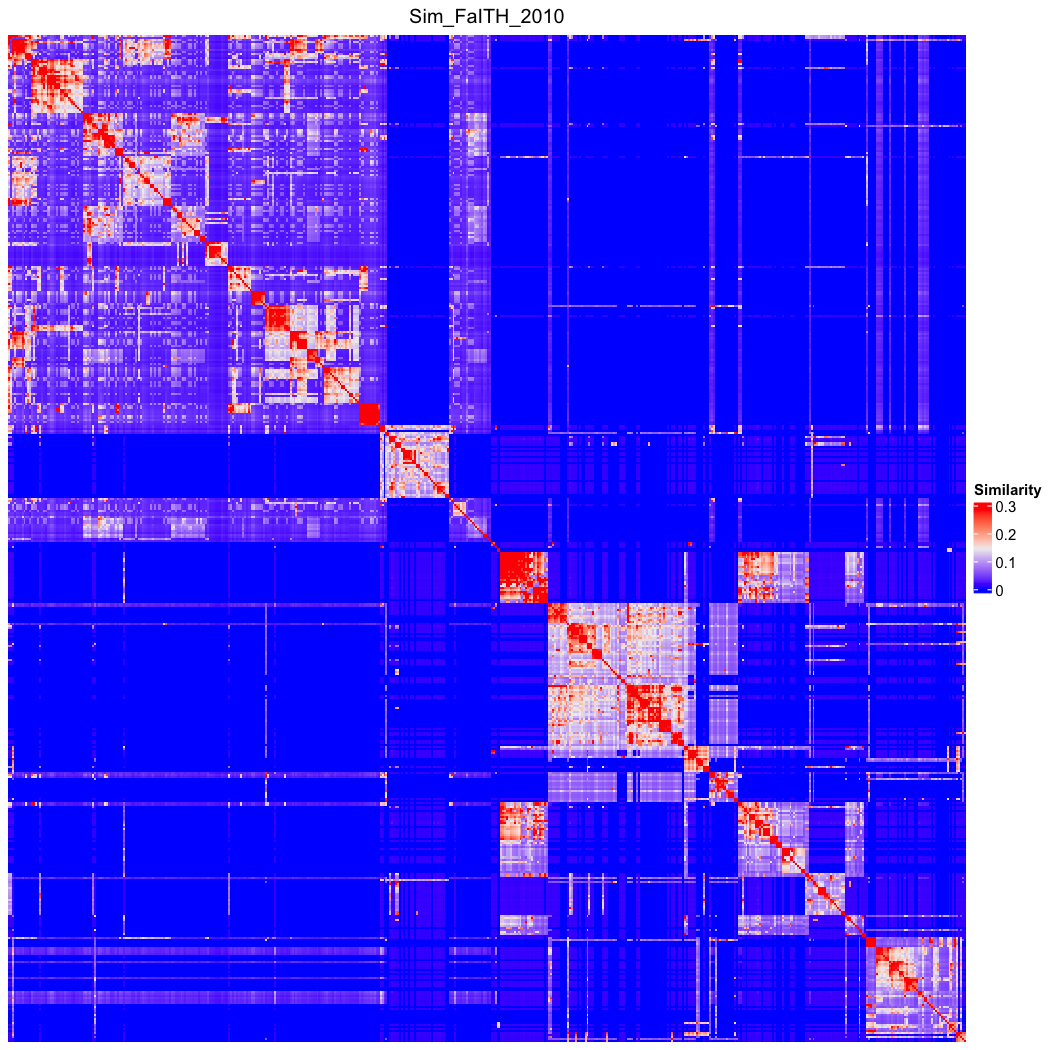

Supplement: Supplementary file 4 — Supplementary Material 4. Compare semantic similarity methods [file 12864_2024_10759_MOESM4_ESM.zip › suppl4_compare_sim_methods/image/go_bp_random_500_sim_Sim_FaITH_2010.png]

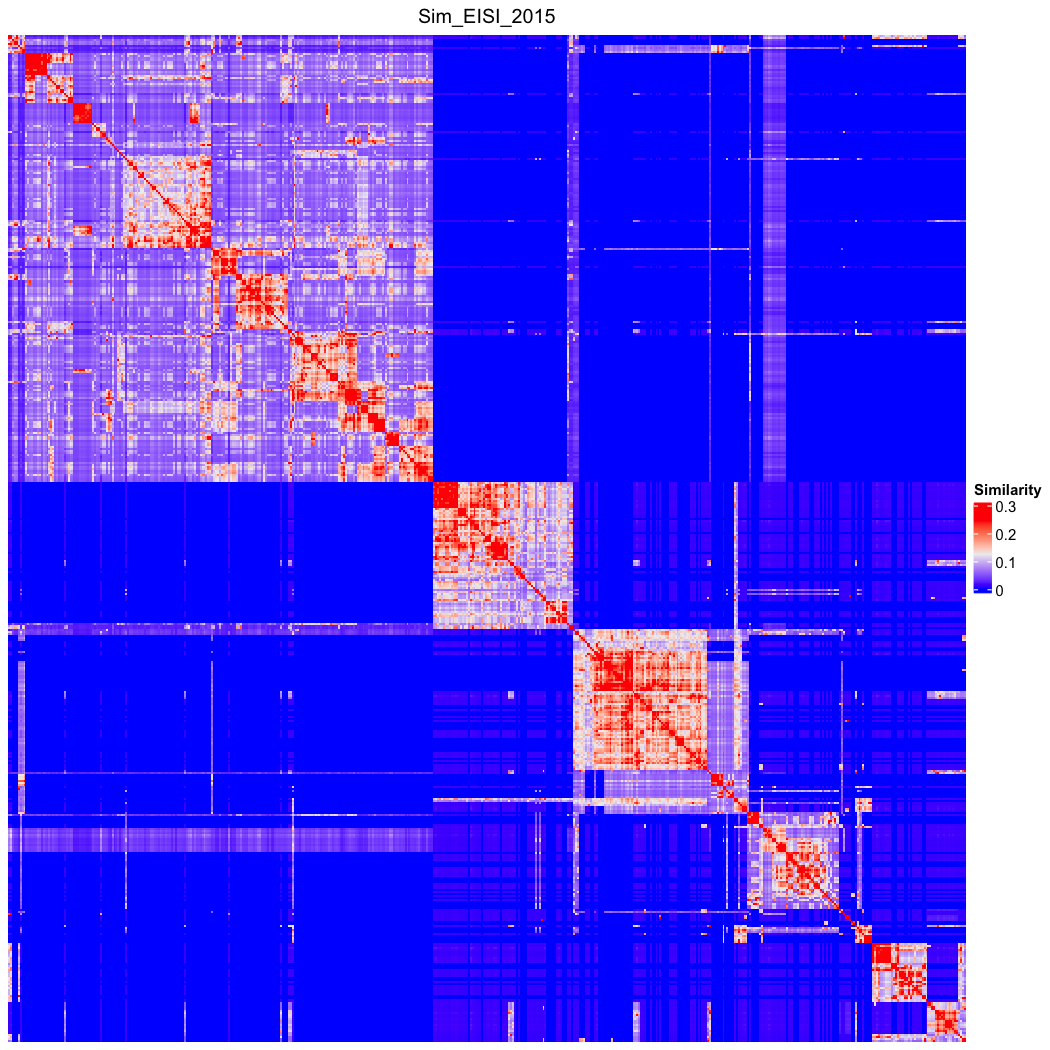

Supplement: Supplementary file 4 — Supplementary Material 4. Compare semantic similarity methods [file 12864_2024_10759_MOESM4_ESM.zip › suppl4_compare_sim_methods/image/go_bp_random_500_sim_Sim_EISI_2015_Lin_order.png]

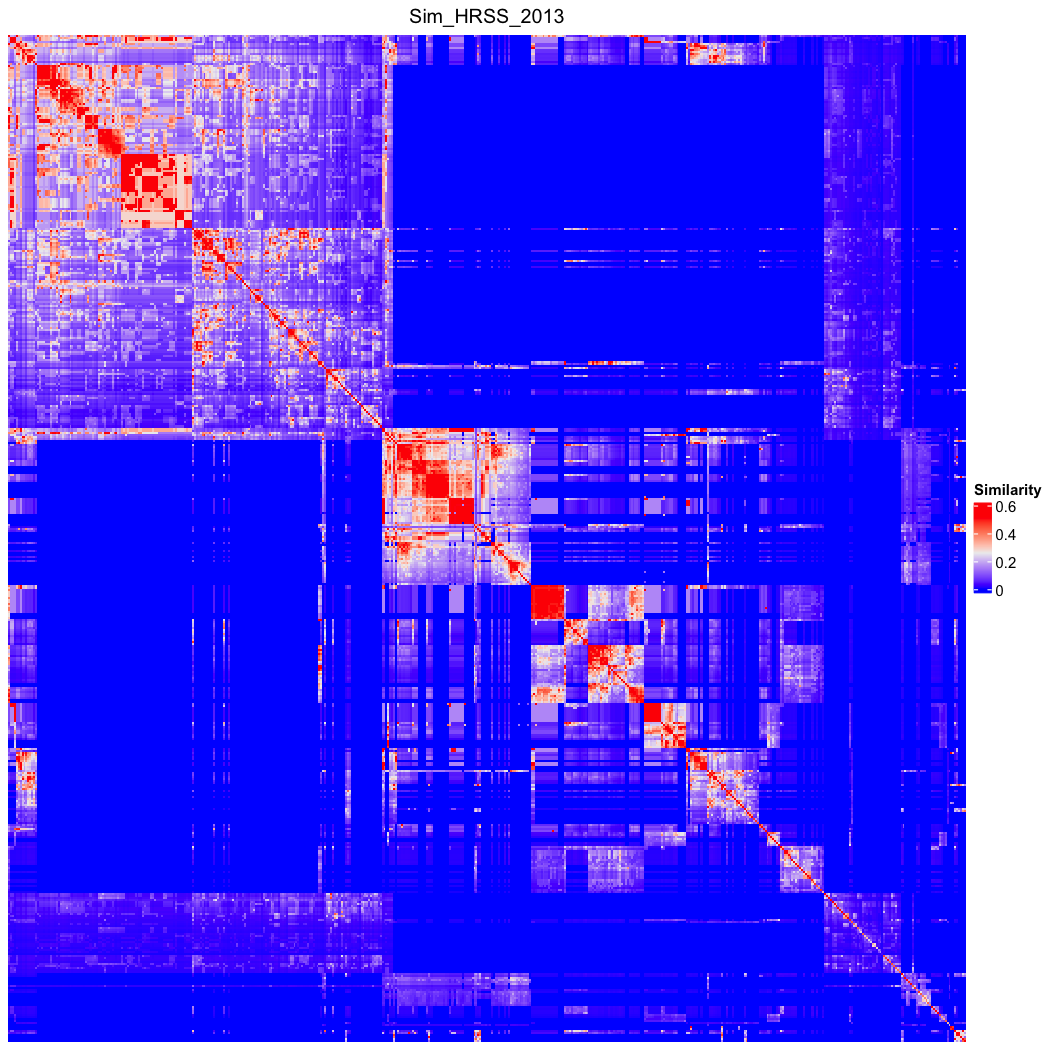

Supplement: Supplementary file 4 — Supplementary Material 4. Compare semantic similarity methods [file 12864_2024_10759_MOESM4_ESM.zip › suppl4_compare_sim_methods/image/go_bp_random_500_sim_Sim_HRSS_2013.png]

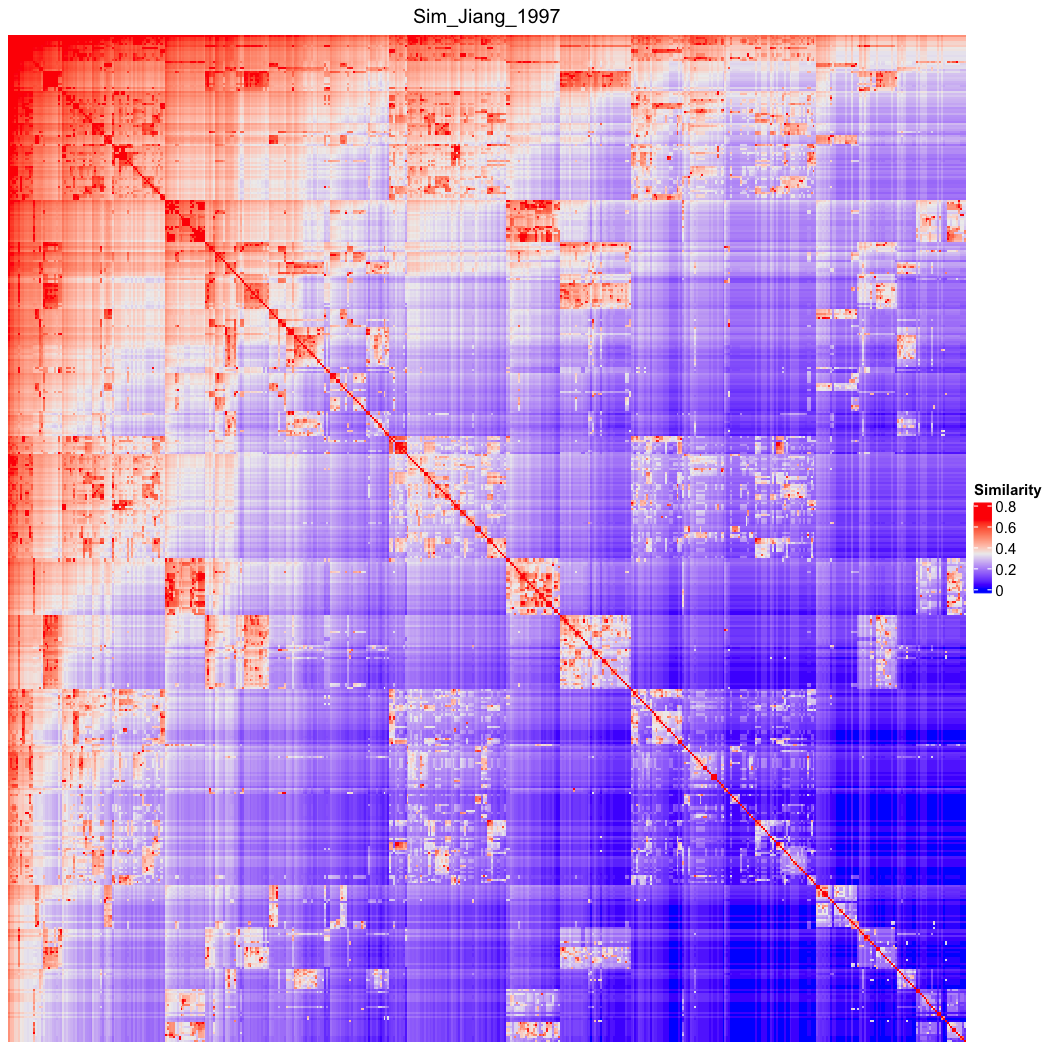

Supplement: Supplementary file 4 — Supplementary Material 4. Compare semantic similarity methods [file 12864_2024_10759_MOESM4_ESM.zip › suppl4_compare_sim_methods/image/go_bp_random_500_sim_Sim_Jiang_1997.png]

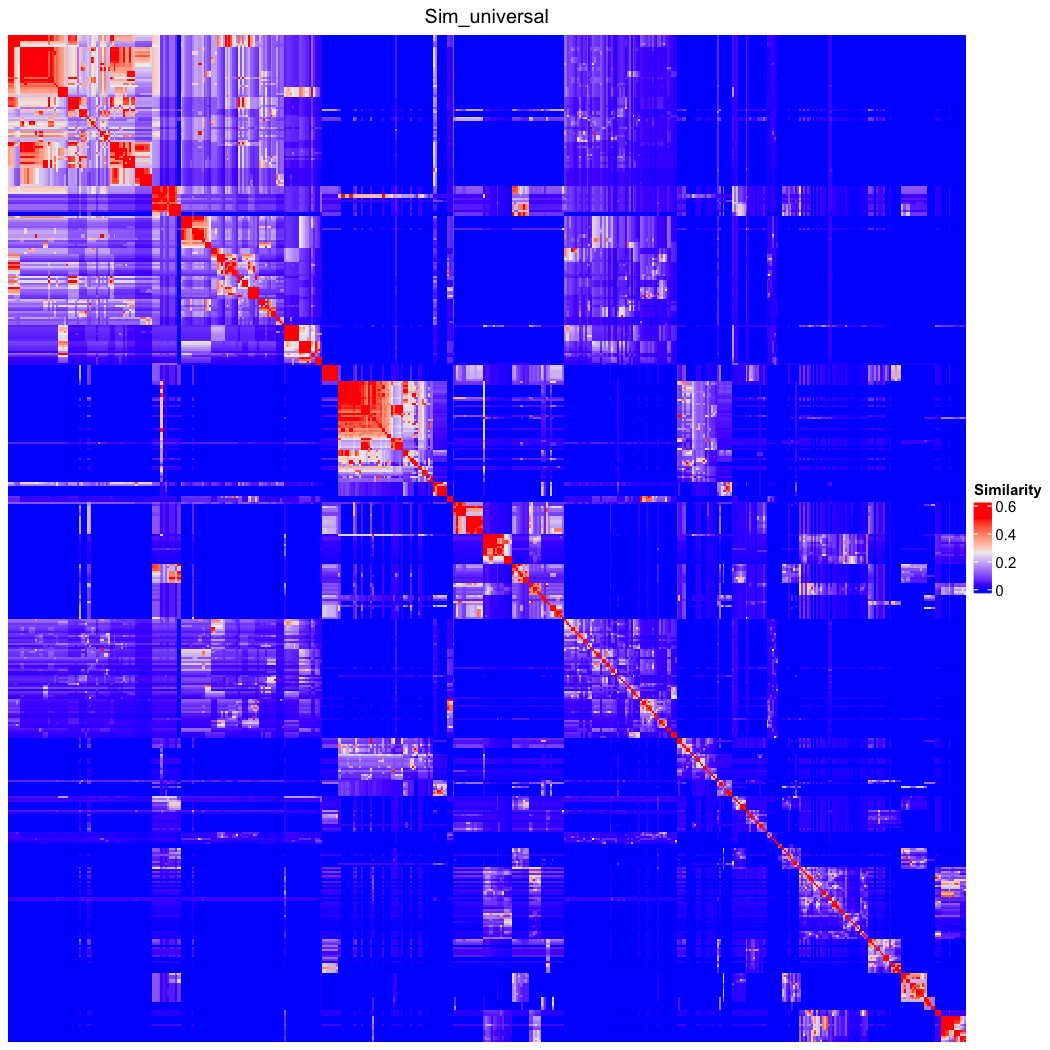

Supplement: Supplementary file 4 — Supplementary Material 4. Compare semantic similarity methods [file 12864_2024_10759_MOESM4_ESM.zip › suppl4_compare_sim_methods/image/go_bp_random_500_sim_Sim_universal.png]

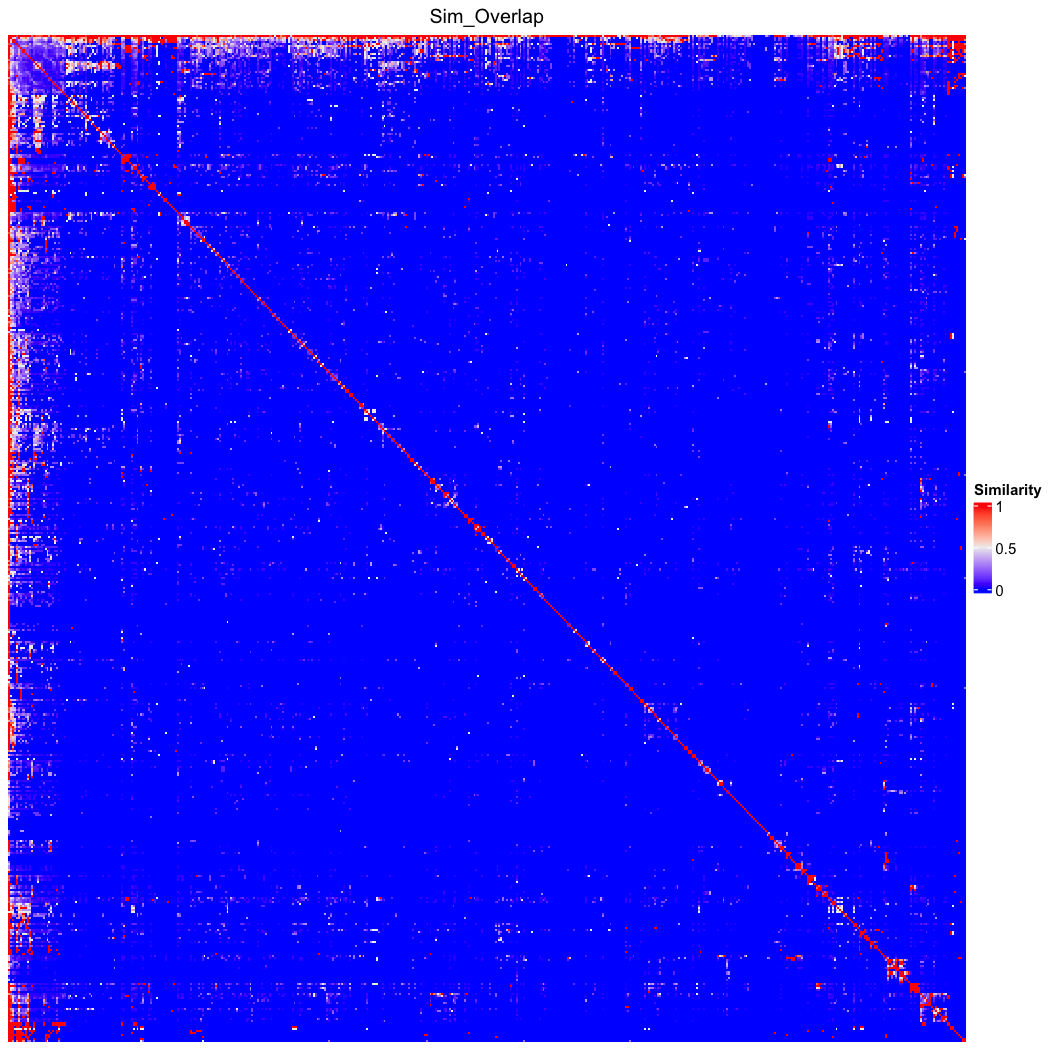

Supplement: Supplementary file 4 — Supplementary Material 4. Compare semantic similarity methods [file 12864_2024_10759_MOESM4_ESM.zip › suppl4_compare_sim_methods/image/go_bp_random_500_sim_Sim_Overlap.png]

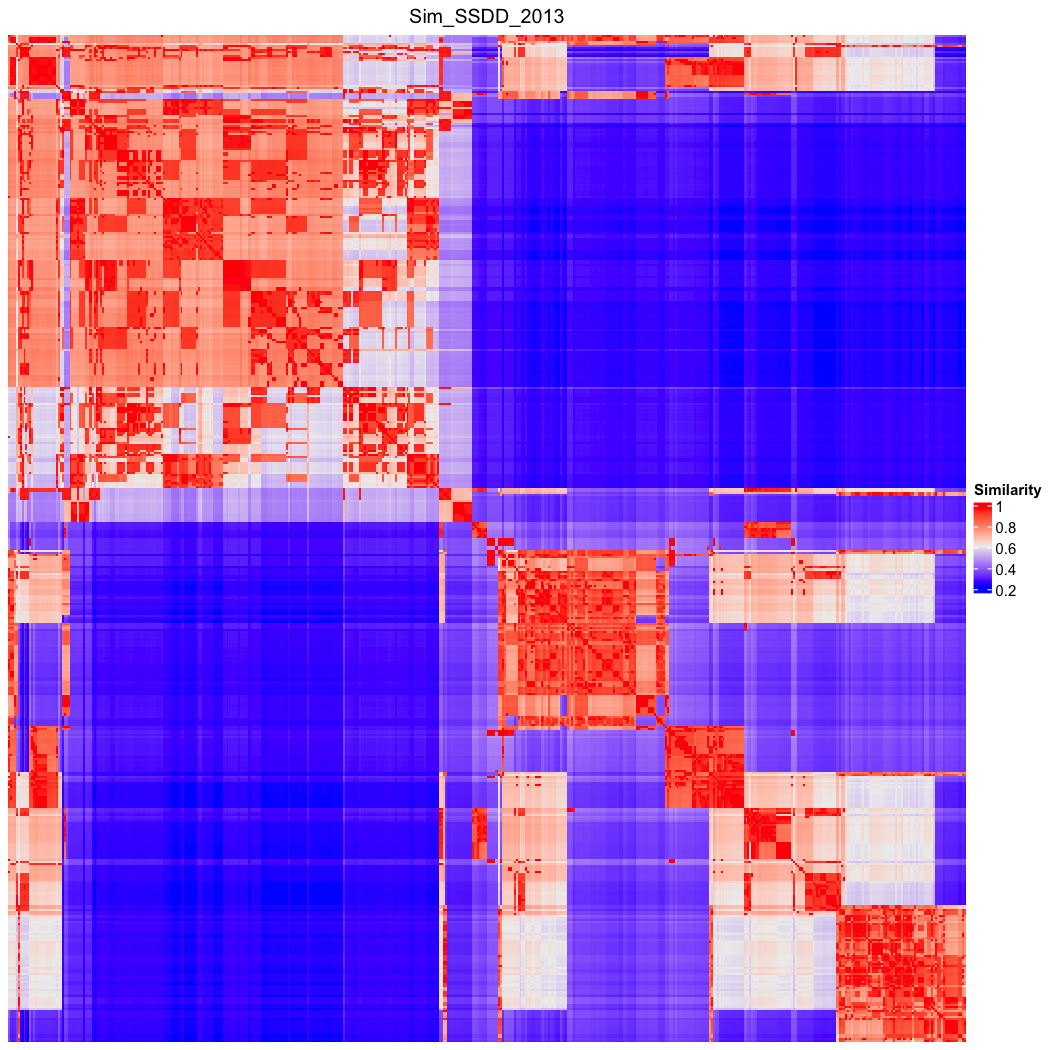

Supplement: Supplementary file 4 — Supplementary Material 4. Compare semantic similarity methods [file 12864_2024_10759_MOESM4_ESM.zip › suppl4_compare_sim_methods/image/go_bp_random_500_sim_Sim_SSDD_2013.png]

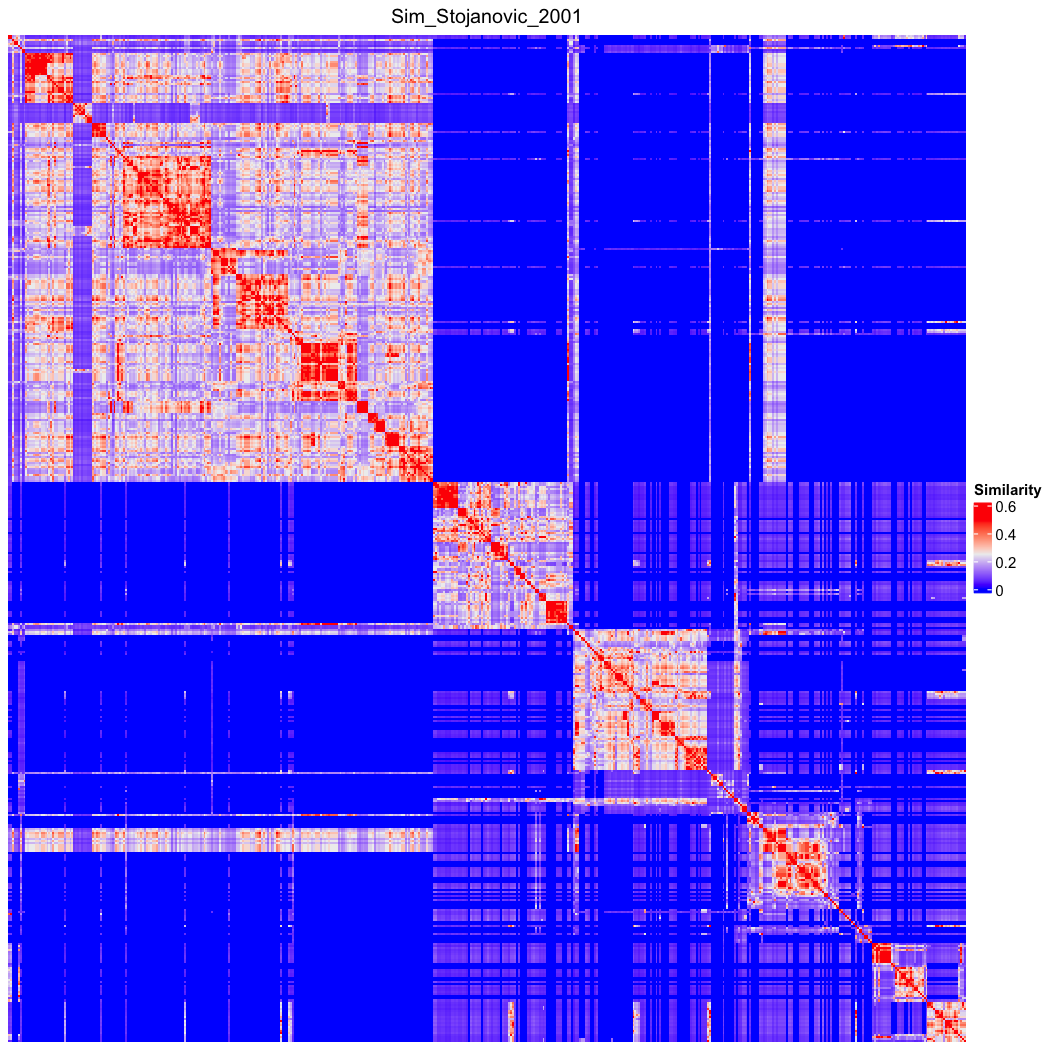

Supplement: Supplementary file 4 — Supplementary Material 4. Compare semantic similarity methods [file 12864_2024_10759_MOESM4_ESM.zip › suppl4_compare_sim_methods/image/go_bp_random_500_sim_Sim_Stojanovic_2001_Lin_order.png]

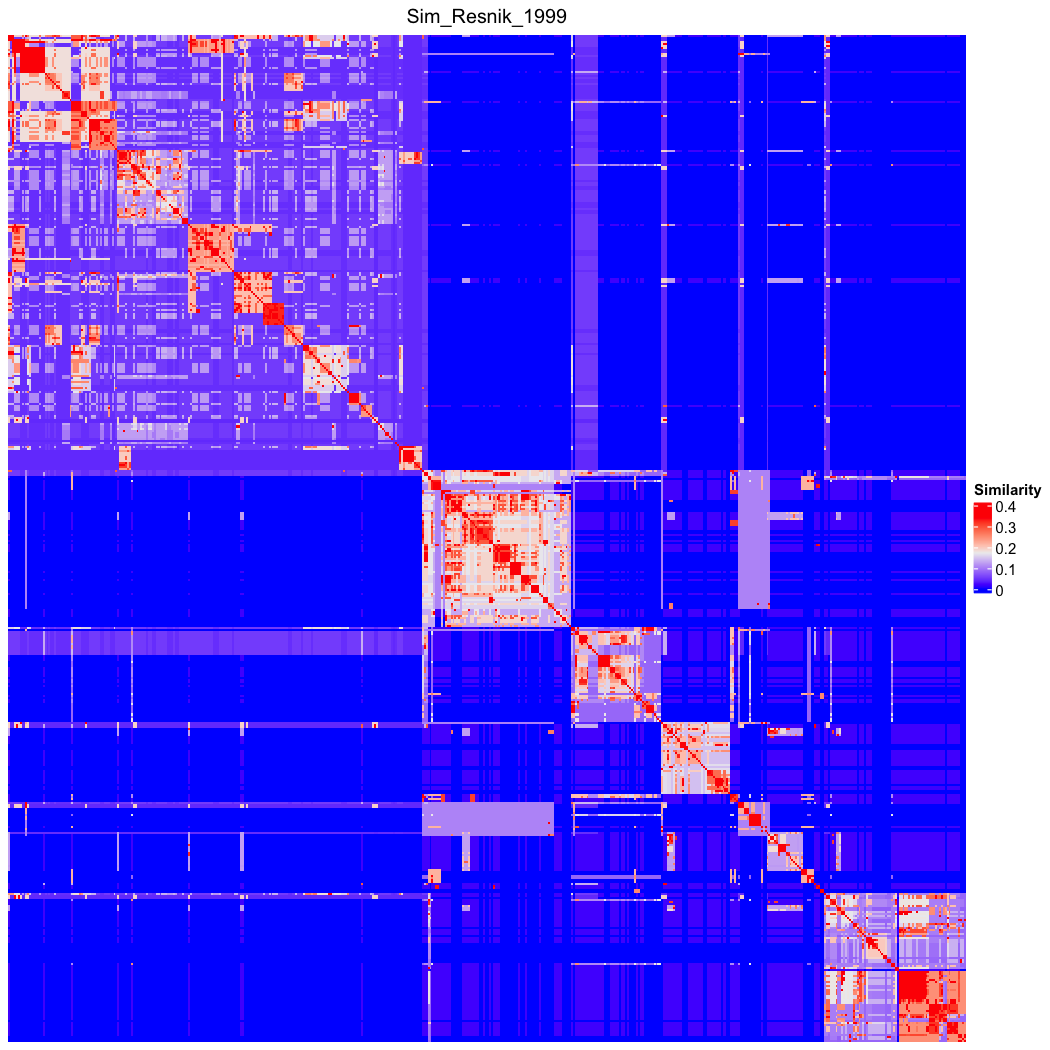

Supplement: Supplementary file 4 — Supplementary Material 4. Compare semantic similarity methods [file 12864_2024_10759_MOESM4_ESM.zip › suppl4_compare_sim_methods/image/go_bp_random_500_sim_Sim_Resnik_1999.png]

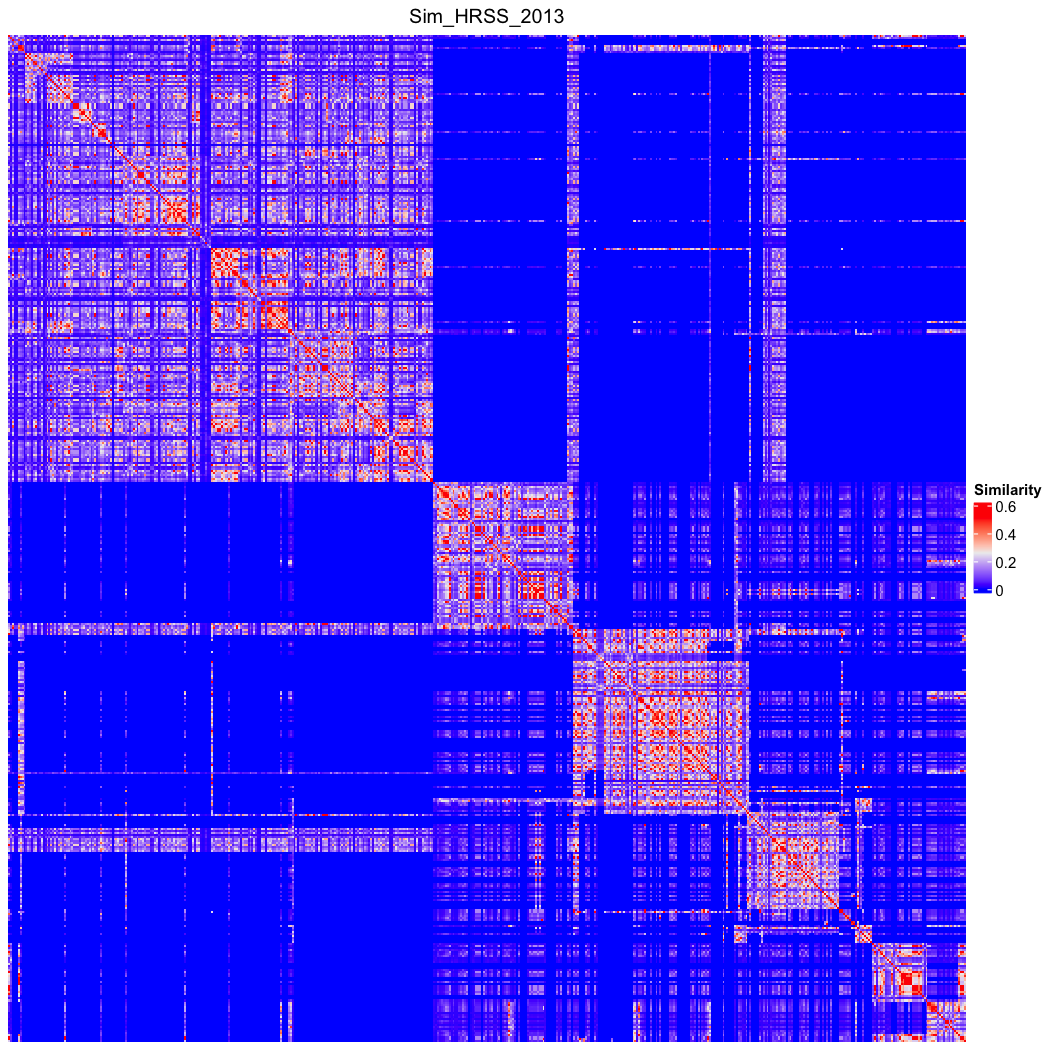

Supplement: Supplementary file 4 — Supplementary Material 4. Compare semantic similarity methods [file 12864_2024_10759_MOESM4_ESM.zip › suppl4_compare_sim_methods/image/go_bp_random_500_sim_Sim_HRSS_2013_Lin_order.png]

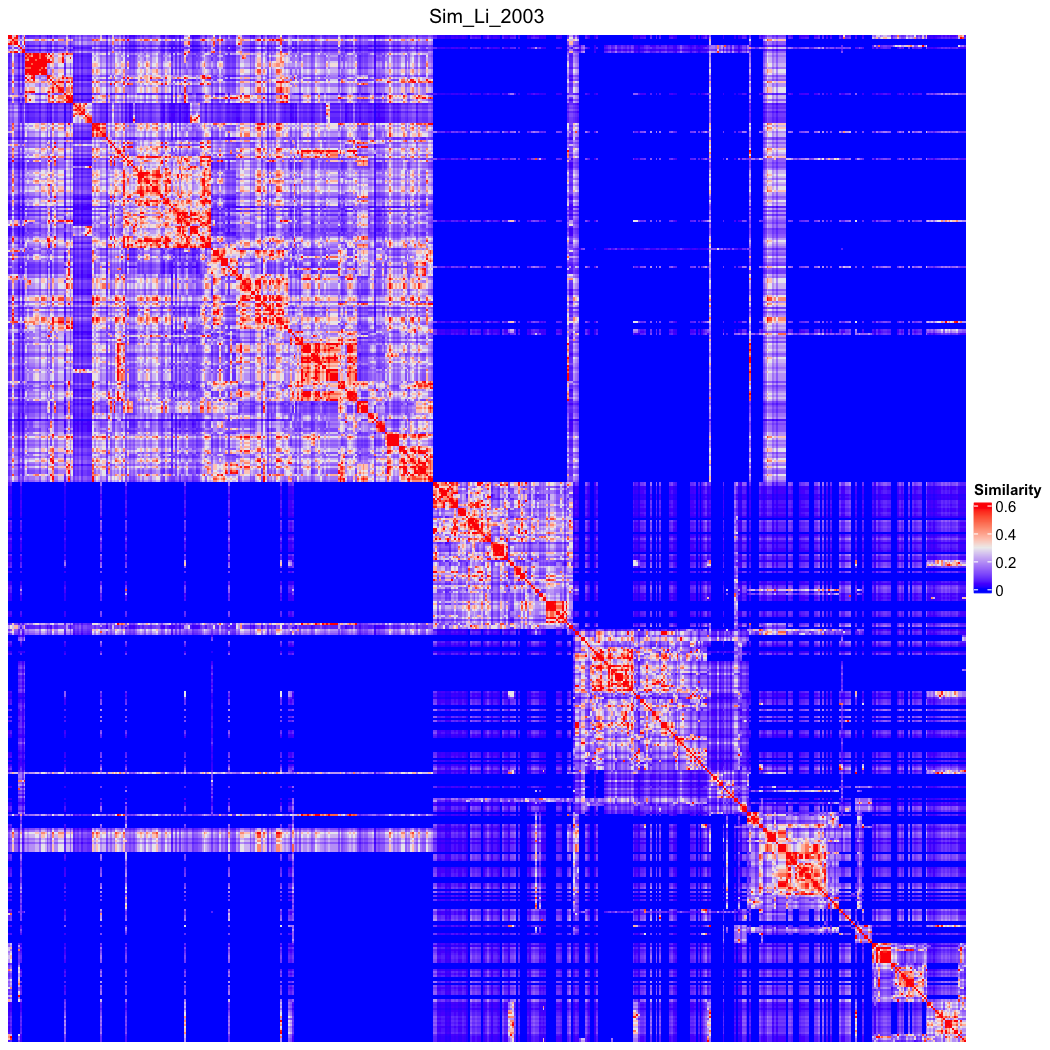

Supplement: Supplementary file 4 — Supplementary Material 4. Compare semantic similarity methods [file 12864_2024_10759_MOESM4_ESM.zip › suppl4_compare_sim_methods/image/go_bp_random_500_sim_Sim_Li_2003_Lin_order.png]

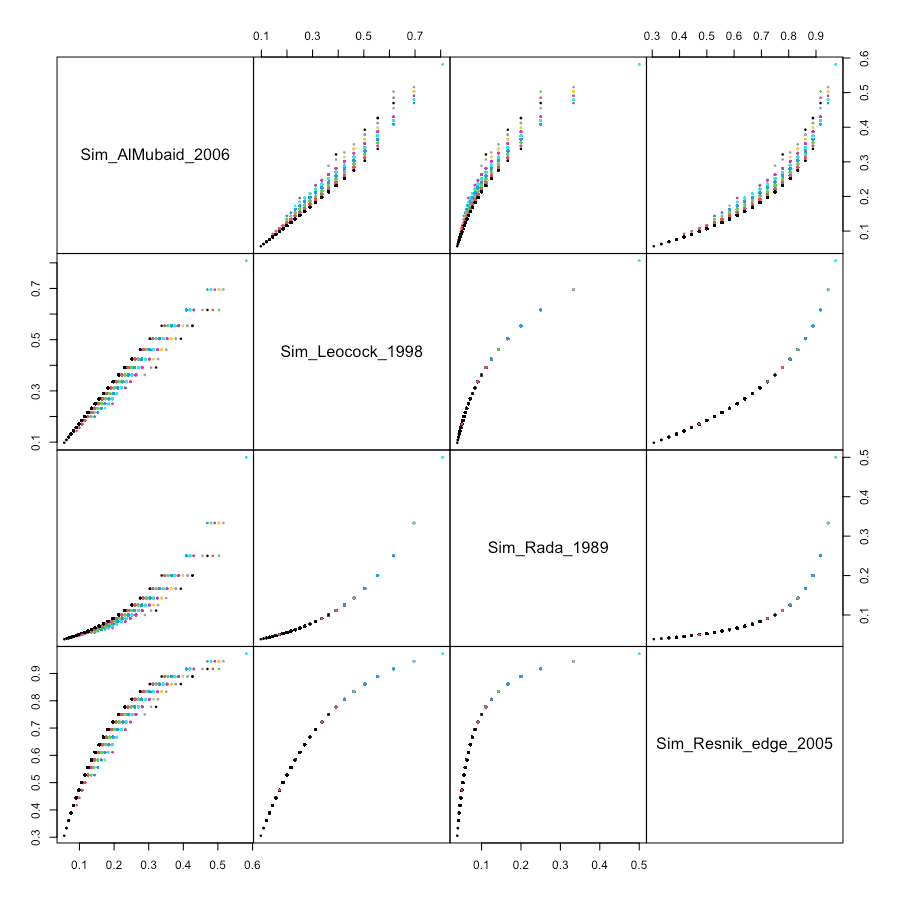

Supplement: Supplementary file 4 — Supplementary Material 4. Compare semantic similarity methods [file 12864_2024_10759_MOESM4_ESM.zip › suppl4_compare_sim_methods/image/scatterplot_group_5.png]

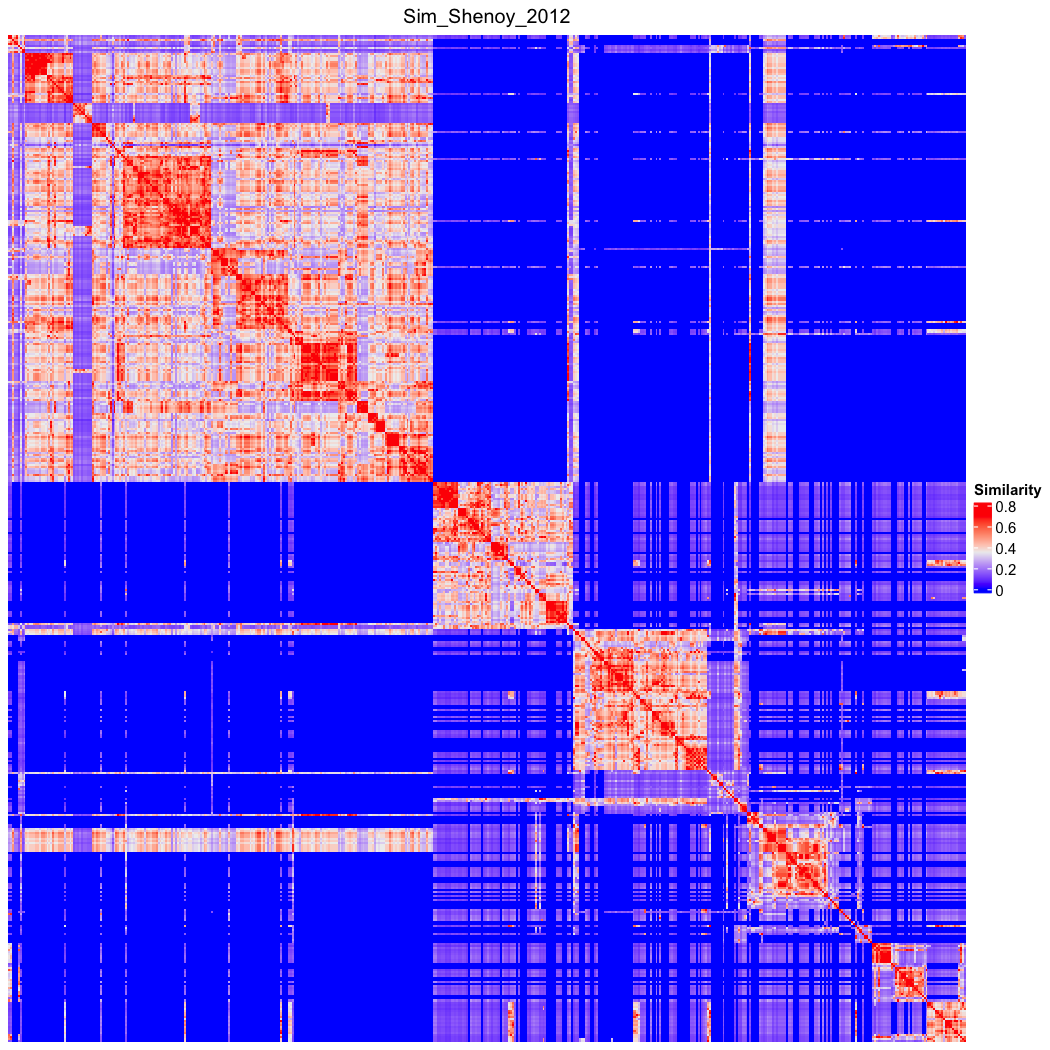

Supplement: Supplementary file 4 — Supplementary Material 4. Compare semantic similarity methods [file 12864_2024_10759_MOESM4_ESM.zip › suppl4_compare_sim_methods/image/go_bp_random_500_sim_Sim_Shenoy_2012_Lin_order.png]

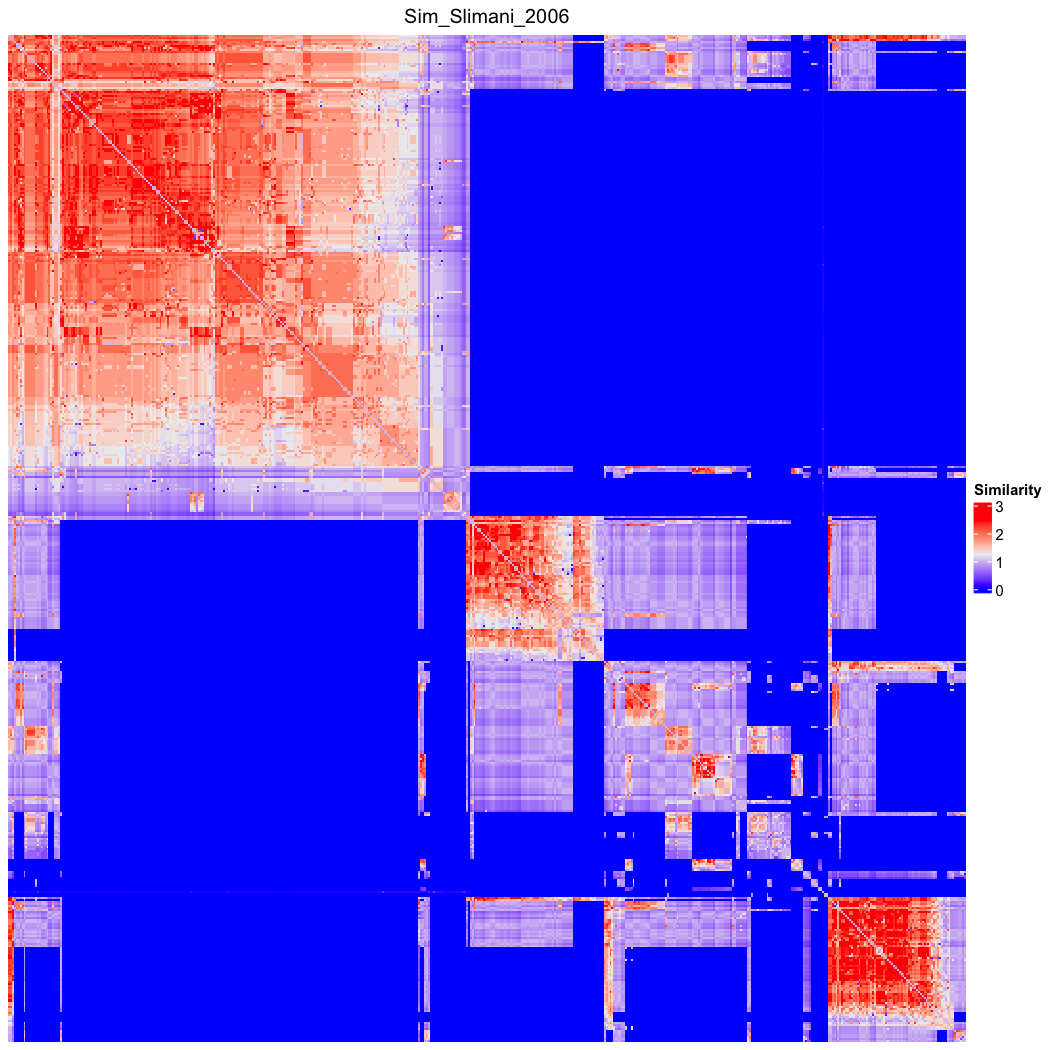

Supplement: Supplementary file 4 — Supplementary Material 4. Compare semantic similarity methods [file 12864_2024_10759_MOESM4_ESM.zip › suppl4_compare_sim_methods/image/go_bp_random_500_sim_Sim_Slimani_2006.png]

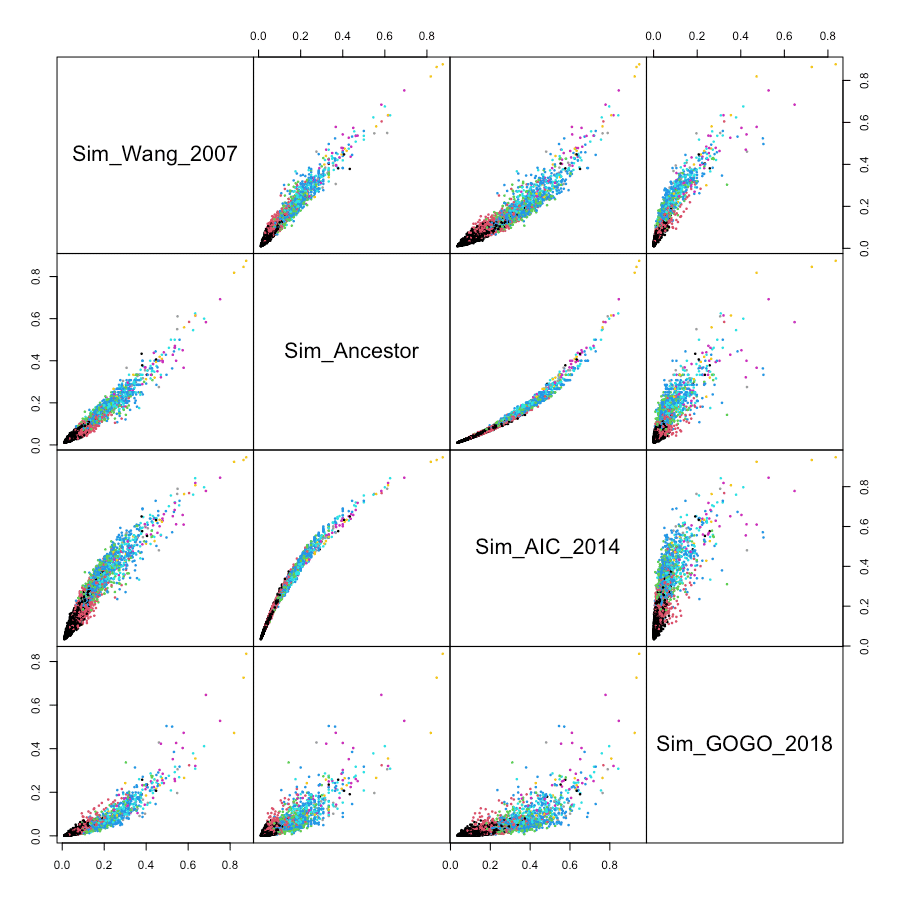

Supplement: Supplementary file 4 — Supplementary Material 4. Compare semantic similarity methods [file 12864_2024_10759_MOESM4_ESM.zip › suppl4_compare_sim_methods/image/scatterplot_group_4.png]

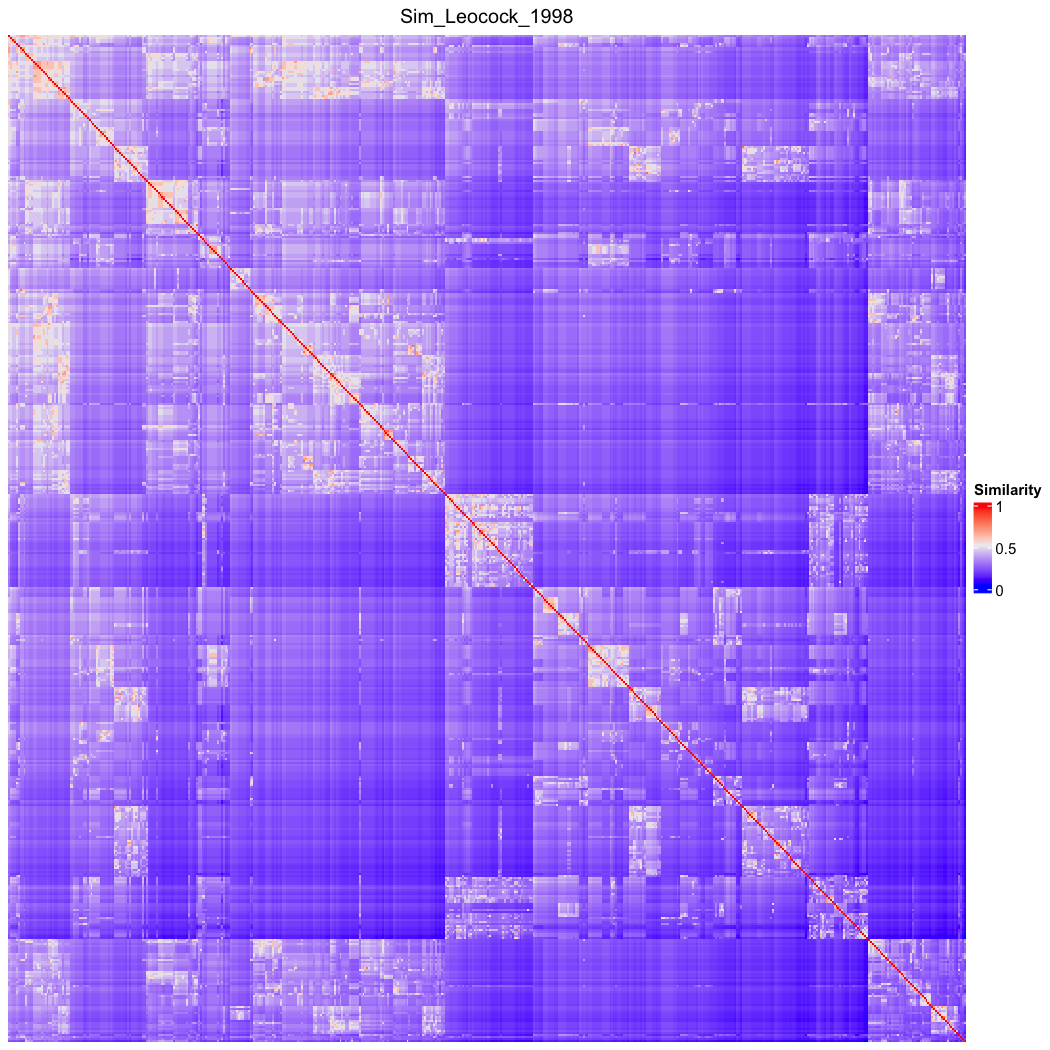

Supplement: Supplementary file 4 — Supplementary Material 4. Compare semantic similarity methods [file 12864_2024_10759_MOESM4_ESM.zip › suppl4_compare_sim_methods/image/go_bp_random_500_sim_Sim_Leocock_1998.png]

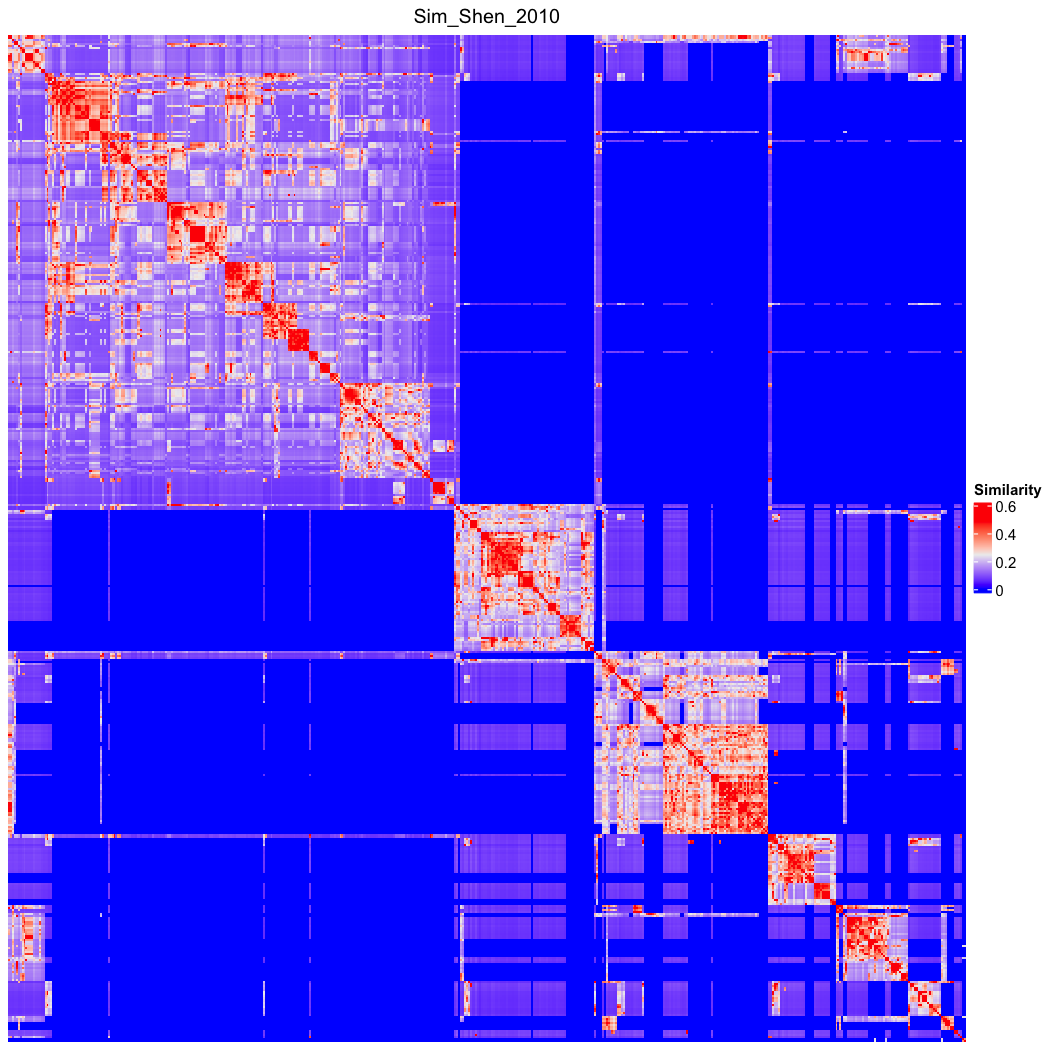

Supplement: Supplementary file 4 — Supplementary Material 4. Compare semantic similarity methods [file 12864_2024_10759_MOESM4_ESM.zip › suppl4_compare_sim_methods/image/go_bp_random_500_sim_Sim_Shen_2010.png]

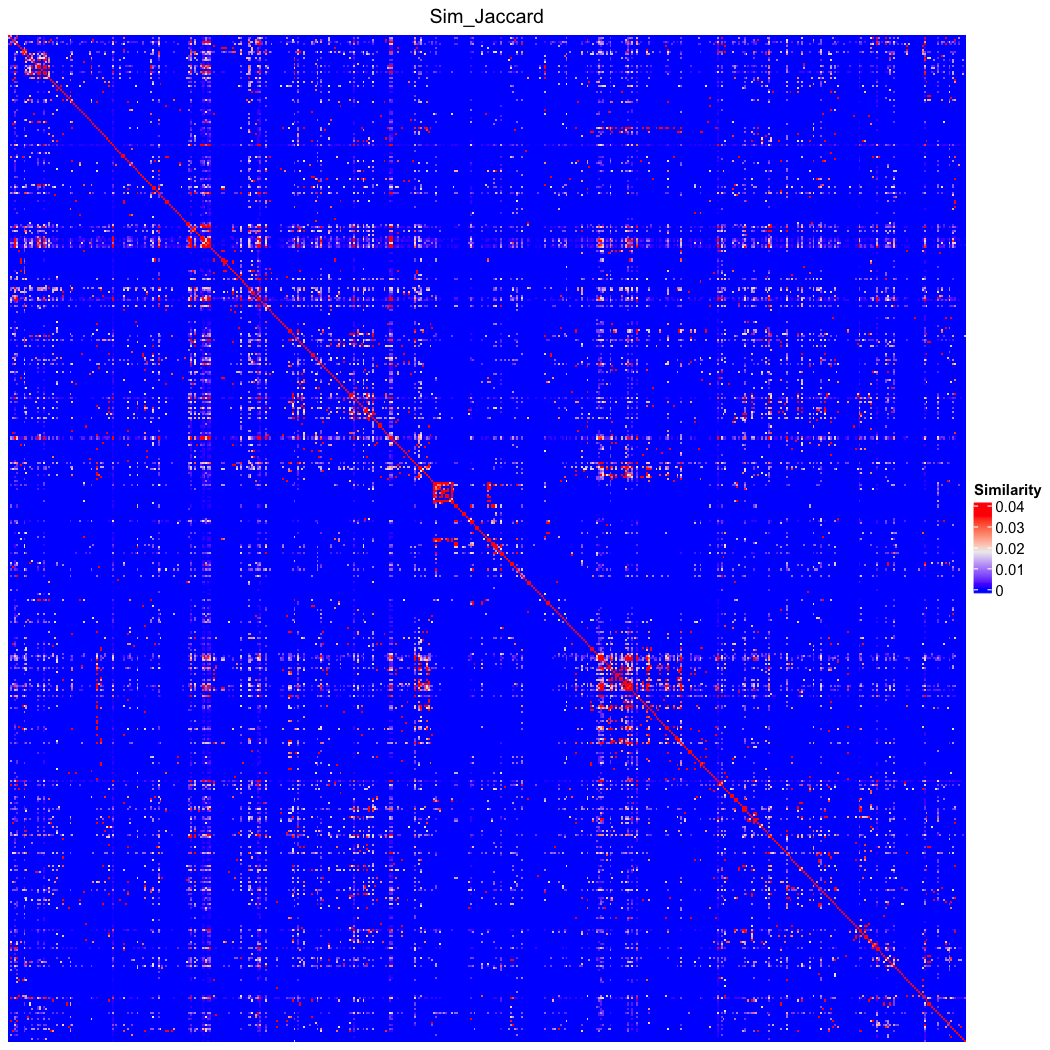

Supplement: Supplementary file 4 — Supplementary Material 4. Compare semantic similarity methods [file 12864_2024_10759_MOESM4_ESM.zip › suppl4_compare_sim_methods/image/go_bp_random_500_sim_Sim_Jaccard_Lin_order.png]

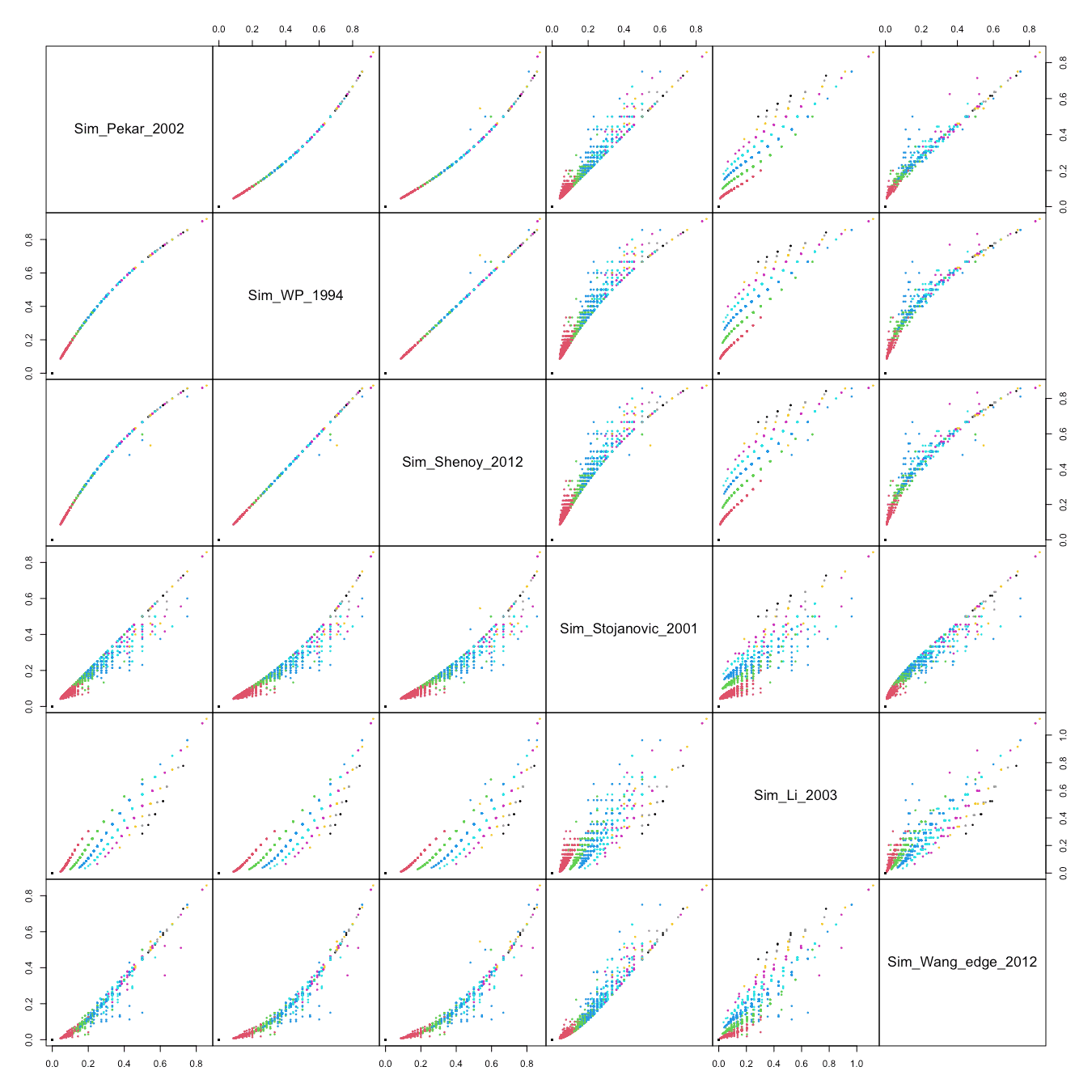

Supplement: Supplementary file 4 — Supplementary Material 4. Compare semantic similarity methods [file 12864_2024_10759_MOESM4_ESM.zip › suppl4_compare_sim_methods/image/scatterplot_group_3.png]

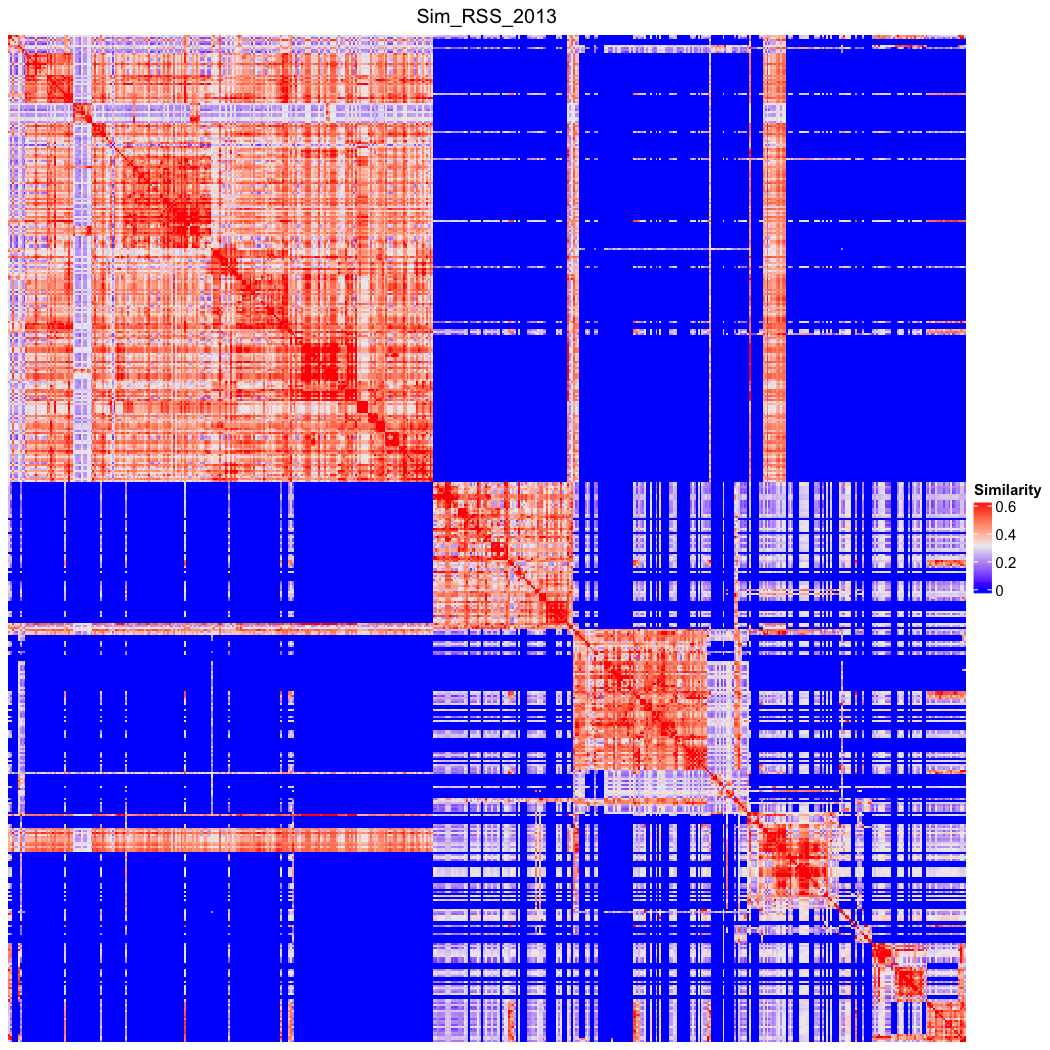

Supplement: Supplementary file 4 — Supplementary Material 4. Compare semantic similarity methods [file 12864_2024_10759_MOESM4_ESM.zip › suppl4_compare_sim_methods/image/go_bp_random_500_sim_Sim_RSS_2013_Lin_order.png]

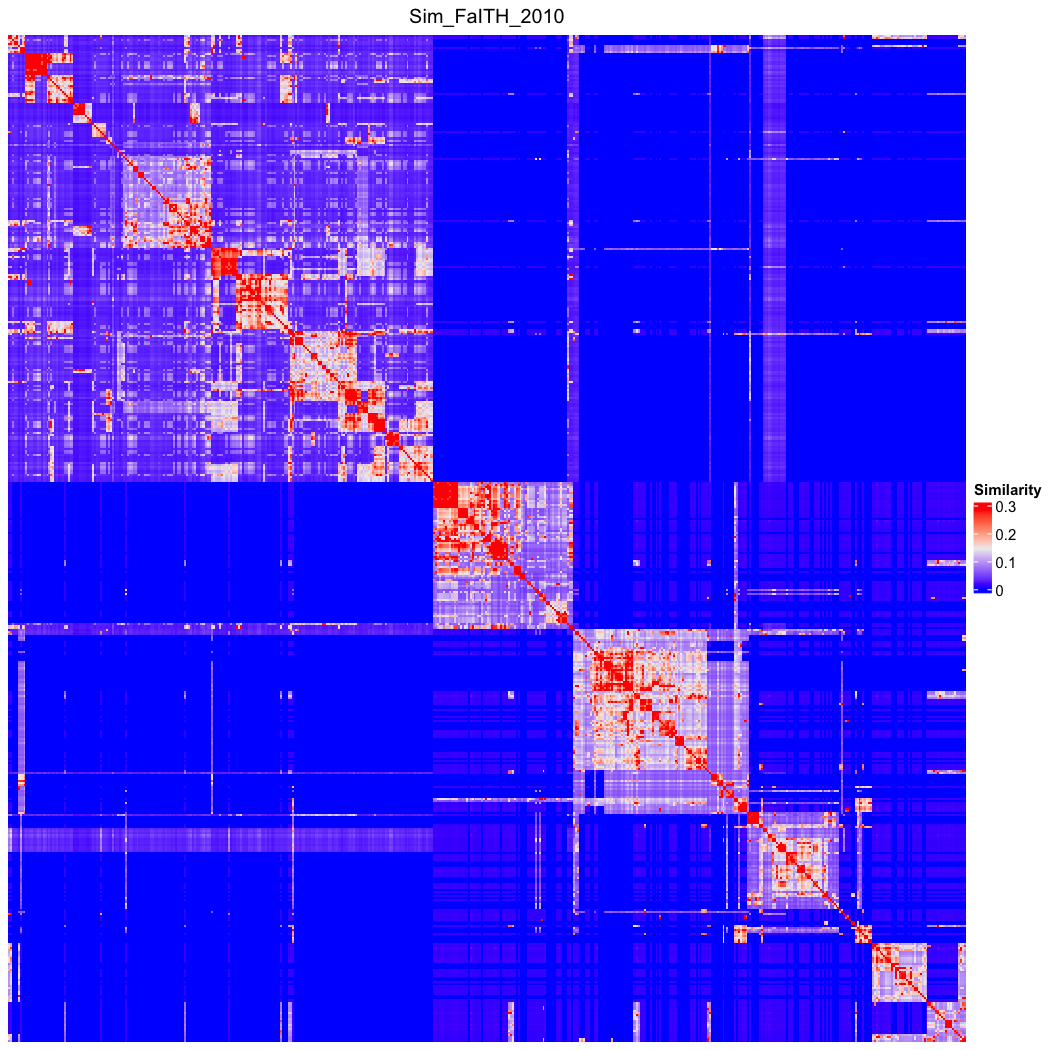

Supplement: Supplementary file 4 — Supplementary Material 4. Compare semantic similarity methods [file 12864_2024_10759_MOESM4_ESM.zip › suppl4_compare_sim_methods/image/go_bp_random_500_sim_Sim_FaITH_2010_Lin_order.png]

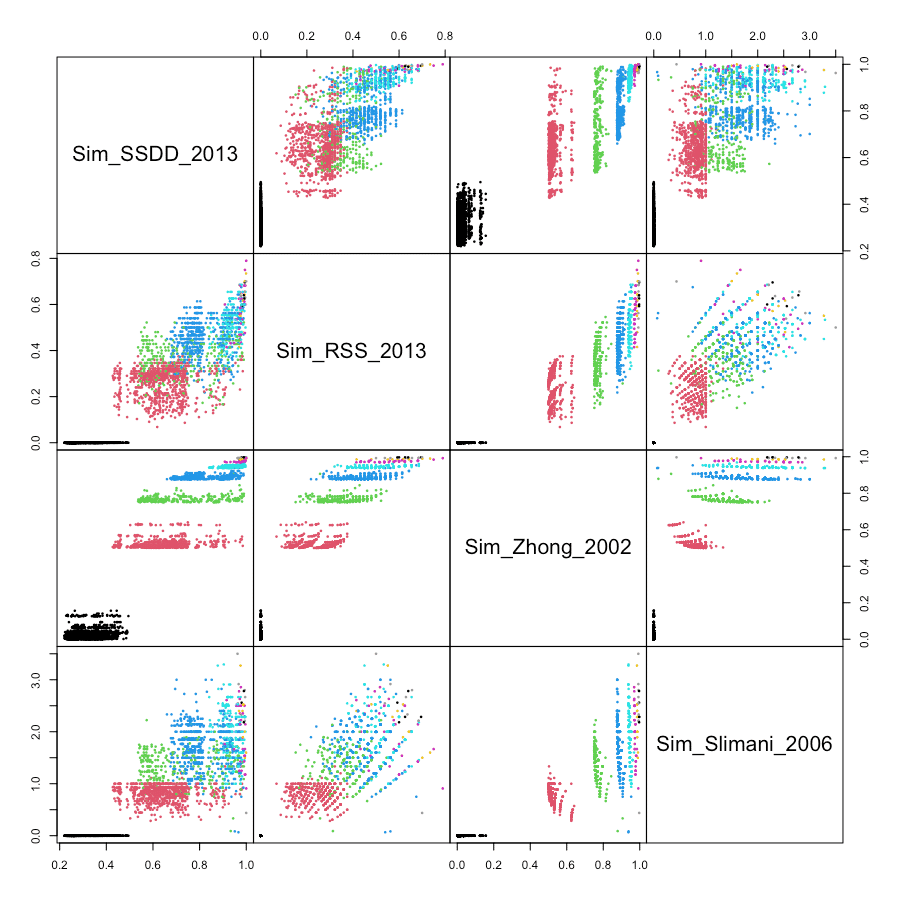

Supplement: Supplementary file 4 — Supplementary Material 4. Compare semantic similarity methods [file 12864_2024_10759_MOESM4_ESM.zip › suppl4_compare_sim_methods/image/scatterplot_group_2.png]

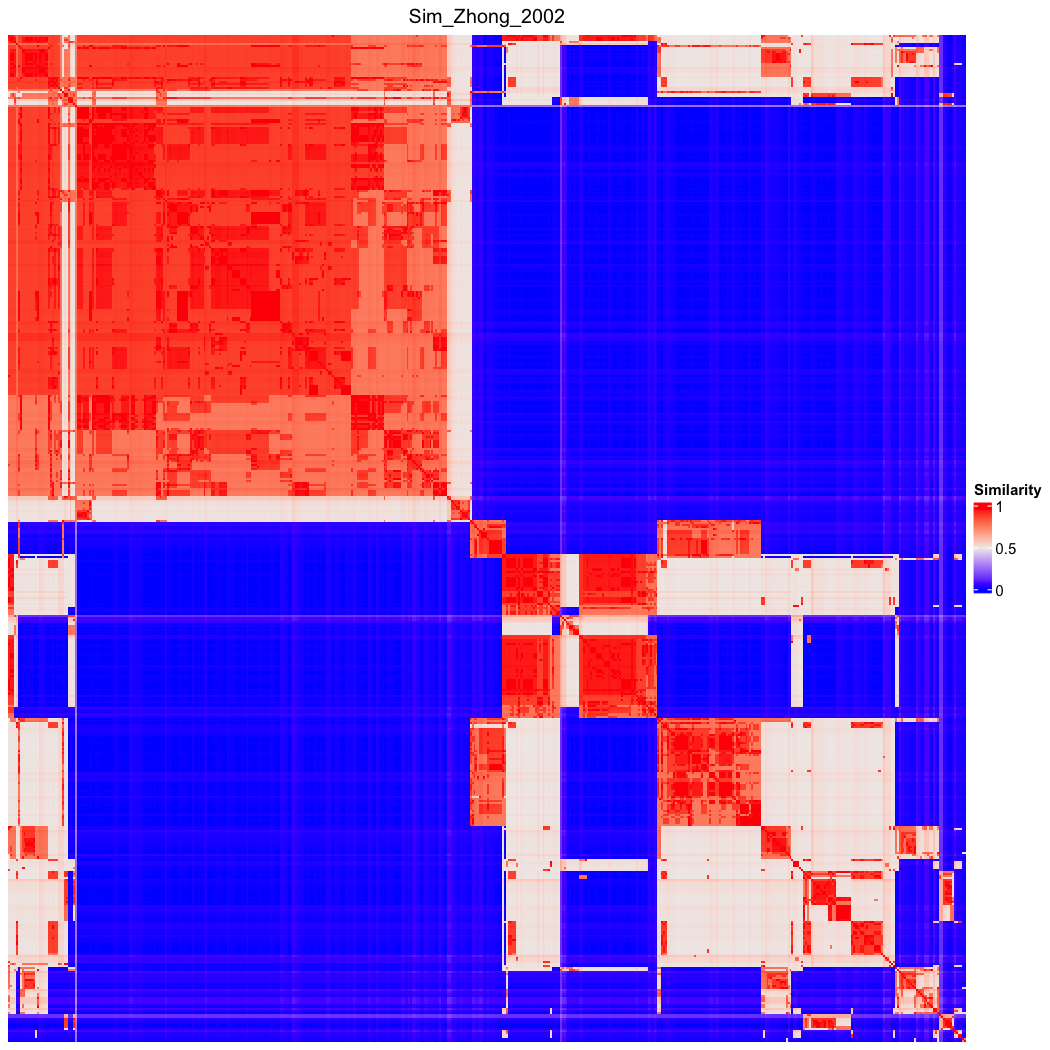

Supplement: Supplementary file 4 — Supplementary Material 4. Compare semantic similarity methods [file 12864_2024_10759_MOESM4_ESM.zip › suppl4_compare_sim_methods/image/go_bp_random_500_sim_Sim_Zhong_2002.png]

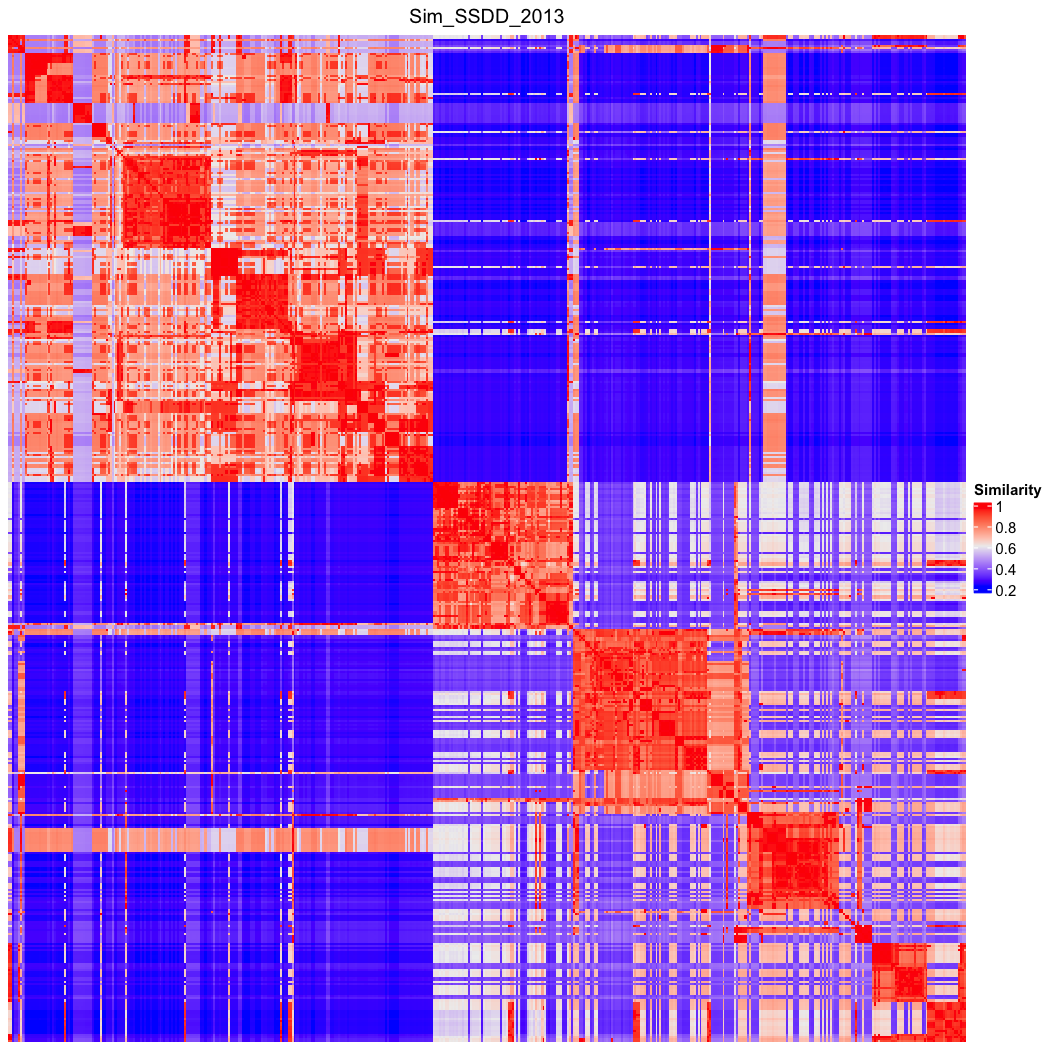

Supplement: Supplementary file 4 — Supplementary Material 4. Compare semantic similarity methods [file 12864_2024_10759_MOESM4_ESM.zip › suppl4_compare_sim_methods/image/go_bp_random_500_sim_Sim_SSDD_2013_Lin_order.png]

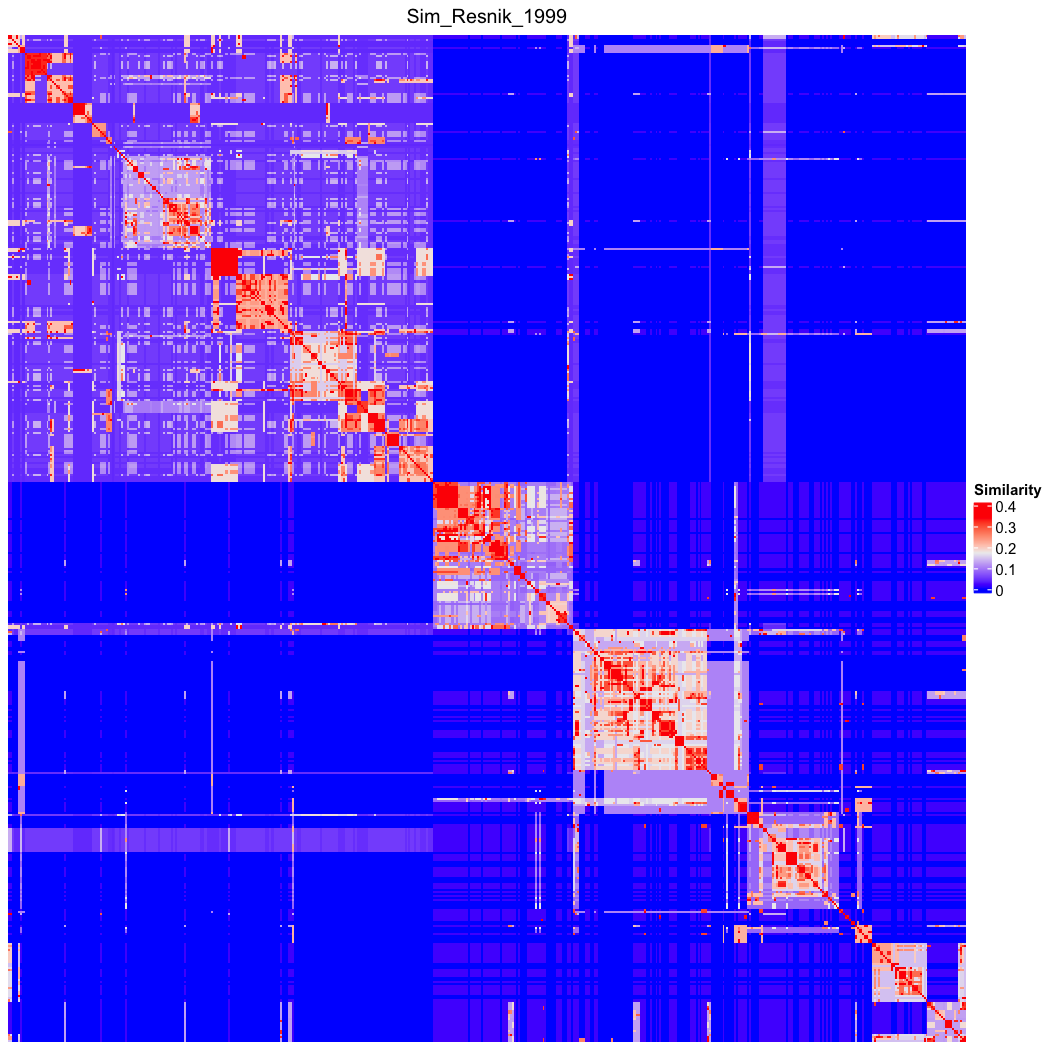

Supplement: Supplementary file 4 — Supplementary Material 4. Compare semantic similarity methods [file 12864_2024_10759_MOESM4_ESM.zip › suppl4_compare_sim_methods/image/go_bp_random_500_sim_Sim_Resnik_1999_Lin_order.png]

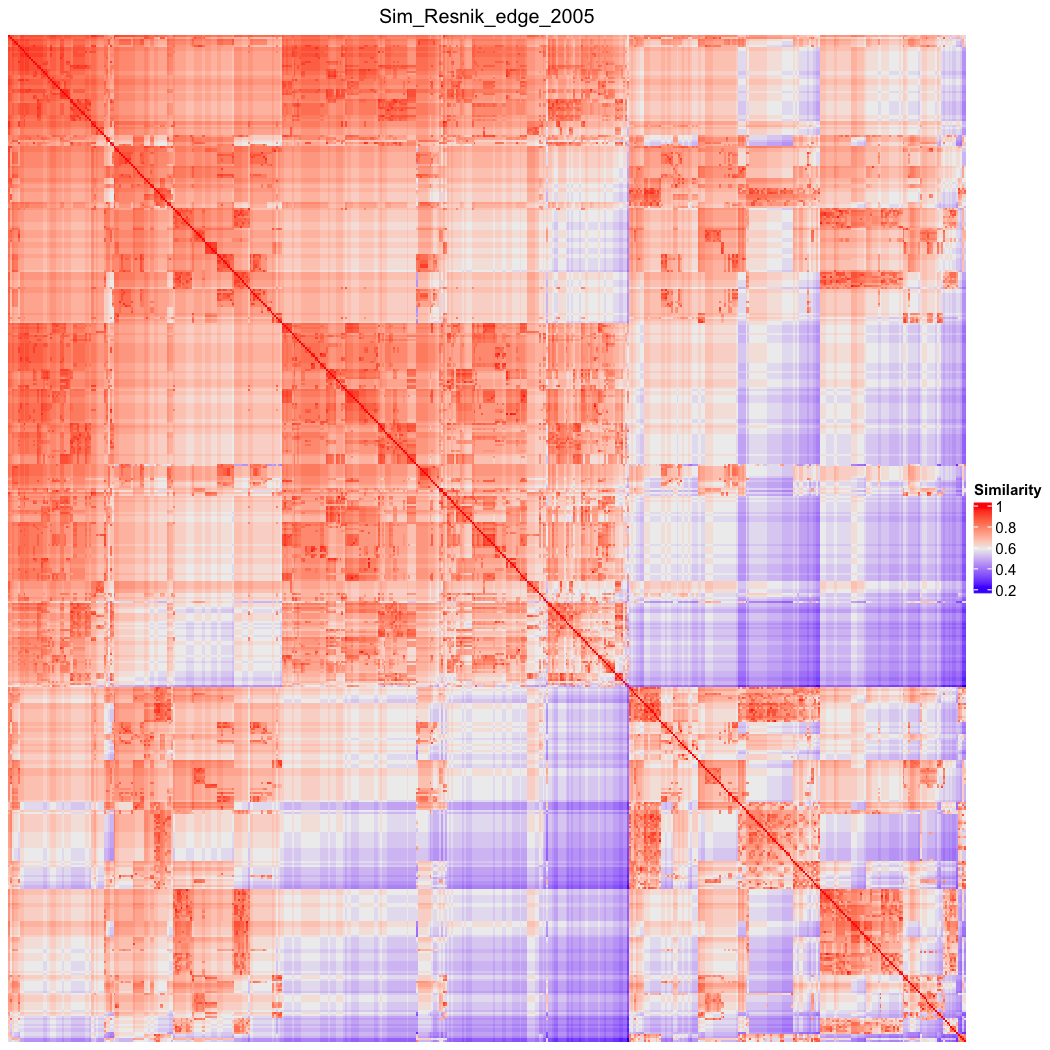

Supplement: Supplementary file 4 — Supplementary Material 4. Compare semantic similarity methods [file 12864_2024_10759_MOESM4_ESM.zip › suppl4_compare_sim_methods/image/go_bp_random_500_sim_Sim_Resnik_edge_2005.png]

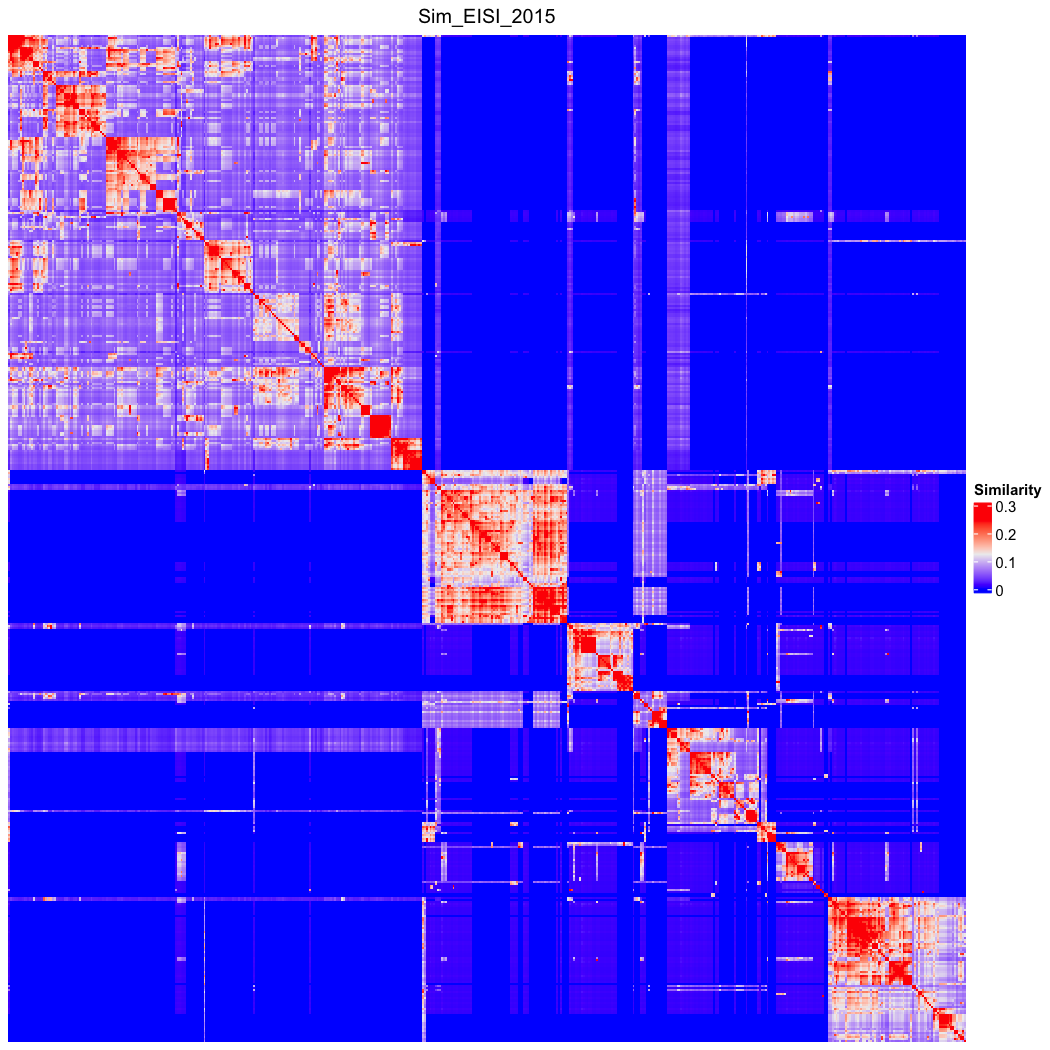

Supplement: Supplementary file 4 — Supplementary Material 4. Compare semantic similarity methods [file 12864_2024_10759_MOESM4_ESM.zip › suppl4_compare_sim_methods/image/go_bp_random_500_sim_Sim_EISI_2015.png]

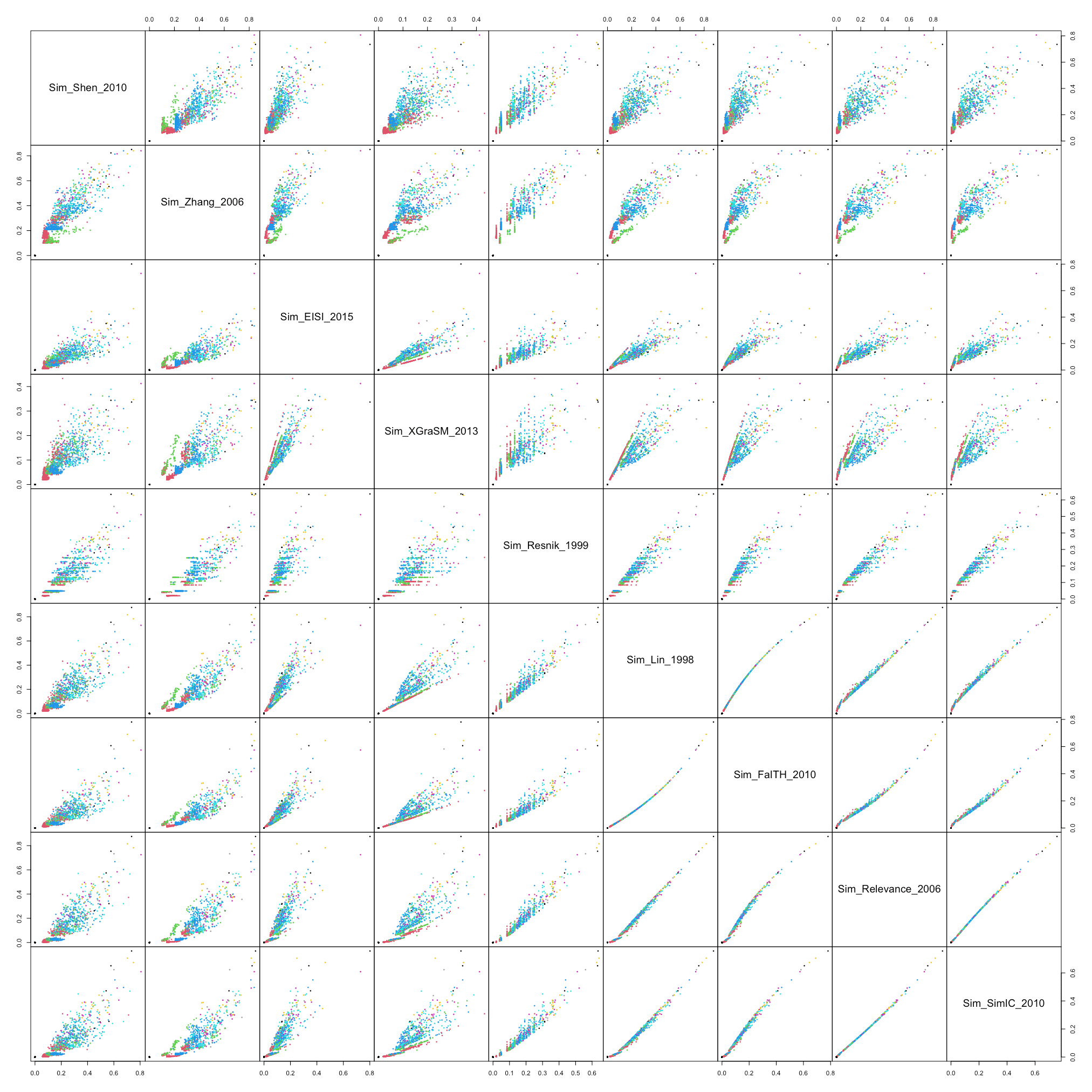

Supplement: Supplementary file 4 — Supplementary Material 4. Compare semantic similarity methods [file 12864_2024_10759_MOESM4_ESM.zip › suppl4_compare_sim_methods/image/scatterplot_group_1.png]

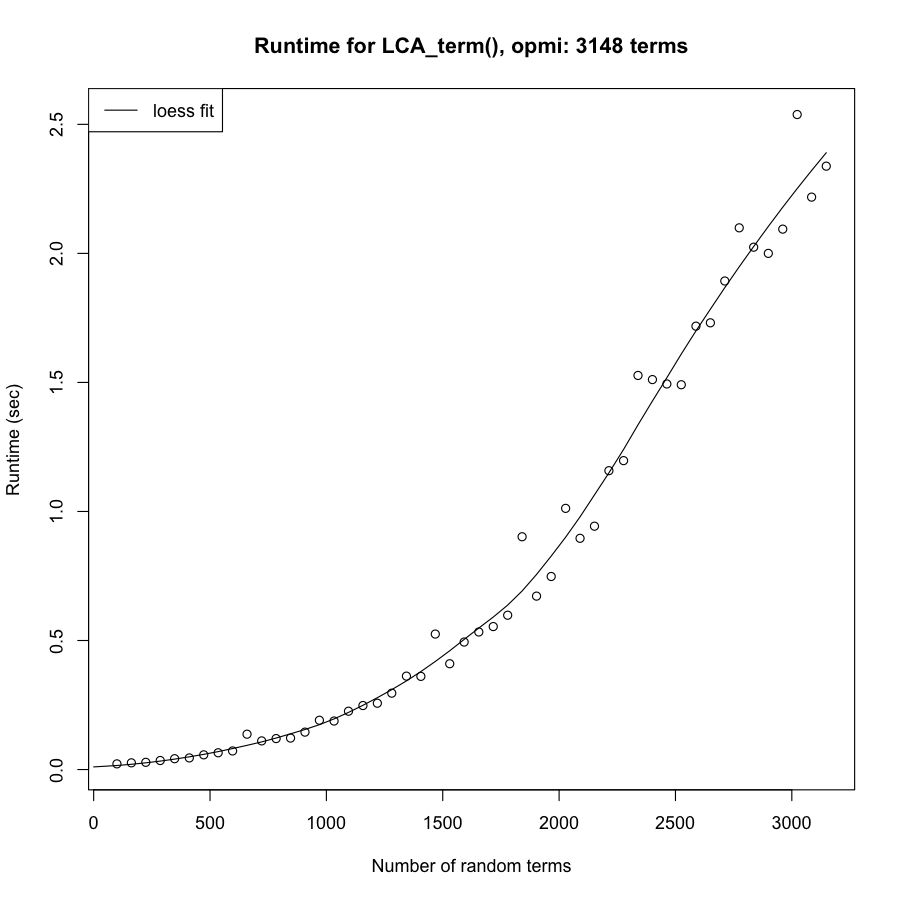

Supplement: Supplementary file 6 — Supplementary Material 6. OBO Foundry gallery [file 12864_2024_10759_MOESM6_ESM.zip › suppl6_OBOFoundry_gallery/image/OBOFoundry_opmi_runtime.png]

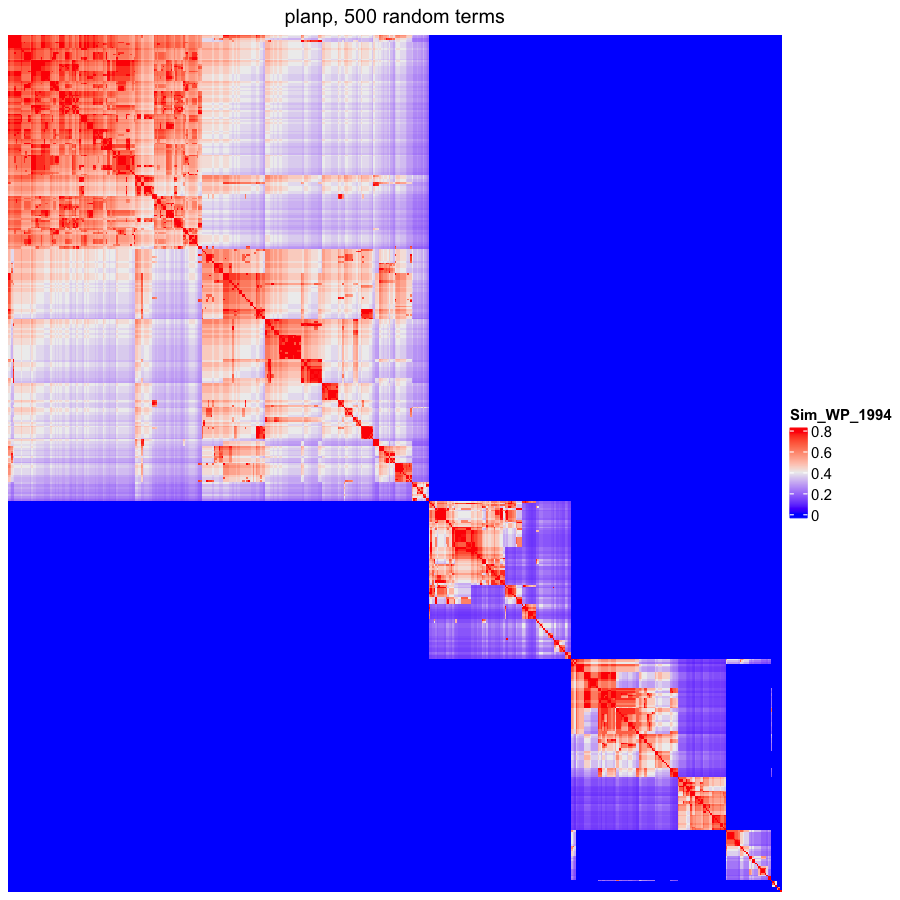

Supplement: Supplementary file 6 — Supplementary Material 6. OBO Foundry gallery [file 12864_2024_10759_MOESM6_ESM.zip › suppl6_OBOFoundry_gallery/image/OBOFoundry_planp_heatmap.png]

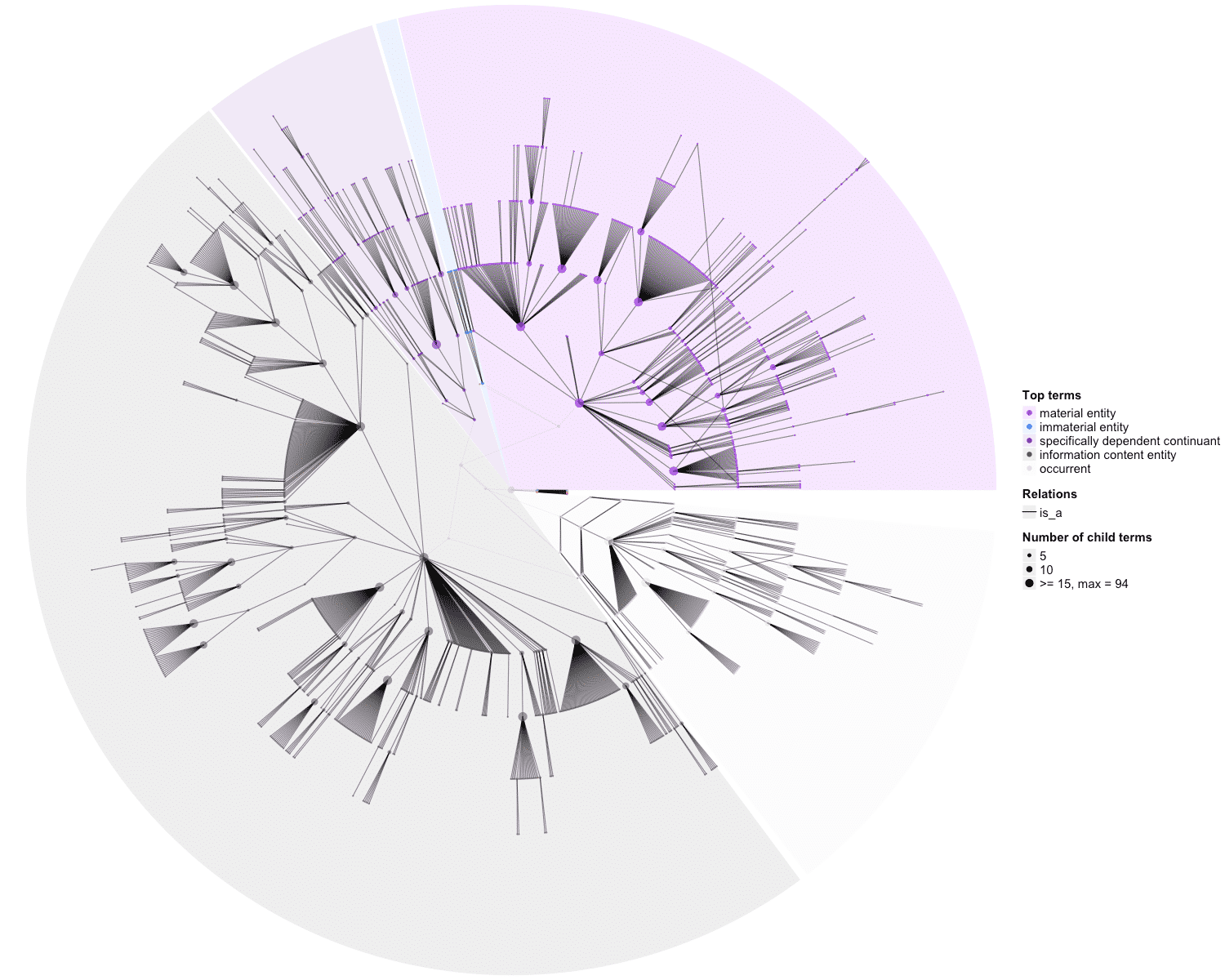

Supplement: Supplementary file 6 — Supplementary Material 6. OBO Foundry gallery [file 12864_2024_10759_MOESM6_ESM.zip › suppl6_OBOFoundry_gallery/image/OBOFoundry_obib.png]

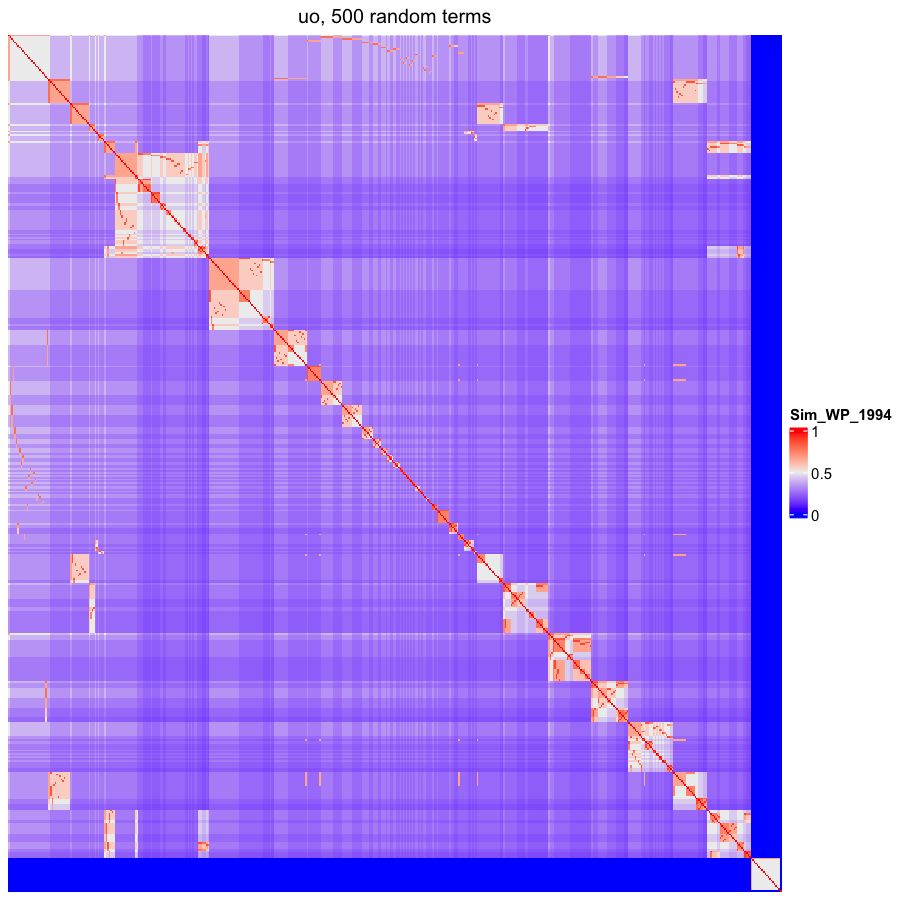

Supplement: Supplementary file 6 — Supplementary Material 6. OBO Foundry gallery [file 12864_2024_10759_MOESM6_ESM.zip › suppl6_OBOFoundry_gallery/image/OBOFoundry_uo_heatmap.png]

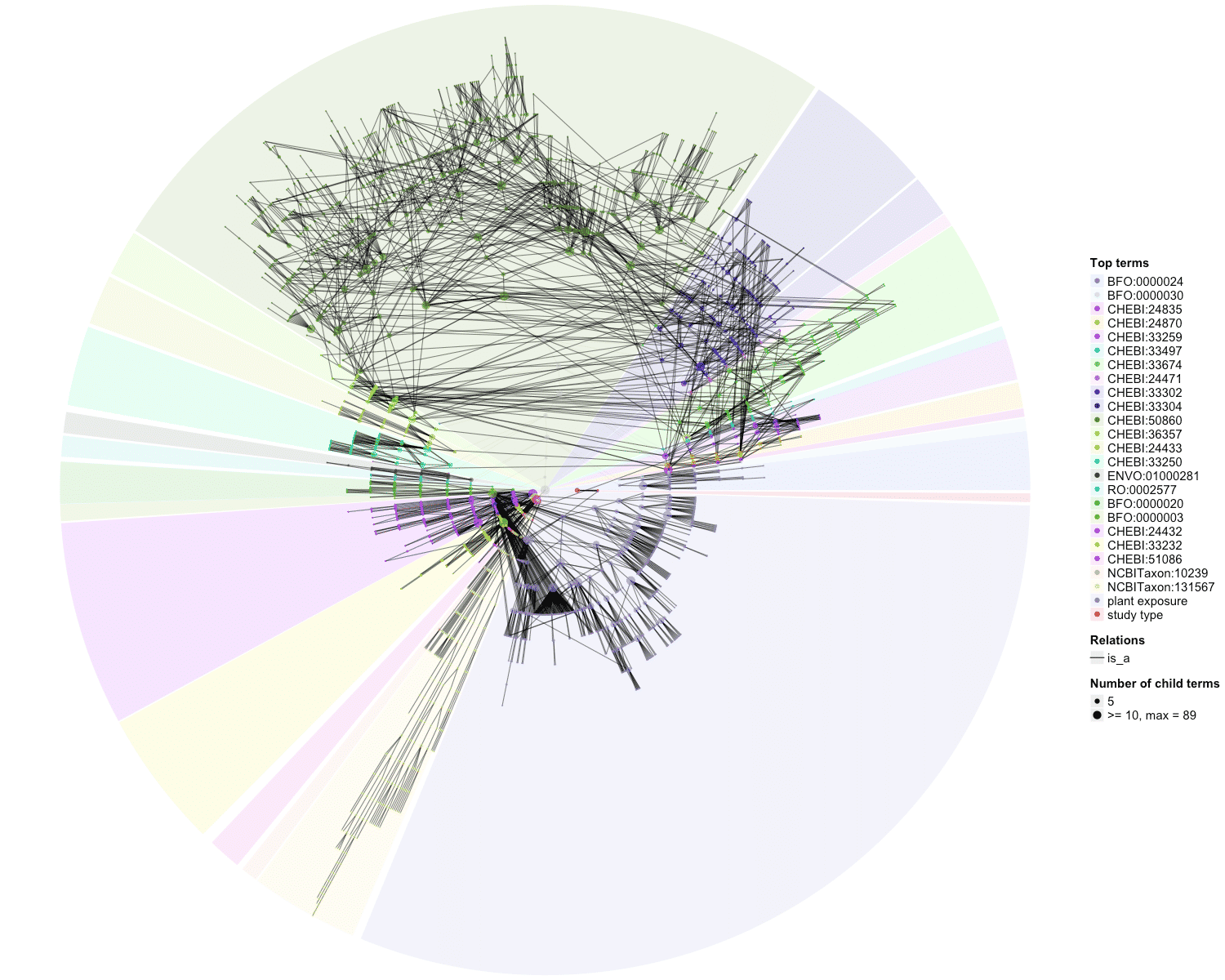

Supplement: Supplementary file 6 — Supplementary Material 6. OBO Foundry gallery [file 12864_2024_10759_MOESM6_ESM.zip › suppl6_OBOFoundry_gallery/image/OBOFoundry_peco.png]

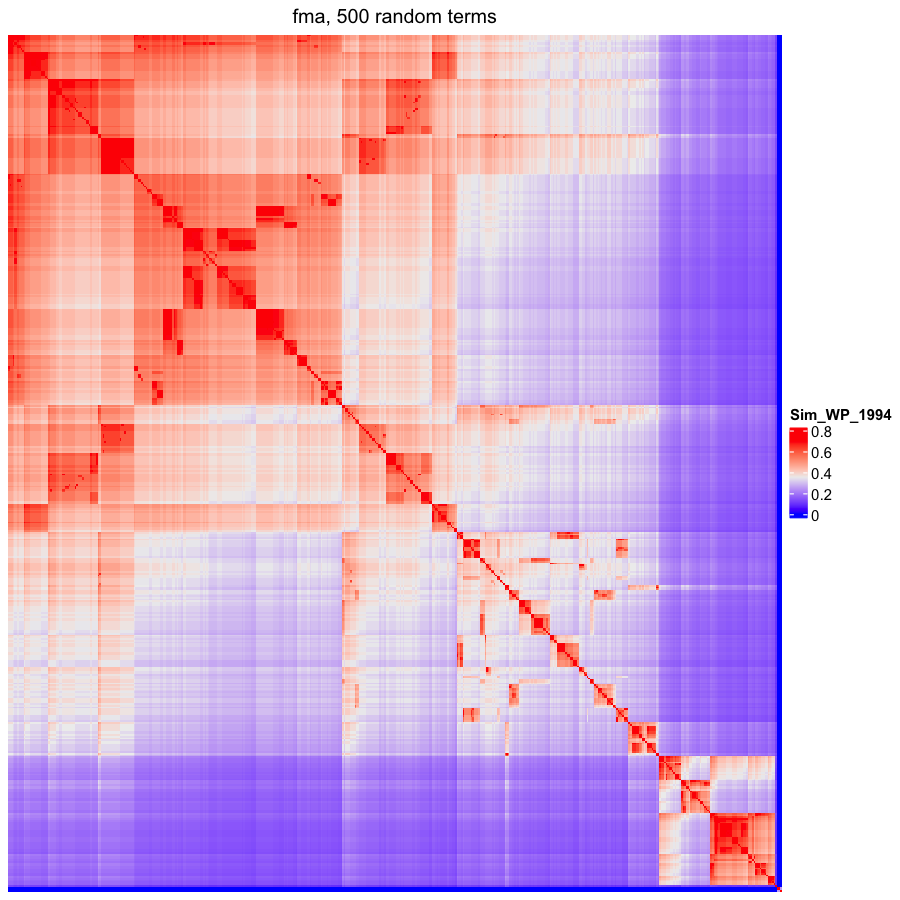

Supplement: Supplementary file 6 — Supplementary Material 6. OBO Foundry gallery [file 12864_2024_10759_MOESM6_ESM.zip › suppl6_OBOFoundry_gallery/image/OBOFoundry_fma_heatmap.png]

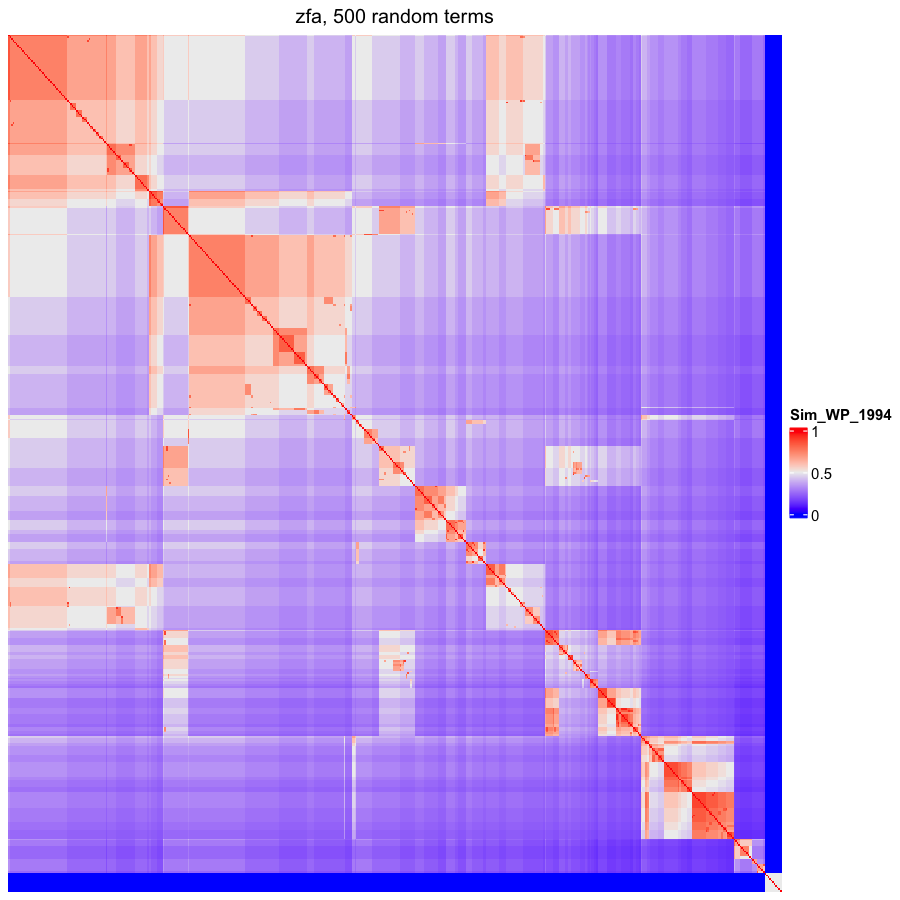

Supplement: Supplementary file 6 — Supplementary Material 6. OBO Foundry gallery [file 12864_2024_10759_MOESM6_ESM.zip › suppl6_OBOFoundry_gallery/image/OBOFoundry_zfa_heatmap.png]

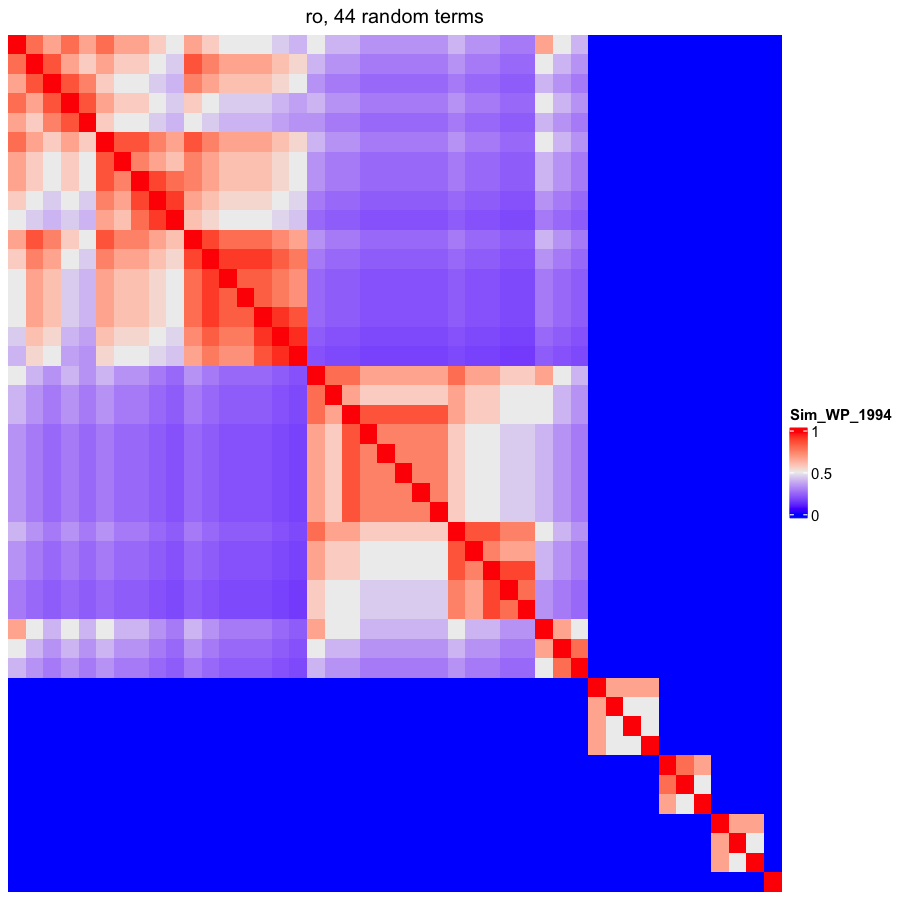

Supplement: Supplementary file 6 — Supplementary Material 6. OBO Foundry gallery [file 12864_2024_10759_MOESM6_ESM.zip › suppl6_OBOFoundry_gallery/image/OBOFoundry_ro_heatmap.png]

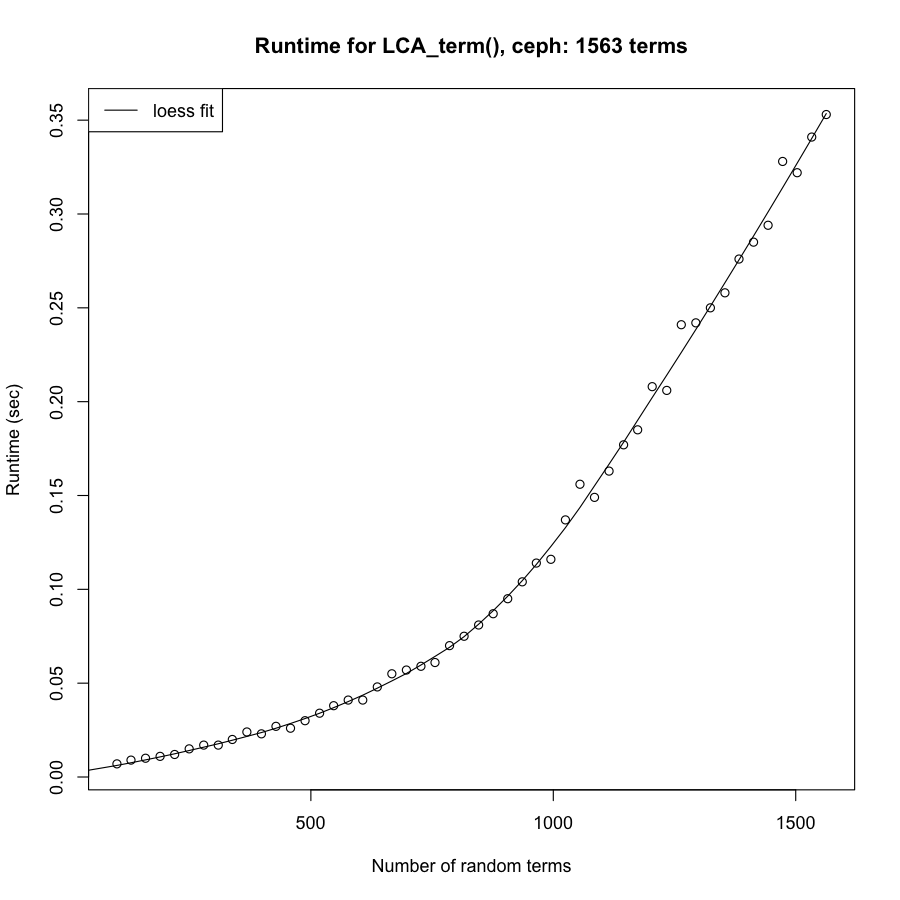

Supplement: Supplementary file 6 — Supplementary Material 6. OBO Foundry gallery [file 12864_2024_10759_MOESM6_ESM.zip › suppl6_OBOFoundry_gallery/image/OBOFoundry_ceph_runtime.png]

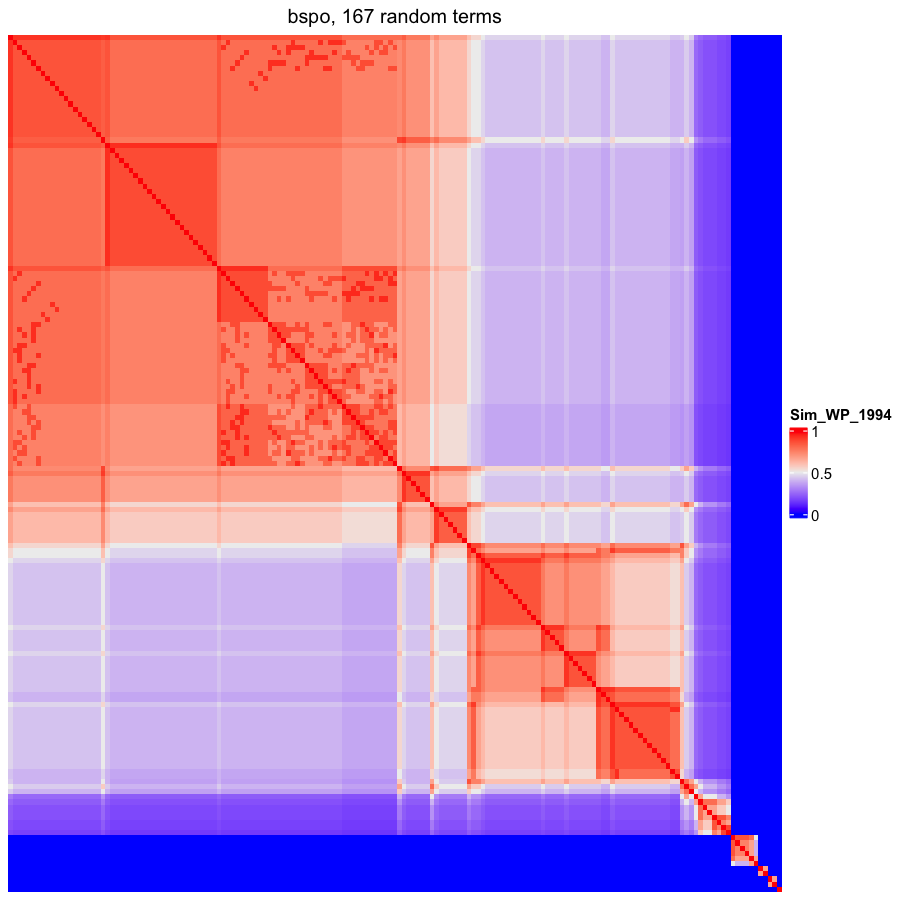

Supplement: Supplementary file 6 — Supplementary Material 6. OBO Foundry gallery [file 12864_2024_10759_MOESM6_ESM.zip › suppl6_OBOFoundry_gallery/image/OBOFoundry_bspo_heatmap.png]

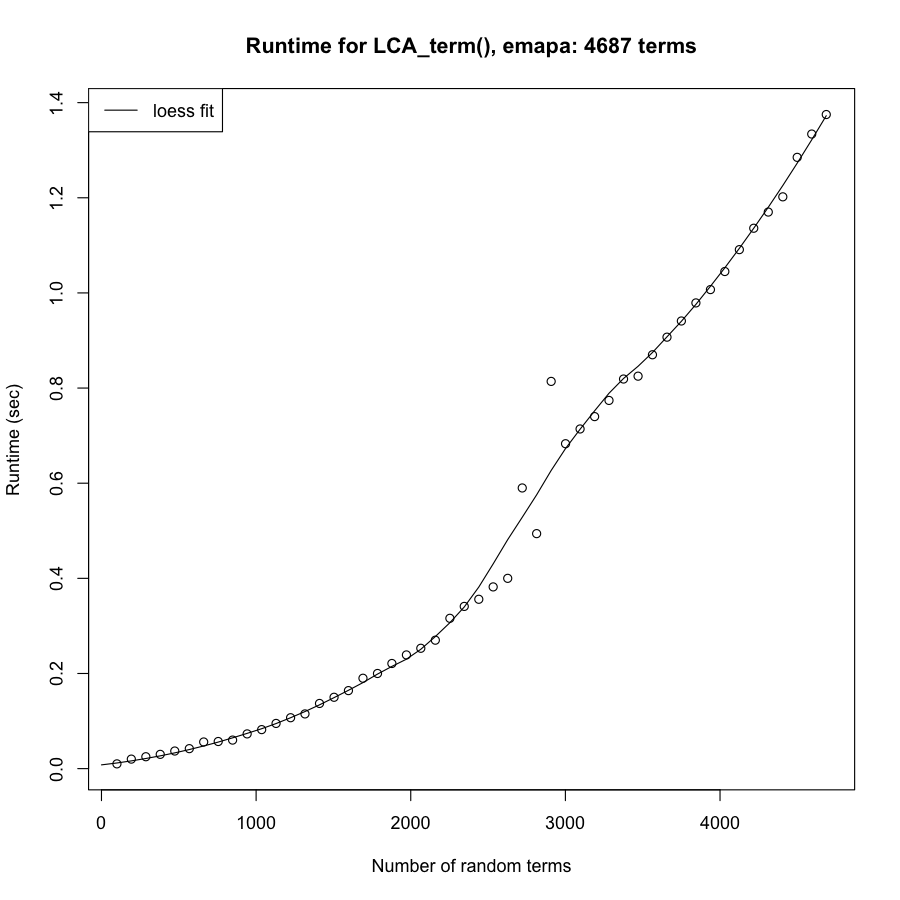

Supplement: Supplementary file 6 — Supplementary Material 6. OBO Foundry gallery [file 12864_2024_10759_MOESM6_ESM.zip › suppl6_OBOFoundry_gallery/image/OBOFoundry_emapa_runtime.png]

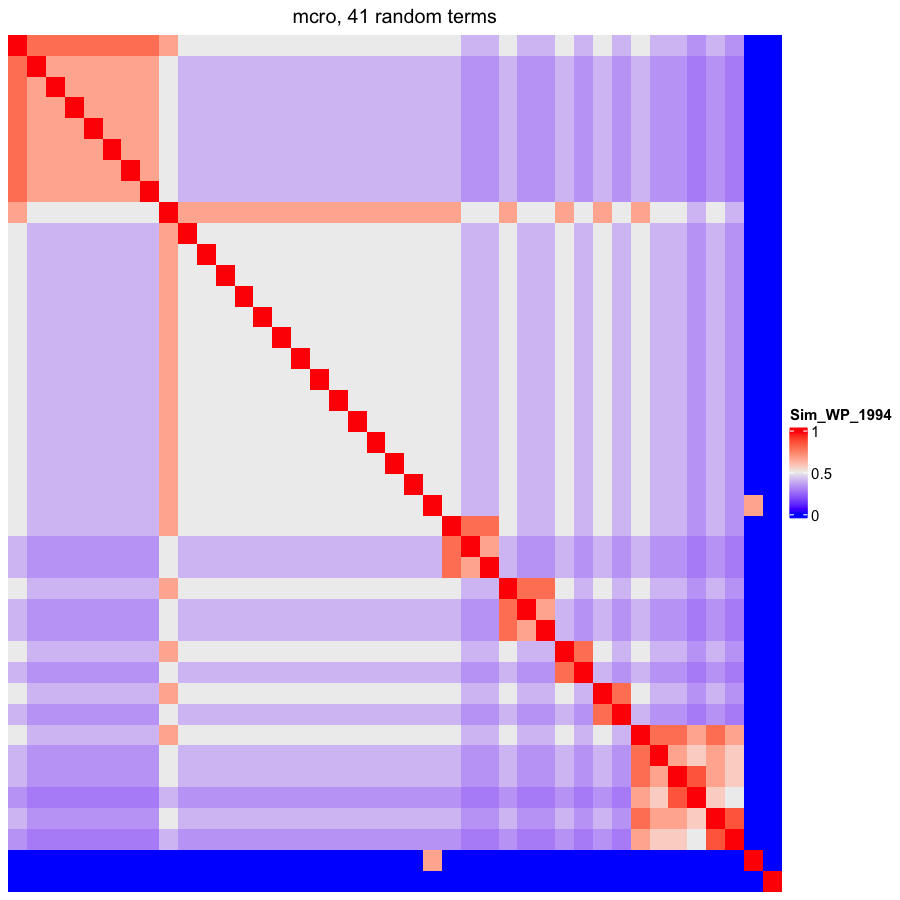

Supplement: Supplementary file 6 — Supplementary Material 6. OBO Foundry gallery [file 12864_2024_10759_MOESM6_ESM.zip › suppl6_OBOFoundry_gallery/image/OBOFoundry_mcro_heatmap.png]

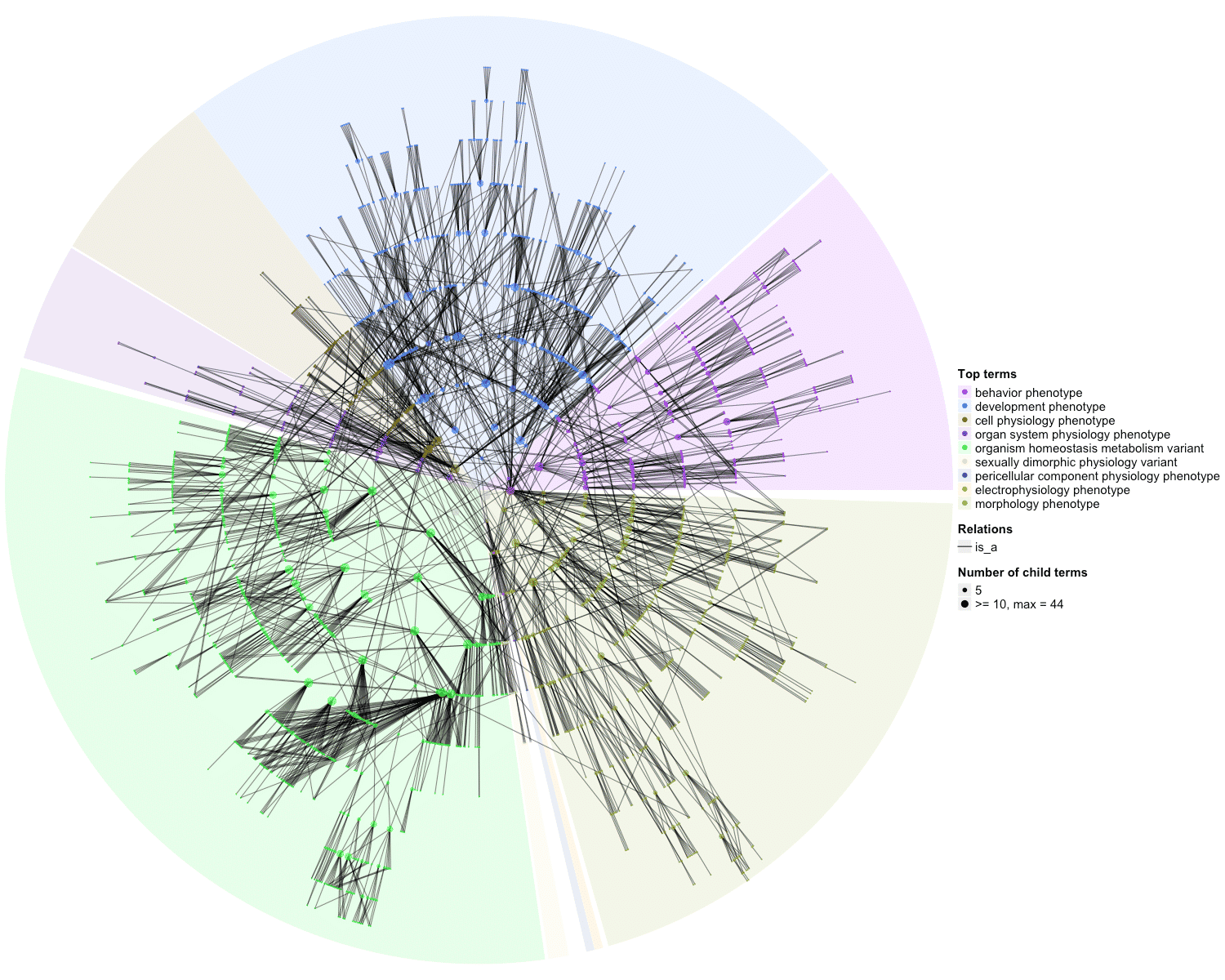

Supplement: Supplementary file 6 — Supplementary Material 6. OBO Foundry gallery [file 12864_2024_10759_MOESM6_ESM.zip › suppl6_OBOFoundry_gallery/image/OBOFoundry_wbphenotype.png]

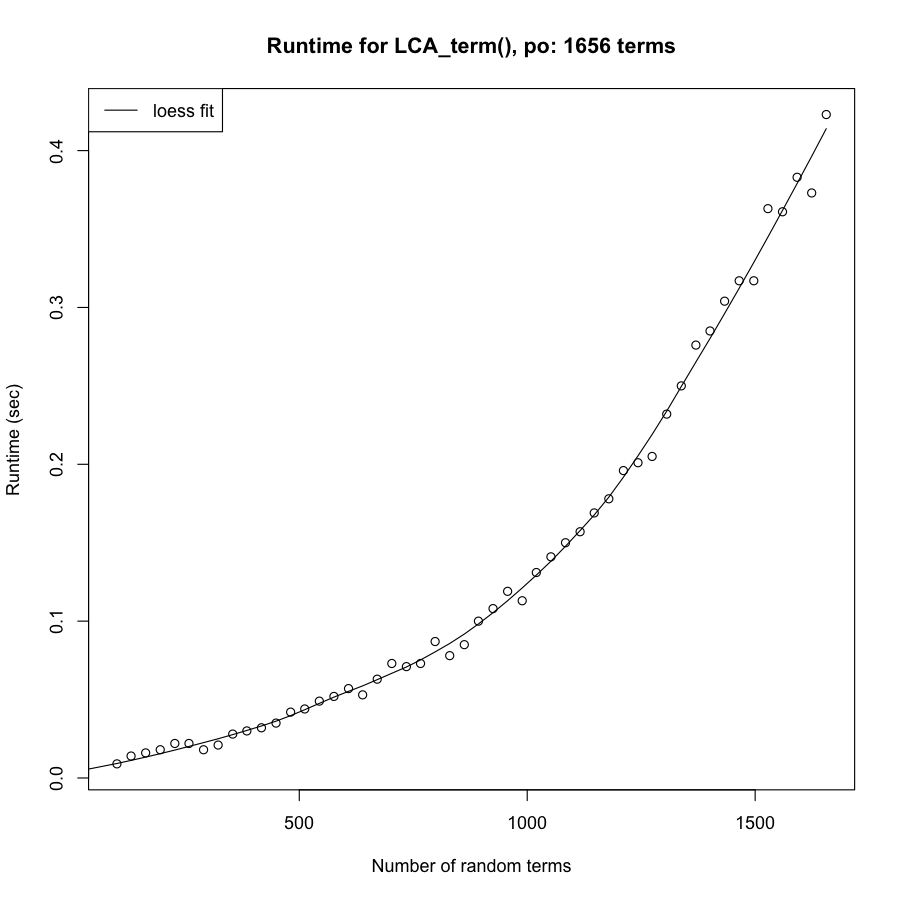

Supplement: Supplementary file 6 — Supplementary Material 6. OBO Foundry gallery [file 12864_2024_10759_MOESM6_ESM.zip › suppl6_OBOFoundry_gallery/image/OBOFoundry_po_runtime.png]

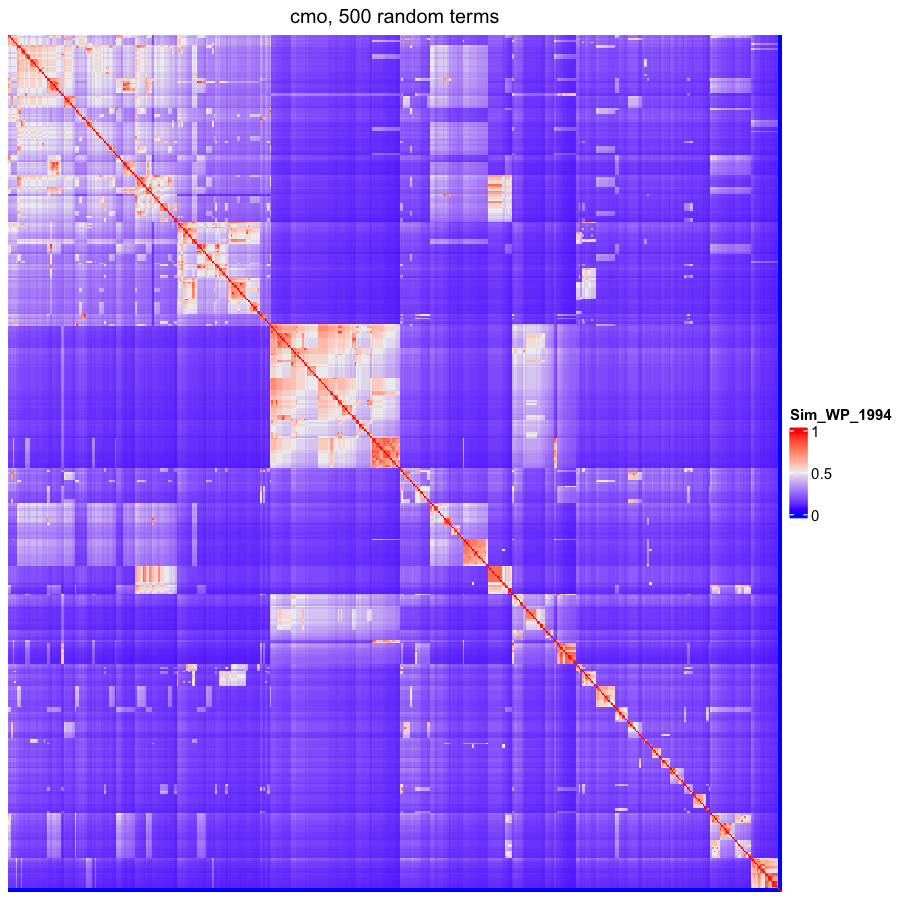

Supplement: Supplementary file 6 — Supplementary Material 6. OBO Foundry gallery [file 12864_2024_10759_MOESM6_ESM.zip › suppl6_OBOFoundry_gallery/image/OBOFoundry_cmo_heatmap.png]

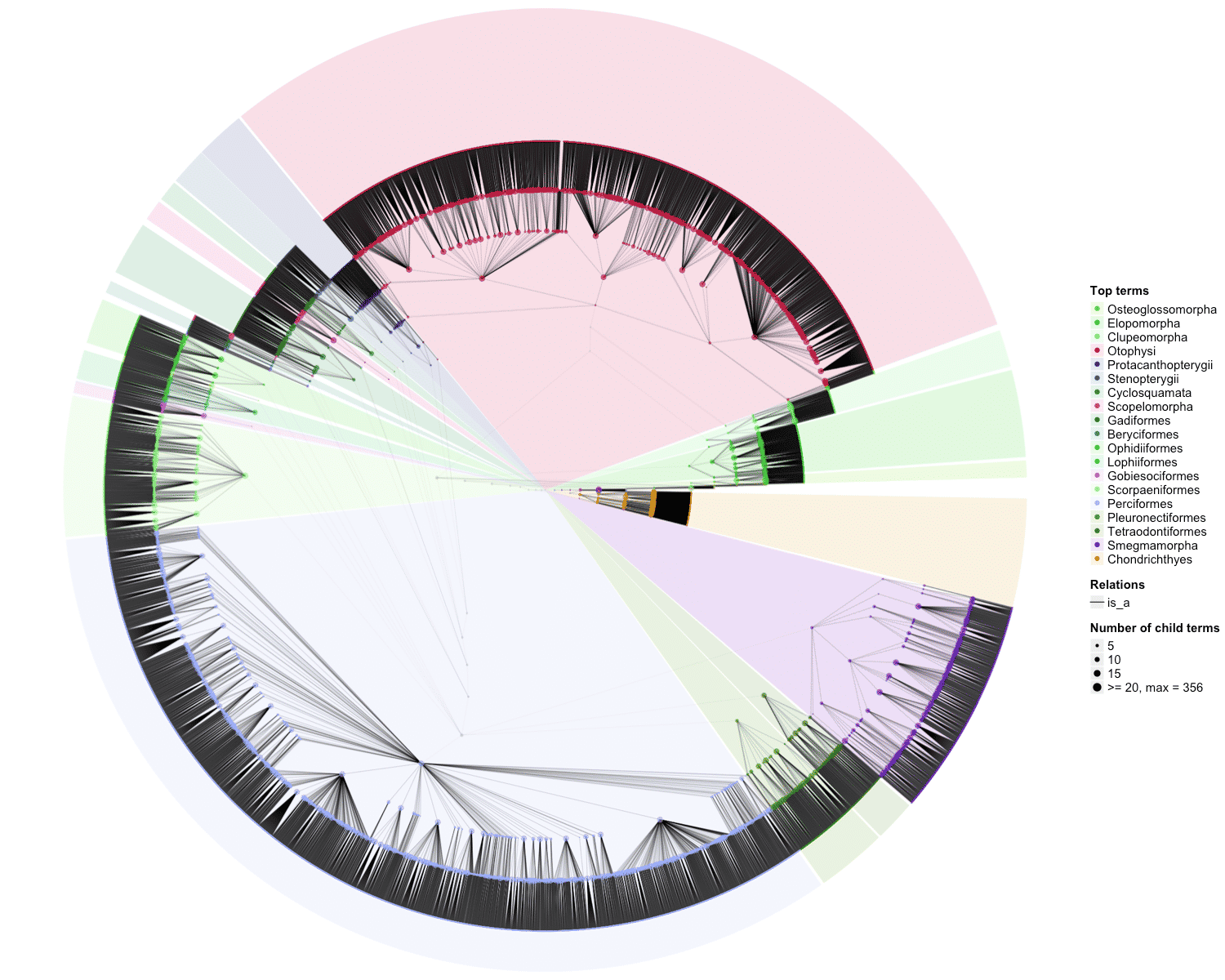

Supplement: Supplementary file 6 — Supplementary Material 6. OBO Foundry gallery [file 12864_2024_10759_MOESM6_ESM.zip › suppl6_OBOFoundry_gallery/image/OBOFoundry_tto.png]

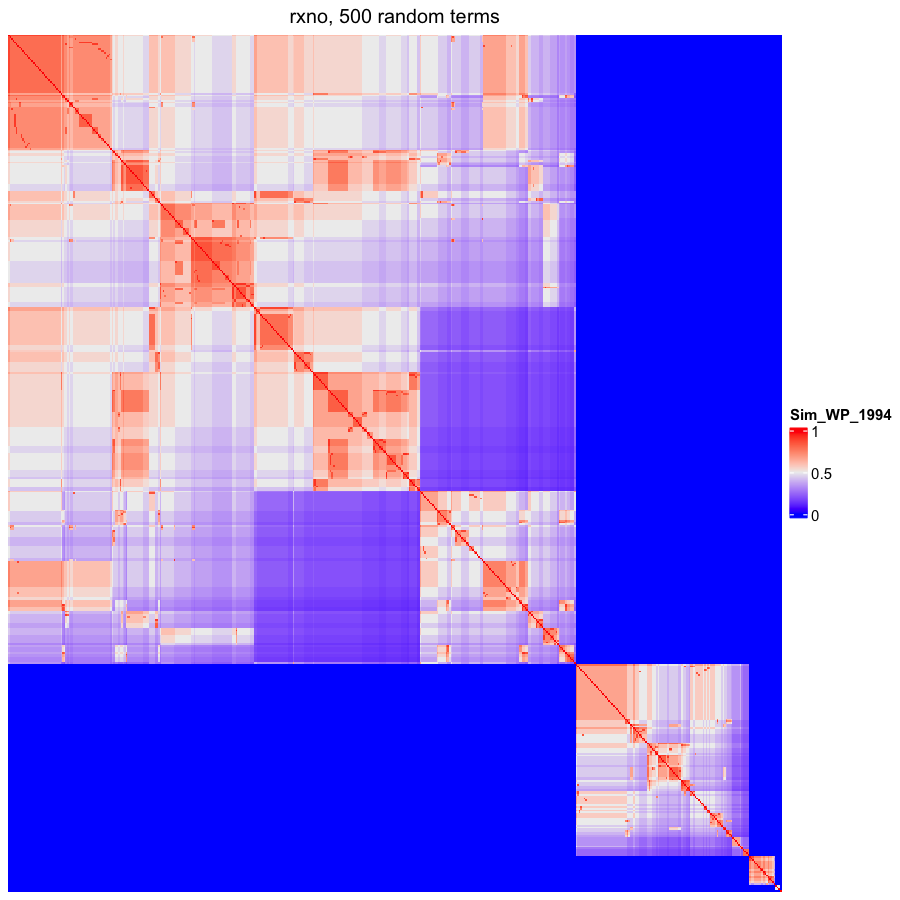

Supplement: Supplementary file 6 — Supplementary Material 6. OBO Foundry gallery [file 12864_2024_10759_MOESM6_ESM.zip › suppl6_OBOFoundry_gallery/image/OBOFoundry_rxno_heatmap.png]

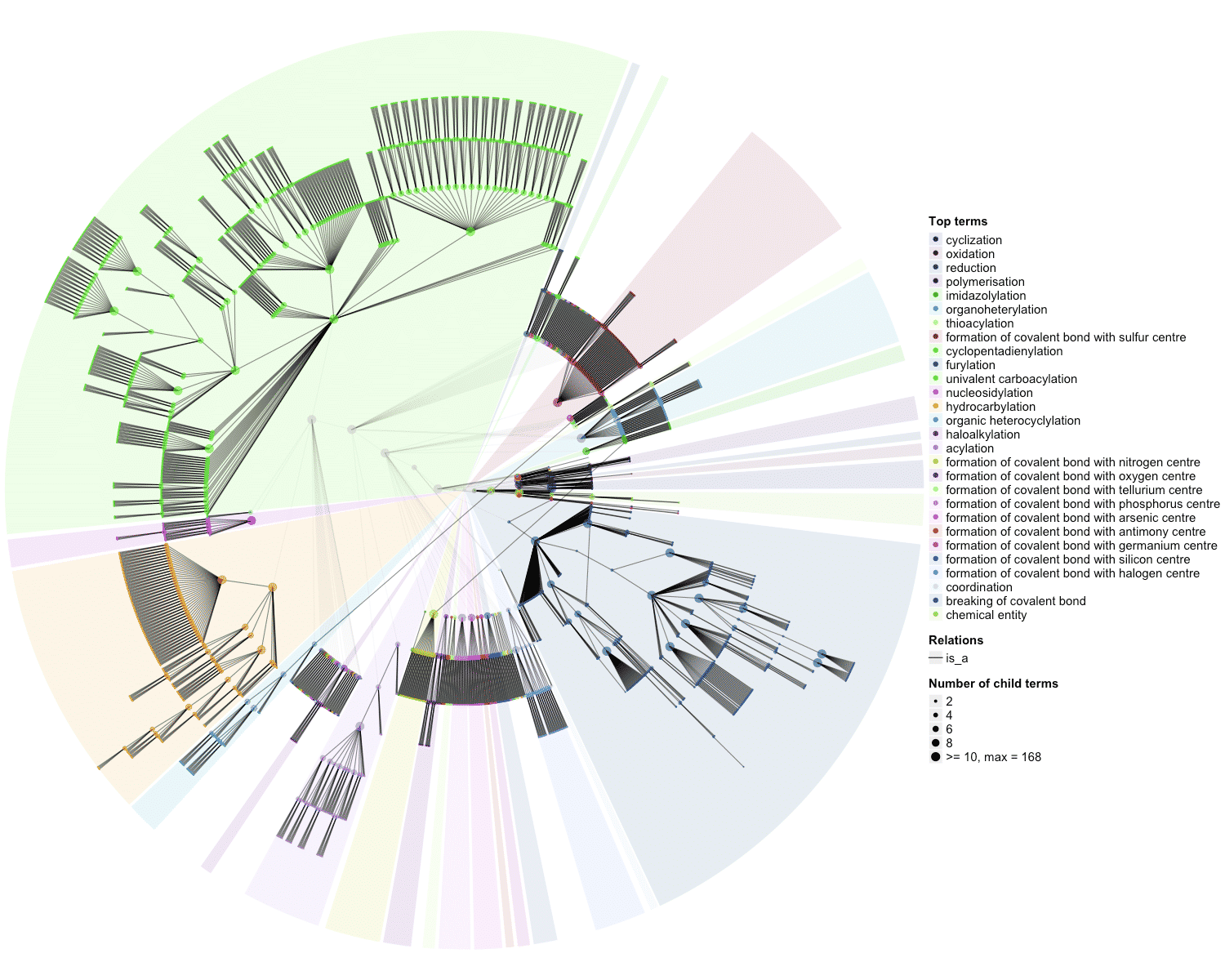

Supplement: Supplementary file 6 — Supplementary Material 6. OBO Foundry gallery [file 12864_2024_10759_MOESM6_ESM.zip › suppl6_OBOFoundry_gallery/image/OBOFoundry_mop.png]

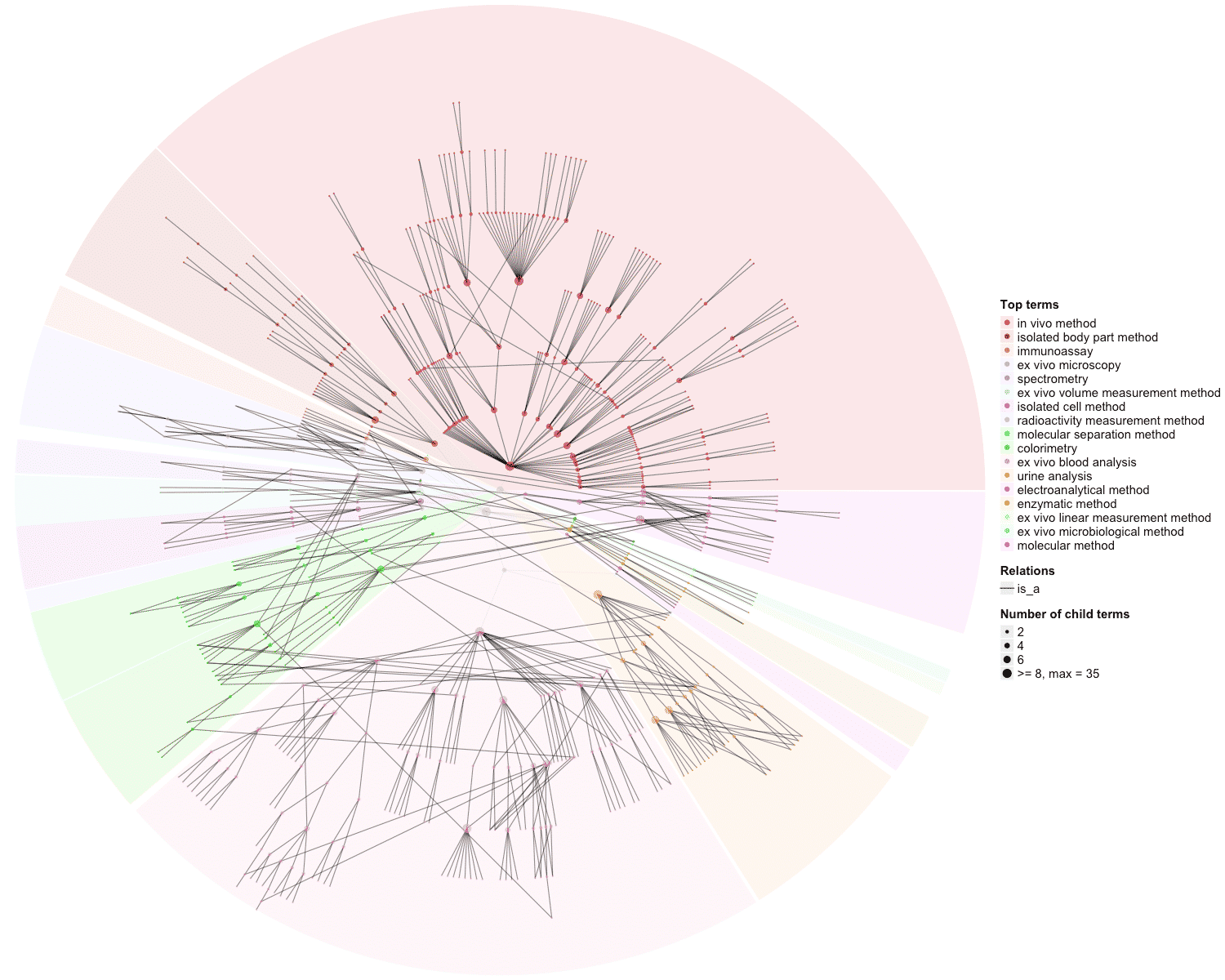

Supplement: Supplementary file 6 — Supplementary Material 6. OBO Foundry gallery [file 12864_2024_10759_MOESM6_ESM.zip › suppl6_OBOFoundry_gallery/image/OBOFoundry_mmo.png]

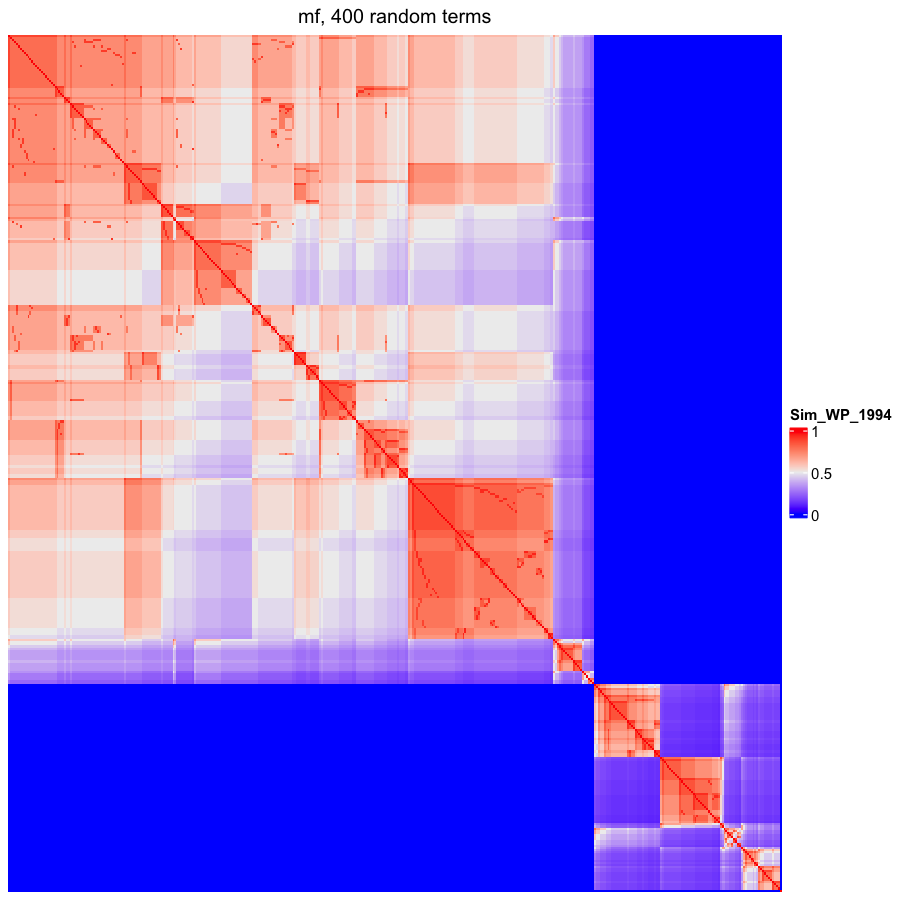

Supplement: Supplementary file 6 — Supplementary Material 6. OBO Foundry gallery [file 12864_2024_10759_MOESM6_ESM.zip › suppl6_OBOFoundry_gallery/image/OBOFoundry_mf_heatmap.png]

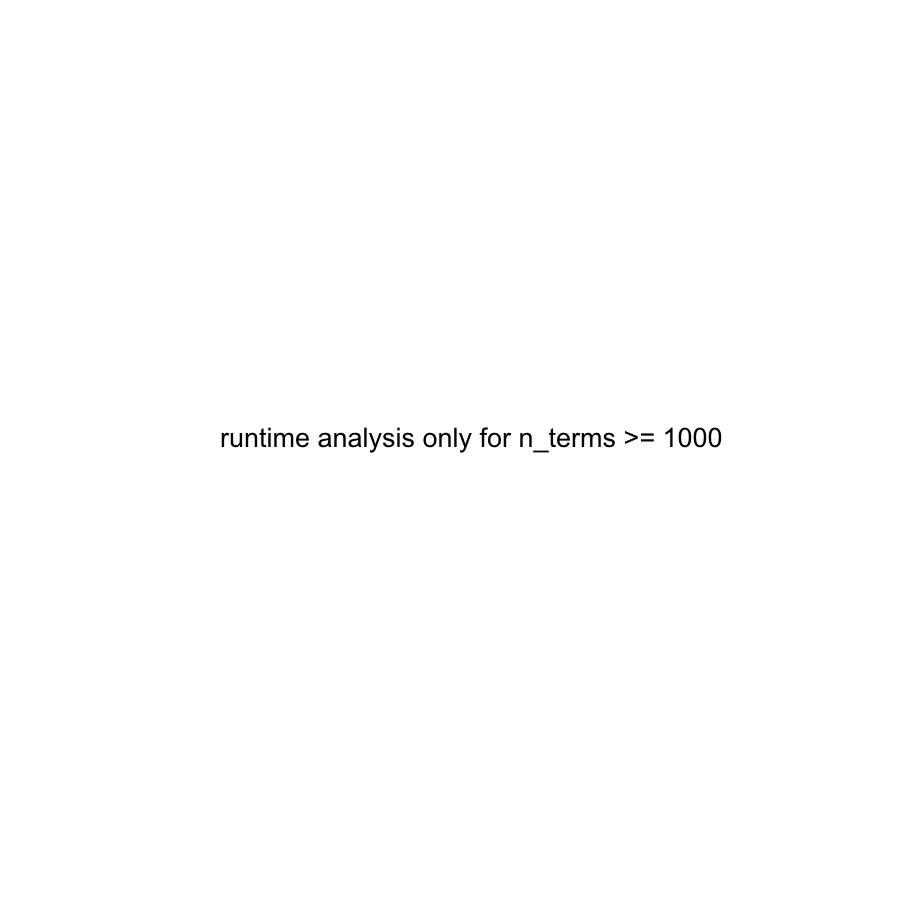

Supplement: Supplementary file 6 — Supplementary Material 6. OBO Foundry gallery [file 12864_2024_10759_MOESM6_ESM.zip › suppl6_OBOFoundry_gallery/image/OBOFoundry_flopo_runtime.png]

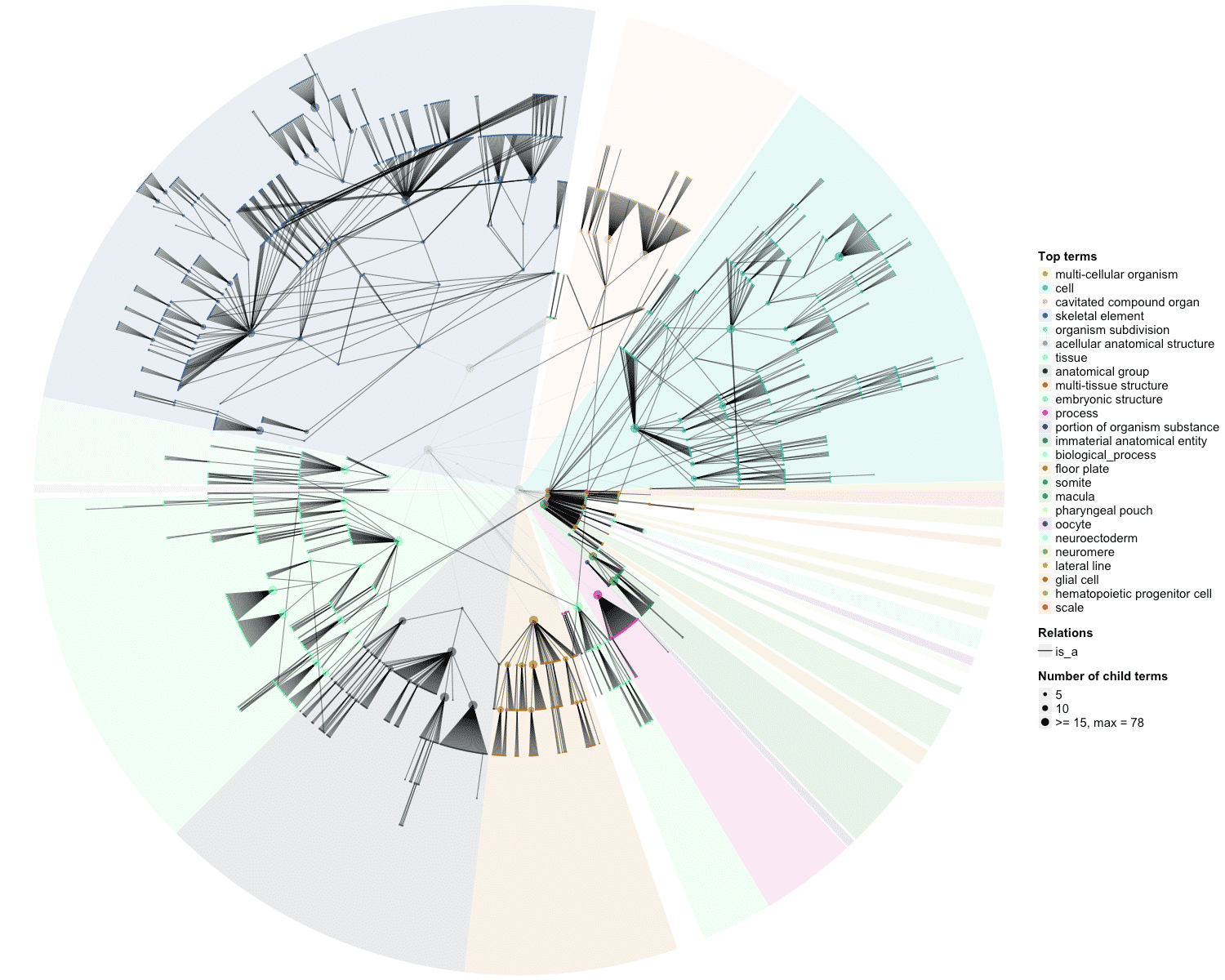

Supplement: Supplementary file 6 — Supplementary Material 6. OBO Foundry gallery [file 12864_2024_10759_MOESM6_ESM.zip › suppl6_OBOFoundry_gallery/image/OBOFoundry_tao.png]

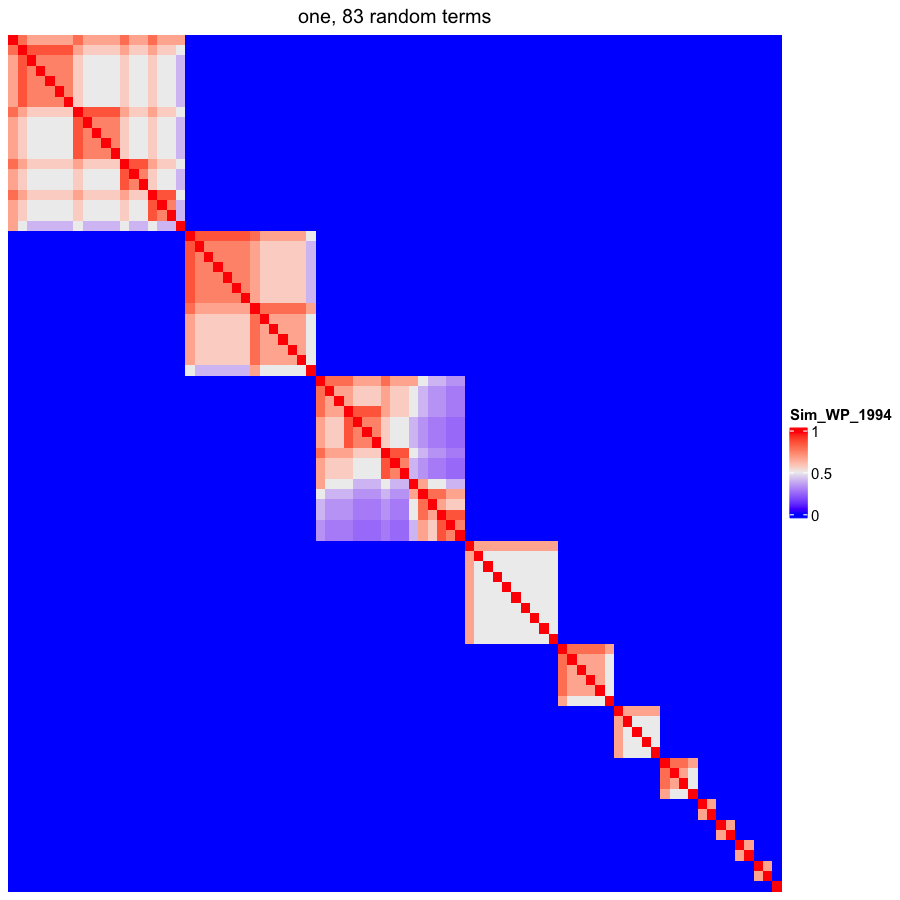

Supplement: Supplementary file 6 — Supplementary Material 6. OBO Foundry gallery [file 12864_2024_10759_MOESM6_ESM.zip › suppl6_OBOFoundry_gallery/image/OBOFoundry_one_heatmap.png]

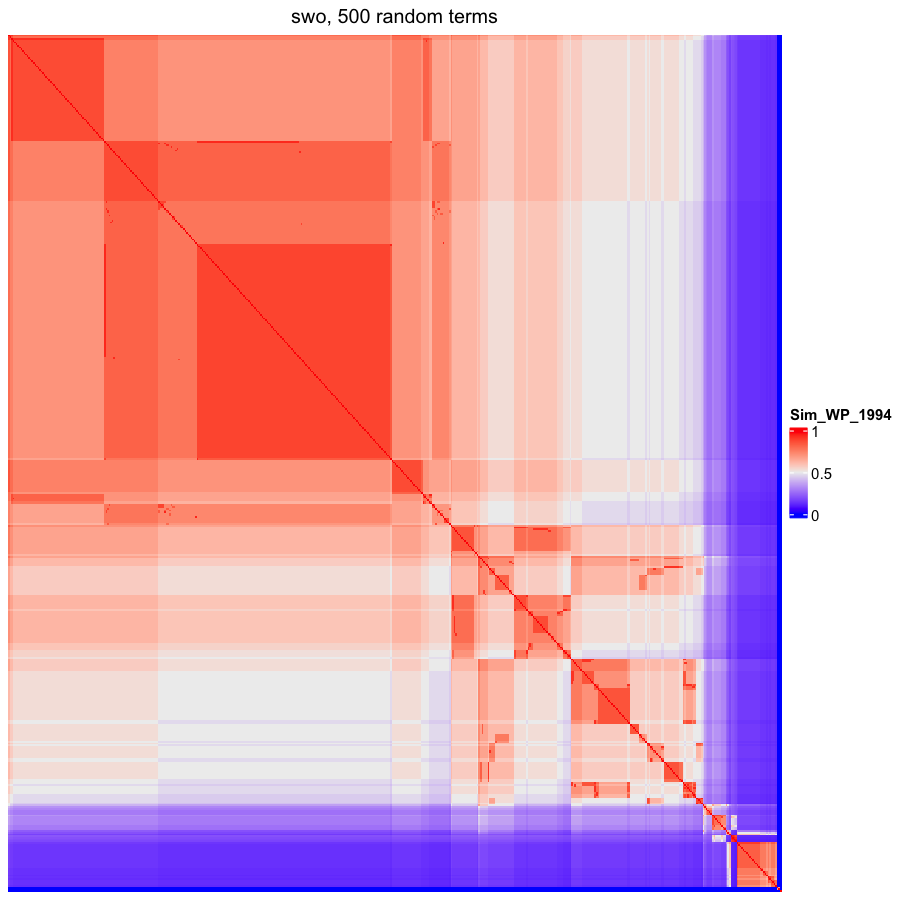

Supplement: Supplementary file 6 — Supplementary Material 6. OBO Foundry gallery [file 12864_2024_10759_MOESM6_ESM.zip › suppl6_OBOFoundry_gallery/image/OBOFoundry_swo_heatmap.png]

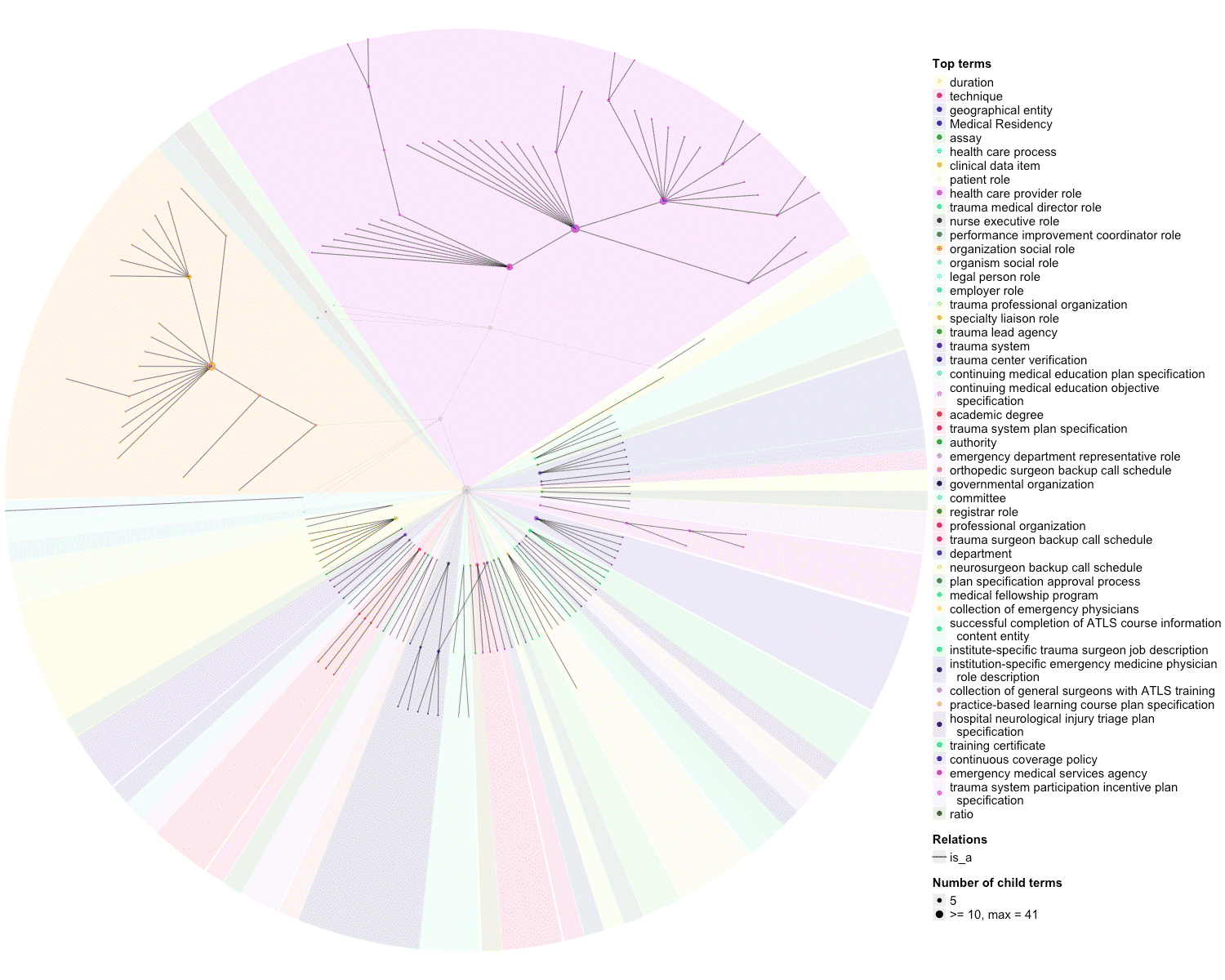

Supplement: Supplementary file 6 — Supplementary Material 6. OBO Foundry gallery [file 12864_2024_10759_MOESM6_ESM.zip › suppl6_OBOFoundry_gallery/image/OBOFoundry_oostt.png]
