# Supplementary figures and images for: simona: a comprehensive R package for semantic similarity analysis on bio-ontologies (part 3 of 3)
Source: BMC Genomics. 2024 Sep 16;25:869. doi: 10.1186/s12864-024-10759-4 (PMC11406866; doi:10.1186/s12864-024-10759-4)

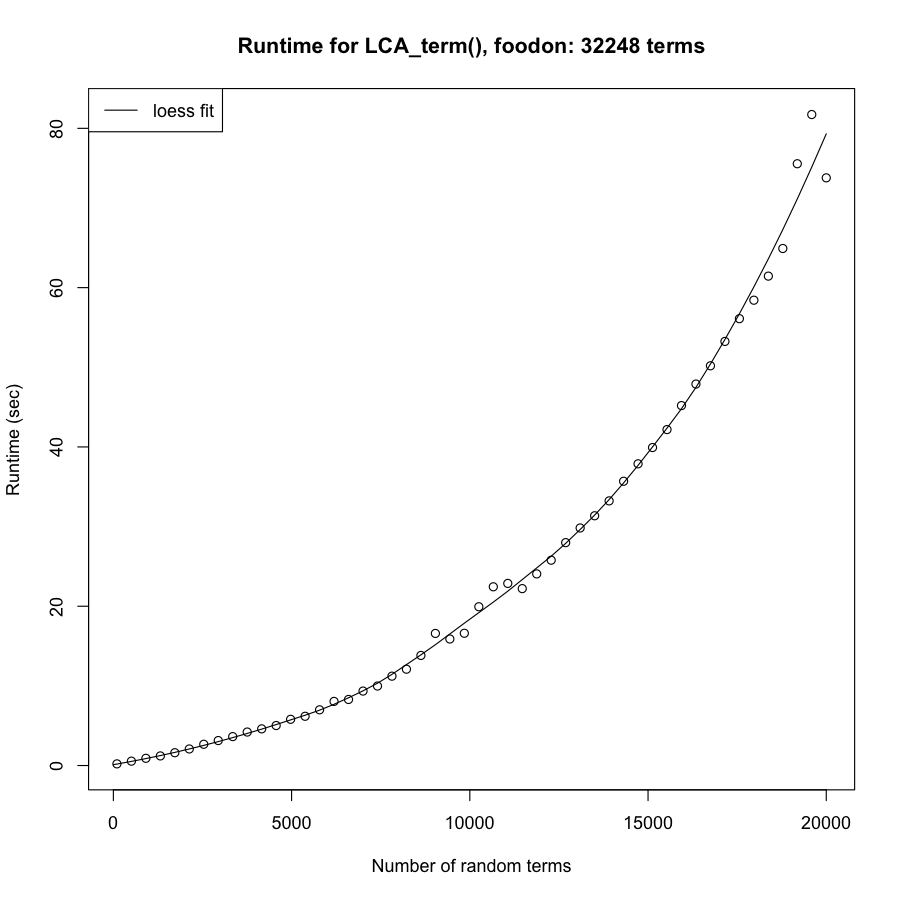

Supplement: Supplementary file 6 — Supplementary Material 6. OBO Foundry gallery [file 12864_2024_10759_MOESM6_ESM.zip › suppl6_OBOFoundry_gallery/image/OBOFoundry_foodon_runtime.png]

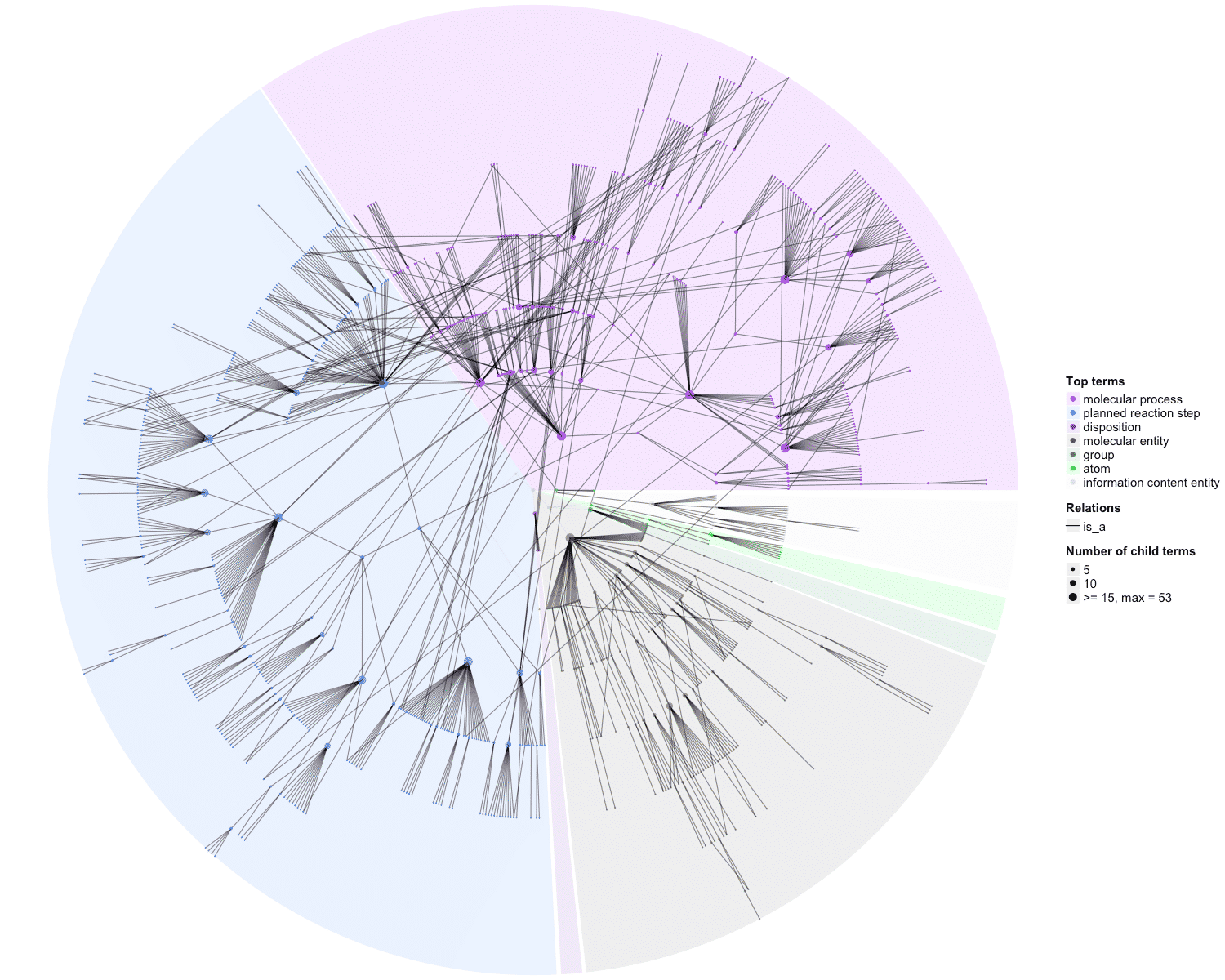

Supplement: Supplementary file 6 — Supplementary Material 6. OBO Foundry gallery [file 12864_2024_10759_MOESM6_ESM.zip › suppl6_OBOFoundry_gallery/image/OBOFoundry_rxno.png]

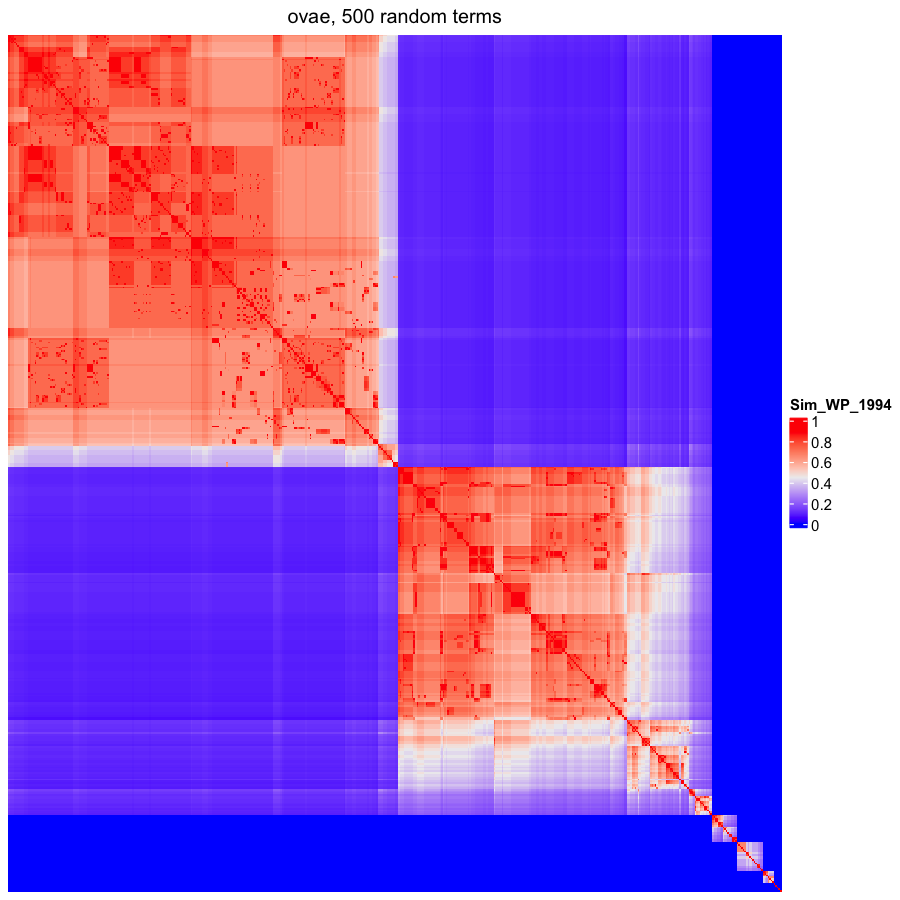

Supplement: Supplementary file 6 — Supplementary Material 6. OBO Foundry gallery [file 12864_2024_10759_MOESM6_ESM.zip › suppl6_OBOFoundry_gallery/image/OBOFoundry_ovae_heatmap.png]

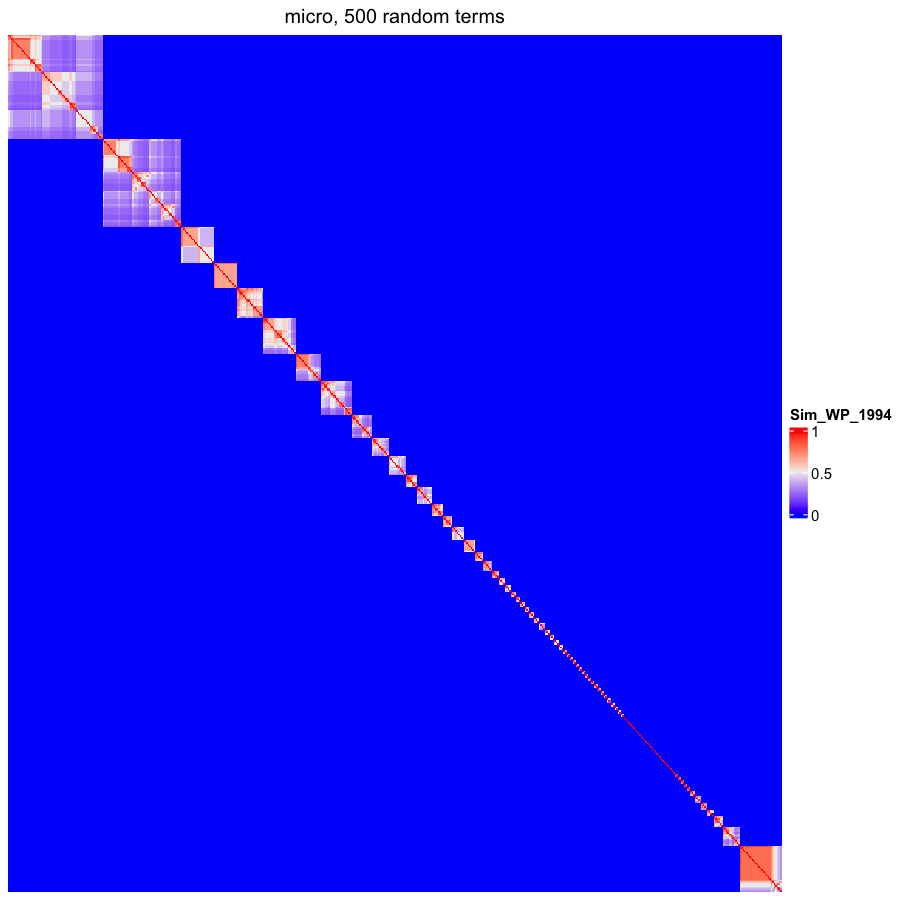

Supplement: Supplementary file 6 — Supplementary Material 6. OBO Foundry gallery [file 12864_2024_10759_MOESM6_ESM.zip › suppl6_OBOFoundry_gallery/image/OBOFoundry_micro_heatmap.png]

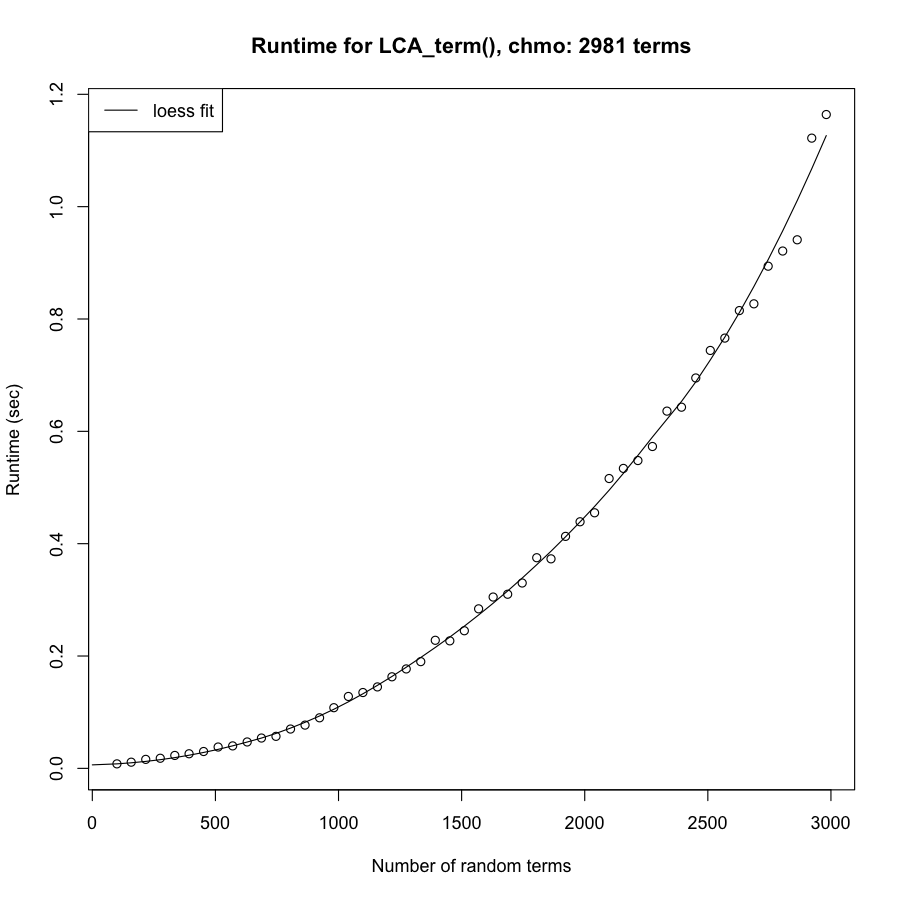

Supplement: Supplementary file 6 — Supplementary Material 6. OBO Foundry gallery [file 12864_2024_10759_MOESM6_ESM.zip › suppl6_OBOFoundry_gallery/image/OBOFoundry_chmo_runtime.png]

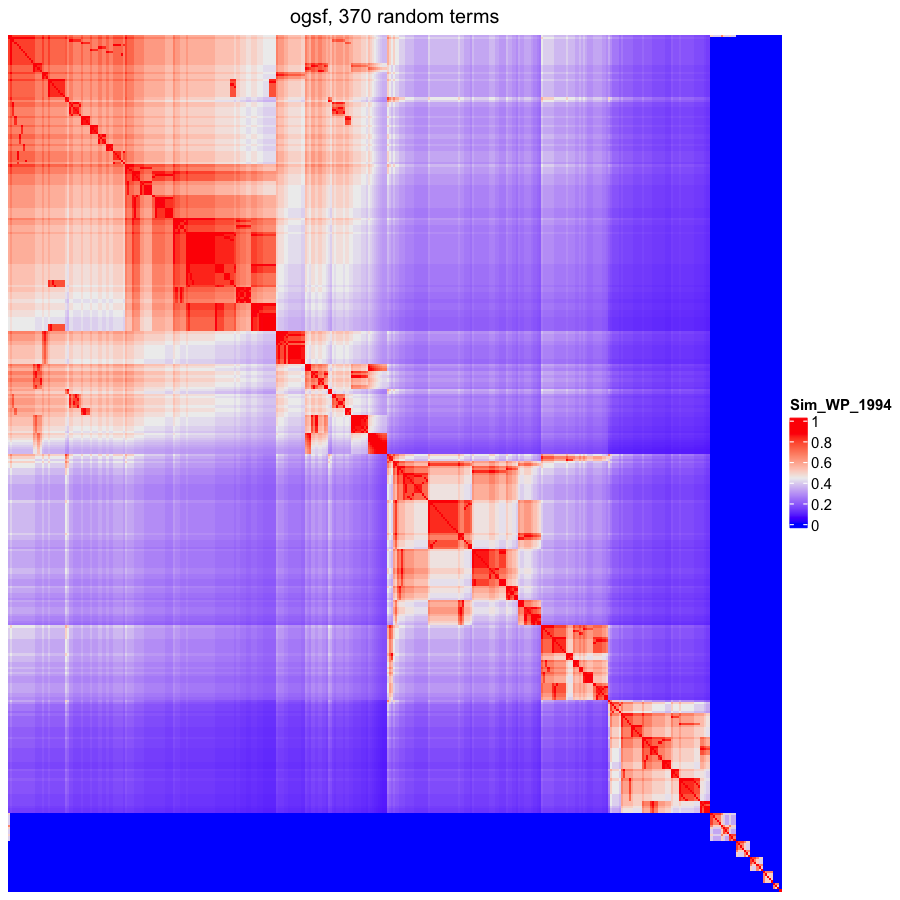

Supplement: Supplementary file 6 — Supplementary Material 6. OBO Foundry gallery [file 12864_2024_10759_MOESM6_ESM.zip › suppl6_OBOFoundry_gallery/image/OBOFoundry_ogsf_heatmap.png]

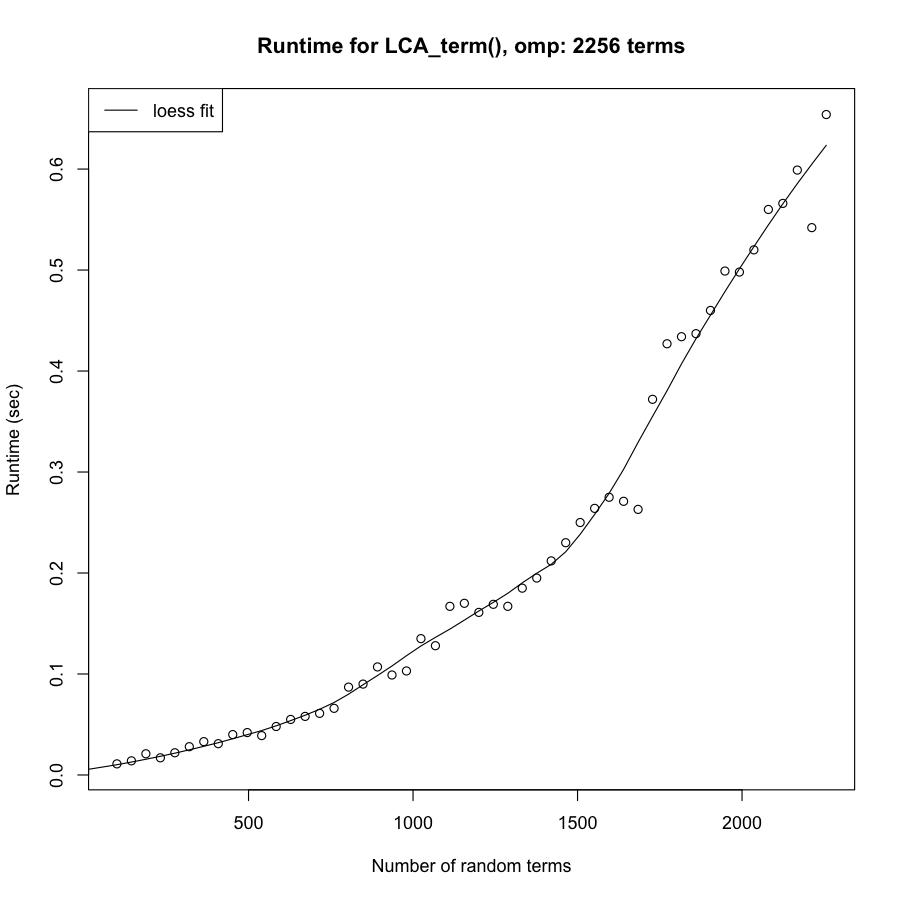

Supplement: Supplementary file 6 — Supplementary Material 6. OBO Foundry gallery [file 12864_2024_10759_MOESM6_ESM.zip › suppl6_OBOFoundry_gallery/image/OBOFoundry_omp_runtime.png]

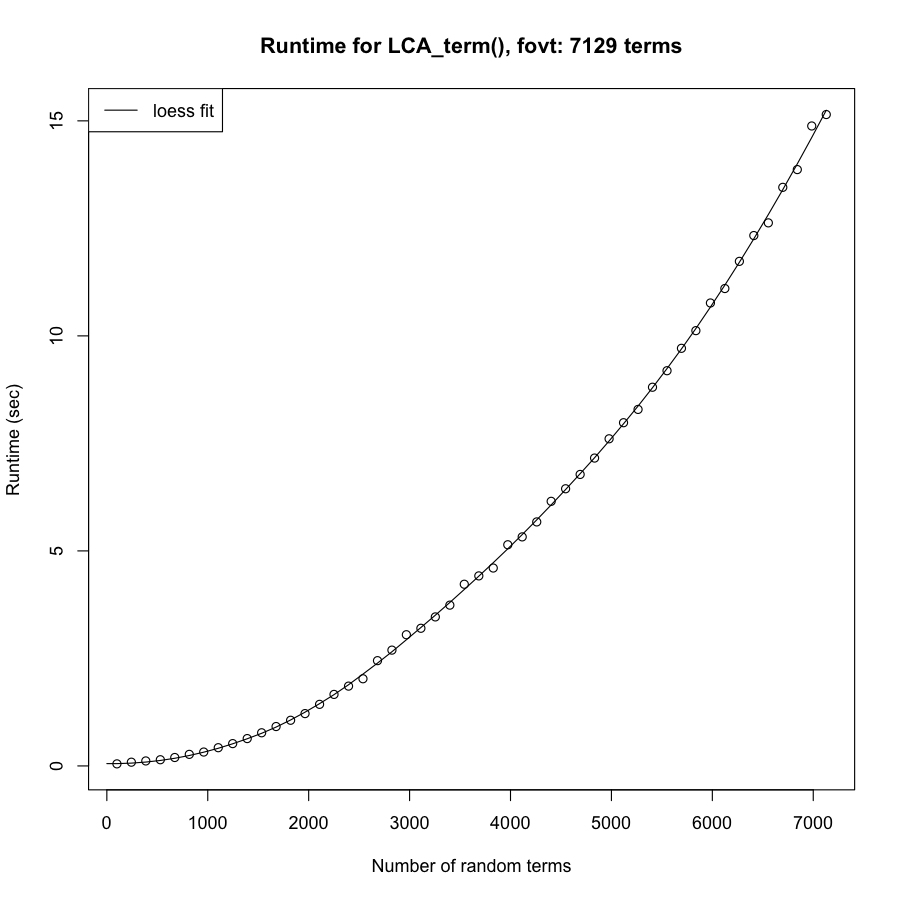

Supplement: Supplementary file 6 — Supplementary Material 6. OBO Foundry gallery [file 12864_2024_10759_MOESM6_ESM.zip › suppl6_OBOFoundry_gallery/image/OBOFoundry_fovt_runtime.png]

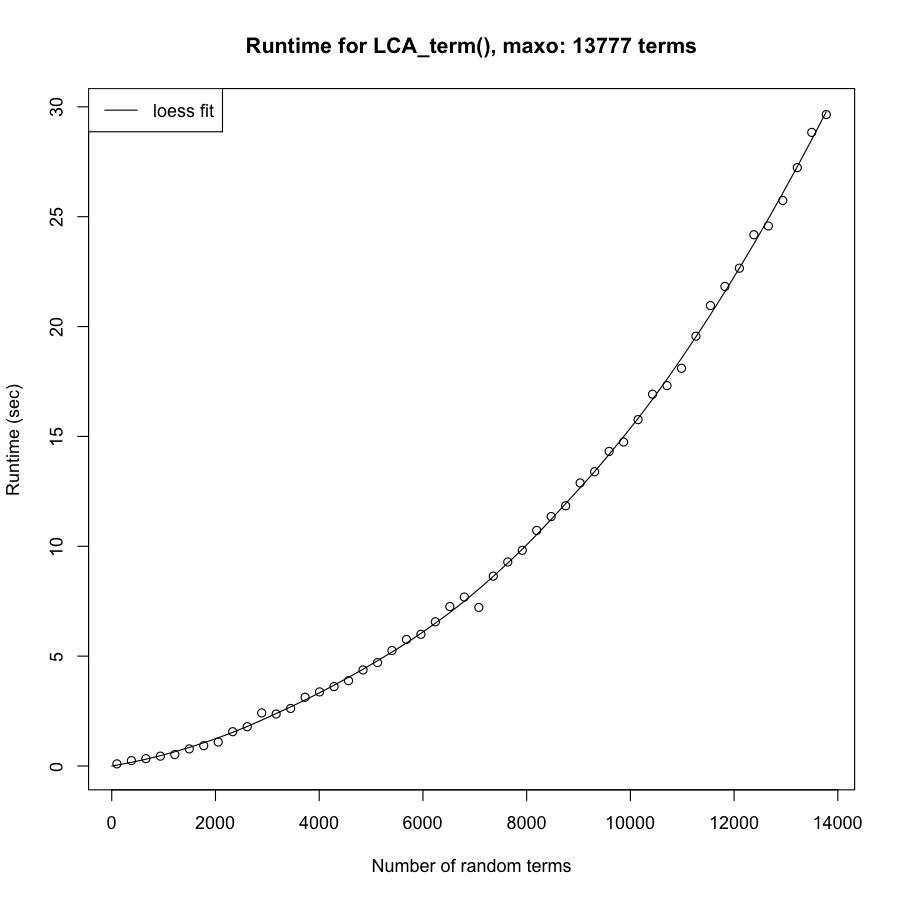

Supplement: Supplementary file 6 — Supplementary Material 6. OBO Foundry gallery [file 12864_2024_10759_MOESM6_ESM.zip › suppl6_OBOFoundry_gallery/image/OBOFoundry_maxo_runtime.png]

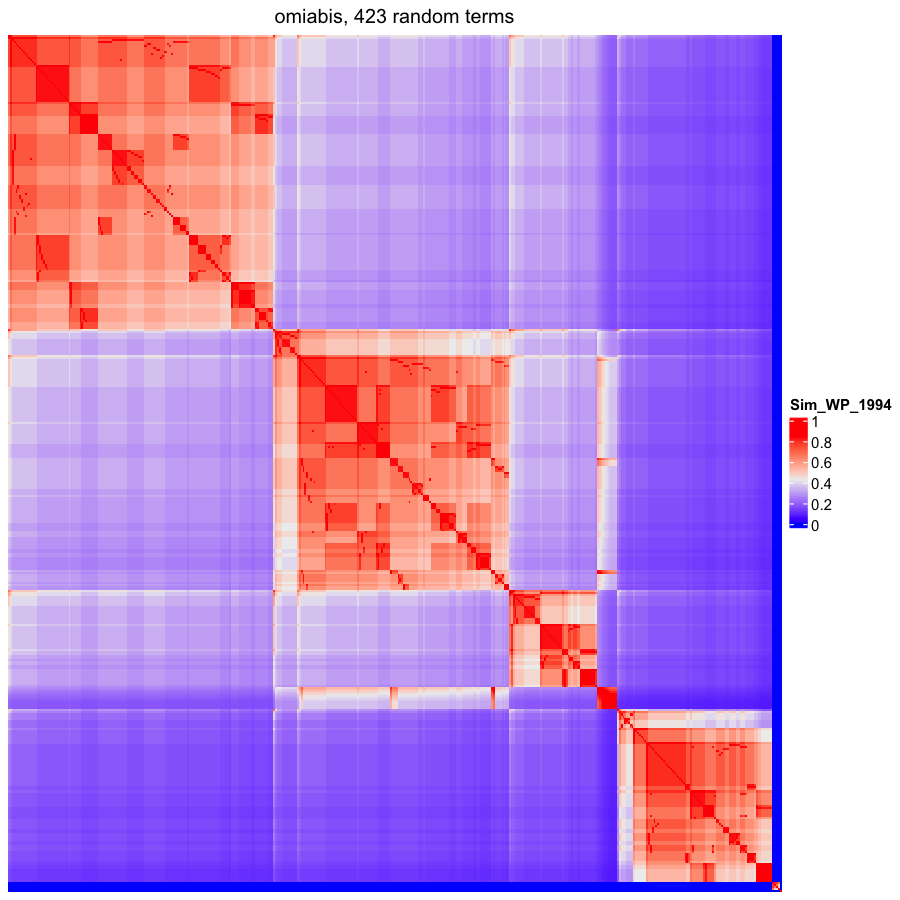

Supplement: Supplementary file 6 — Supplementary Material 6. OBO Foundry gallery [file 12864_2024_10759_MOESM6_ESM.zip › suppl6_OBOFoundry_gallery/image/OBOFoundry_omiabis_heatmap.png]

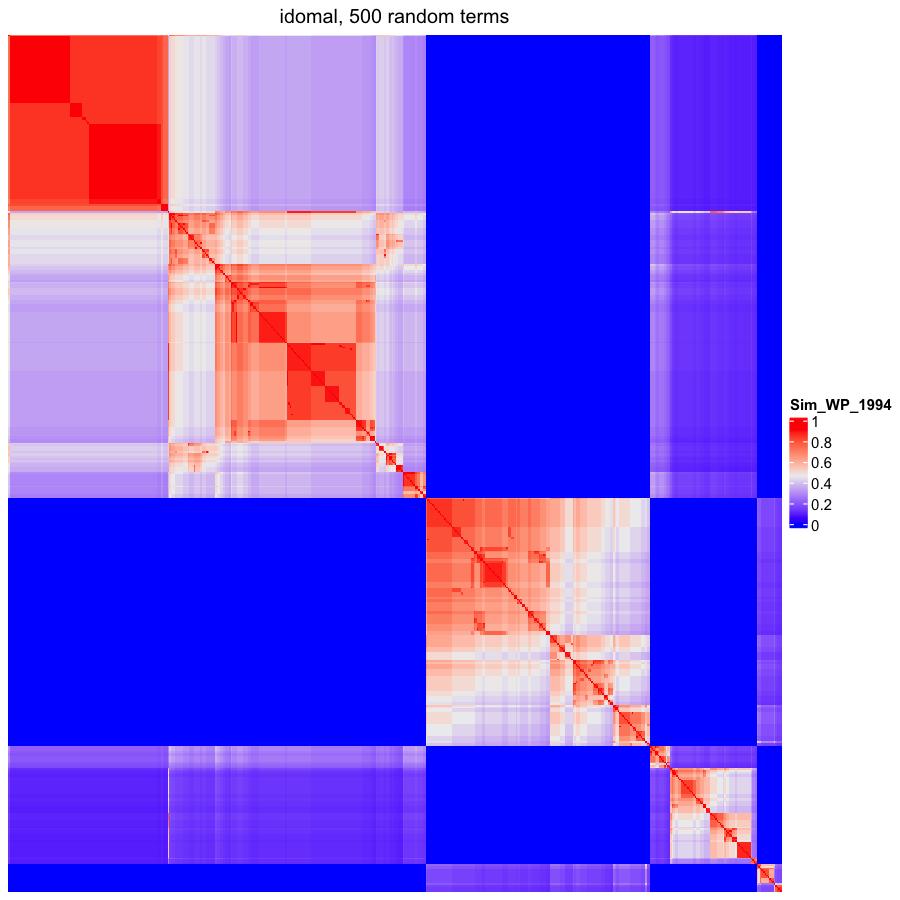

Supplement: Supplementary file 6 — Supplementary Material 6. OBO Foundry gallery [file 12864_2024_10759_MOESM6_ESM.zip › suppl6_OBOFoundry_gallery/image/OBOFoundry_idomal_heatmap.png]

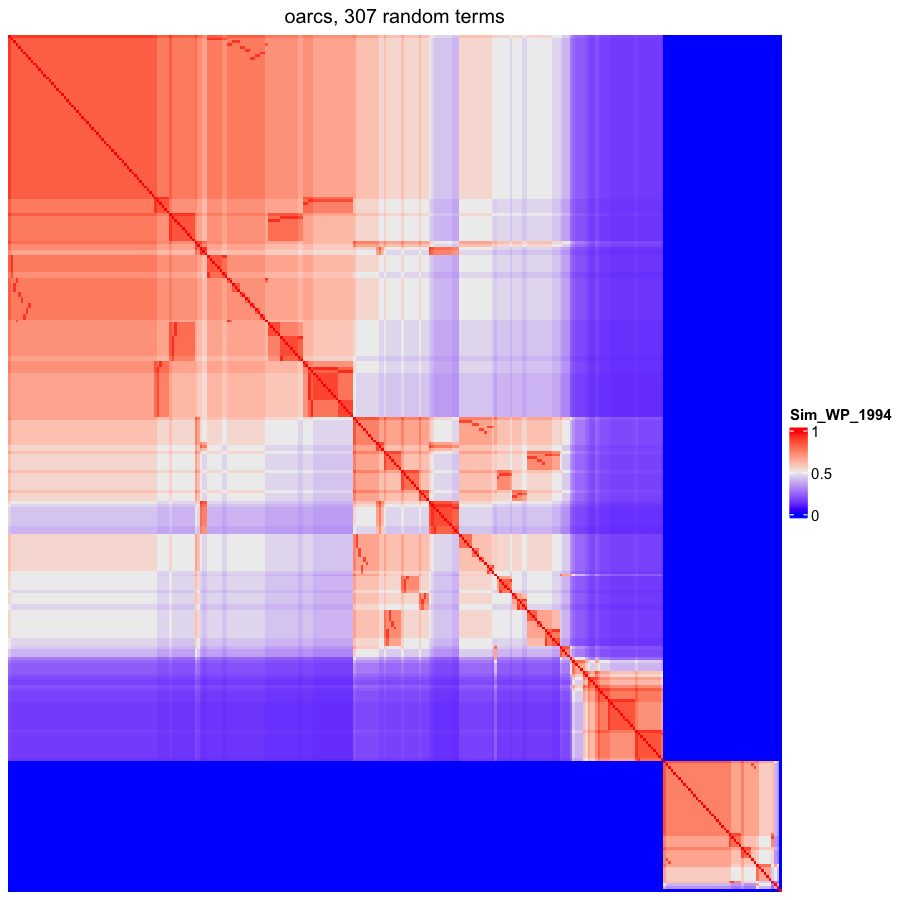

Supplement: Supplementary file 6 — Supplementary Material 6. OBO Foundry gallery [file 12864_2024_10759_MOESM6_ESM.zip › suppl6_OBOFoundry_gallery/image/OBOFoundry_oarcs_heatmap.png]

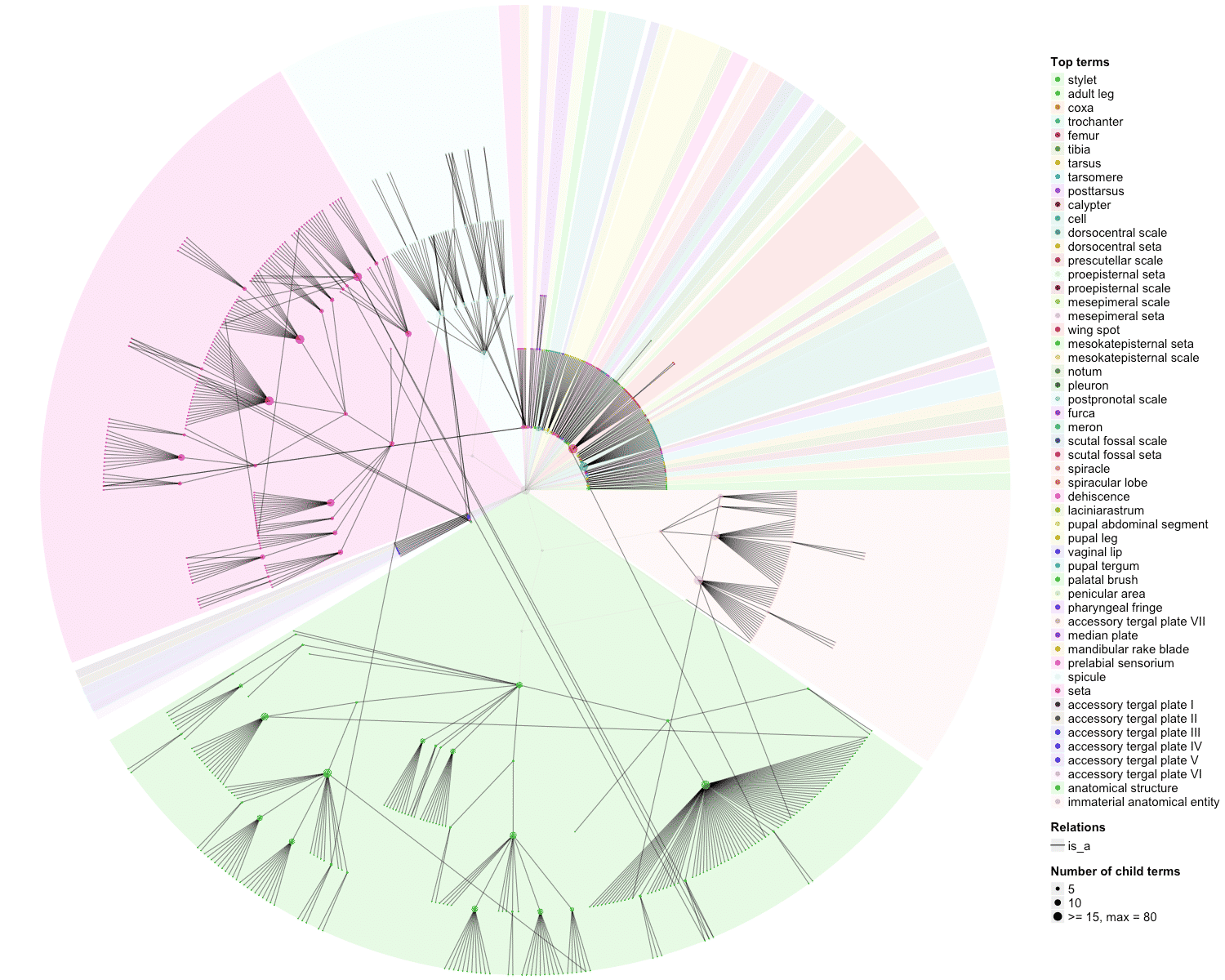

Supplement: Supplementary file 6 — Supplementary Material 6. OBO Foundry gallery [file 12864_2024_10759_MOESM6_ESM.zip › suppl6_OBOFoundry_gallery/image/OBOFoundry_tgma.png]

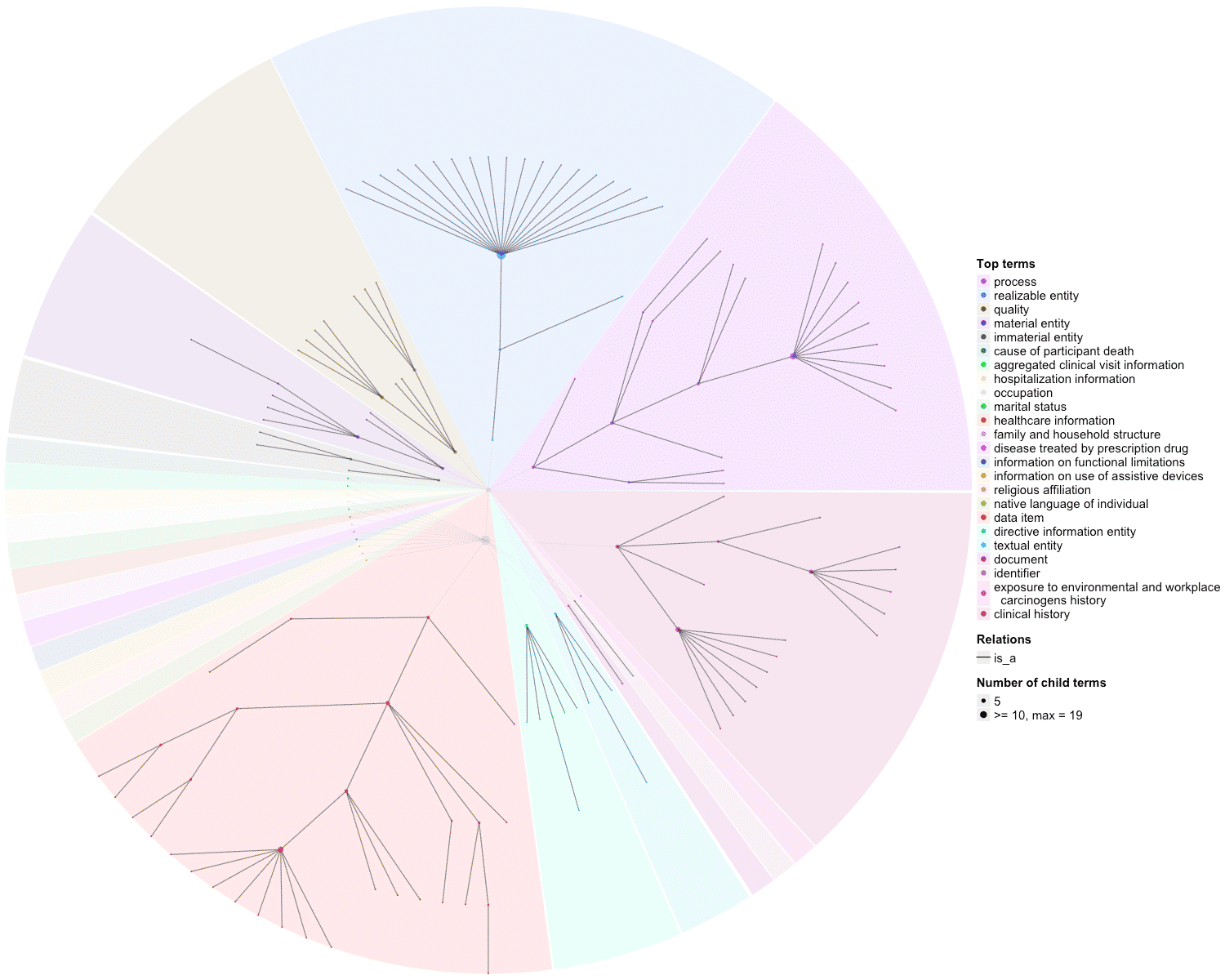

Supplement: Supplementary file 6 — Supplementary Material 6. OBO Foundry gallery [file 12864_2024_10759_MOESM6_ESM.zip › suppl6_OBOFoundry_gallery/image/OBOFoundry_gecko.png]

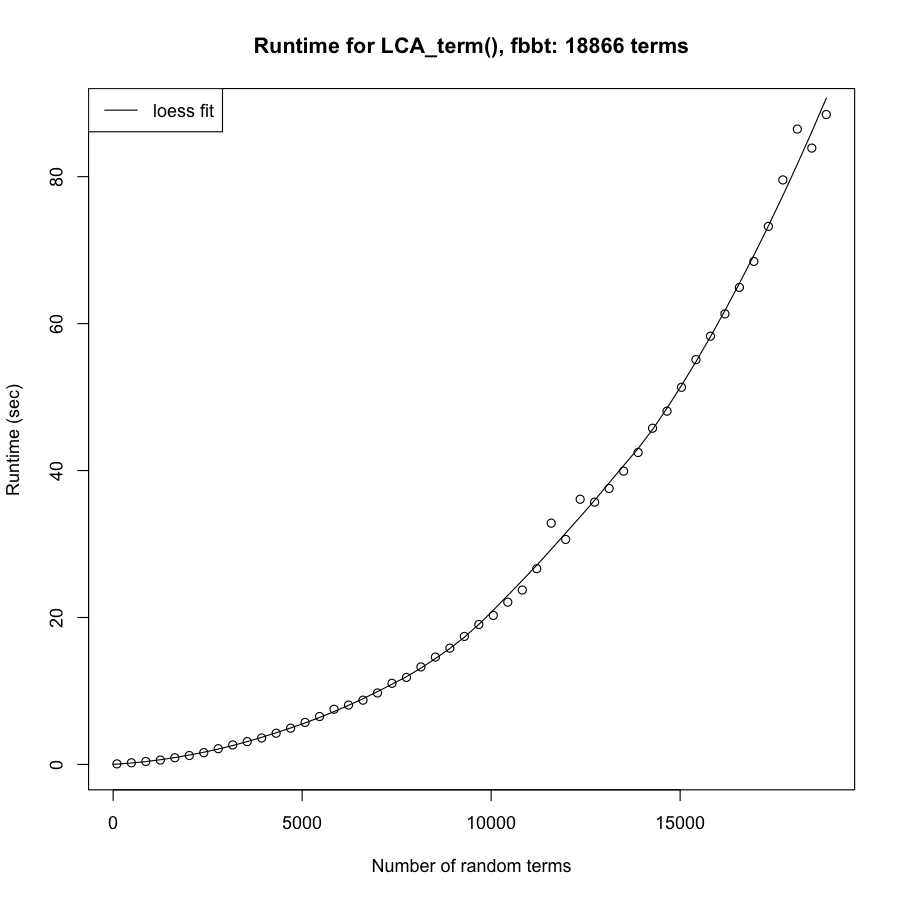

Supplement: Supplementary file 6 — Supplementary Material 6. OBO Foundry gallery [file 12864_2024_10759_MOESM6_ESM.zip › suppl6_OBOFoundry_gallery/image/OBOFoundry_fbbt_runtime.png]

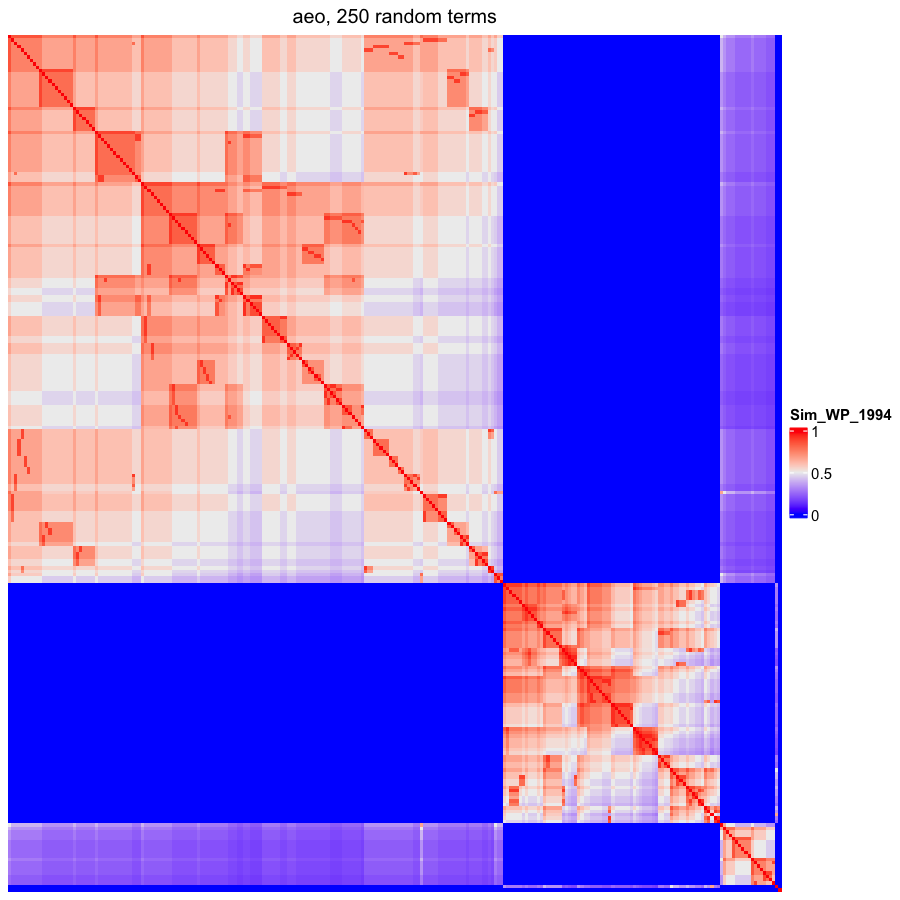

Supplement: Supplementary file 6 — Supplementary Material 6. OBO Foundry gallery [file 12864_2024_10759_MOESM6_ESM.zip › suppl6_OBOFoundry_gallery/image/OBOFoundry_aeo_heatmap.png]

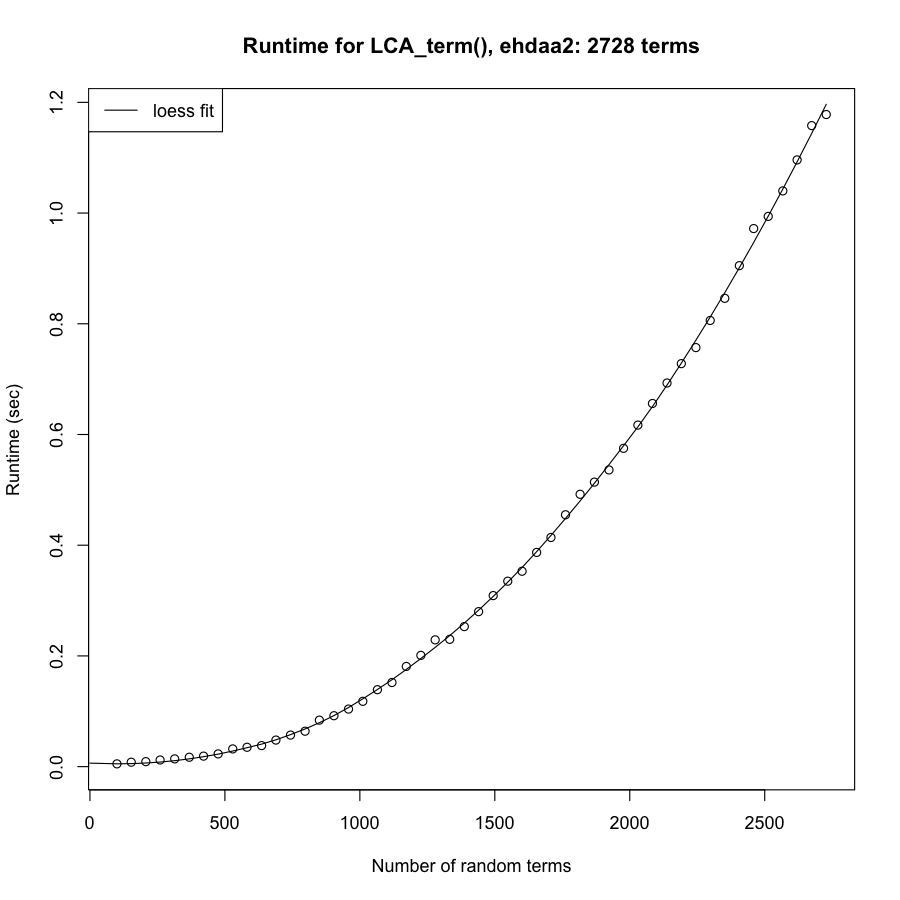

Supplement: Supplementary file 6 — Supplementary Material 6. OBO Foundry gallery [file 12864_2024_10759_MOESM6_ESM.zip › suppl6_OBOFoundry_gallery/image/OBOFoundry_ehdaa2_runtime.png]

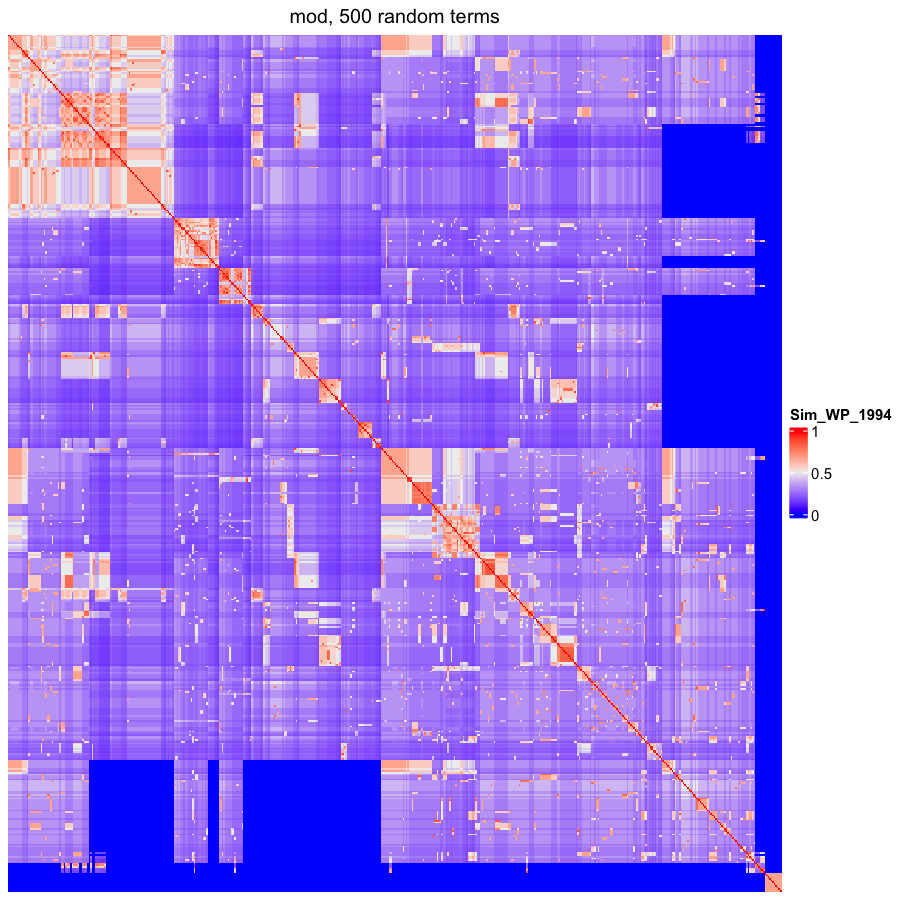

Supplement: Supplementary file 6 — Supplementary Material 6. OBO Foundry gallery [file 12864_2024_10759_MOESM6_ESM.zip › suppl6_OBOFoundry_gallery/image/OBOFoundry_mod_heatmap.png]

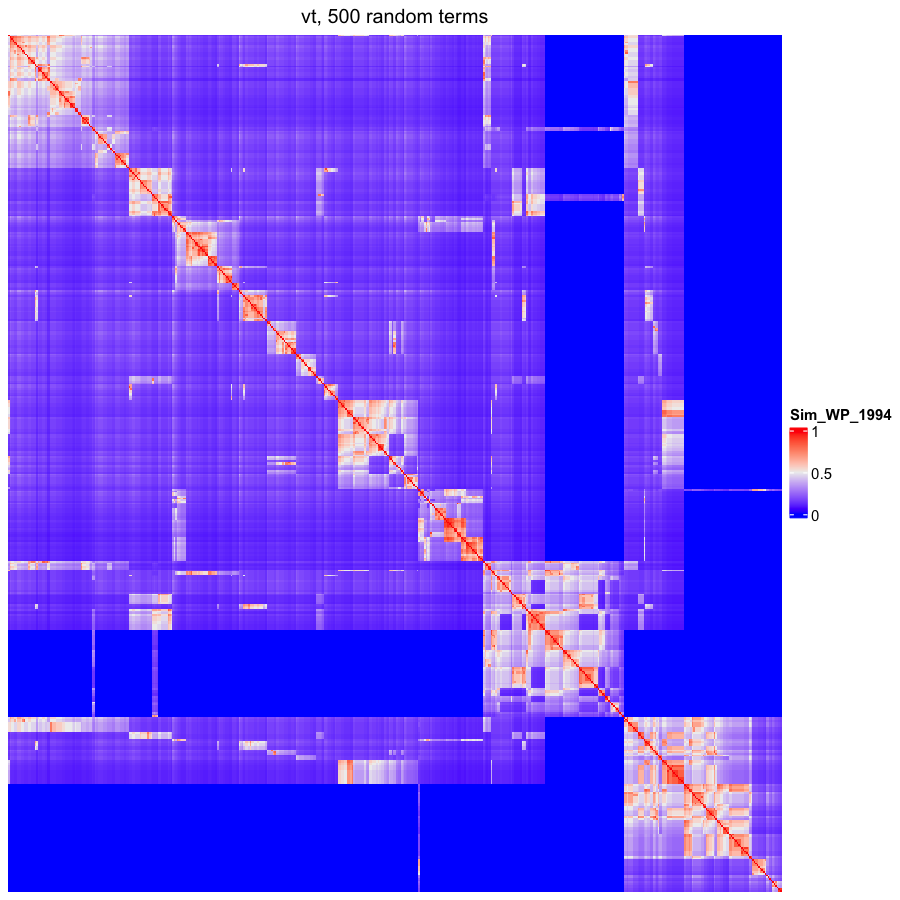

Supplement: Supplementary file 6 — Supplementary Material 6. OBO Foundry gallery [file 12864_2024_10759_MOESM6_ESM.zip › suppl6_OBOFoundry_gallery/image/OBOFoundry_vt_heatmap.png]

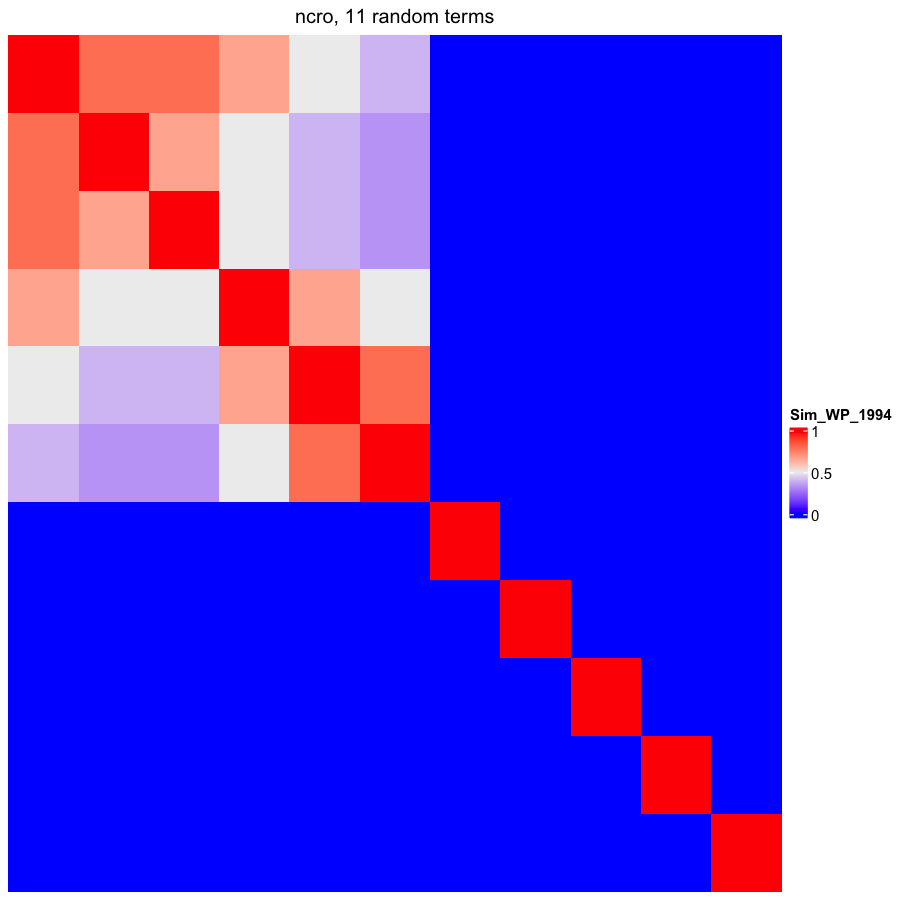

Supplement: Supplementary file 6 — Supplementary Material 6. OBO Foundry gallery [file 12864_2024_10759_MOESM6_ESM.zip › suppl6_OBOFoundry_gallery/image/OBOFoundry_ncro_heatmap.png]

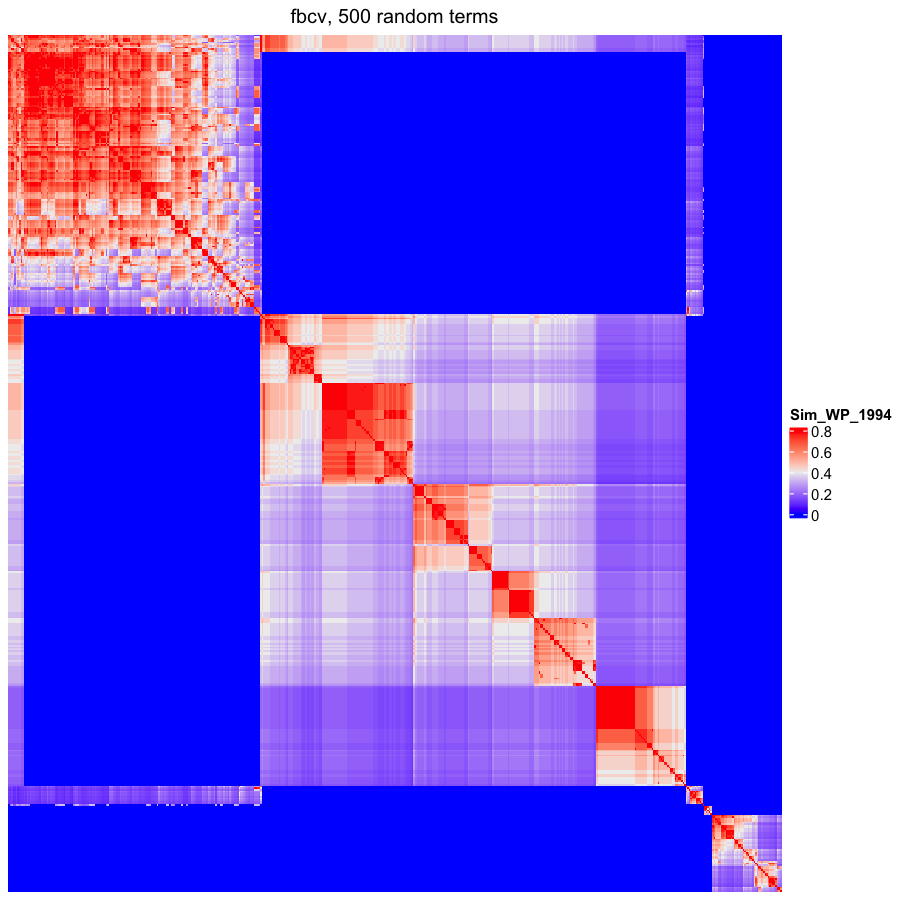

Supplement: Supplementary file 6 — Supplementary Material 6. OBO Foundry gallery [file 12864_2024_10759_MOESM6_ESM.zip › suppl6_OBOFoundry_gallery/image/OBOFoundry_fbcv_heatmap.png]

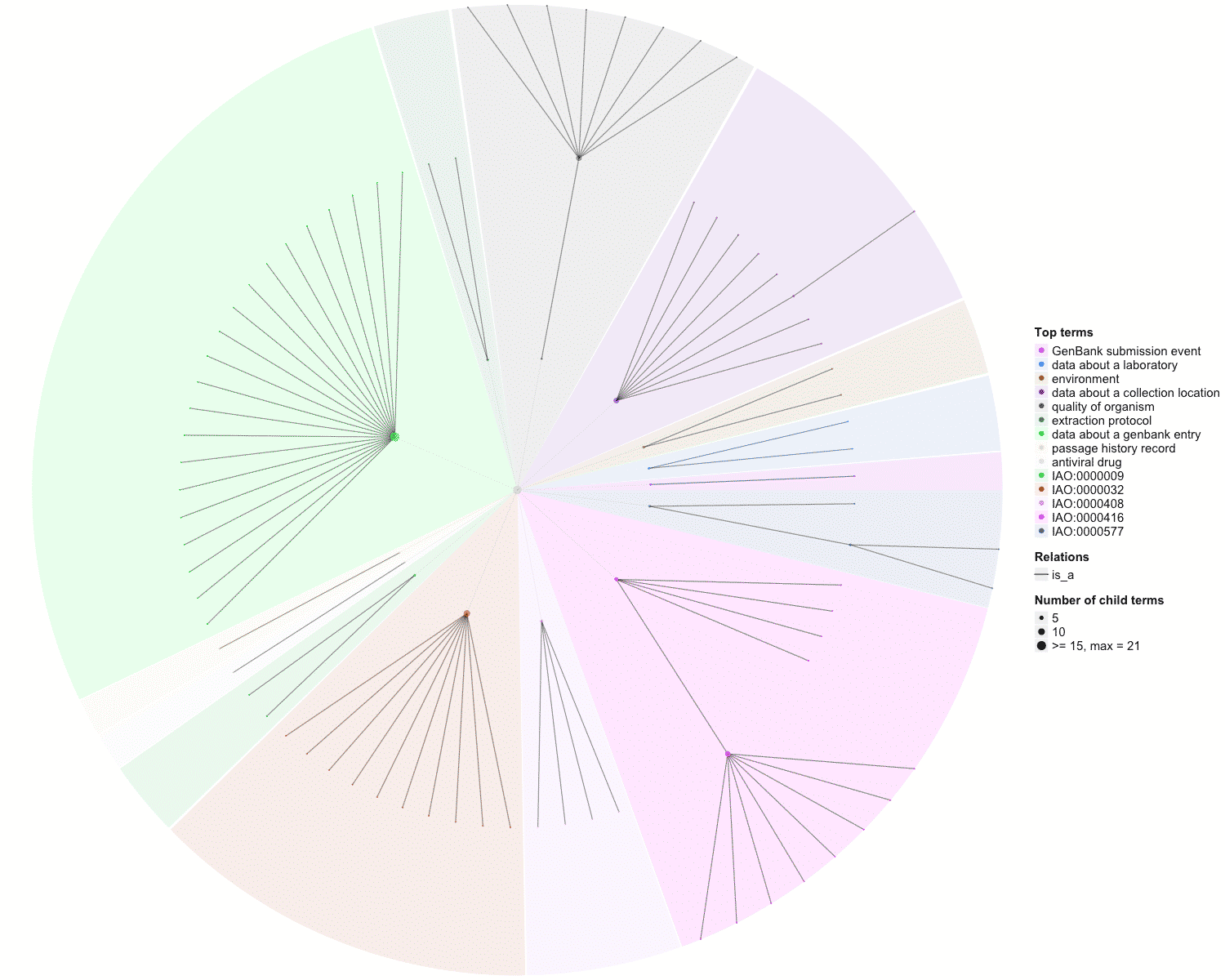

Supplement: Supplementary file 6 — Supplementary Material 6. OBO Foundry gallery [file 12864_2024_10759_MOESM6_ESM.zip › suppl6_OBOFoundry_gallery/image/OBOFoundry_flu.png]

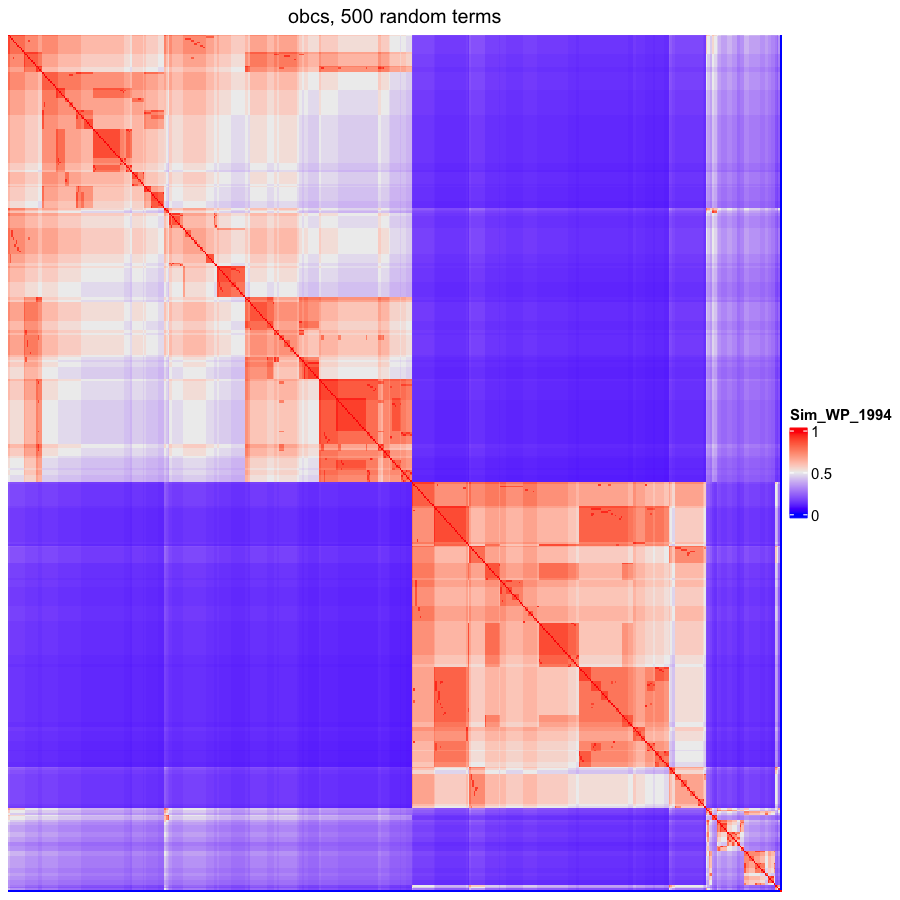

Supplement: Supplementary file 6 — Supplementary Material 6. OBO Foundry gallery [file 12864_2024_10759_MOESM6_ESM.zip › suppl6_OBOFoundry_gallery/image/OBOFoundry_obcs_heatmap.png]

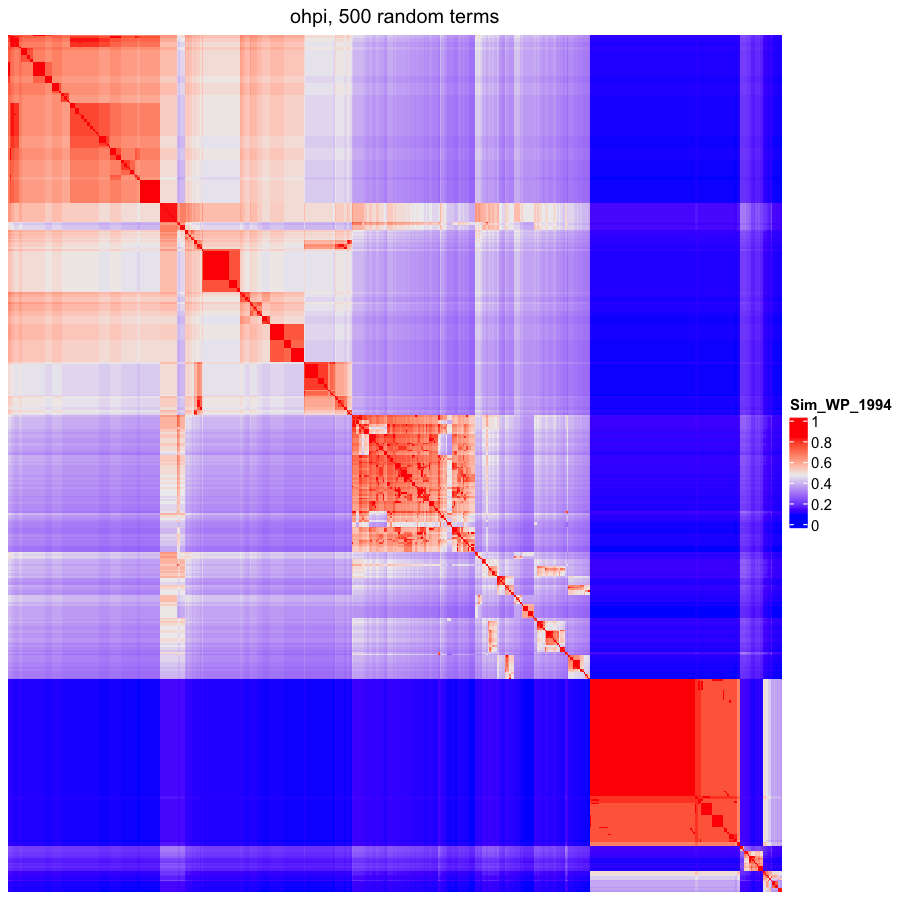

Supplement: Supplementary file 6 — Supplementary Material 6. OBO Foundry gallery [file 12864_2024_10759_MOESM6_ESM.zip › suppl6_OBOFoundry_gallery/image/OBOFoundry_ohpi_heatmap.png]

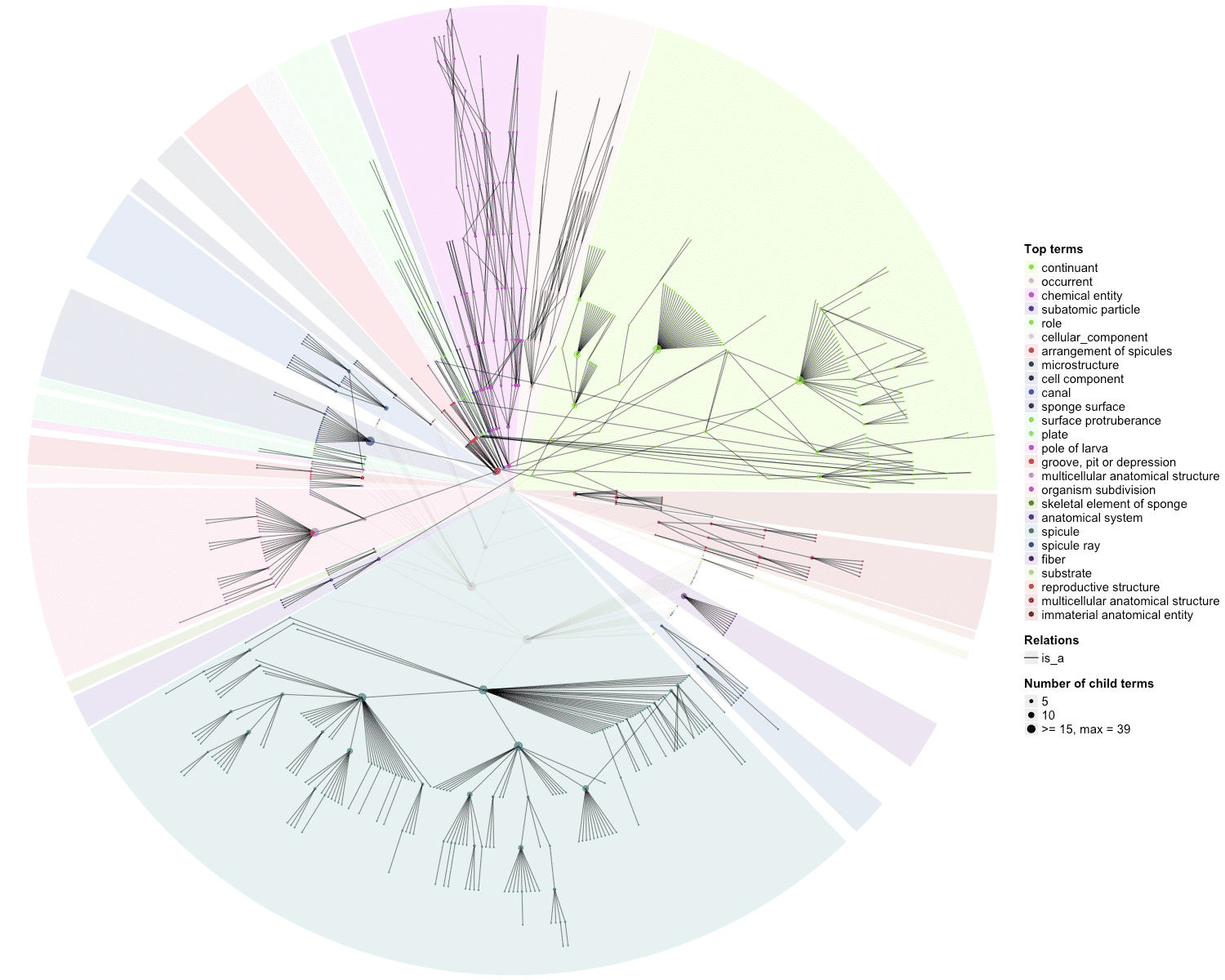

Supplement: Supplementary file 6 — Supplementary Material 6. OBO Foundry gallery [file 12864_2024_10759_MOESM6_ESM.zip › suppl6_OBOFoundry_gallery/image/OBOFoundry_poro.png]

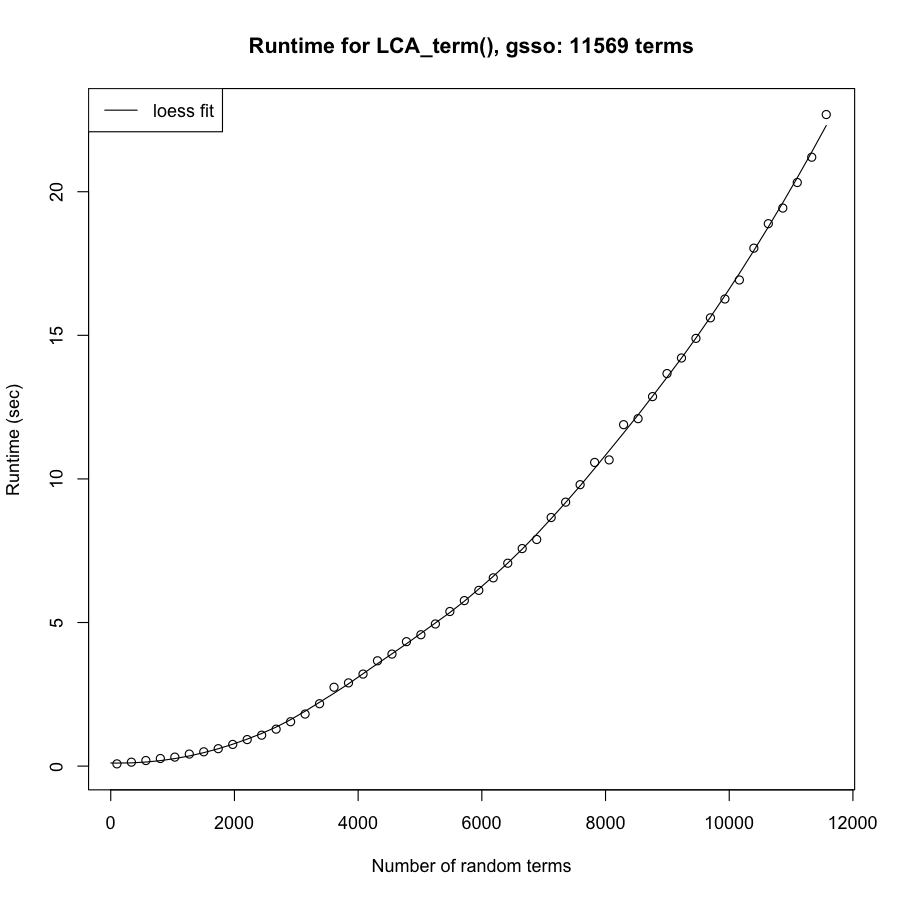

Supplement: Supplementary file 6 — Supplementary Material 6. OBO Foundry gallery [file 12864_2024_10759_MOESM6_ESM.zip › suppl6_OBOFoundry_gallery/image/OBOFoundry_gsso_runtime.png]

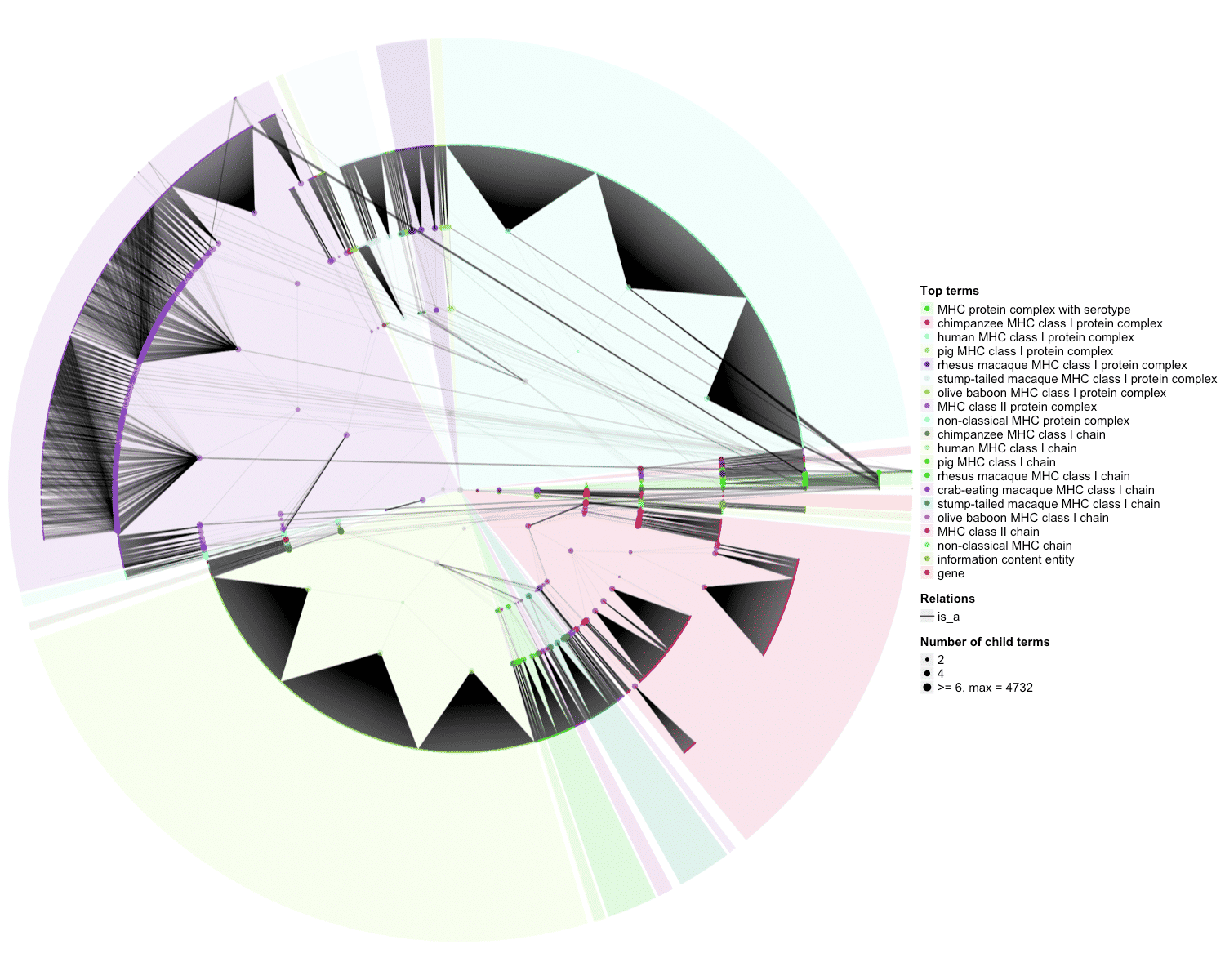

Supplement: Supplementary file 6 — Supplementary Material 6. OBO Foundry gallery [file 12864_2024_10759_MOESM6_ESM.zip › suppl6_OBOFoundry_gallery/image/OBOFoundry_mro.png]

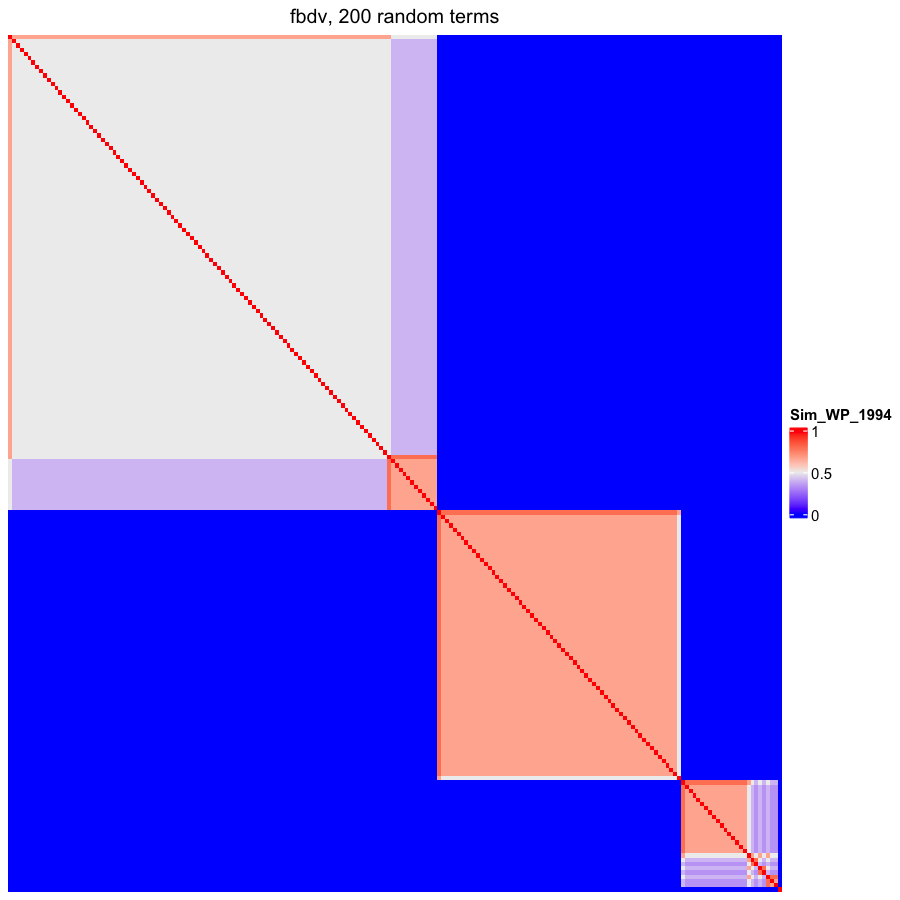

Supplement: Supplementary file 6 — Supplementary Material 6. OBO Foundry gallery [file 12864_2024_10759_MOESM6_ESM.zip › suppl6_OBOFoundry_gallery/image/OBOFoundry_fbdv_heatmap.png]

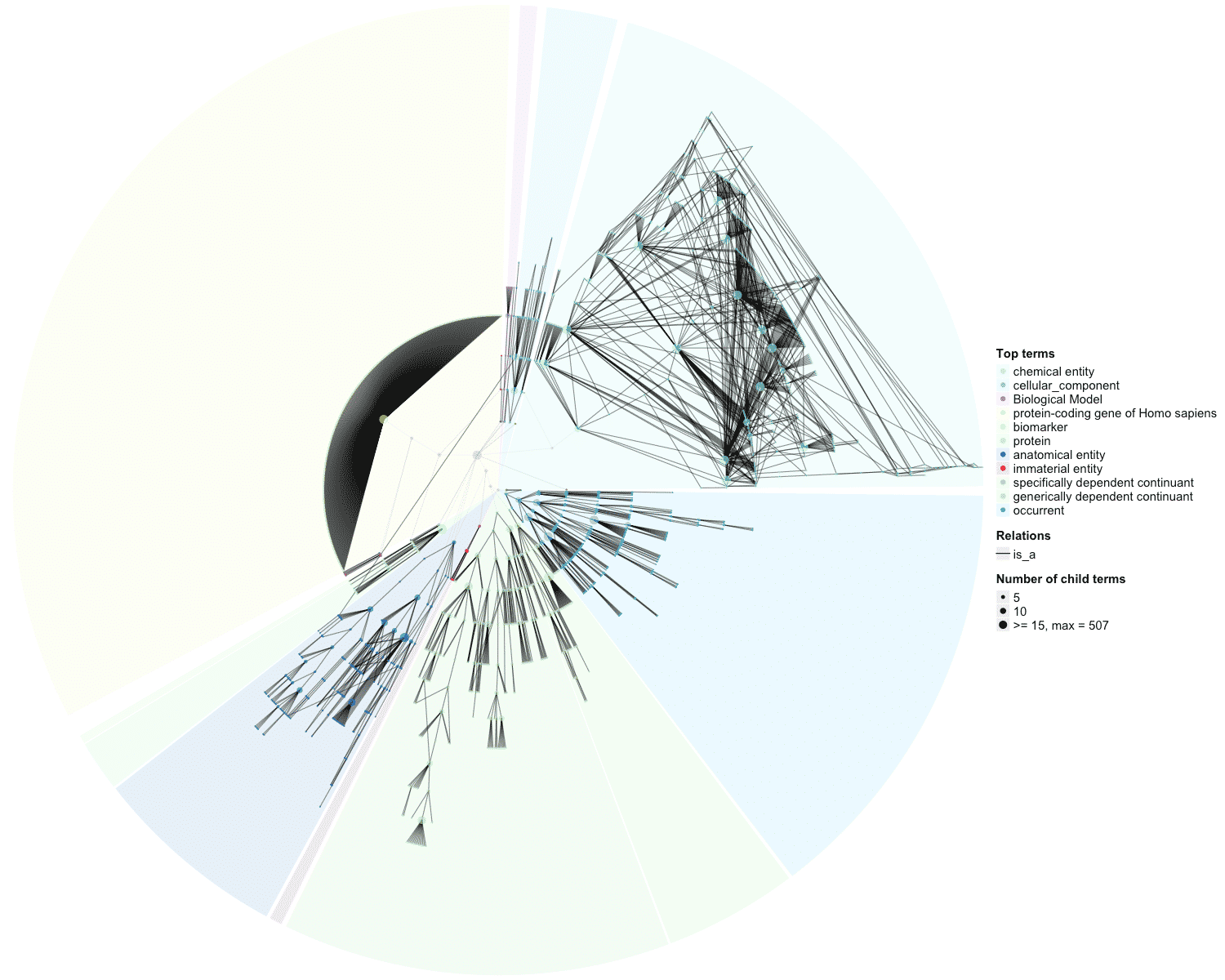

Supplement: Supplementary file 6 — Supplementary Material 6. OBO Foundry gallery [file 12864_2024_10759_MOESM6_ESM.zip › suppl6_OBOFoundry_gallery/image/OBOFoundry_ado.png]

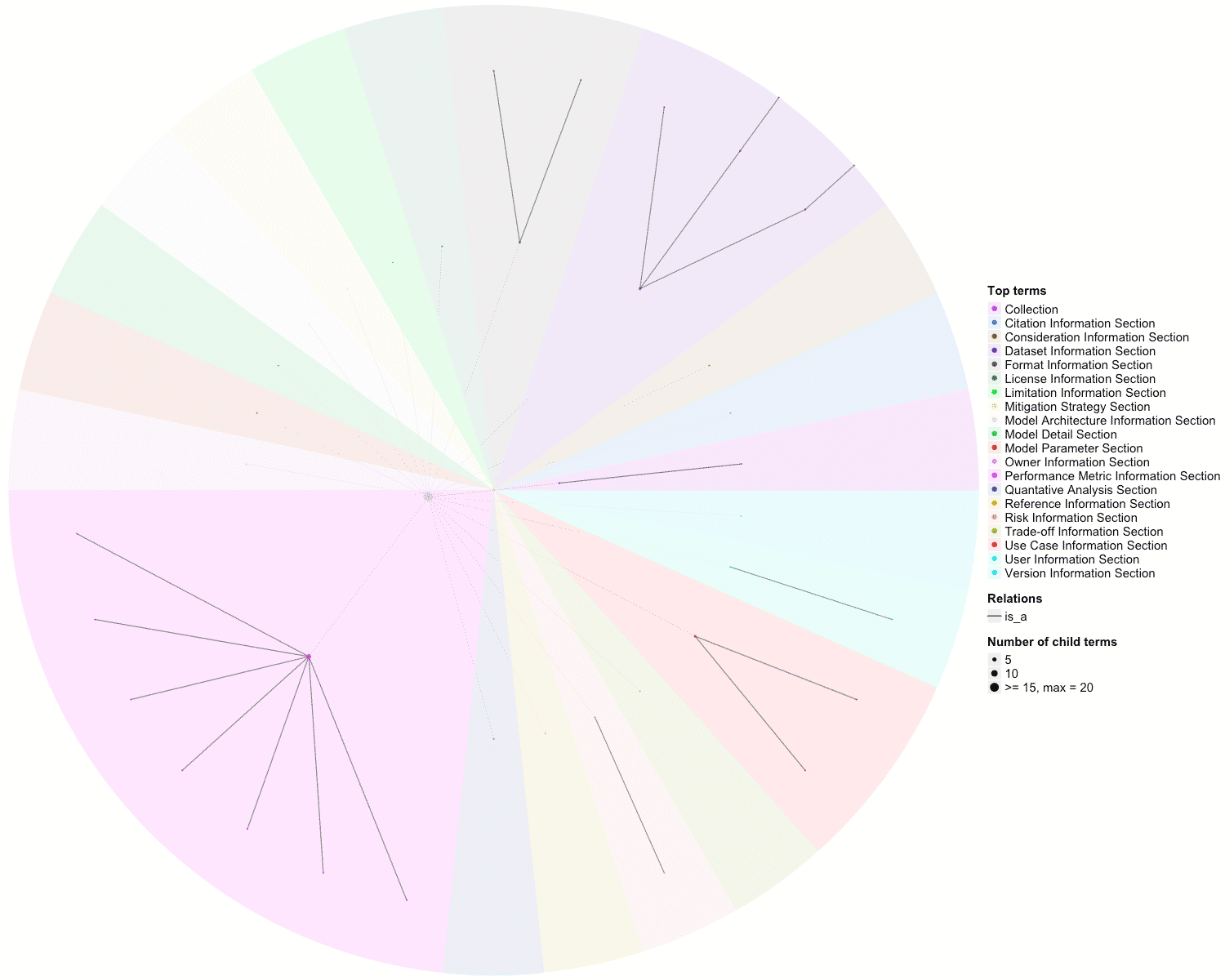

Supplement: Supplementary file 6 — Supplementary Material 6. OBO Foundry gallery [file 12864_2024_10759_MOESM6_ESM.zip › suppl6_OBOFoundry_gallery/image/OBOFoundry_mcro.png]

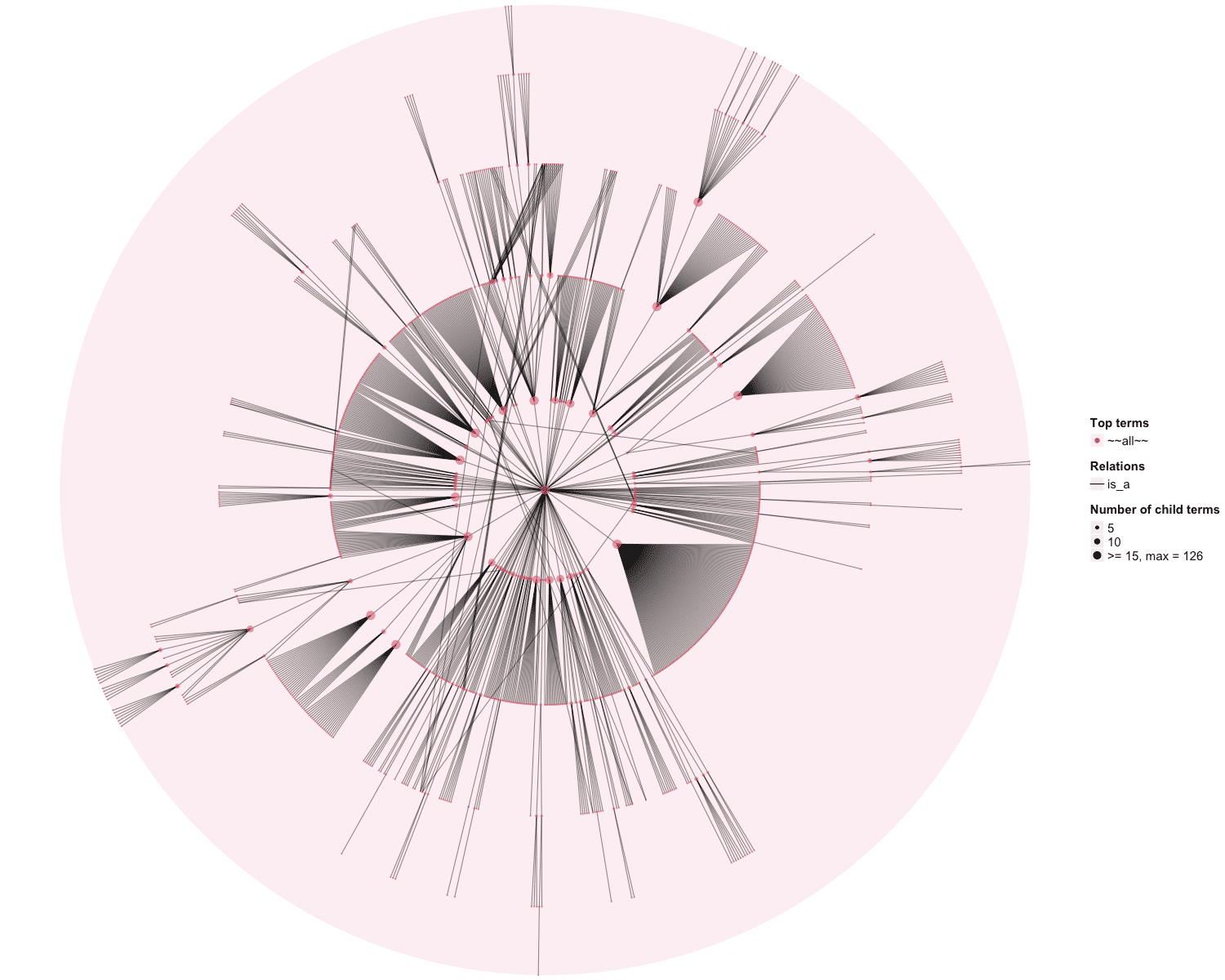

Supplement: Supplementary file 6 — Supplementary Material 6. OBO Foundry gallery [file 12864_2024_10759_MOESM6_ESM.zip › suppl6_OBOFoundry_gallery/image/OBOFoundry_genepio.png]

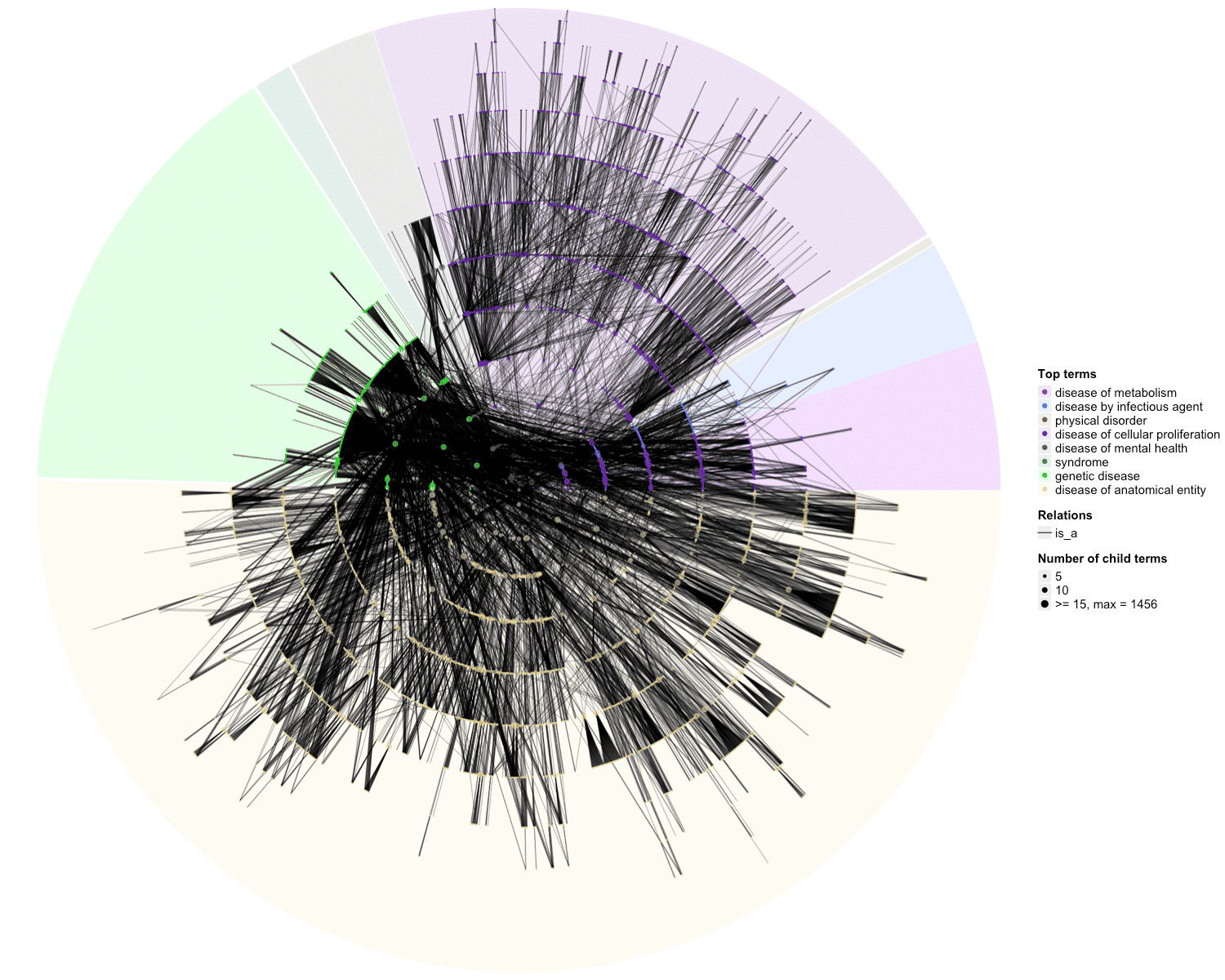

Supplement: Supplementary file 6 — Supplementary Material 6. OBO Foundry gallery [file 12864_2024_10759_MOESM6_ESM.zip › suppl6_OBOFoundry_gallery/image/OBOFoundry_doid.png]

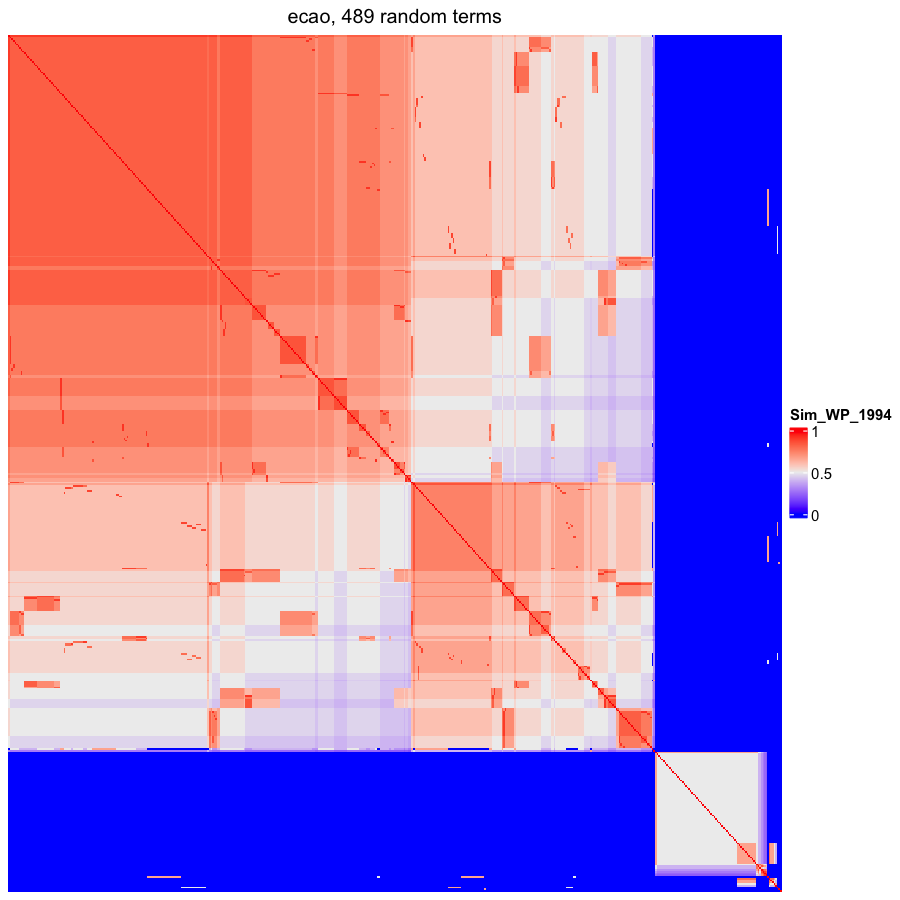

Supplement: Supplementary file 6 — Supplementary Material 6. OBO Foundry gallery [file 12864_2024_10759_MOESM6_ESM.zip › suppl6_OBOFoundry_gallery/image/OBOFoundry_ecao_heatmap.png]

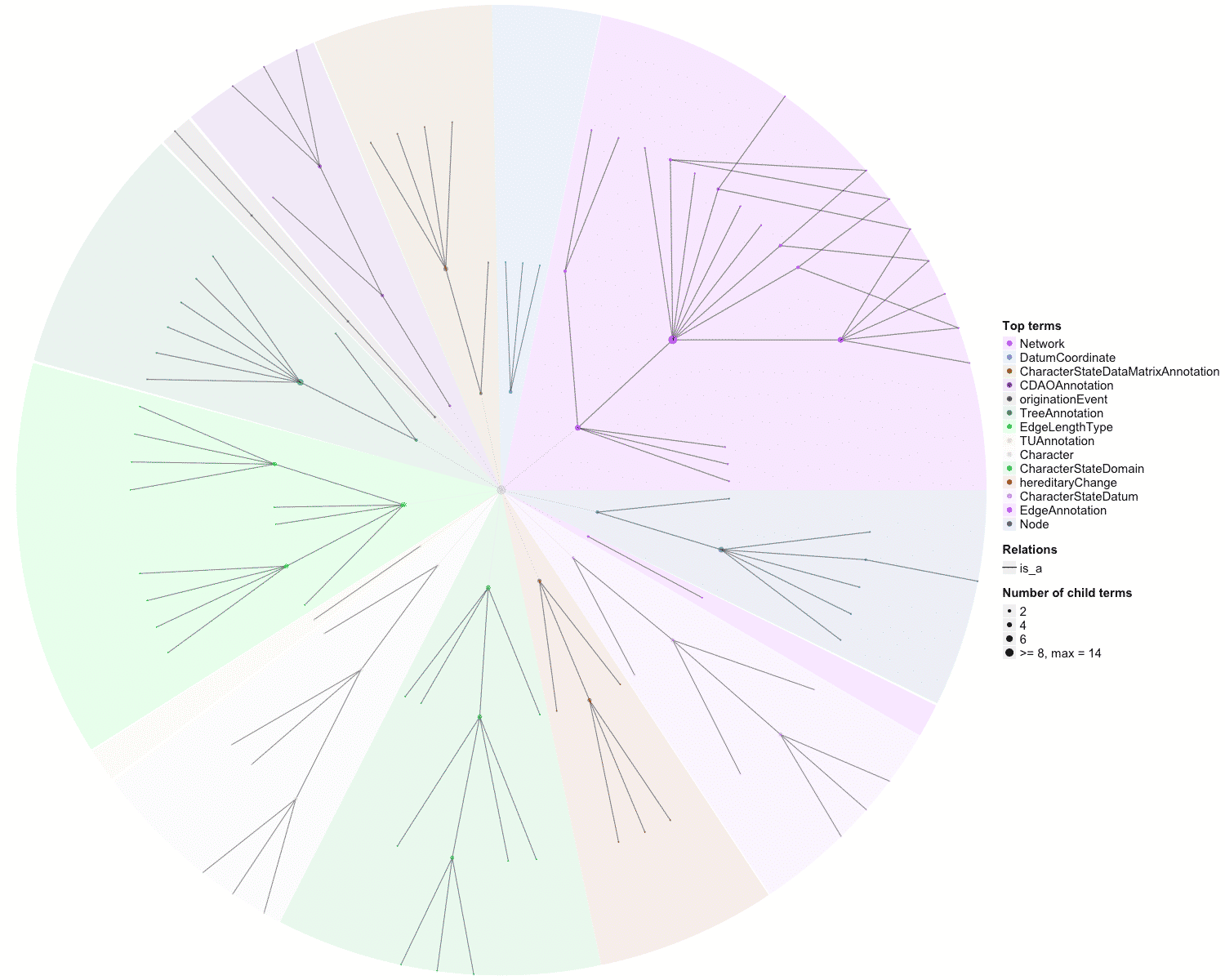

Supplement: Supplementary file 6 — Supplementary Material 6. OBO Foundry gallery [file 12864_2024_10759_MOESM6_ESM.zip › suppl6_OBOFoundry_gallery/image/OBOFoundry_cdao.png]

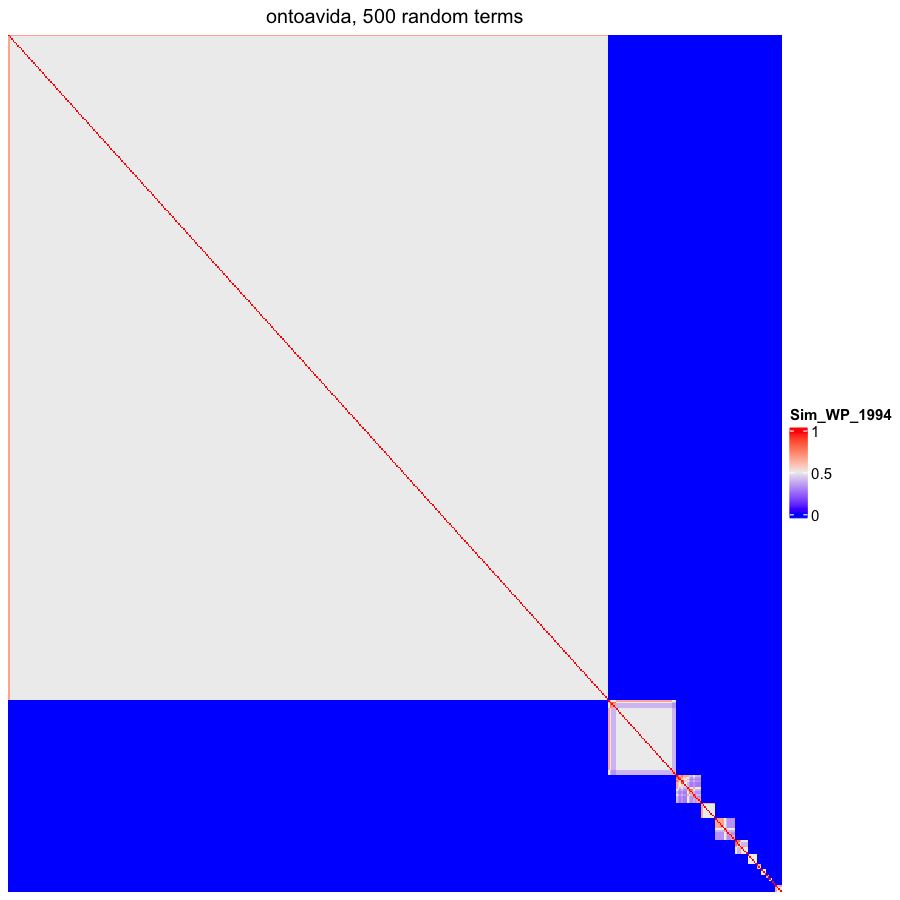

Supplement: Supplementary file 6 — Supplementary Material 6. OBO Foundry gallery [file 12864_2024_10759_MOESM6_ESM.zip › suppl6_OBOFoundry_gallery/image/OBOFoundry_ontoavida_heatmap.png]

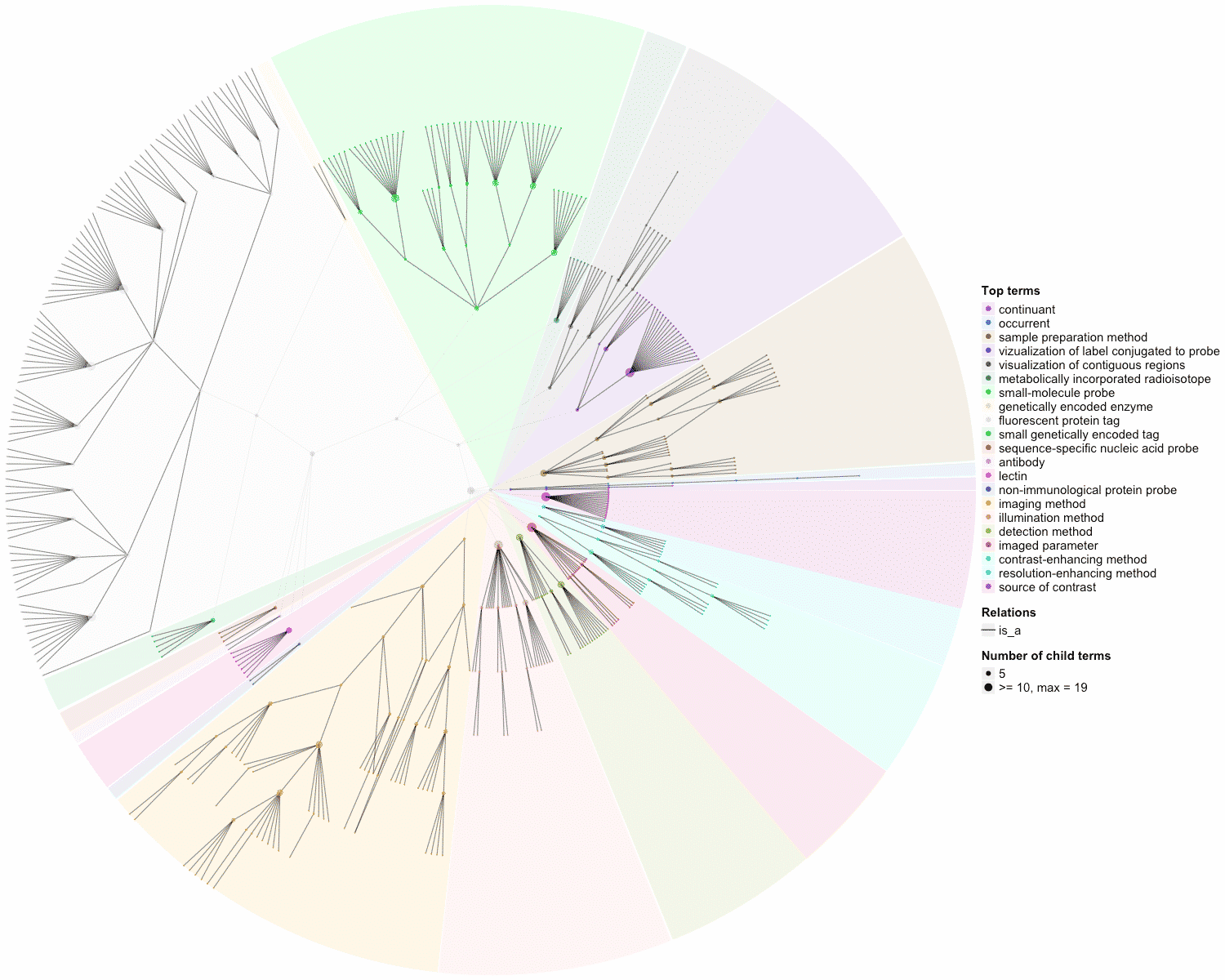

Supplement: Supplementary file 6 — Supplementary Material 6. OBO Foundry gallery [file 12864_2024_10759_MOESM6_ESM.zip › suppl6_OBOFoundry_gallery/image/OBOFoundry_fbbi.png]

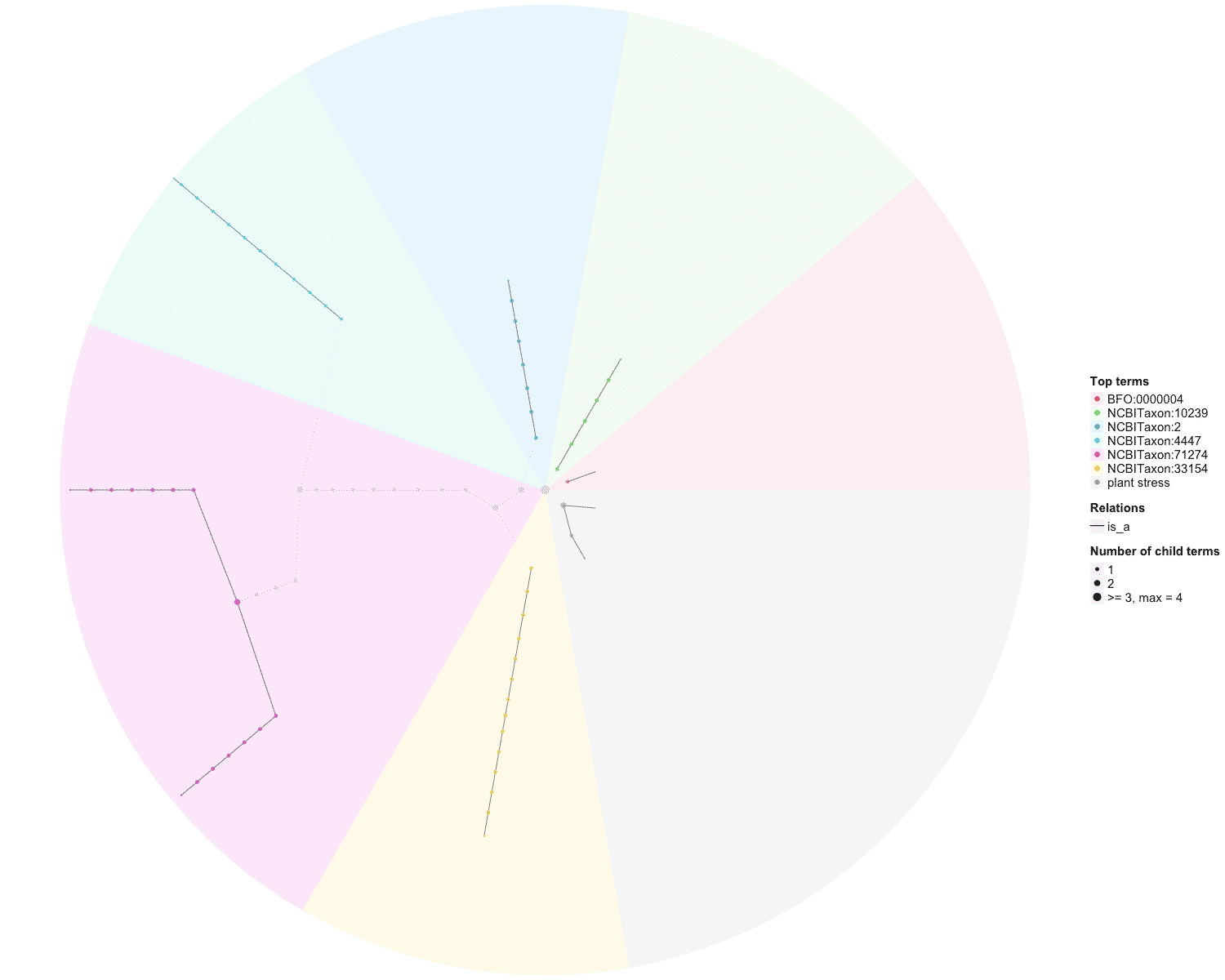

Supplement: Supplementary file 6 — Supplementary Material 6. OBO Foundry gallery [file 12864_2024_10759_MOESM6_ESM.zip › suppl6_OBOFoundry_gallery/image/OBOFoundry_pso.png]

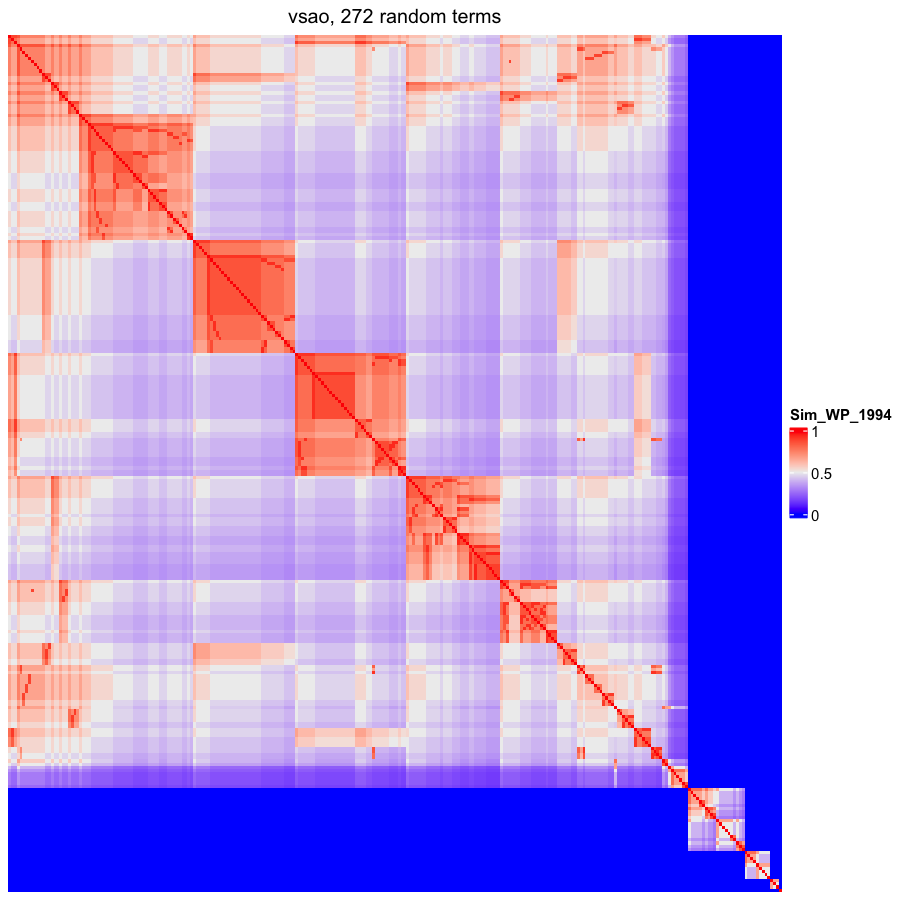

Supplement: Supplementary file 6 — Supplementary Material 6. OBO Foundry gallery [file 12864_2024_10759_MOESM6_ESM.zip › suppl6_OBOFoundry_gallery/image/OBOFoundry_vsao_heatmap.png]

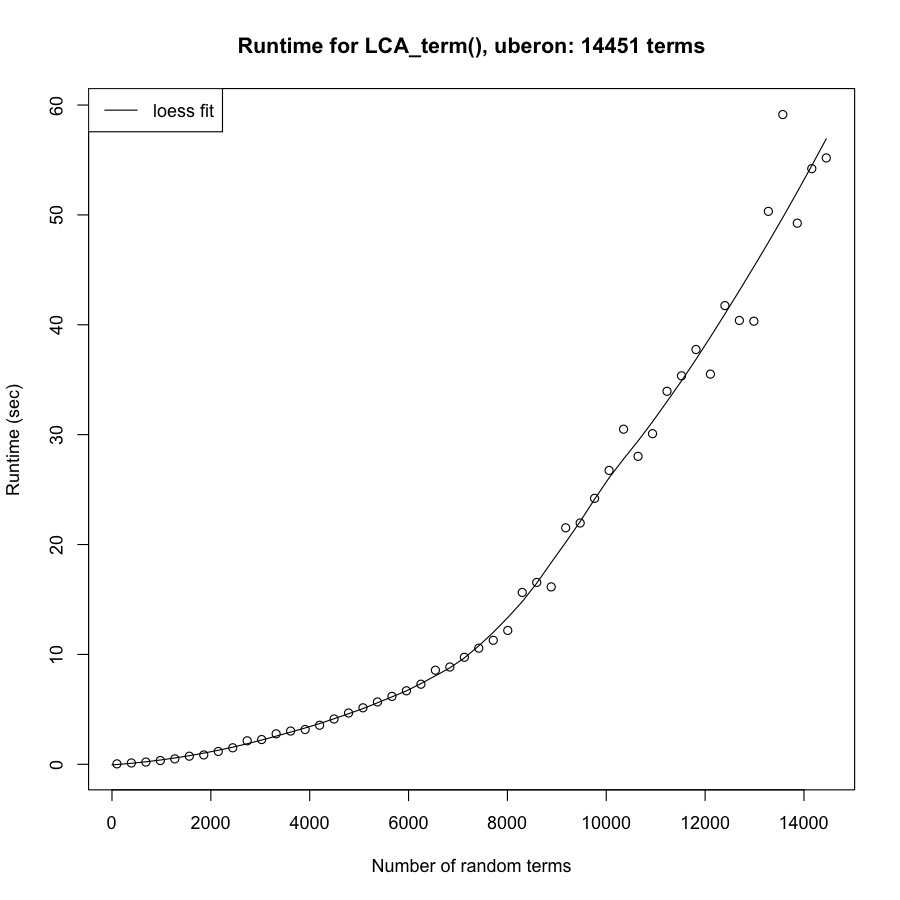

Supplement: Supplementary file 6 — Supplementary Material 6. OBO Foundry gallery [file 12864_2024_10759_MOESM6_ESM.zip › suppl6_OBOFoundry_gallery/image/OBOFoundry_uberon_runtime.png]

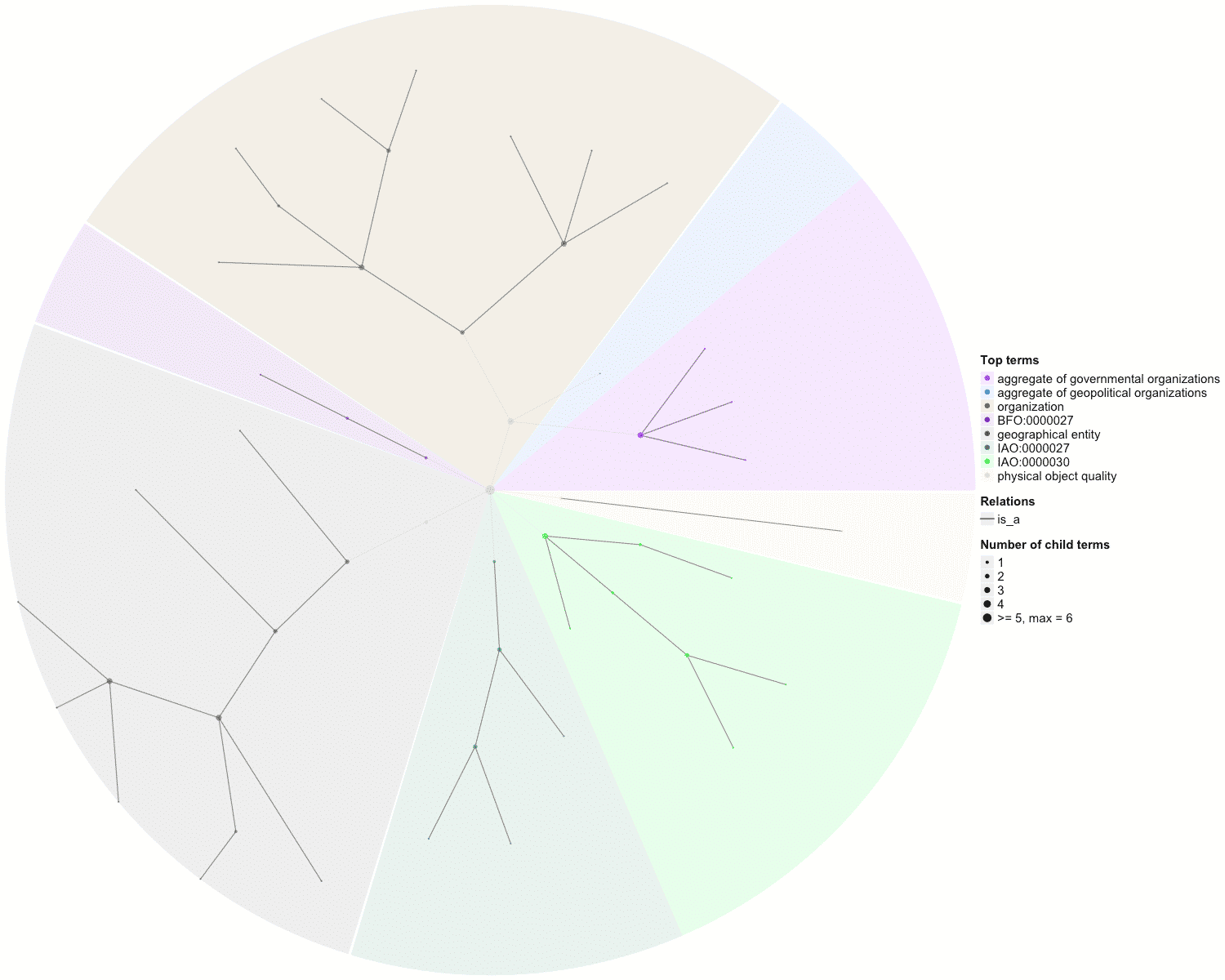

Supplement: Supplementary file 6 — Supplementary Material 6. OBO Foundry gallery [file 12864_2024_10759_MOESM6_ESM.zip › suppl6_OBOFoundry_gallery/image/OBOFoundry_geo.png]
