# Supplementary figures and images for: simona: a comprehensive R package for semantic similarity analysis on bio-ontologies (part 2 of 3)
Source: BMC Genomics. 2024 Sep 16;25:869. doi: 10.1186/s12864-024-10759-4 (PMC11406866; doi:10.1186/s12864-024-10759-4)

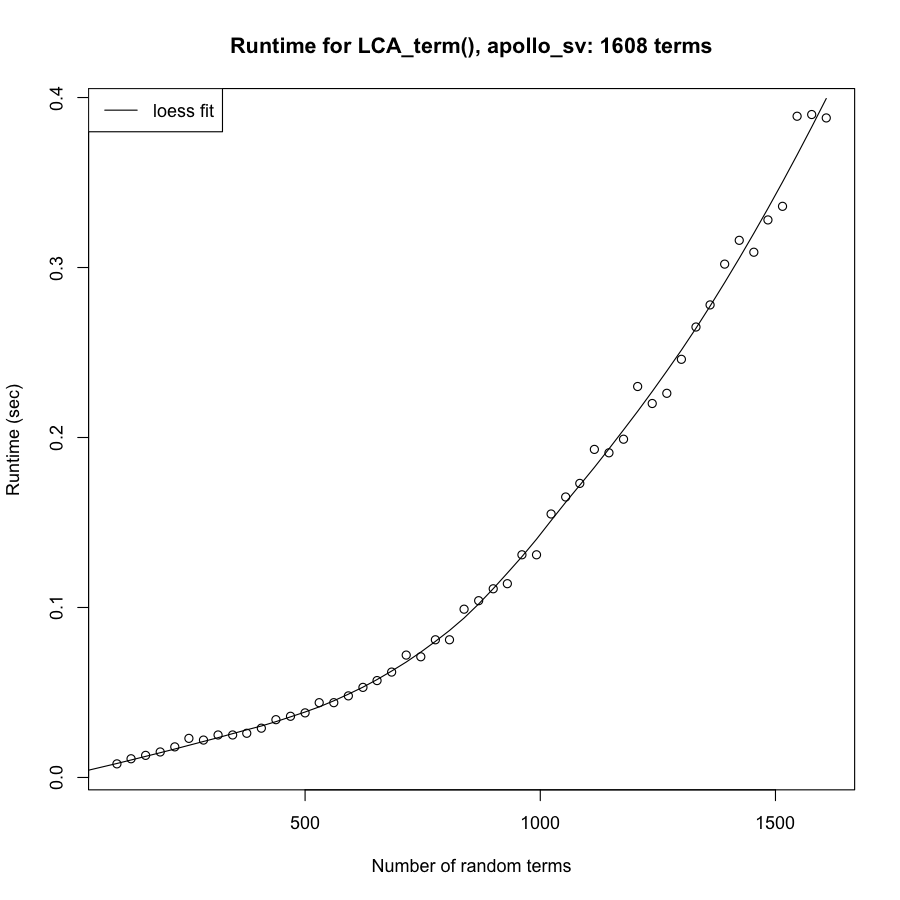

Supplement: Supplementary file 6 — Supplementary Material 6. OBO Foundry gallery [file 12864_2024_10759_MOESM6_ESM.zip › suppl6_OBOFoundry_gallery/image/OBOFoundry_apollo_sv_runtime.png]

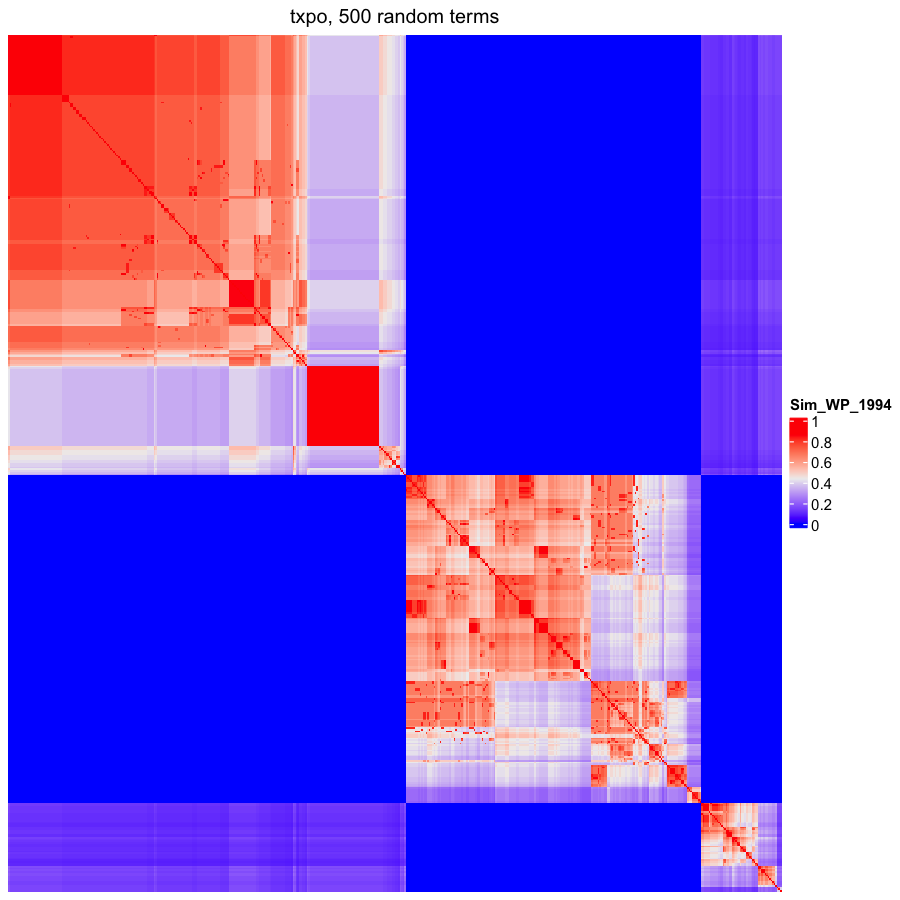

Supplement: Supplementary file 6 — Supplementary Material 6. OBO Foundry gallery [file 12864_2024_10759_MOESM6_ESM.zip › suppl6_OBOFoundry_gallery/image/OBOFoundry_txpo_heatmap.png]

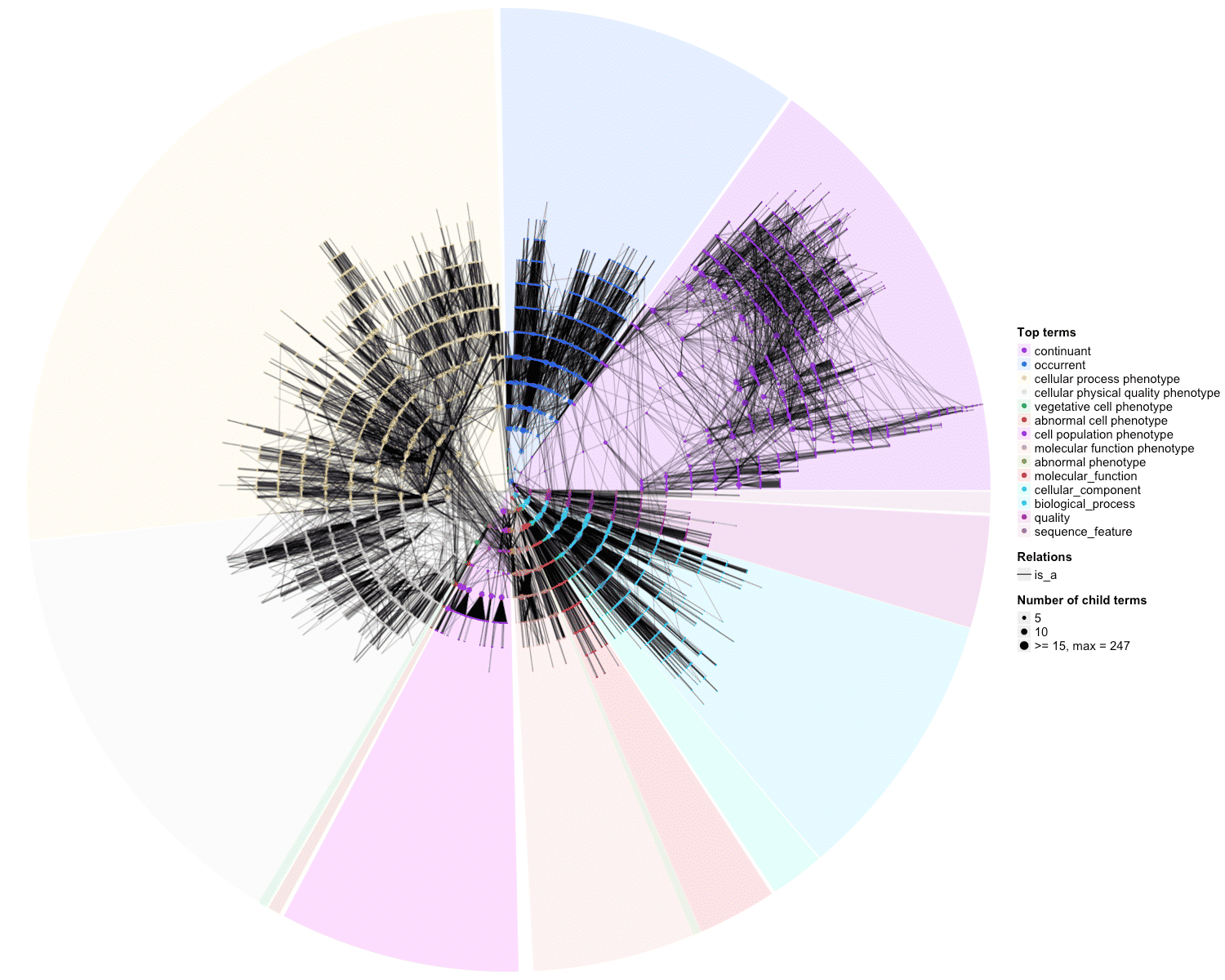

Supplement: Supplementary file 6 — Supplementary Material 6. OBO Foundry gallery [file 12864_2024_10759_MOESM6_ESM.zip › suppl6_OBOFoundry_gallery/image/OBOFoundry_fypo.png]

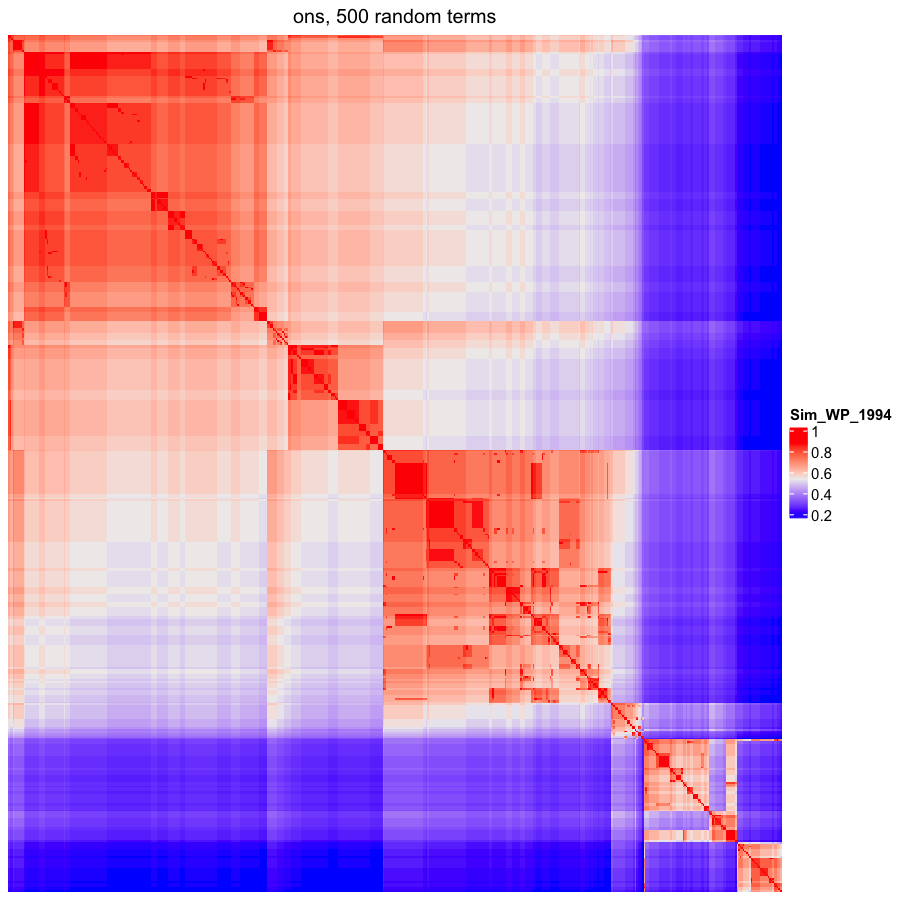

Supplement: Supplementary file 6 — Supplementary Material 6. OBO Foundry gallery [file 12864_2024_10759_MOESM6_ESM.zip › suppl6_OBOFoundry_gallery/image/OBOFoundry_ons_heatmap.png]

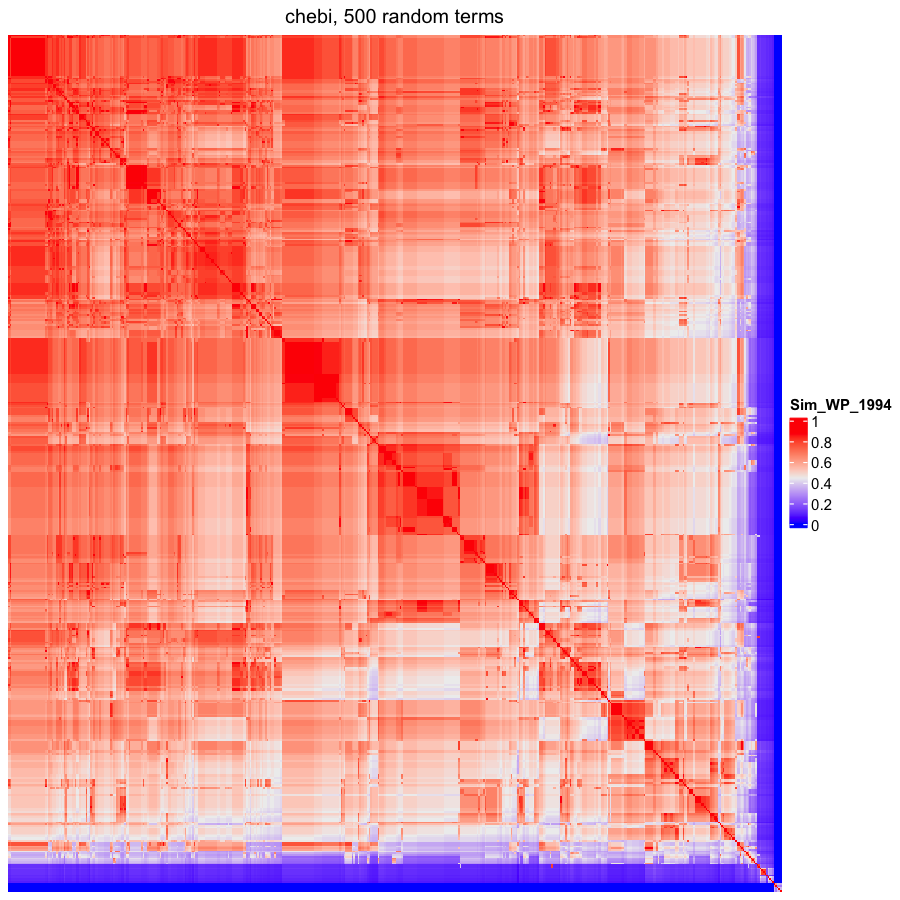

Supplement: Supplementary file 6 — Supplementary Material 6. OBO Foundry gallery [file 12864_2024_10759_MOESM6_ESM.zip › suppl6_OBOFoundry_gallery/image/OBOFoundry_chebi_heatmap.png]

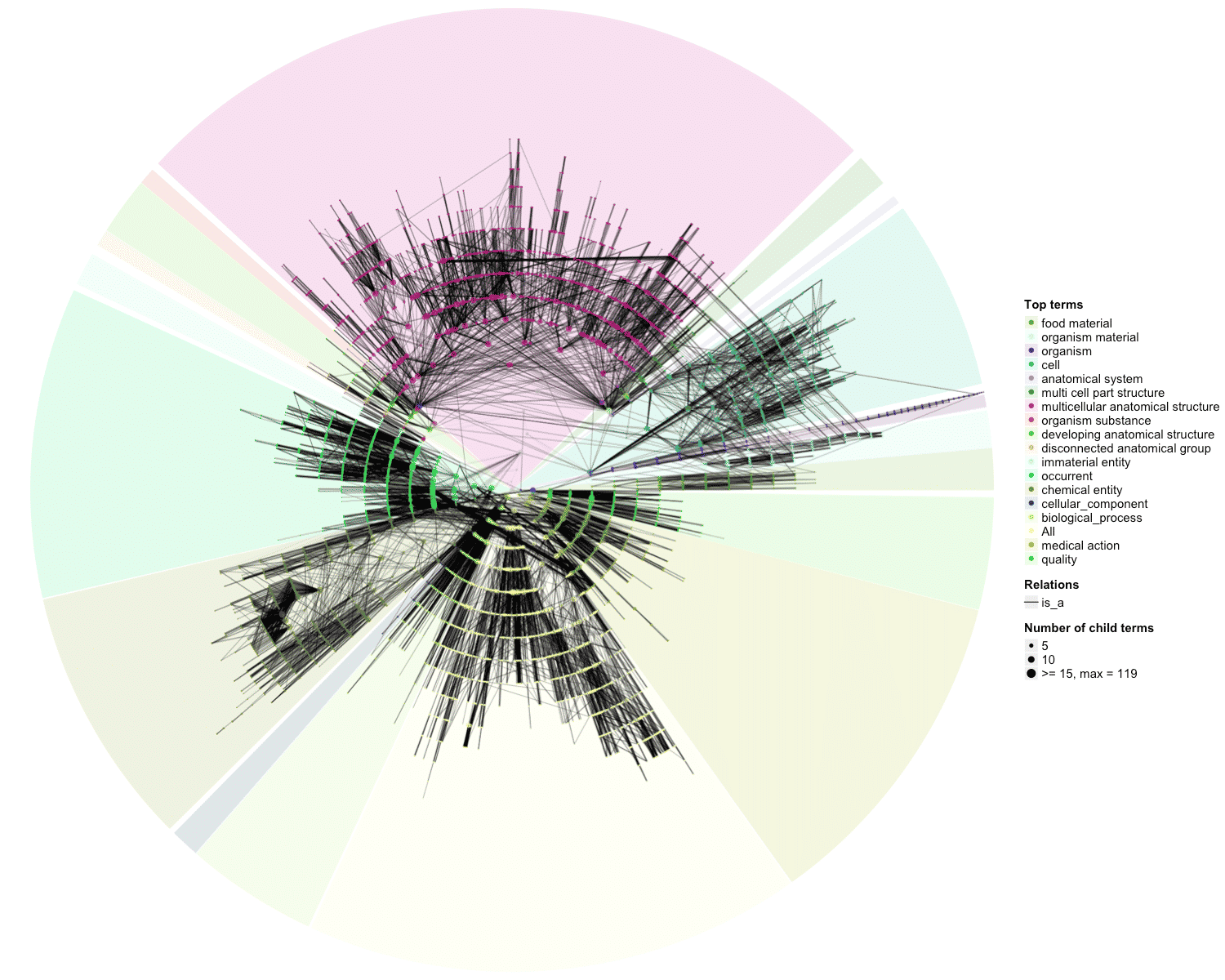

Supplement: Supplementary file 6 — Supplementary Material 6. OBO Foundry gallery [file 12864_2024_10759_MOESM6_ESM.zip › suppl6_OBOFoundry_gallery/image/OBOFoundry_maxo.png]

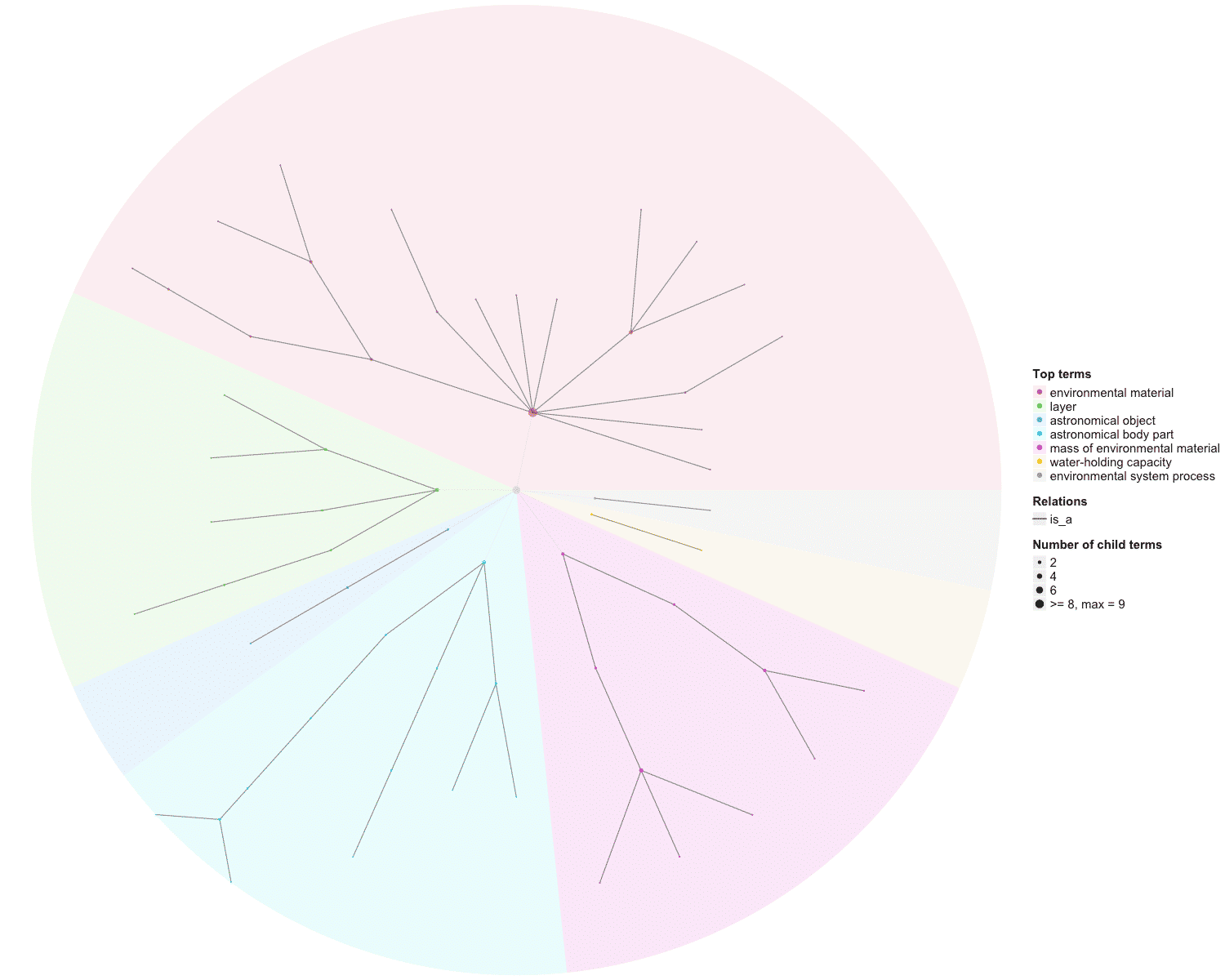

Supplement: Supplementary file 6 — Supplementary Material 6. OBO Foundry gallery [file 12864_2024_10759_MOESM6_ESM.zip › suppl6_OBOFoundry_gallery/image/OBOFoundry_envo.png]

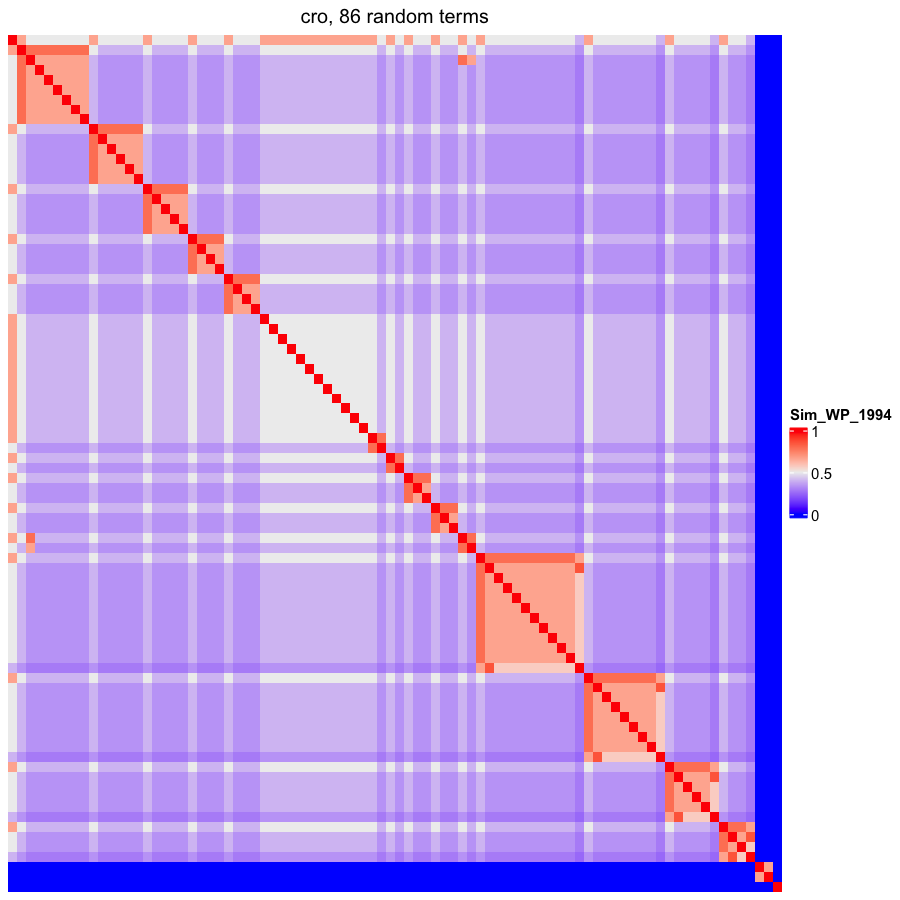

Supplement: Supplementary file 6 — Supplementary Material 6. OBO Foundry gallery [file 12864_2024_10759_MOESM6_ESM.zip › suppl6_OBOFoundry_gallery/image/OBOFoundry_cro_heatmap.png]

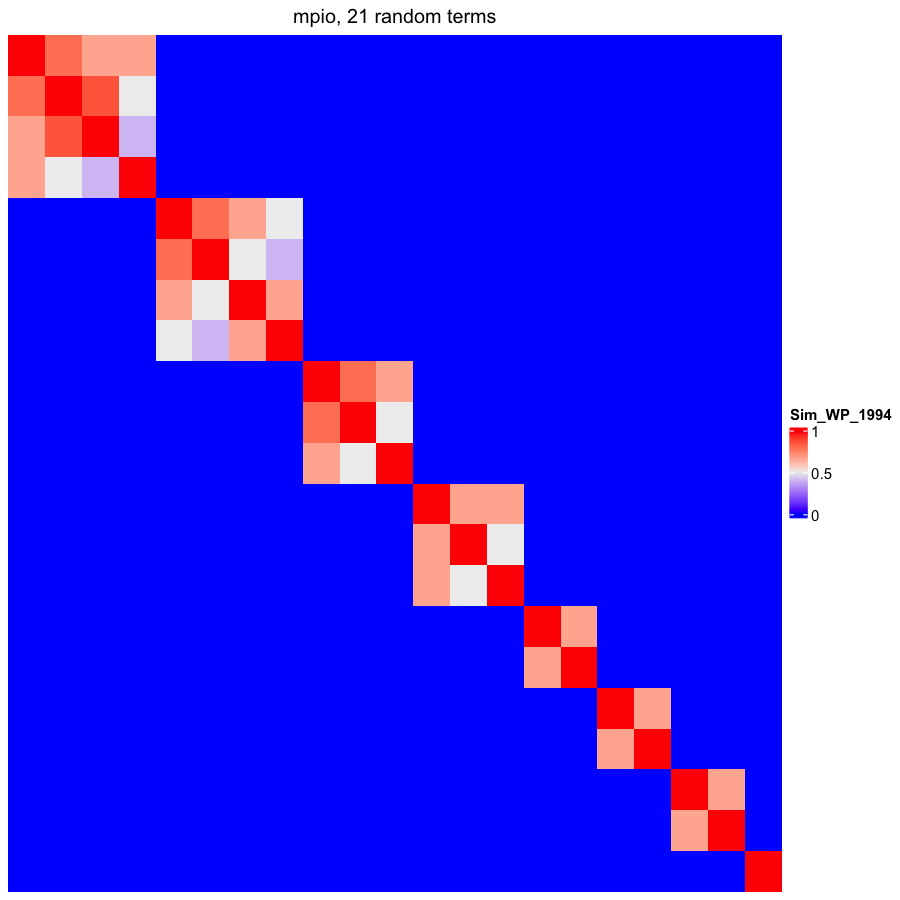

Supplement: Supplementary file 6 — Supplementary Material 6. OBO Foundry gallery [file 12864_2024_10759_MOESM6_ESM.zip › suppl6_OBOFoundry_gallery/image/OBOFoundry_mpio_heatmap.png]

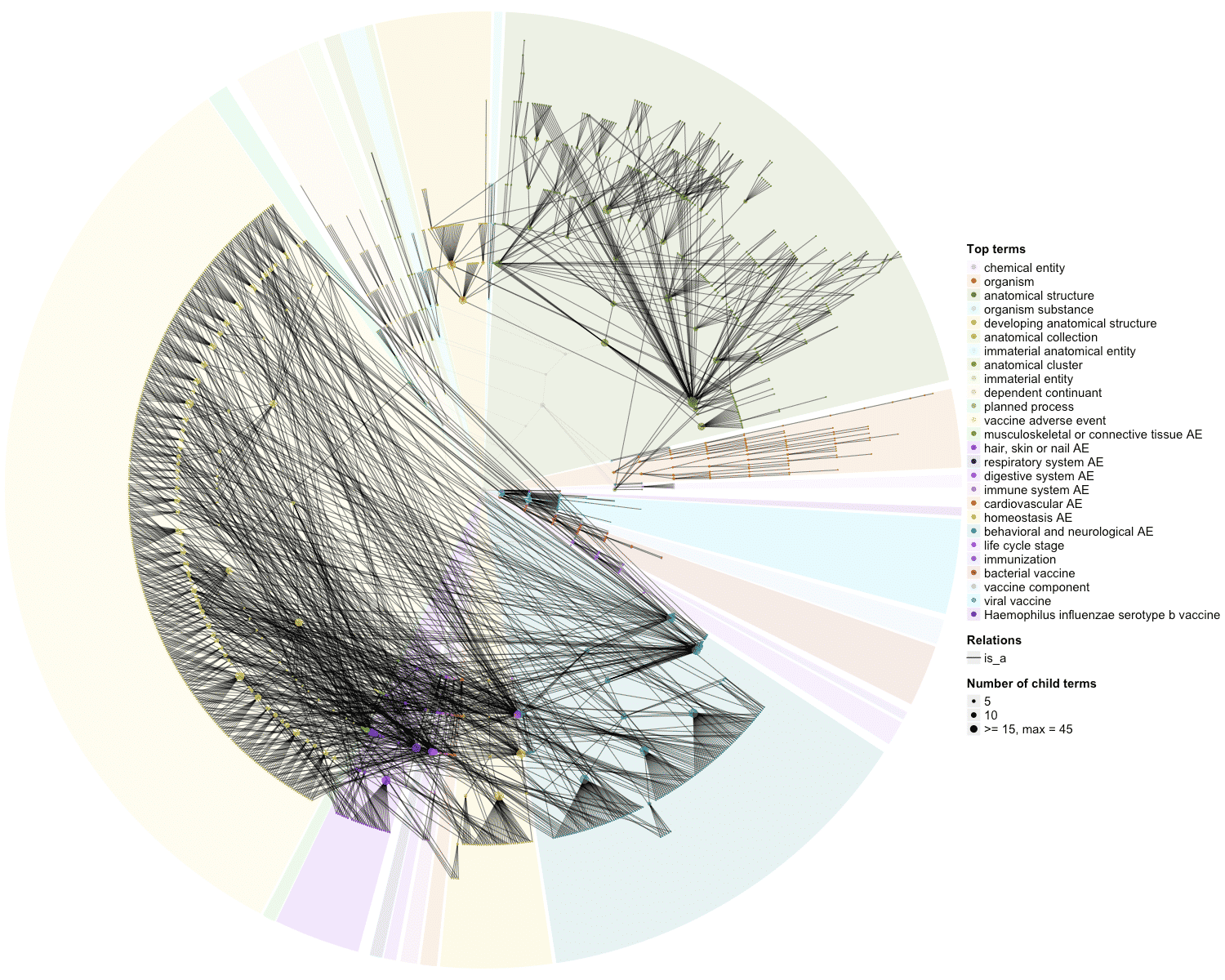

Supplement: Supplementary file 6 — Supplementary Material 6. OBO Foundry gallery [file 12864_2024_10759_MOESM6_ESM.zip › suppl6_OBOFoundry_gallery/image/OBOFoundry_ovae.png]

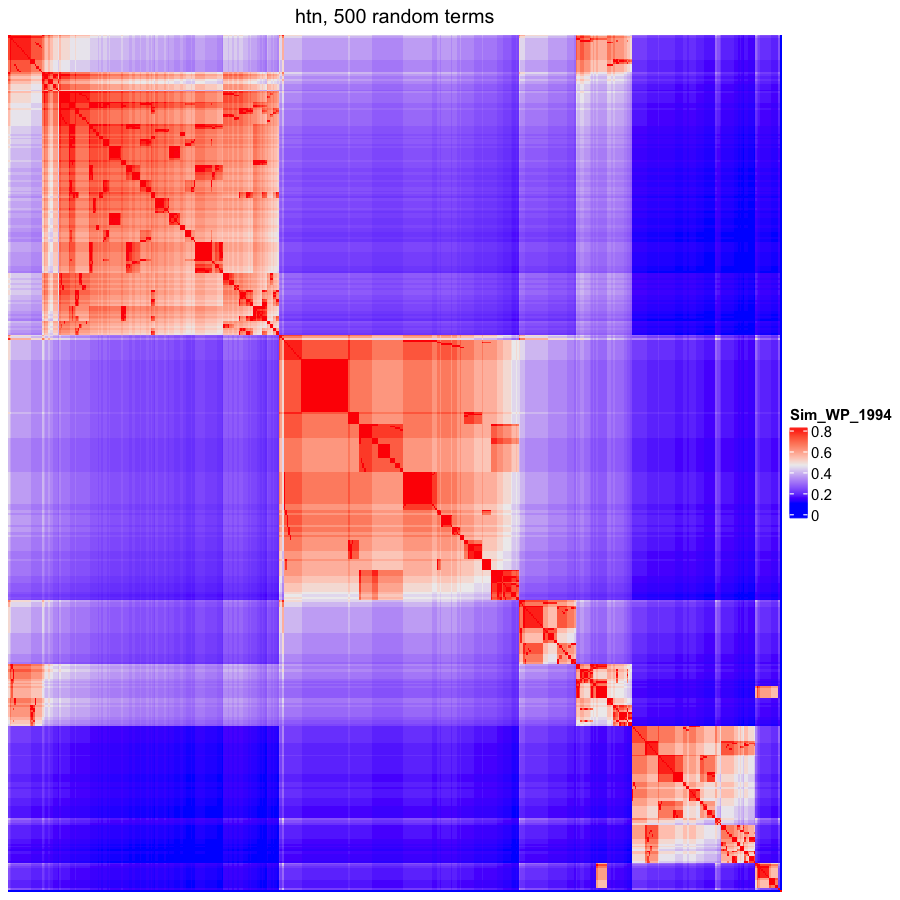

Supplement: Supplementary file 6 — Supplementary Material 6. OBO Foundry gallery [file 12864_2024_10759_MOESM6_ESM.zip › suppl6_OBOFoundry_gallery/image/OBOFoundry_htn_heatmap.png]

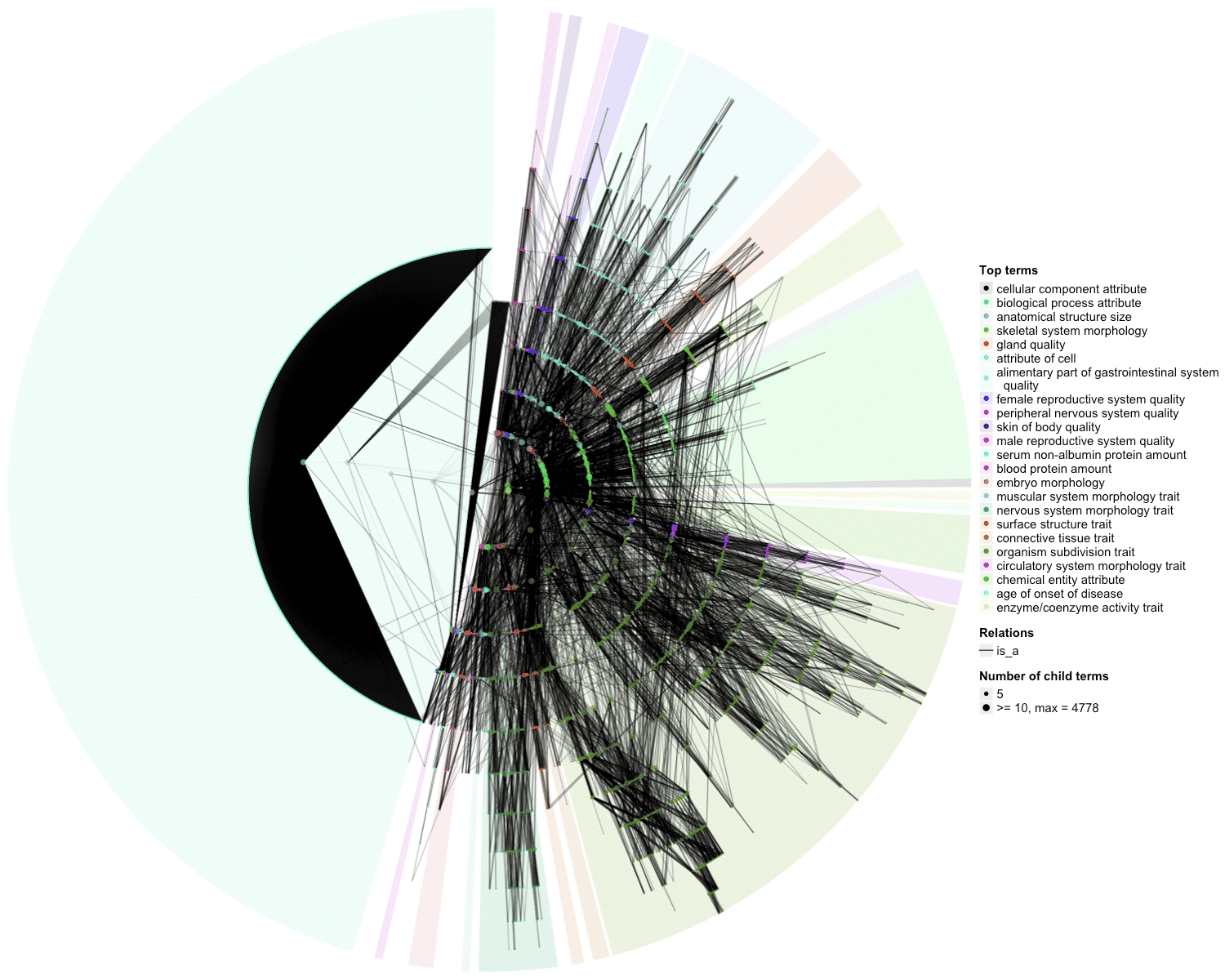

Supplement: Supplementary file 6 — Supplementary Material 6. OBO Foundry gallery [file 12864_2024_10759_MOESM6_ESM.zip › suppl6_OBOFoundry_gallery/image/OBOFoundry_oba.png]

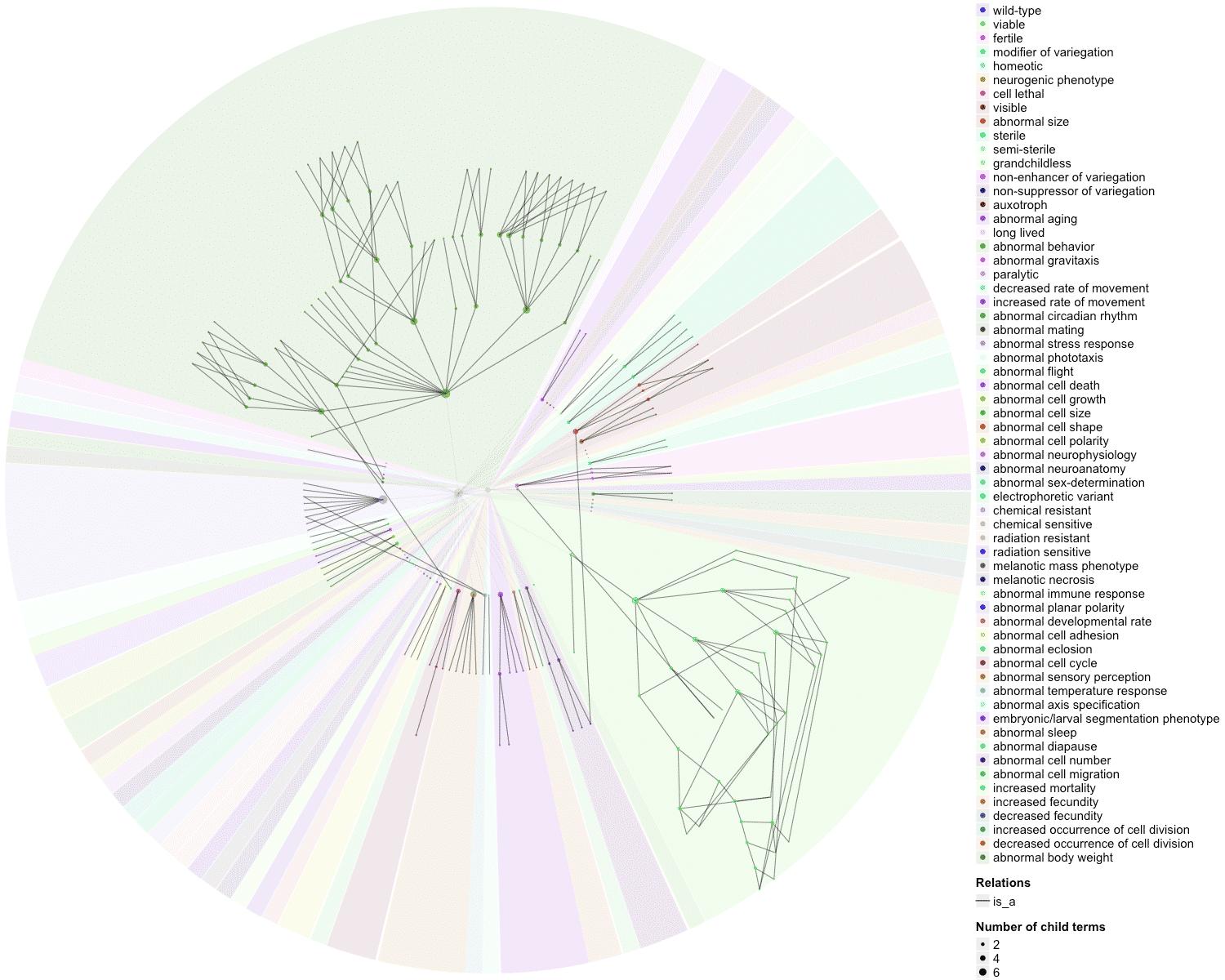

Supplement: Supplementary file 6 — Supplementary Material 6. OBO Foundry gallery [file 12864_2024_10759_MOESM6_ESM.zip › suppl6_OBOFoundry_gallery/image/OBOFoundry_dpo.png]

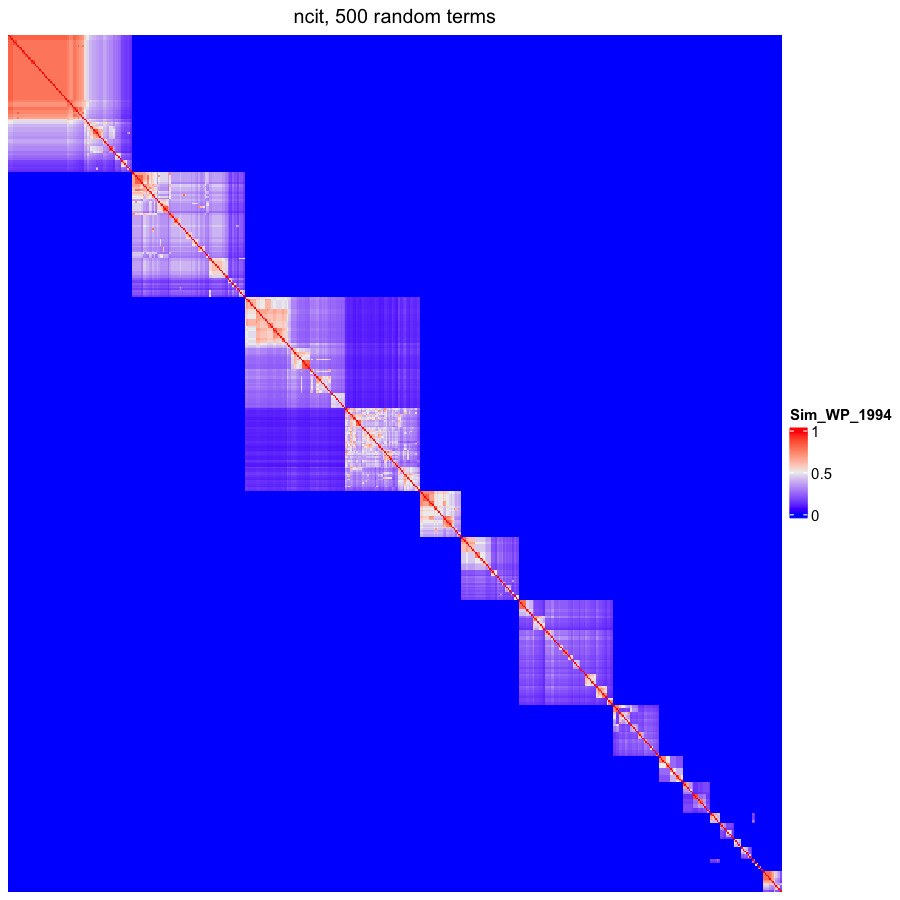

Supplement: Supplementary file 6 — Supplementary Material 6. OBO Foundry gallery [file 12864_2024_10759_MOESM6_ESM.zip › suppl6_OBOFoundry_gallery/image/OBOFoundry_ncit_heatmap.png]

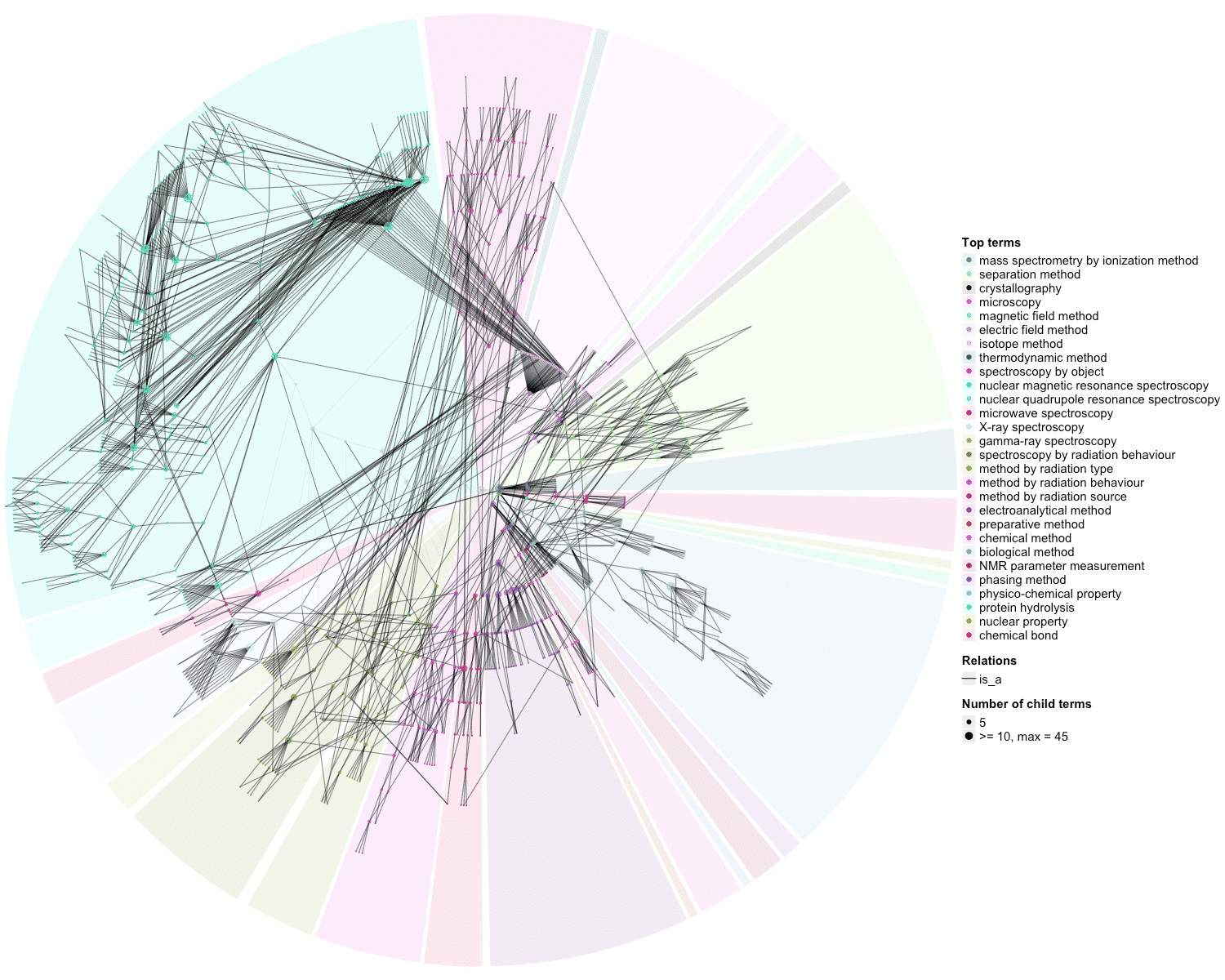

Supplement: Supplementary file 6 — Supplementary Material 6. OBO Foundry gallery [file 12864_2024_10759_MOESM6_ESM.zip › suppl6_OBOFoundry_gallery/image/OBOFoundry_fix.png]

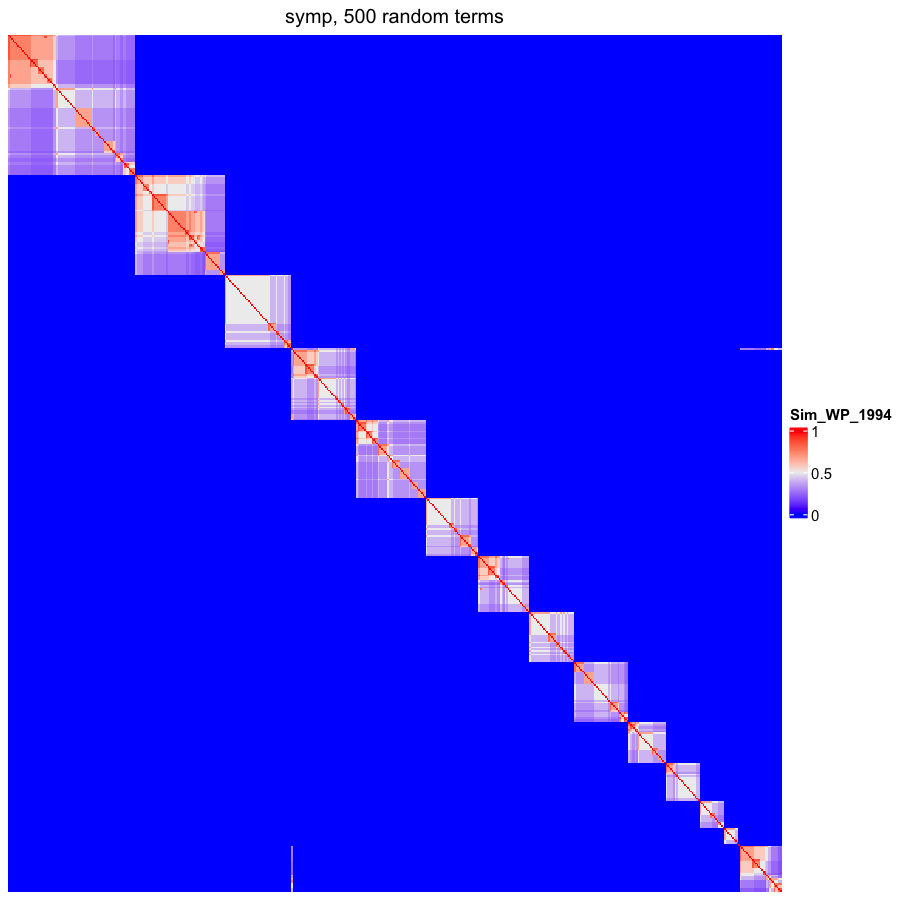

Supplement: Supplementary file 6 — Supplementary Material 6. OBO Foundry gallery [file 12864_2024_10759_MOESM6_ESM.zip › suppl6_OBOFoundry_gallery/image/OBOFoundry_symp_heatmap.png]

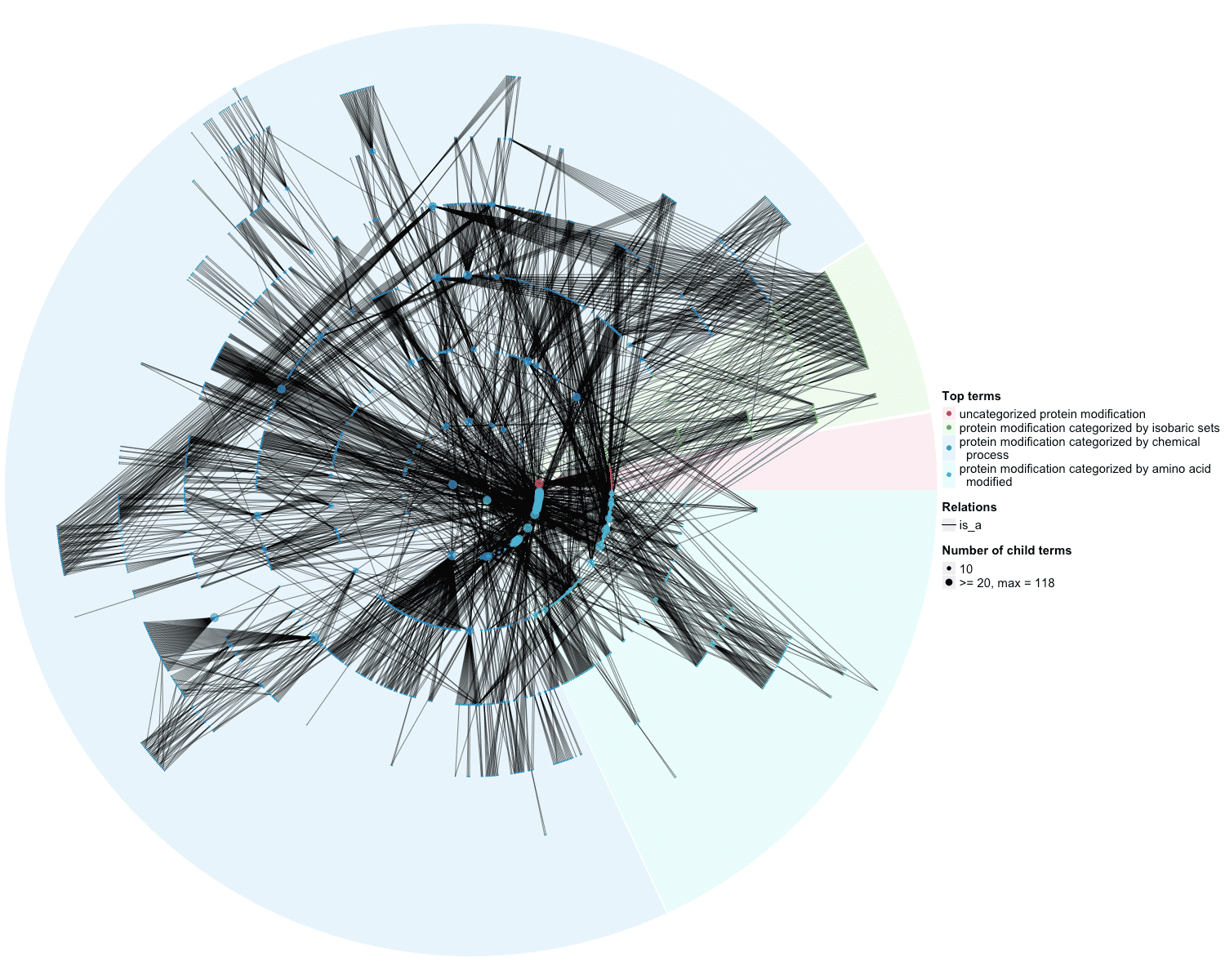

Supplement: Supplementary file 6 — Supplementary Material 6. OBO Foundry gallery [file 12864_2024_10759_MOESM6_ESM.zip › suppl6_OBOFoundry_gallery/image/OBOFoundry_mod.png]

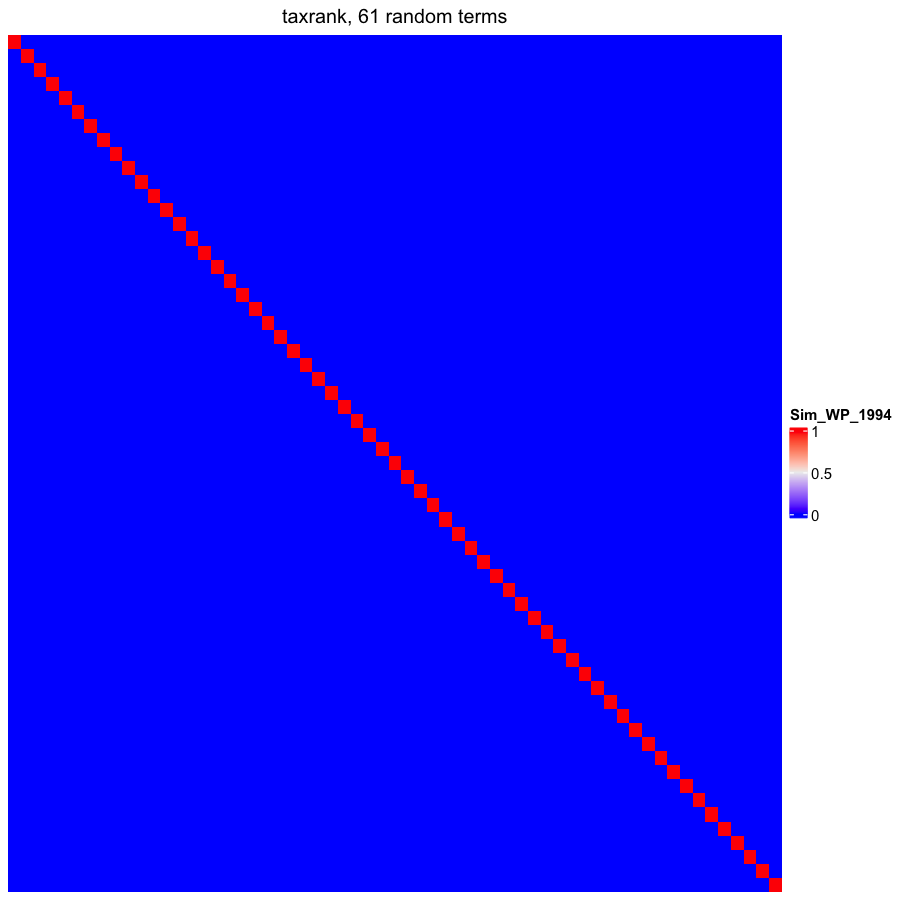

Supplement: Supplementary file 6 — Supplementary Material 6. OBO Foundry gallery [file 12864_2024_10759_MOESM6_ESM.zip › suppl6_OBOFoundry_gallery/image/OBOFoundry_taxrank_heatmap.png]

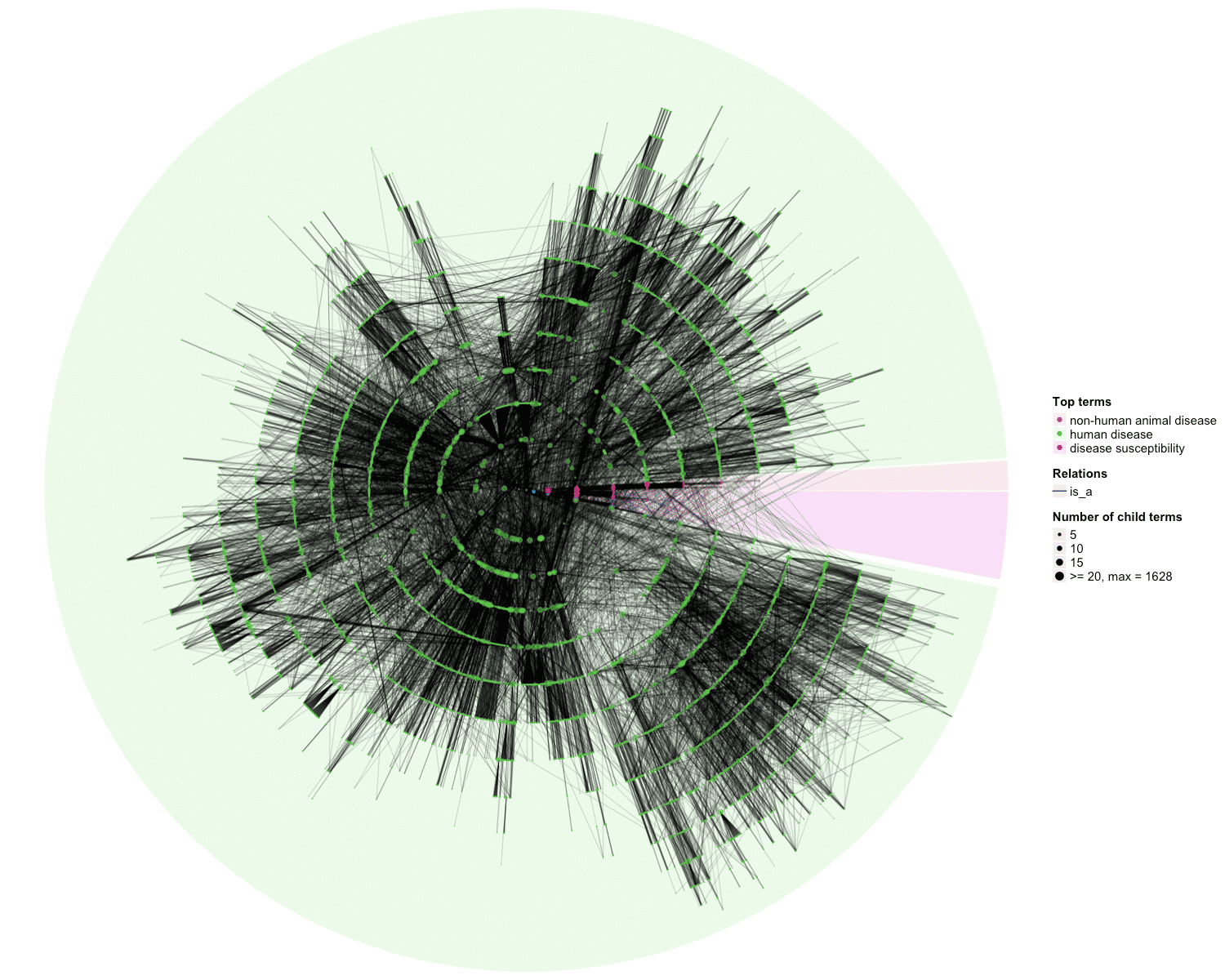

Supplement: Supplementary file 6 — Supplementary Material 6. OBO Foundry gallery [file 12864_2024_10759_MOESM6_ESM.zip › suppl6_OBOFoundry_gallery/image/OBOFoundry_mondo.png]

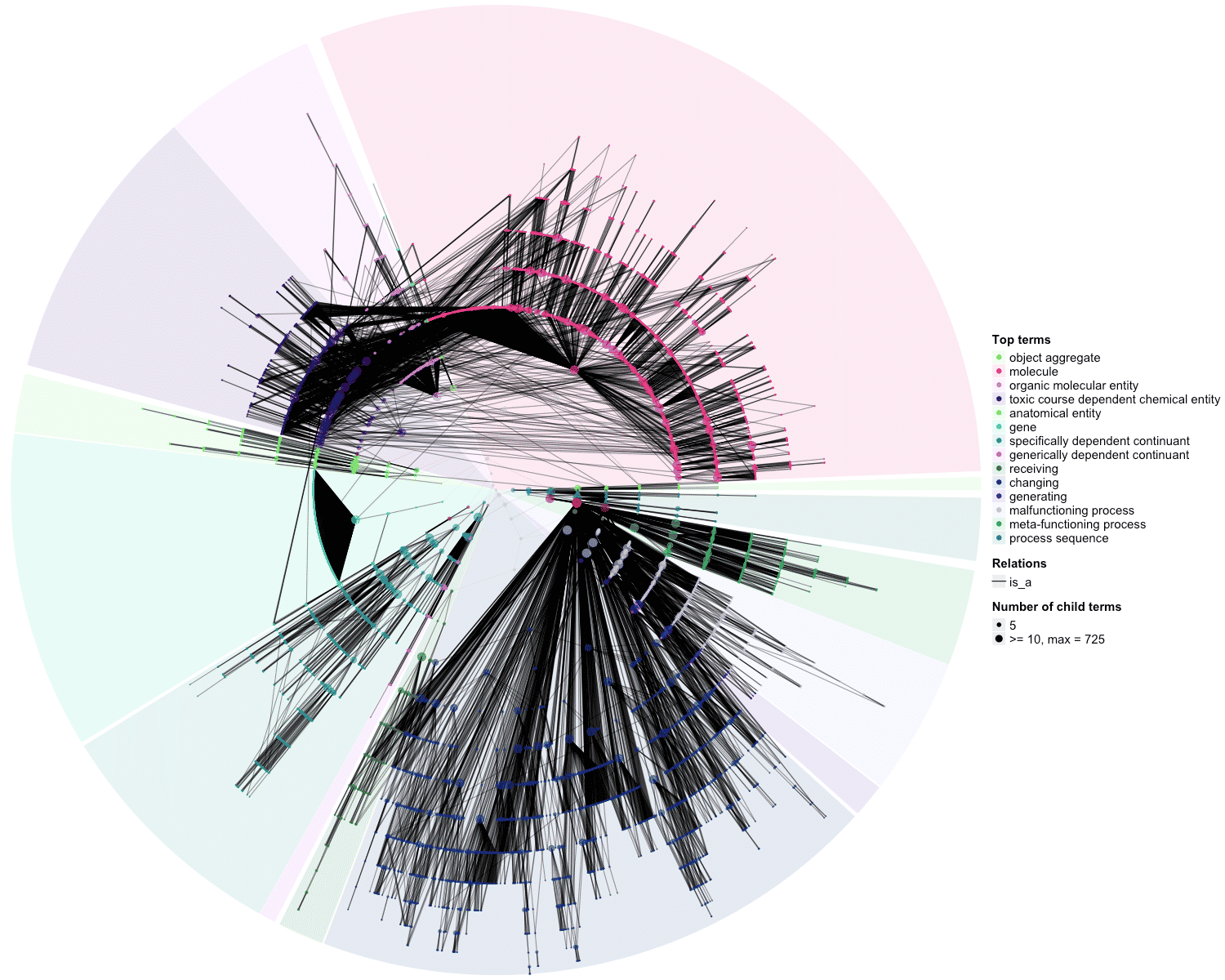

Supplement: Supplementary file 6 — Supplementary Material 6. OBO Foundry gallery [file 12864_2024_10759_MOESM6_ESM.zip › suppl6_OBOFoundry_gallery/image/OBOFoundry_txpo.png]

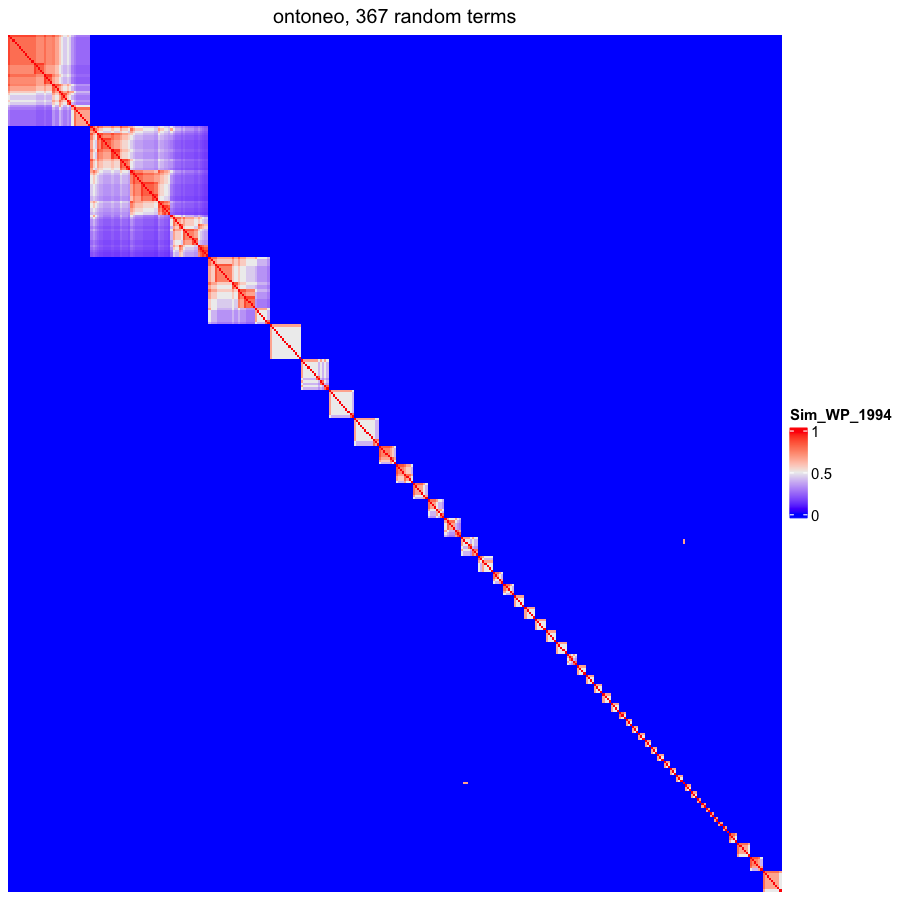

Supplement: Supplementary file 6 — Supplementary Material 6. OBO Foundry gallery [file 12864_2024_10759_MOESM6_ESM.zip › suppl6_OBOFoundry_gallery/image/OBOFoundry_ontoneo_heatmap.png]

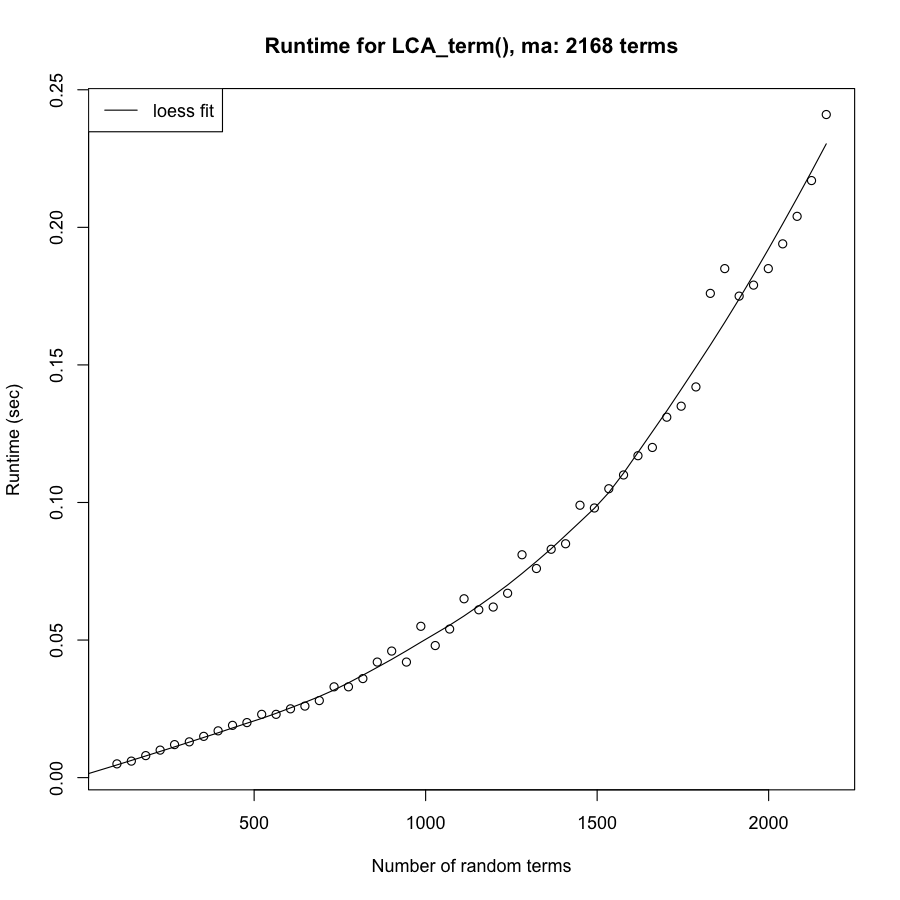

Supplement: Supplementary file 6 — Supplementary Material 6. OBO Foundry gallery [file 12864_2024_10759_MOESM6_ESM.zip › suppl6_OBOFoundry_gallery/image/OBOFoundry_ma_runtime.png]

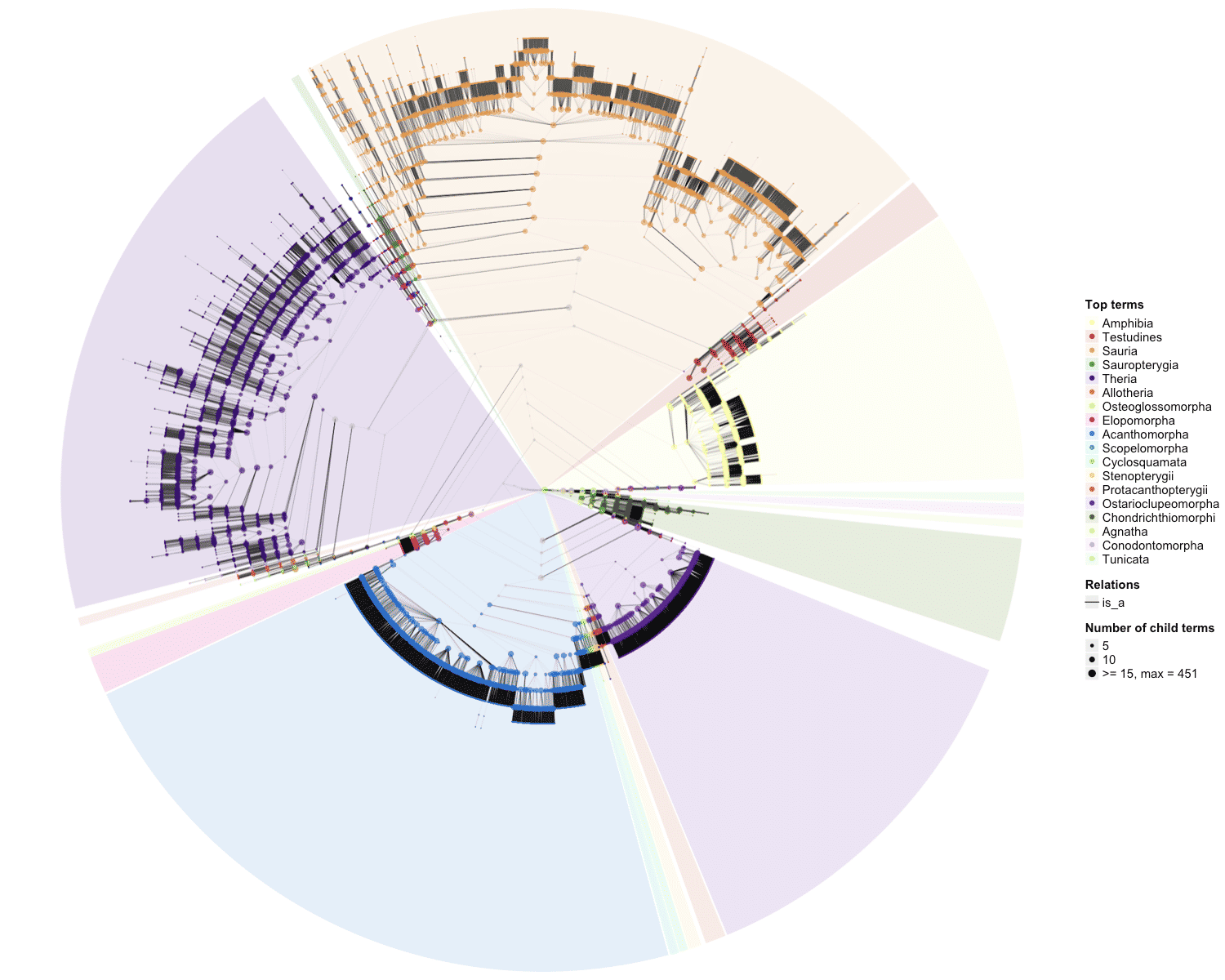

Supplement: Supplementary file 6 — Supplementary Material 6. OBO Foundry gallery [file 12864_2024_10759_MOESM6_ESM.zip › suppl6_OBOFoundry_gallery/image/OBOFoundry_vto.png]

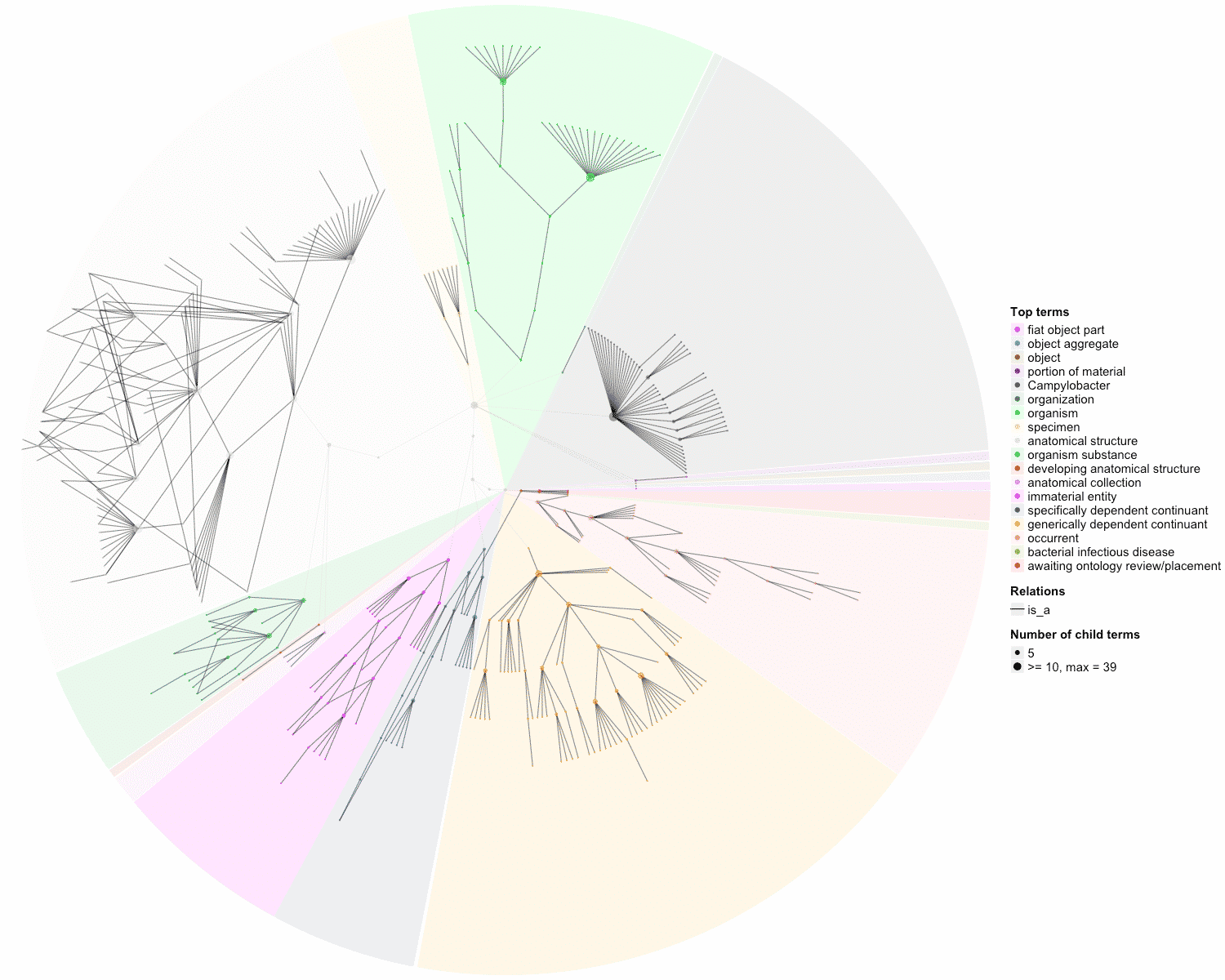

Supplement: Supplementary file 6 — Supplementary Material 6. OBO Foundry gallery [file 12864_2024_10759_MOESM6_ESM.zip › suppl6_OBOFoundry_gallery/image/OBOFoundry_hso.png]

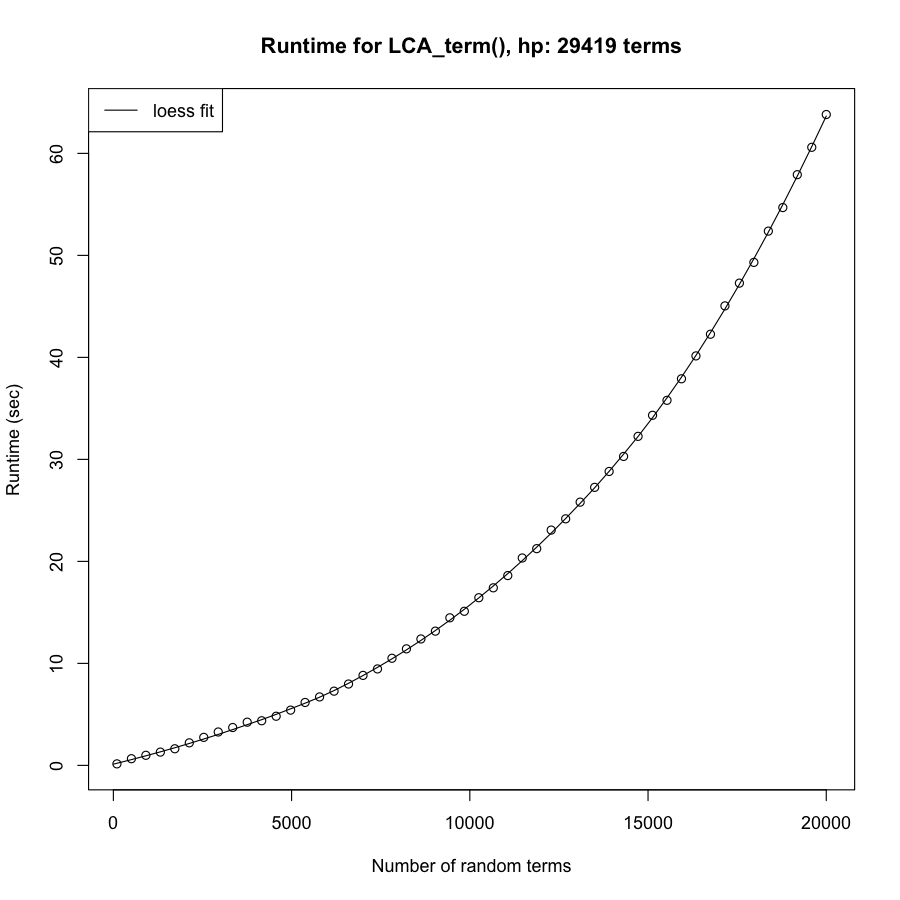

Supplement: Supplementary file 6 — Supplementary Material 6. OBO Foundry gallery [file 12864_2024_10759_MOESM6_ESM.zip › suppl6_OBOFoundry_gallery/image/OBOFoundry_hp_runtime.png]

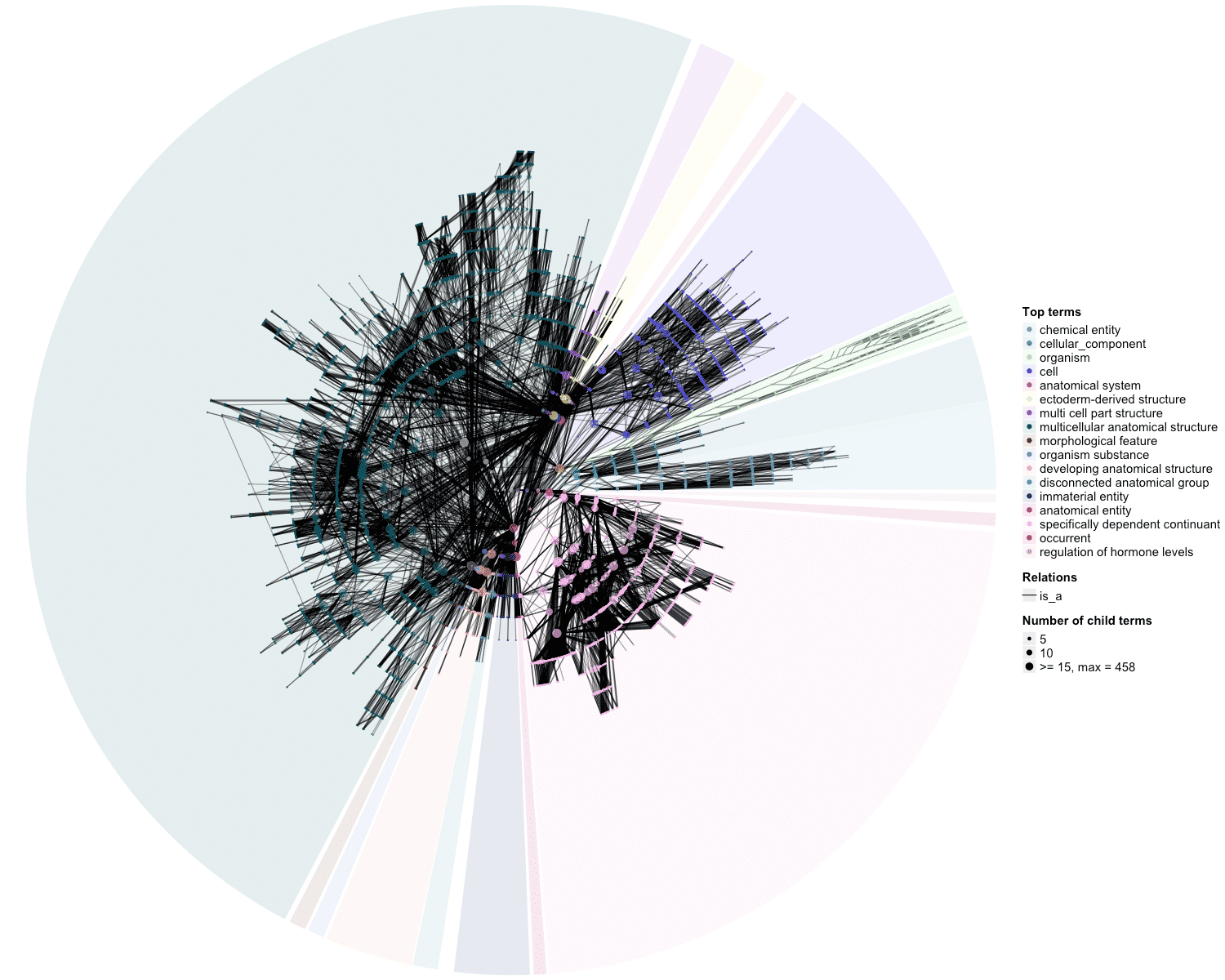

Supplement: Supplementary file 6 — Supplementary Material 6. OBO Foundry gallery [file 12864_2024_10759_MOESM6_ESM.zip › suppl6_OBOFoundry_gallery/image/OBOFoundry_fovt.png]

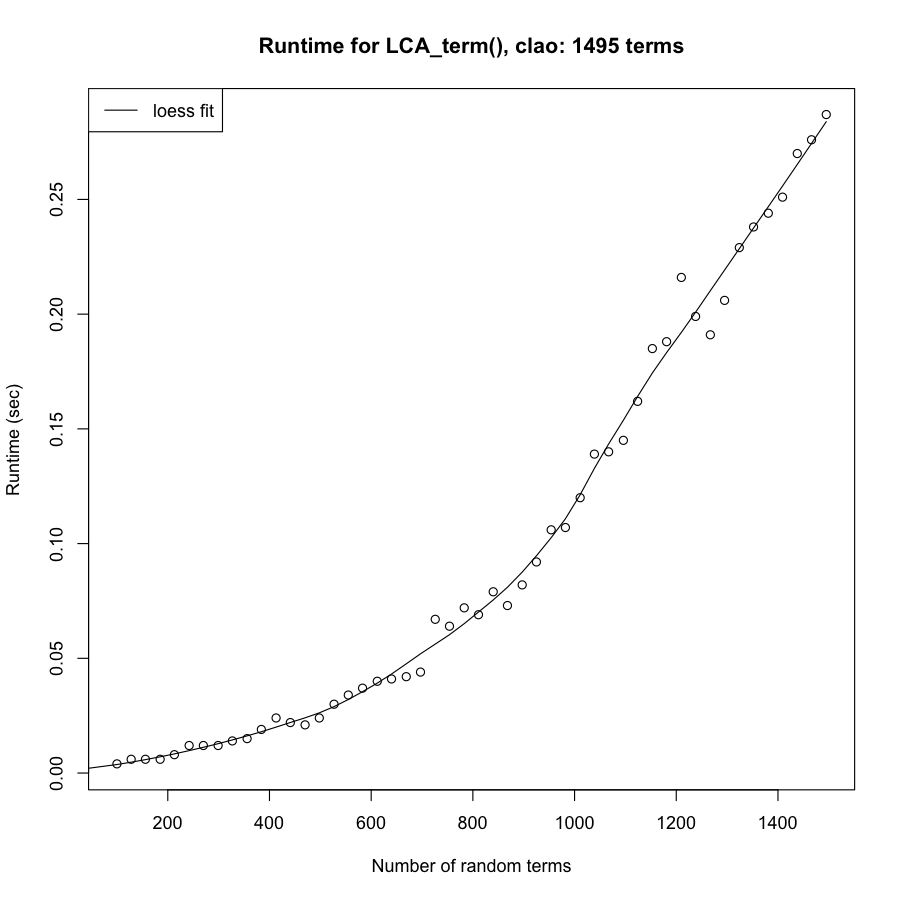

Supplement: Supplementary file 6 — Supplementary Material 6. OBO Foundry gallery [file 12864_2024_10759_MOESM6_ESM.zip › suppl6_OBOFoundry_gallery/image/OBOFoundry_clao_runtime.png]

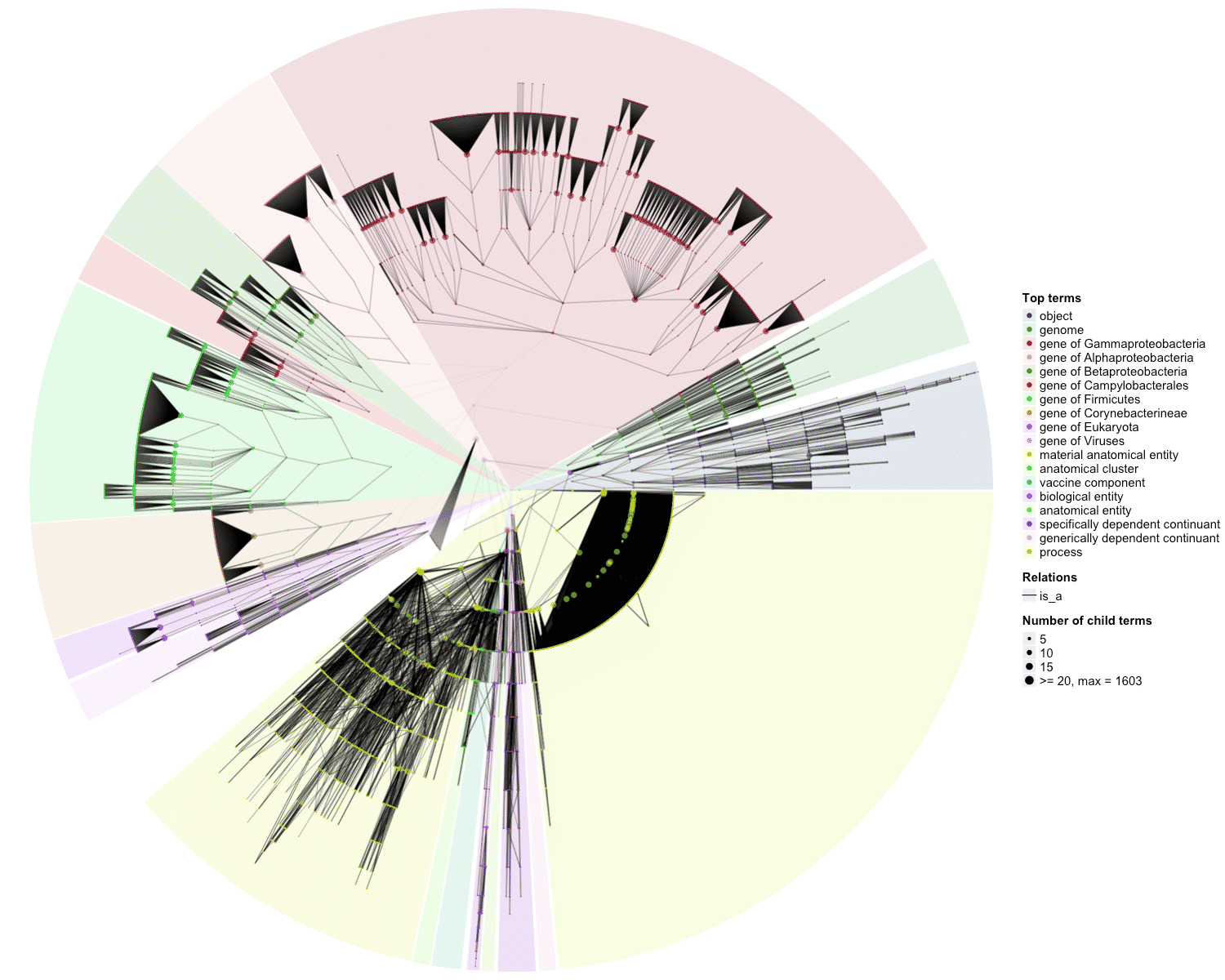

Supplement: Supplementary file 6 — Supplementary Material 6. OBO Foundry gallery [file 12864_2024_10759_MOESM6_ESM.zip › suppl6_OBOFoundry_gallery/image/OBOFoundry_ohpi.png]

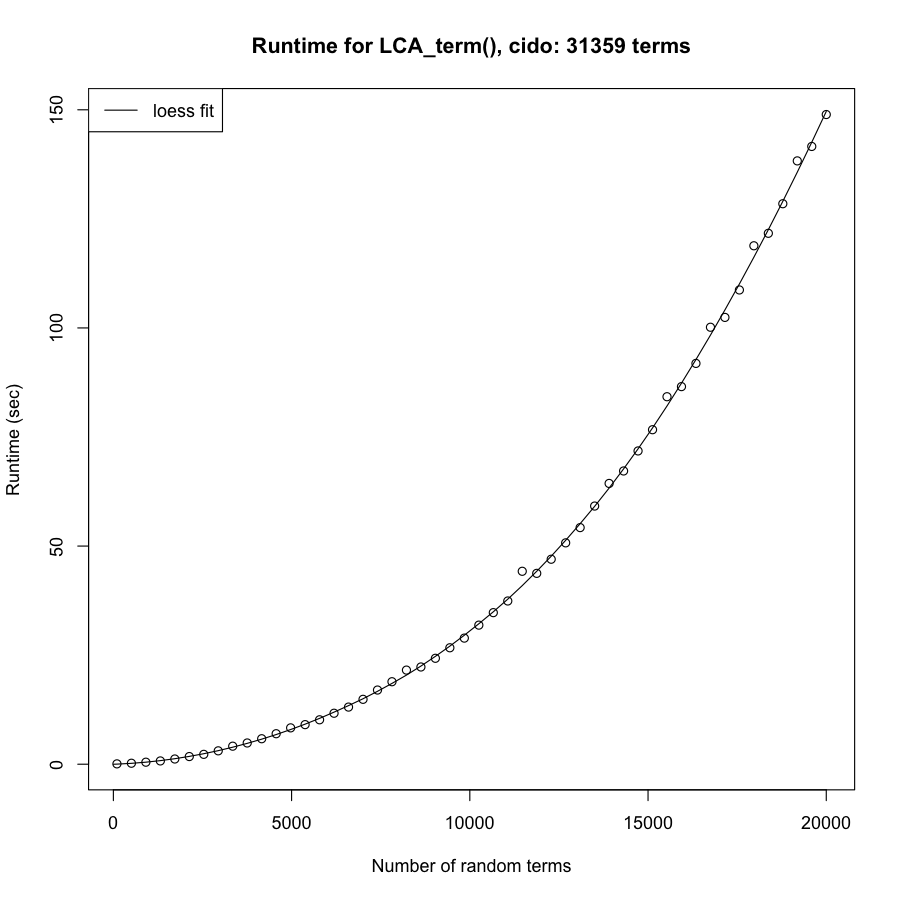

Supplement: Supplementary file 6 — Supplementary Material 6. OBO Foundry gallery [file 12864_2024_10759_MOESM6_ESM.zip › suppl6_OBOFoundry_gallery/image/OBOFoundry_cido_runtime.png]

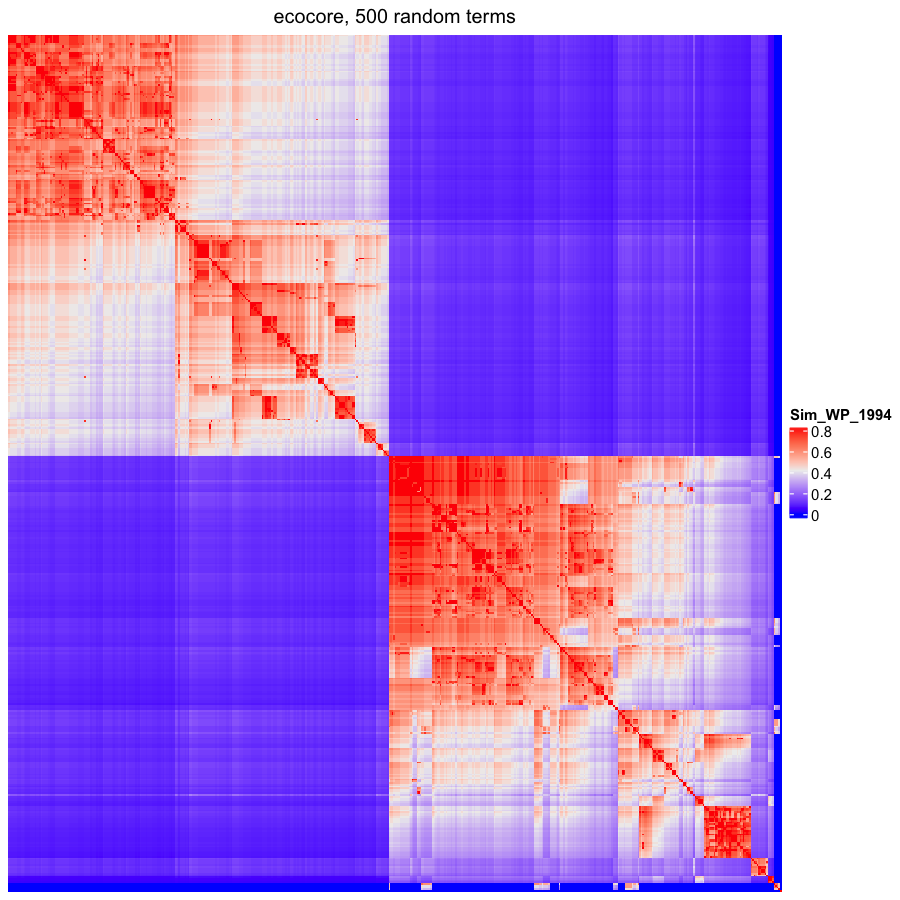

Supplement: Supplementary file 6 — Supplementary Material 6. OBO Foundry gallery [file 12864_2024_10759_MOESM6_ESM.zip › suppl6_OBOFoundry_gallery/image/OBOFoundry_ecocore_heatmap.png]

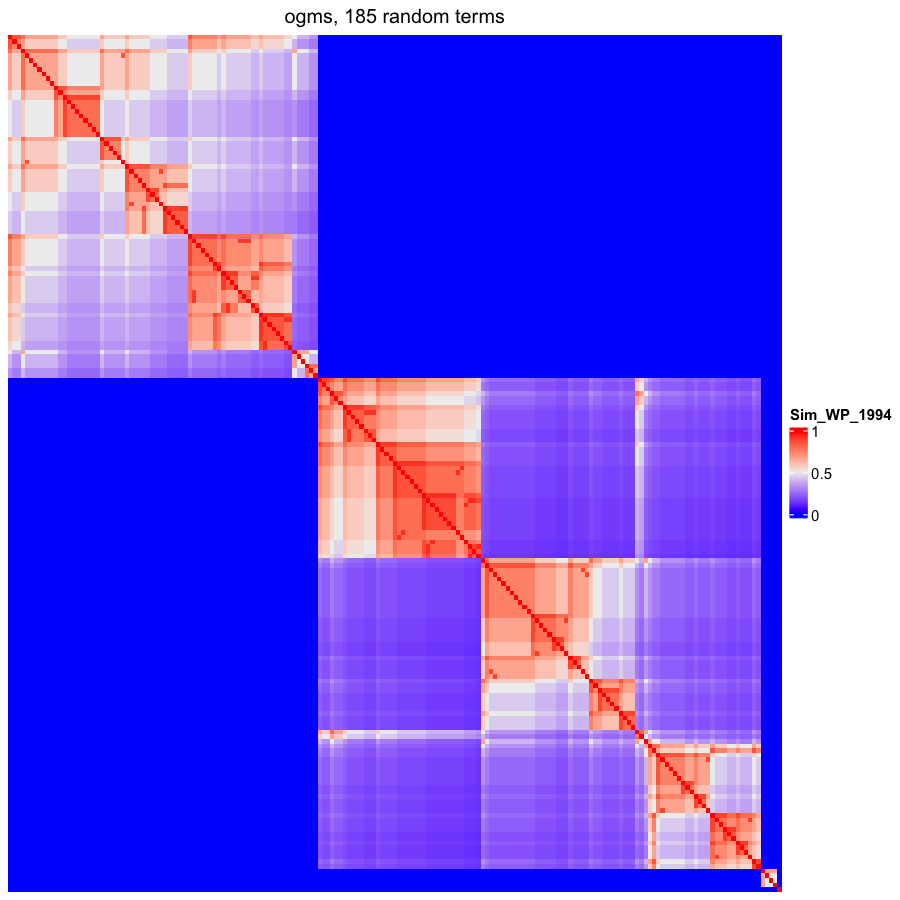

Supplement: Supplementary file 6 — Supplementary Material 6. OBO Foundry gallery [file 12864_2024_10759_MOESM6_ESM.zip › suppl6_OBOFoundry_gallery/image/OBOFoundry_ogms_heatmap.png]

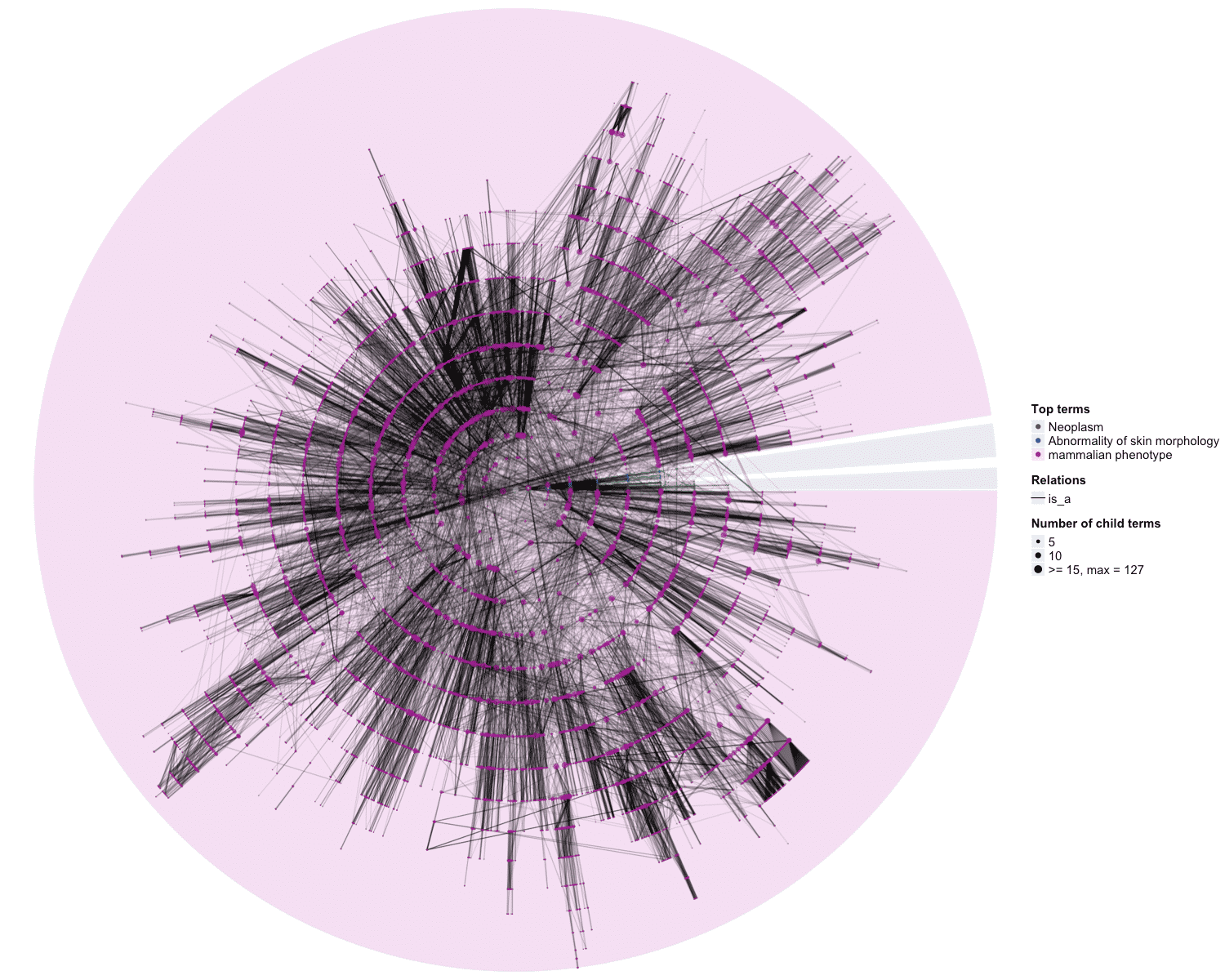

Supplement: Supplementary file 6 — Supplementary Material 6. OBO Foundry gallery [file 12864_2024_10759_MOESM6_ESM.zip › suppl6_OBOFoundry_gallery/image/OBOFoundry_upheno.png]

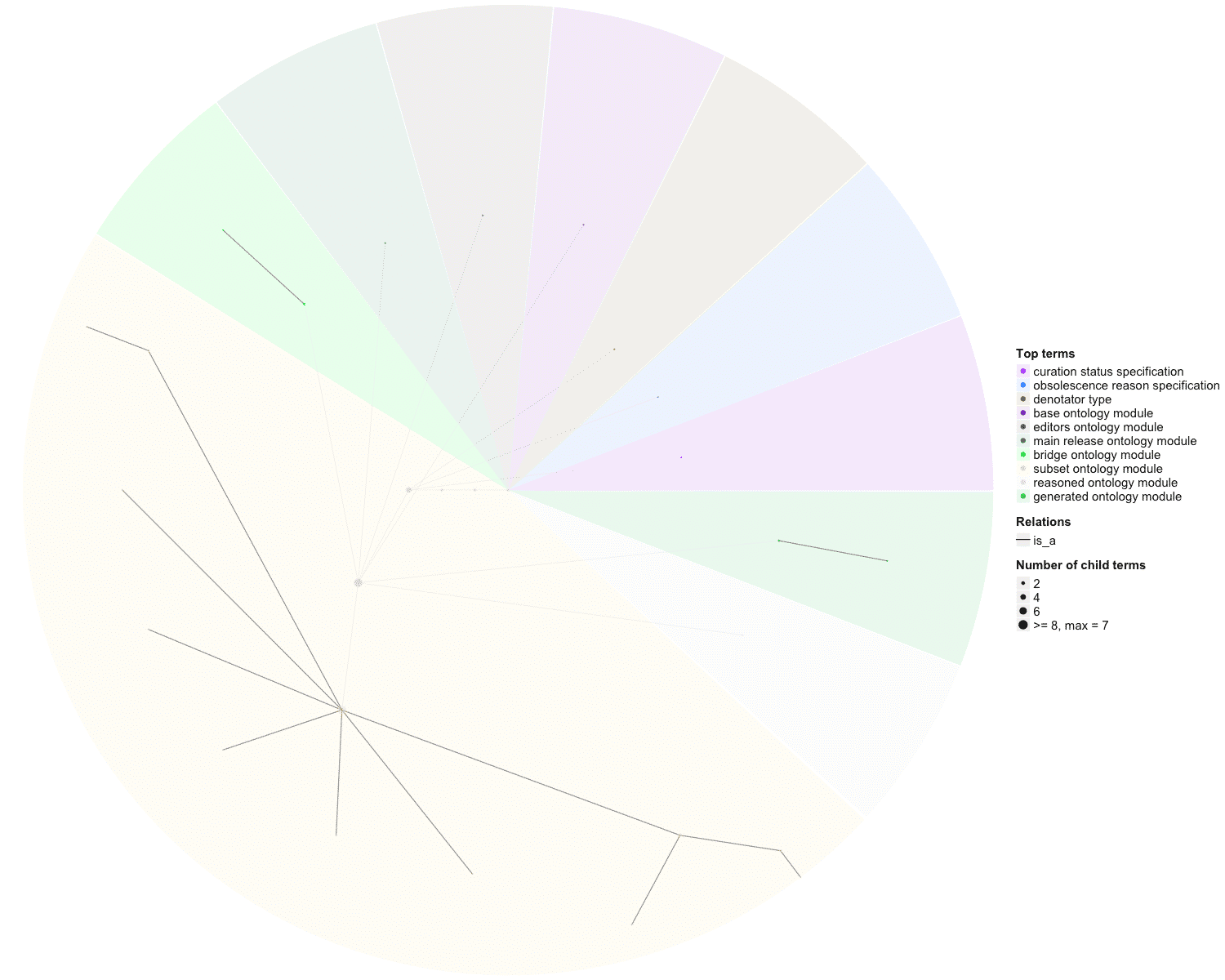

Supplement: Supplementary file 6 — Supplementary Material 6. OBO Foundry gallery [file 12864_2024_10759_MOESM6_ESM.zip › suppl6_OBOFoundry_gallery/image/OBOFoundry_omo.png]

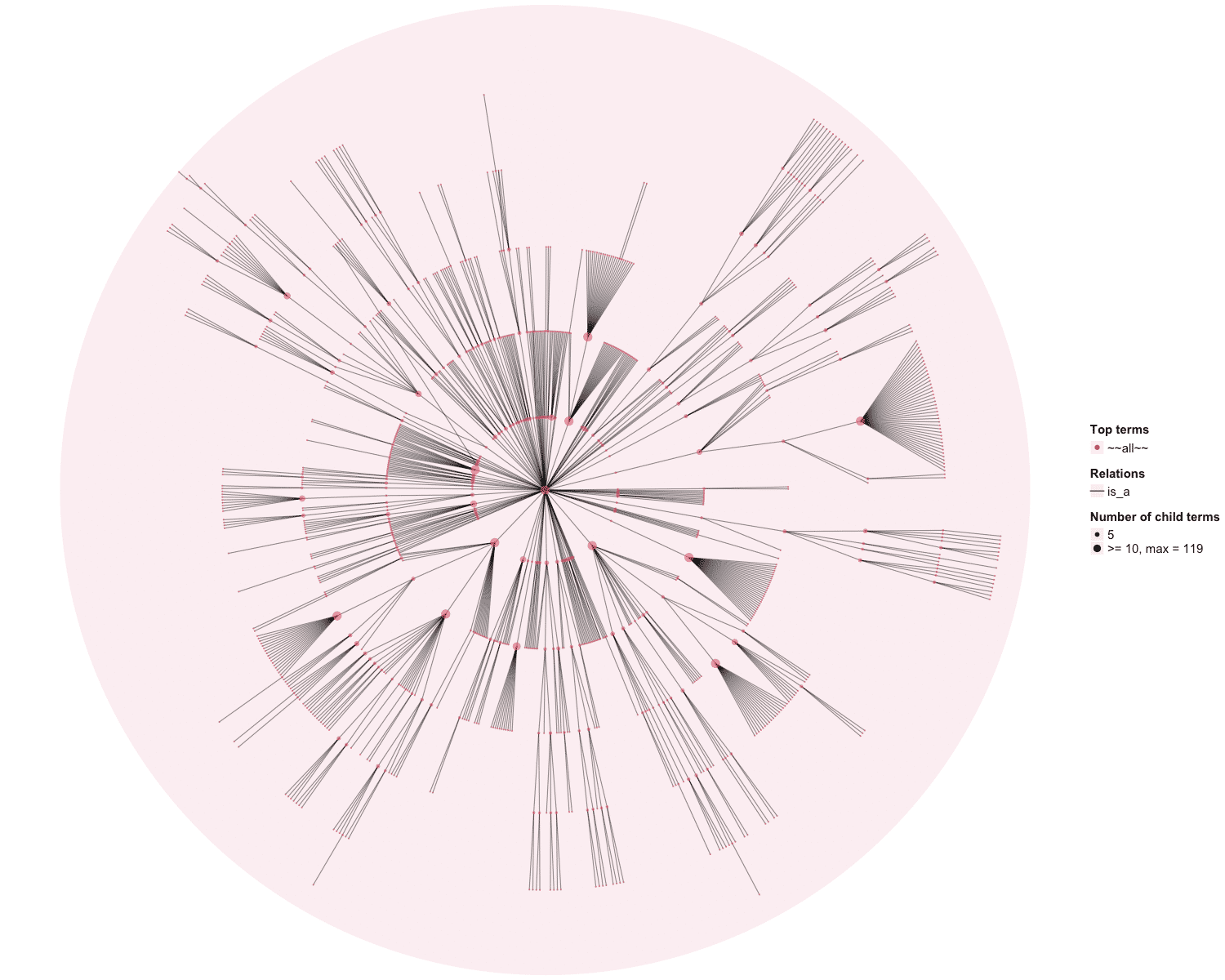

Supplement: Supplementary file 6 — Supplementary Material 6. OBO Foundry gallery [file 12864_2024_10759_MOESM6_ESM.zip › suppl6_OBOFoundry_gallery/image/OBOFoundry_micro.png]

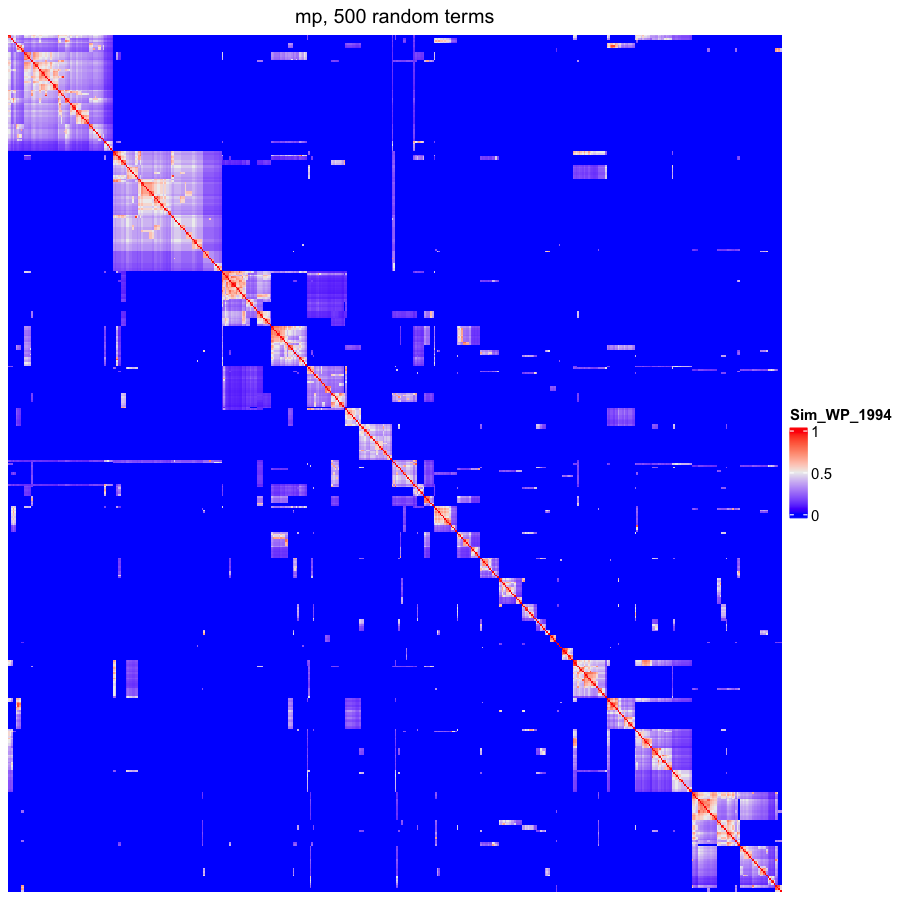

Supplement: Supplementary file 6 — Supplementary Material 6. OBO Foundry gallery [file 12864_2024_10759_MOESM6_ESM.zip › suppl6_OBOFoundry_gallery/image/OBOFoundry_mp_heatmap.png]

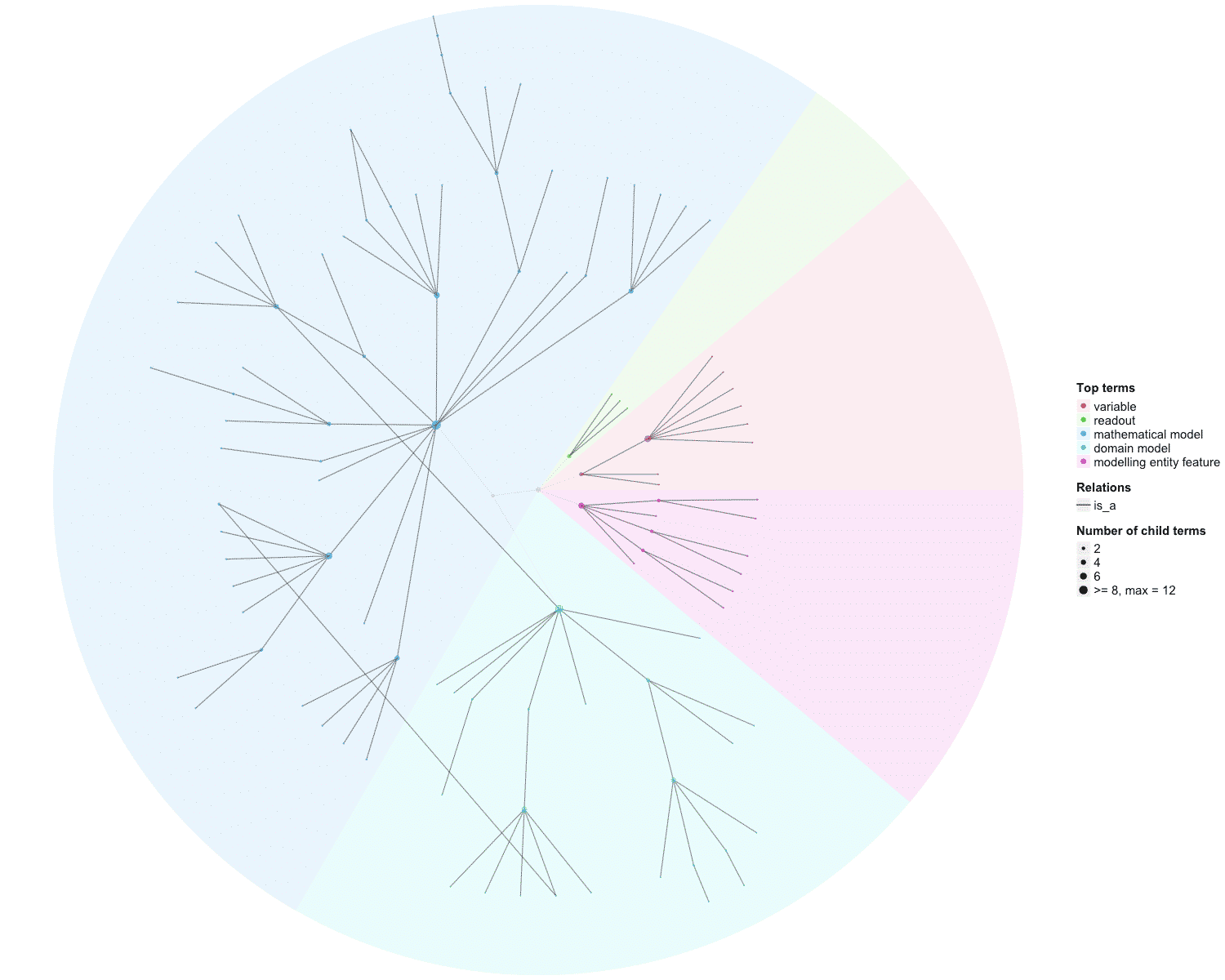

Supplement: Supplementary file 6 — Supplementary Material 6. OBO Foundry gallery [file 12864_2024_10759_MOESM6_ESM.zip › suppl6_OBOFoundry_gallery/image/OBOFoundry_mamo.png]

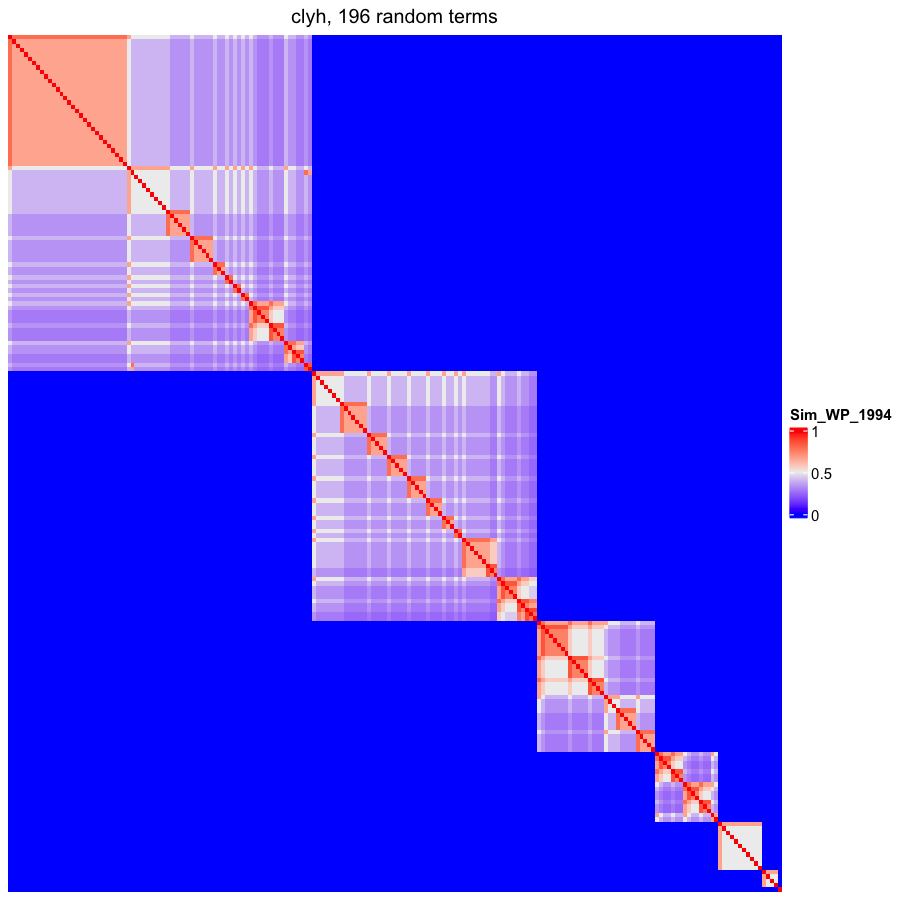

Supplement: Supplementary file 6 — Supplementary Material 6. OBO Foundry gallery [file 12864_2024_10759_MOESM6_ESM.zip › suppl6_OBOFoundry_gallery/image/OBOFoundry_clyh_heatmap.png]

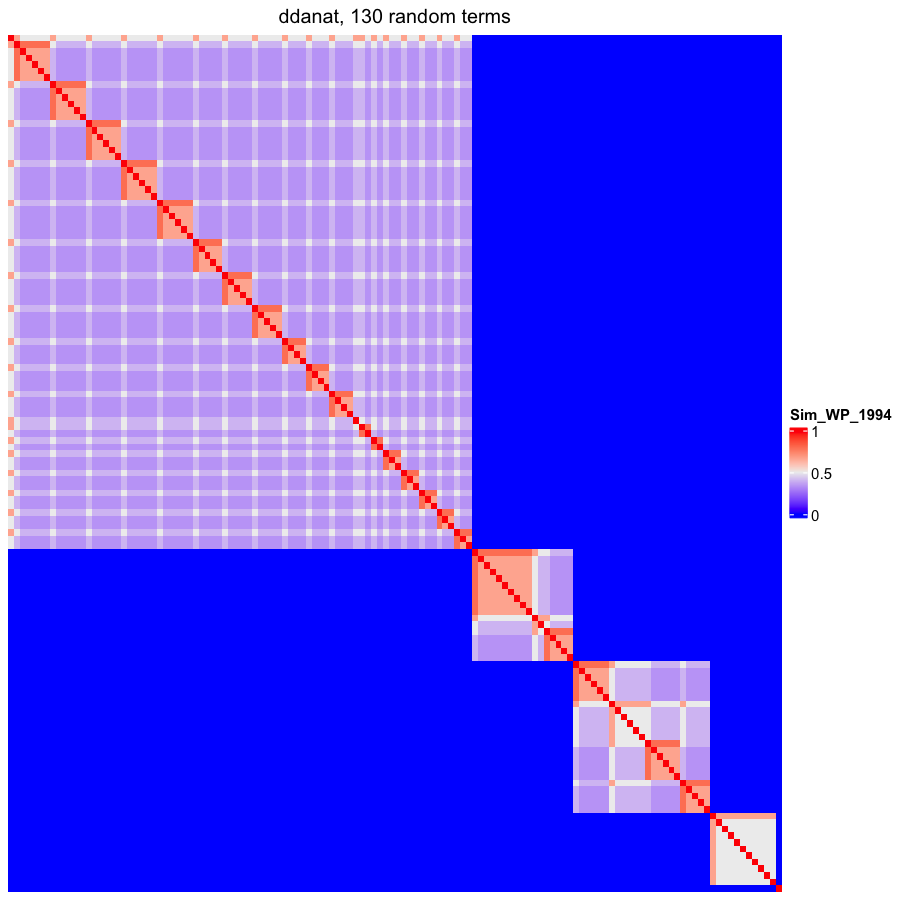

Supplement: Supplementary file 6 — Supplementary Material 6. OBO Foundry gallery [file 12864_2024_10759_MOESM6_ESM.zip › suppl6_OBOFoundry_gallery/image/OBOFoundry_ddanat_heatmap.png]

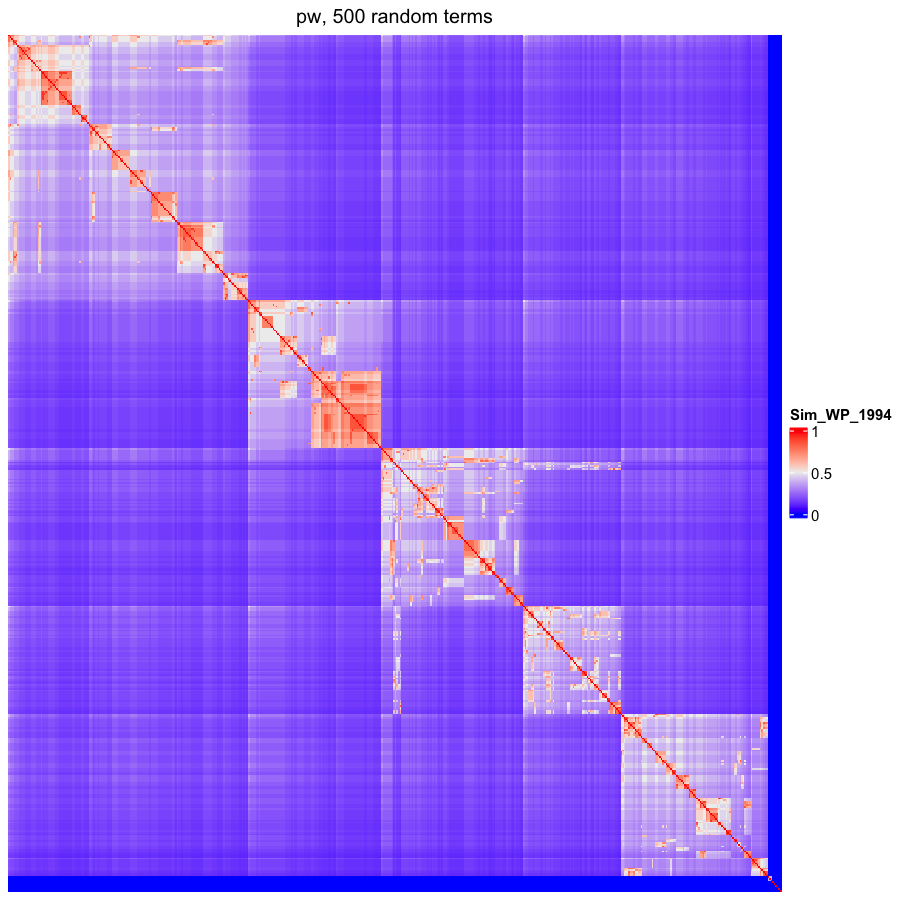

Supplement: Supplementary file 6 — Supplementary Material 6. OBO Foundry gallery [file 12864_2024_10759_MOESM6_ESM.zip › suppl6_OBOFoundry_gallery/image/OBOFoundry_pw_heatmap.png]

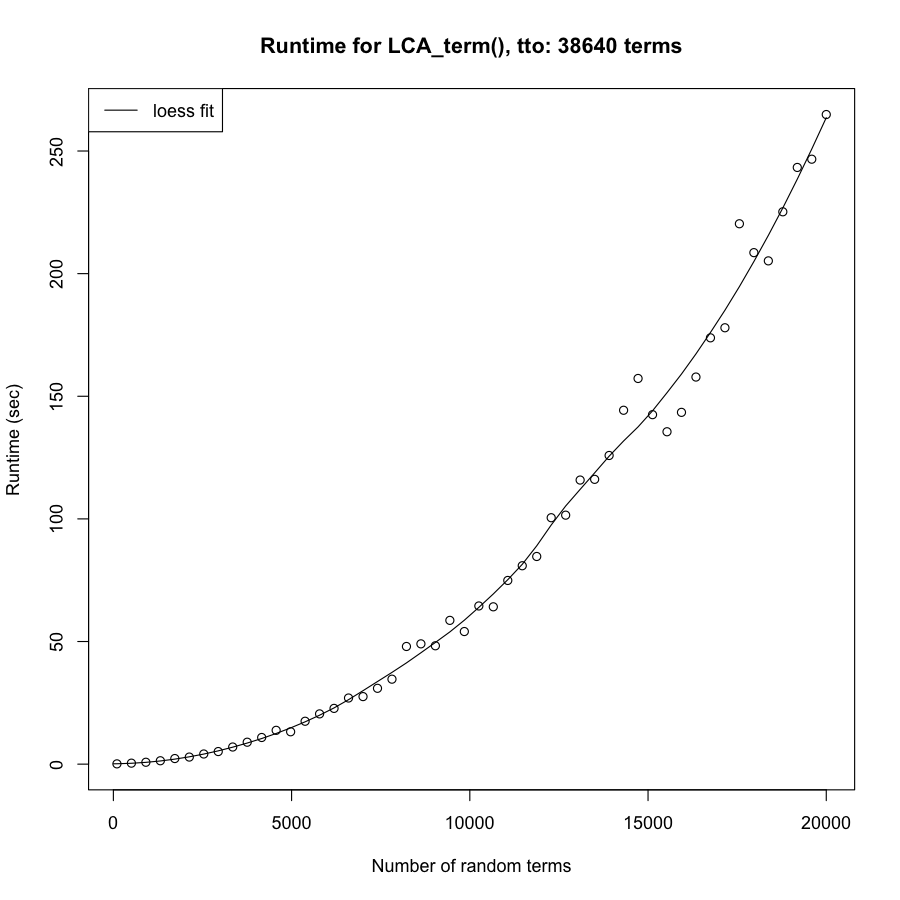

Supplement: Supplementary file 6 — Supplementary Material 6. OBO Foundry gallery [file 12864_2024_10759_MOESM6_ESM.zip › suppl6_OBOFoundry_gallery/image/OBOFoundry_tto_runtime.png]

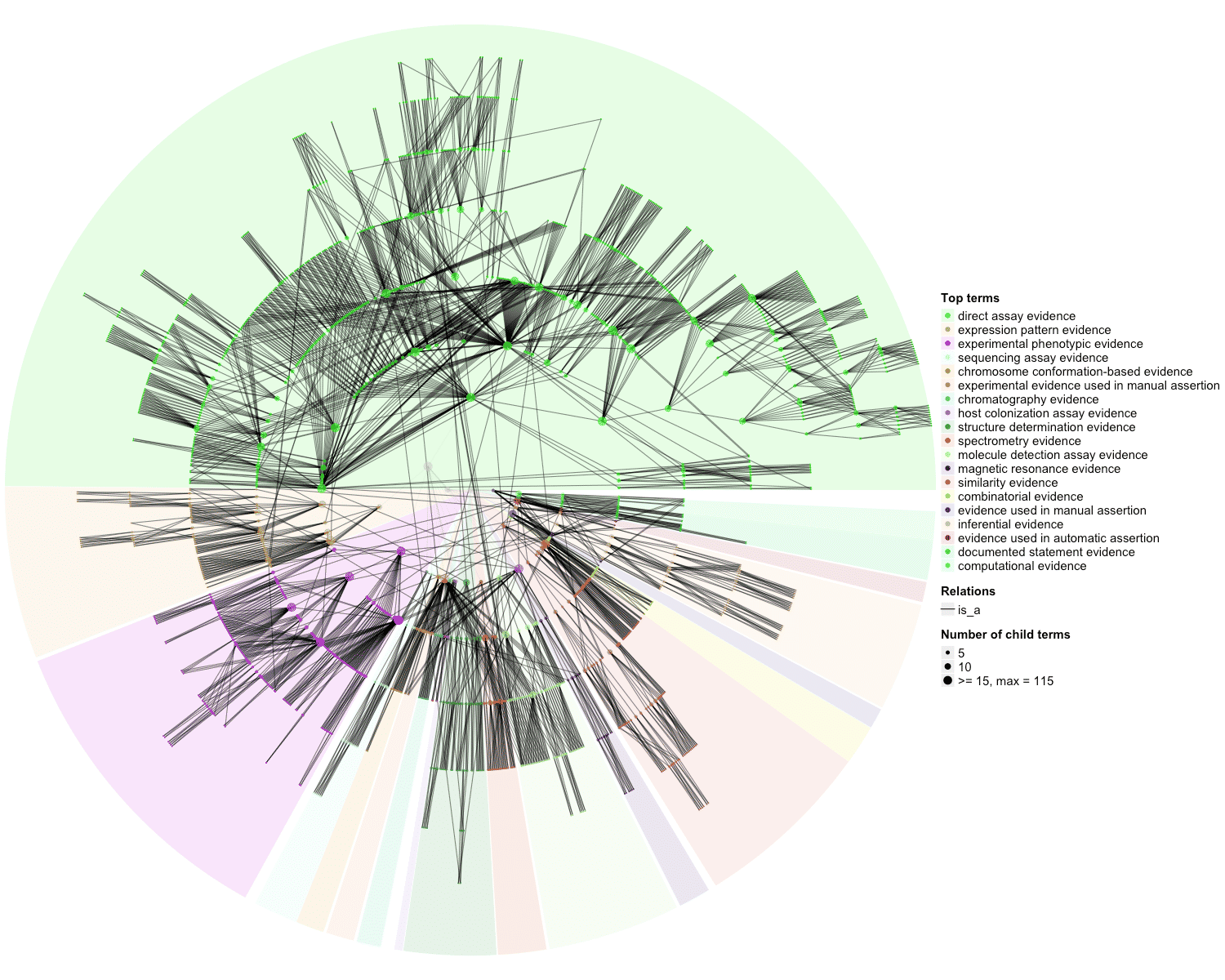

Supplement: Supplementary file 6 — Supplementary Material 6. OBO Foundry gallery [file 12864_2024_10759_MOESM6_ESM.zip › suppl6_OBOFoundry_gallery/image/OBOFoundry_eco.png]

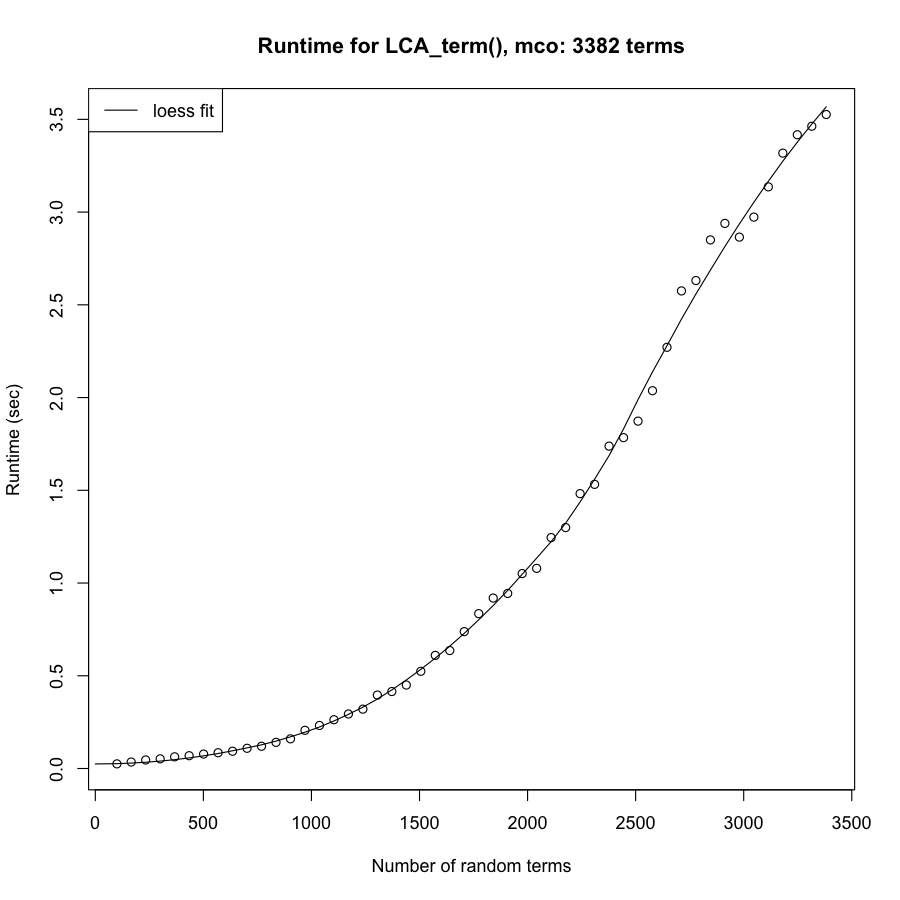

Supplement: Supplementary file 6 — Supplementary Material 6. OBO Foundry gallery [file 12864_2024_10759_MOESM6_ESM.zip › suppl6_OBOFoundry_gallery/image/OBOFoundry_mco_runtime.png]

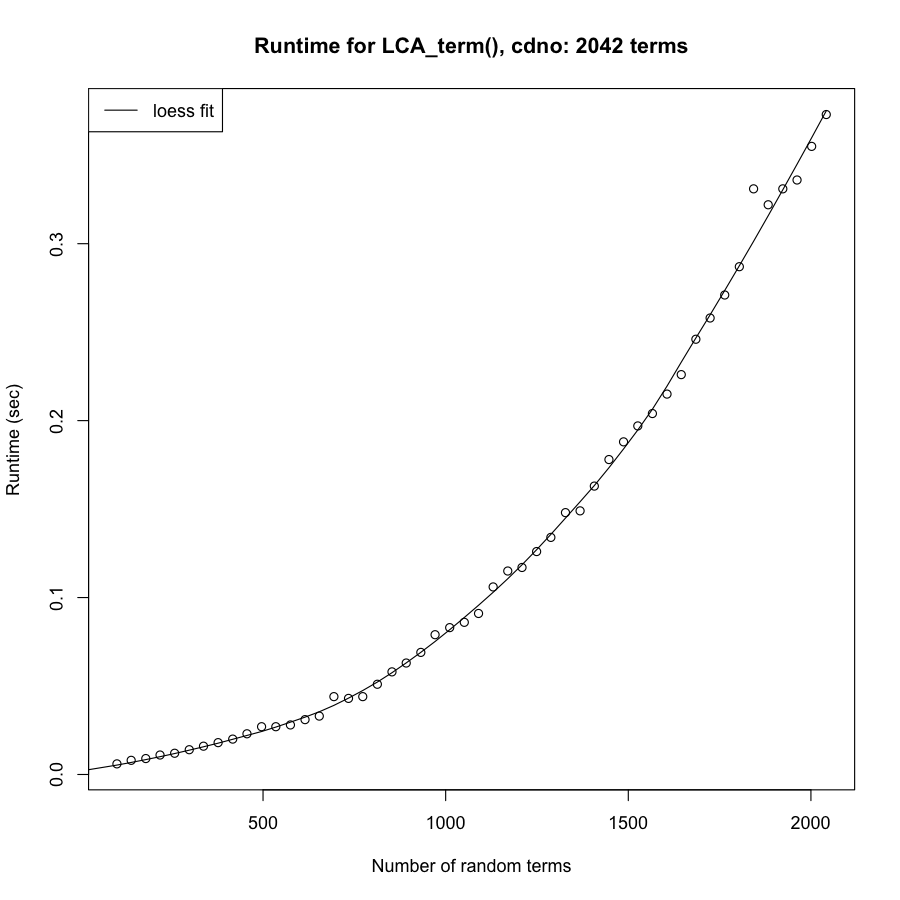

Supplement: Supplementary file 6 — Supplementary Material 6. OBO Foundry gallery [file 12864_2024_10759_MOESM6_ESM.zip › suppl6_OBOFoundry_gallery/image/OBOFoundry_cdno_runtime.png]

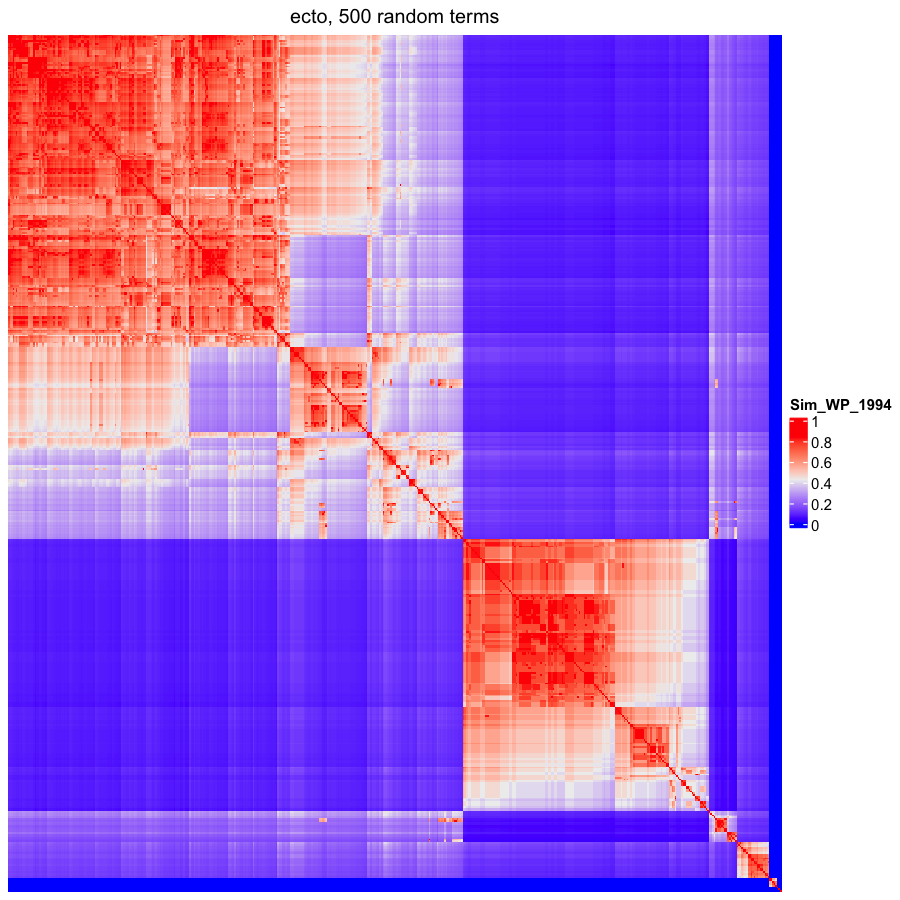

Supplement: Supplementary file 6 — Supplementary Material 6. OBO Foundry gallery [file 12864_2024_10759_MOESM6_ESM.zip › suppl6_OBOFoundry_gallery/image/OBOFoundry_ecto_heatmap.png]

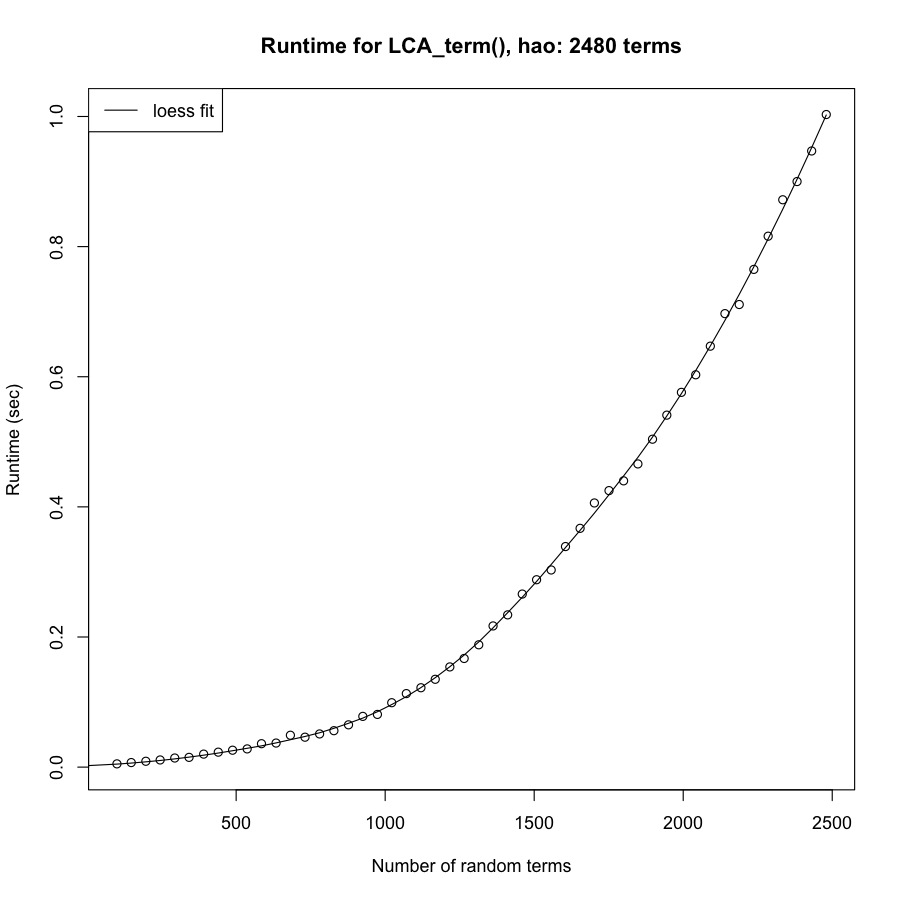

Supplement: Supplementary file 6 — Supplementary Material 6. OBO Foundry gallery [file 12864_2024_10759_MOESM6_ESM.zip › suppl6_OBOFoundry_gallery/image/OBOFoundry_hao_runtime.png]

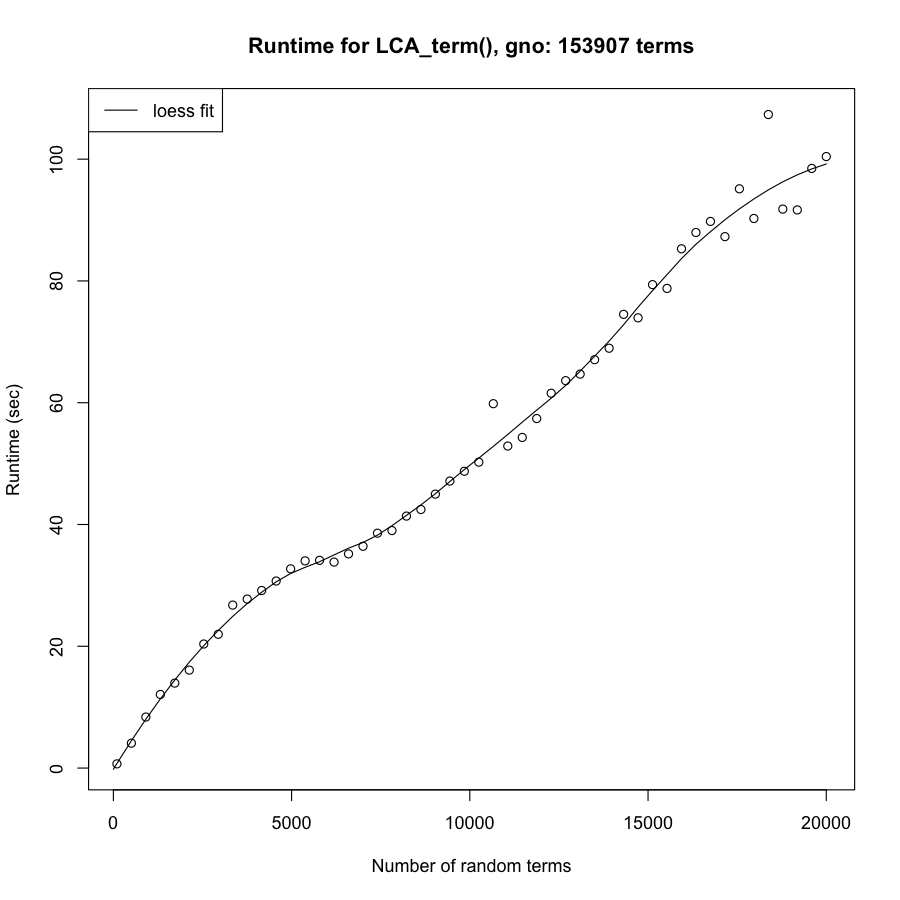

Supplement: Supplementary file 6 — Supplementary Material 6. OBO Foundry gallery [file 12864_2024_10759_MOESM6_ESM.zip › suppl6_OBOFoundry_gallery/image/OBOFoundry_gno_runtime.png]

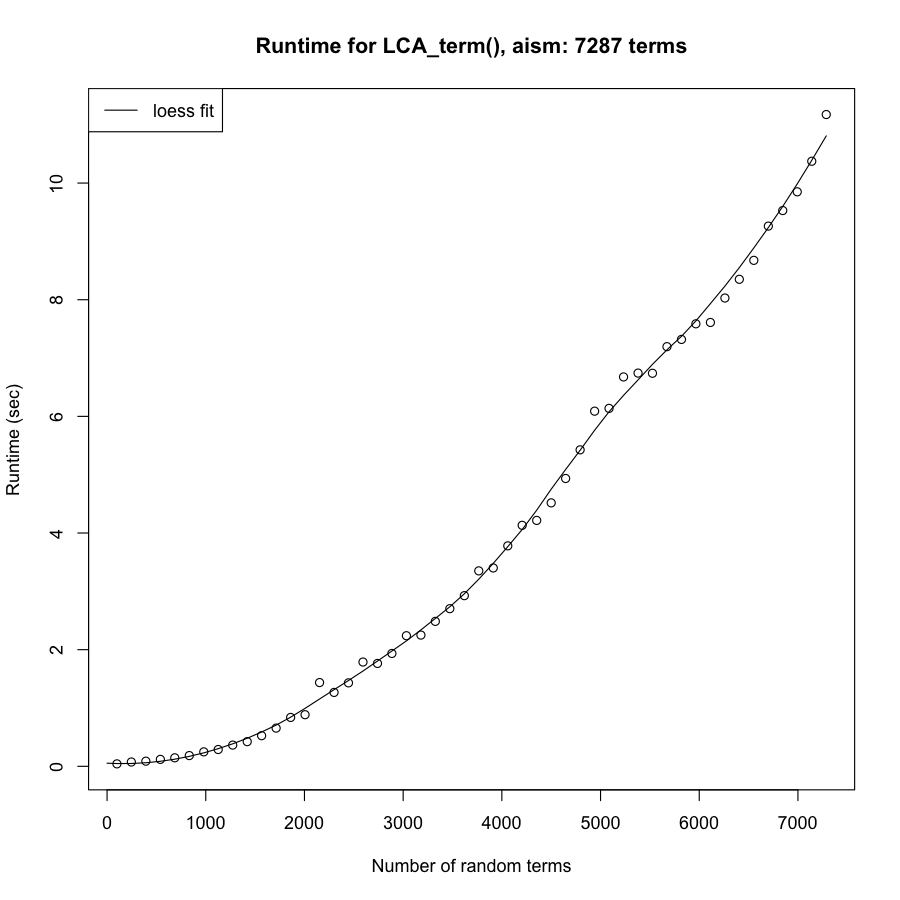

Supplement: Supplementary file 6 — Supplementary Material 6. OBO Foundry gallery [file 12864_2024_10759_MOESM6_ESM.zip › suppl6_OBOFoundry_gallery/image/OBOFoundry_aism_runtime.png]

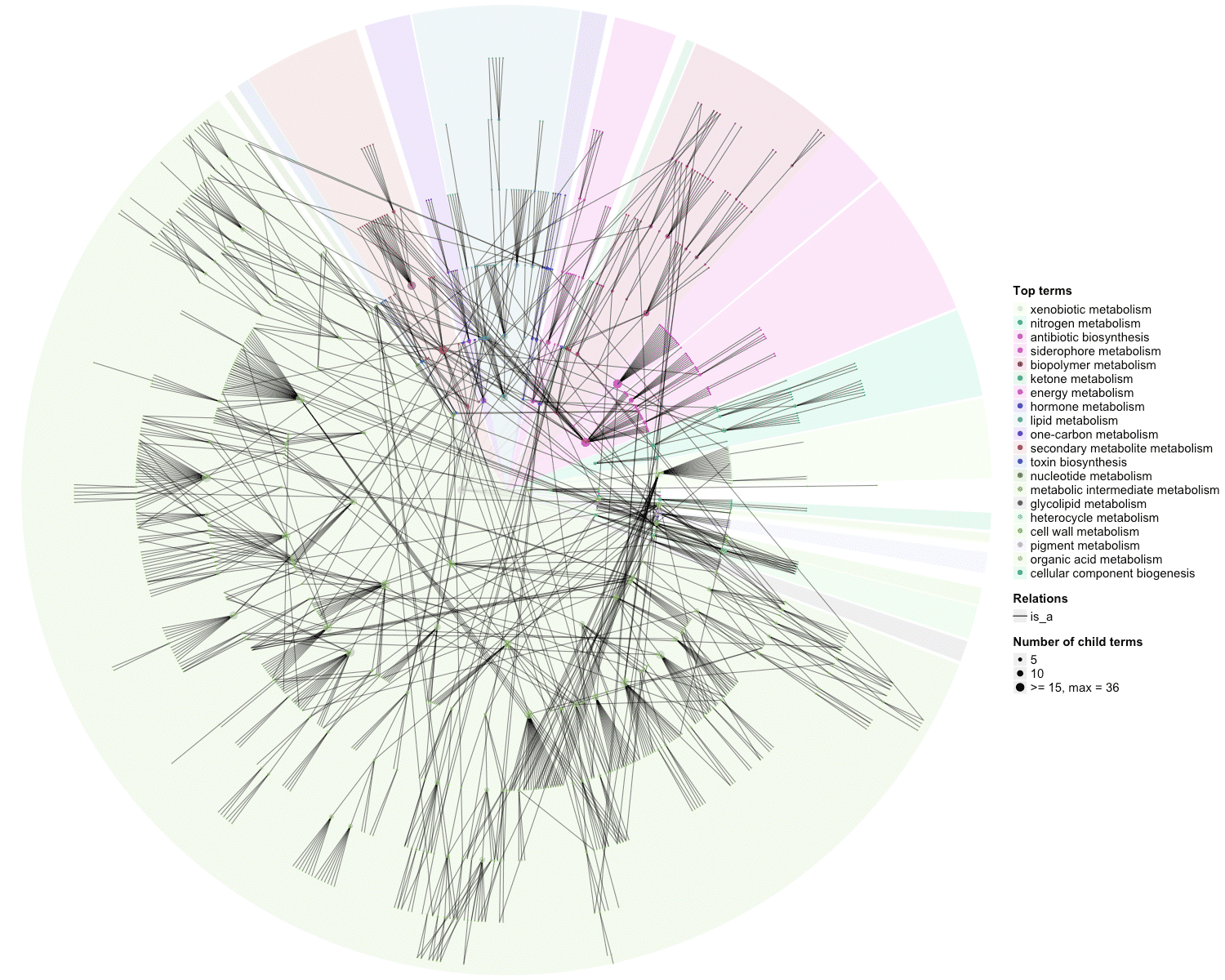

Supplement: Supplementary file 6 — Supplementary Material 6. OBO Foundry gallery [file 12864_2024_10759_MOESM6_ESM.zip › suppl6_OBOFoundry_gallery/image/OBOFoundry_upa.png]

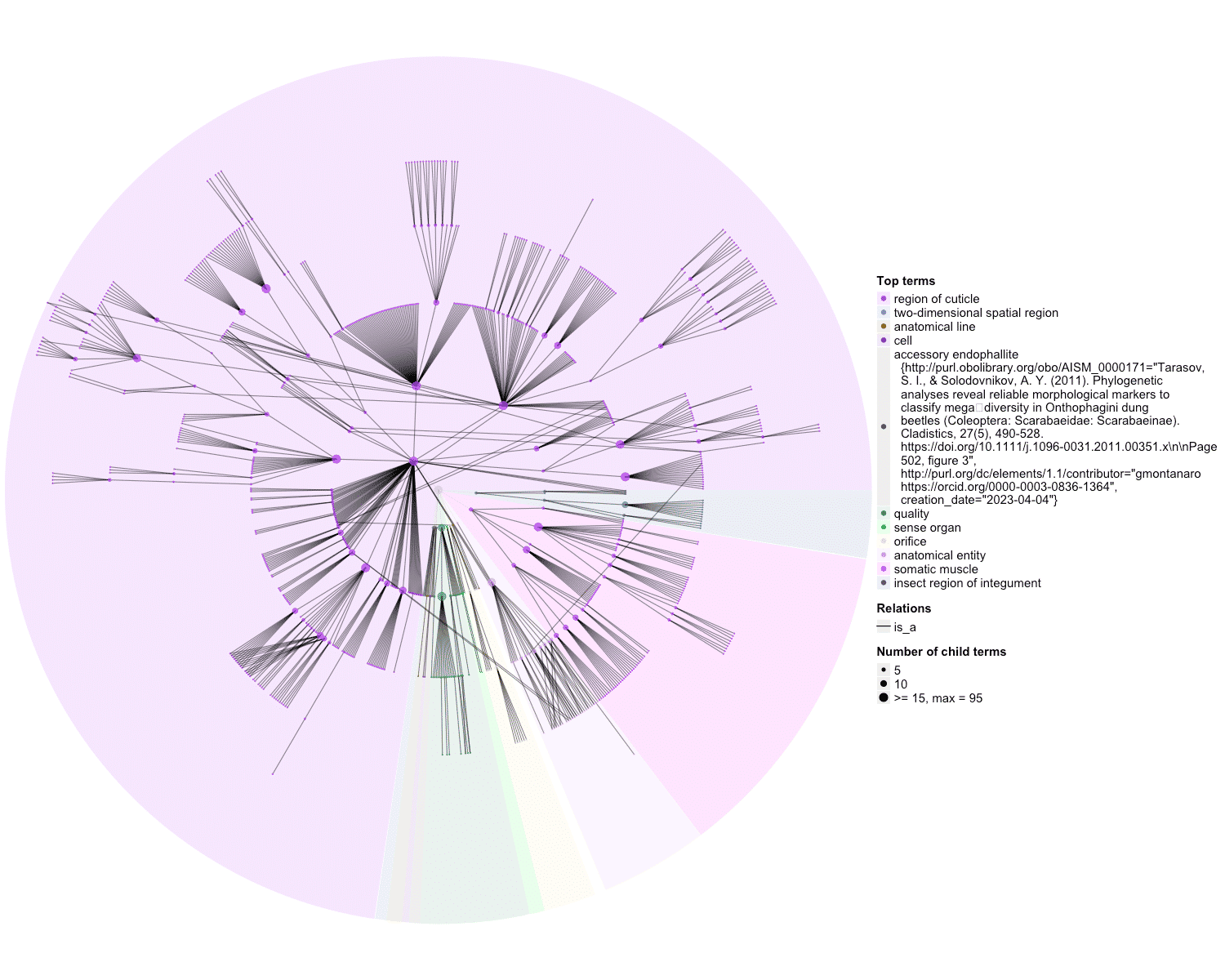

Supplement: Supplementary file 6 — Supplementary Material 6. OBO Foundry gallery [file 12864_2024_10759_MOESM6_ESM.zip › suppl6_OBOFoundry_gallery/image/OBOFoundry_colao.png]

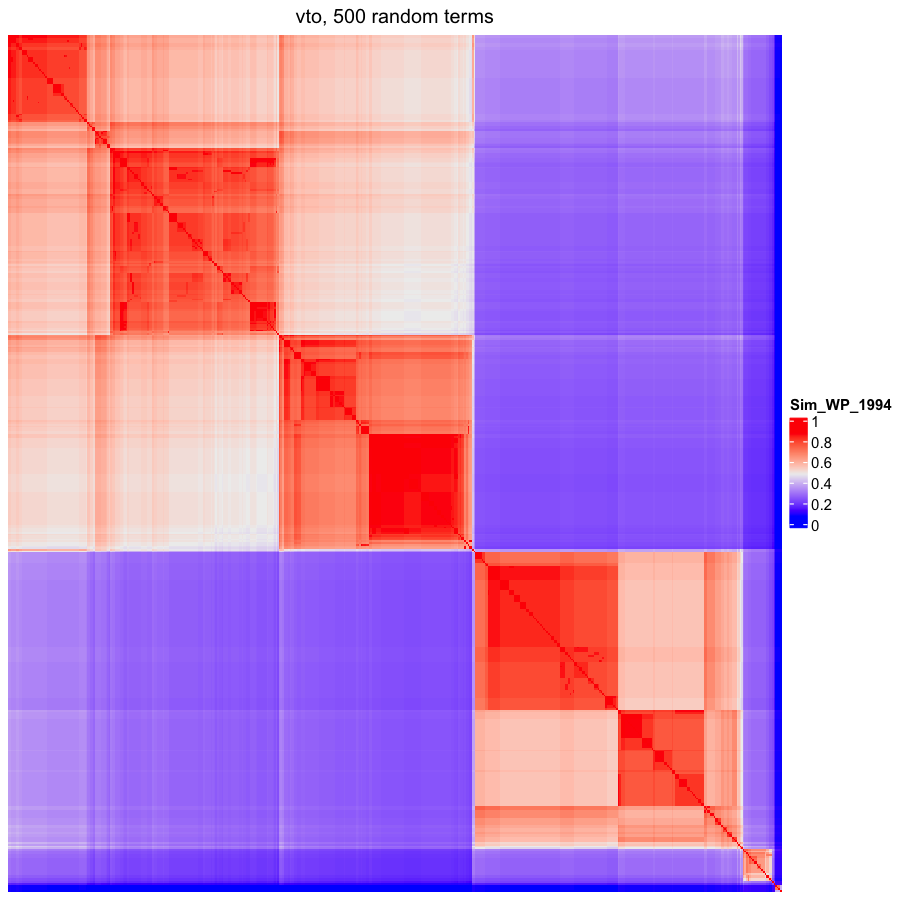

Supplement: Supplementary file 6 — Supplementary Material 6. OBO Foundry gallery [file 12864_2024_10759_MOESM6_ESM.zip › suppl6_OBOFoundry_gallery/image/OBOFoundry_vto_heatmap.png]

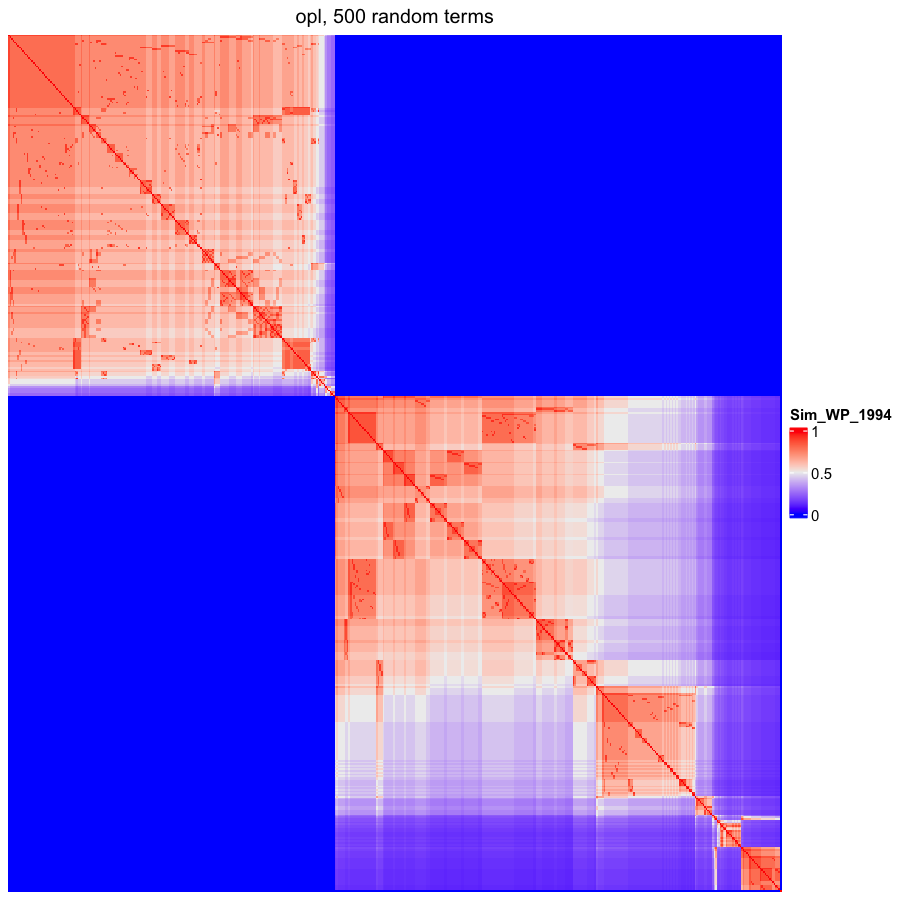

Supplement: Supplementary file 6 — Supplementary Material 6. OBO Foundry gallery [file 12864_2024_10759_MOESM6_ESM.zip › suppl6_OBOFoundry_gallery/image/OBOFoundry_opl_heatmap.png]

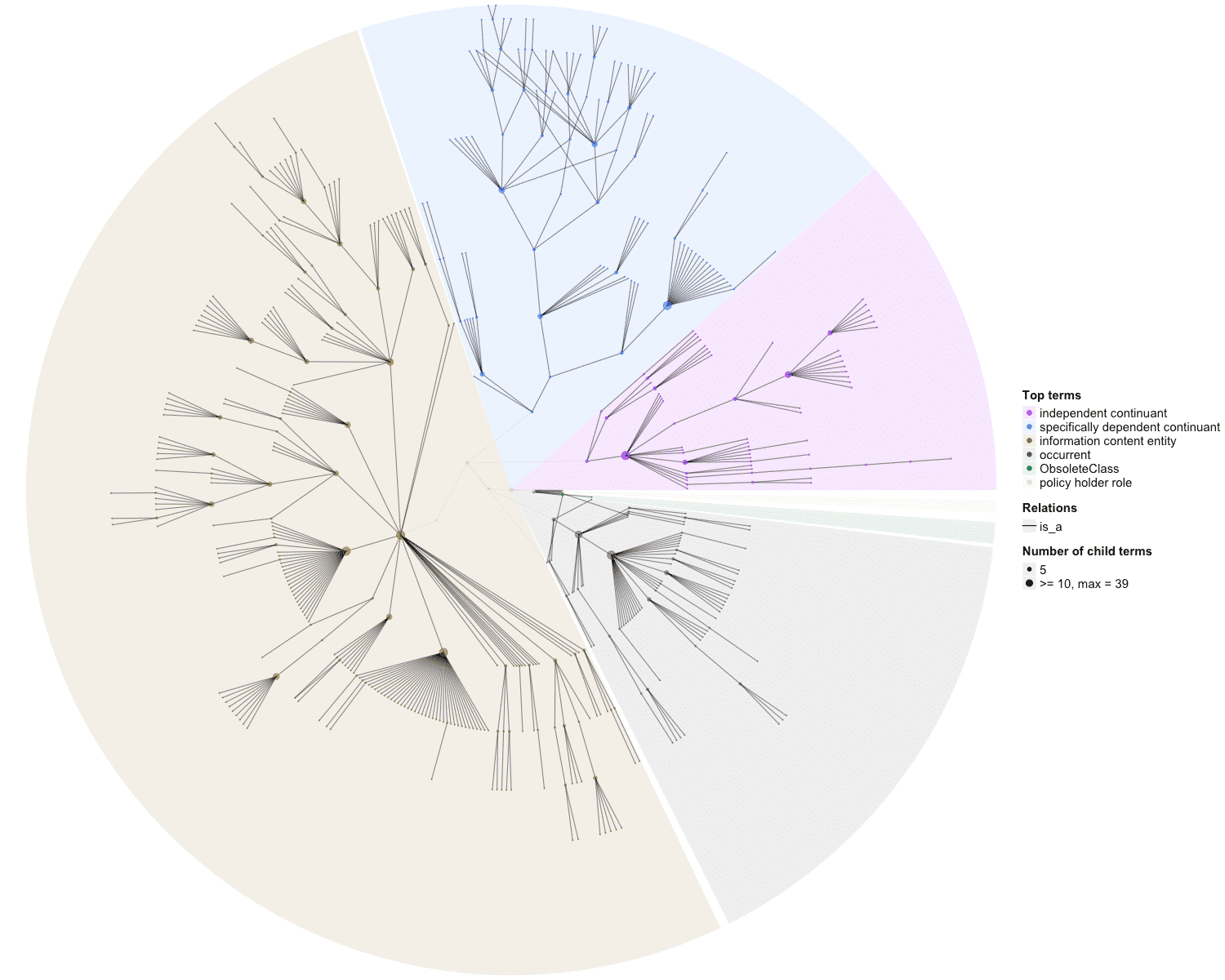

Supplement: Supplementary file 6 — Supplementary Material 6. OBO Foundry gallery [file 12864_2024_10759_MOESM6_ESM.zip › suppl6_OBOFoundry_gallery/image/OBOFoundry_omrse.png]

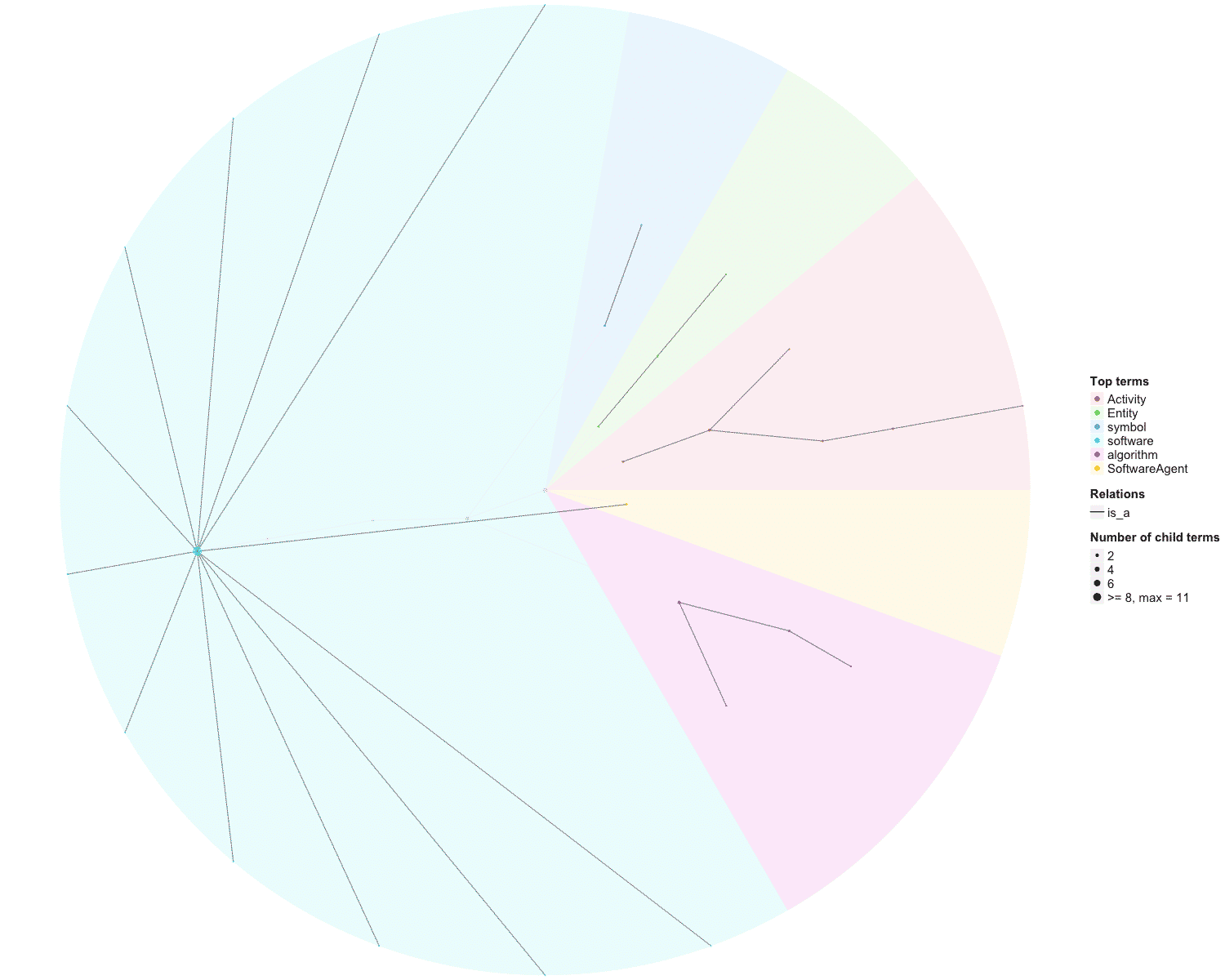

Supplement: Supplementary file 6 — Supplementary Material 6. OBO Foundry gallery [file 12864_2024_10759_MOESM6_ESM.zip › suppl6_OBOFoundry_gallery/image/OBOFoundry_miapa.png]

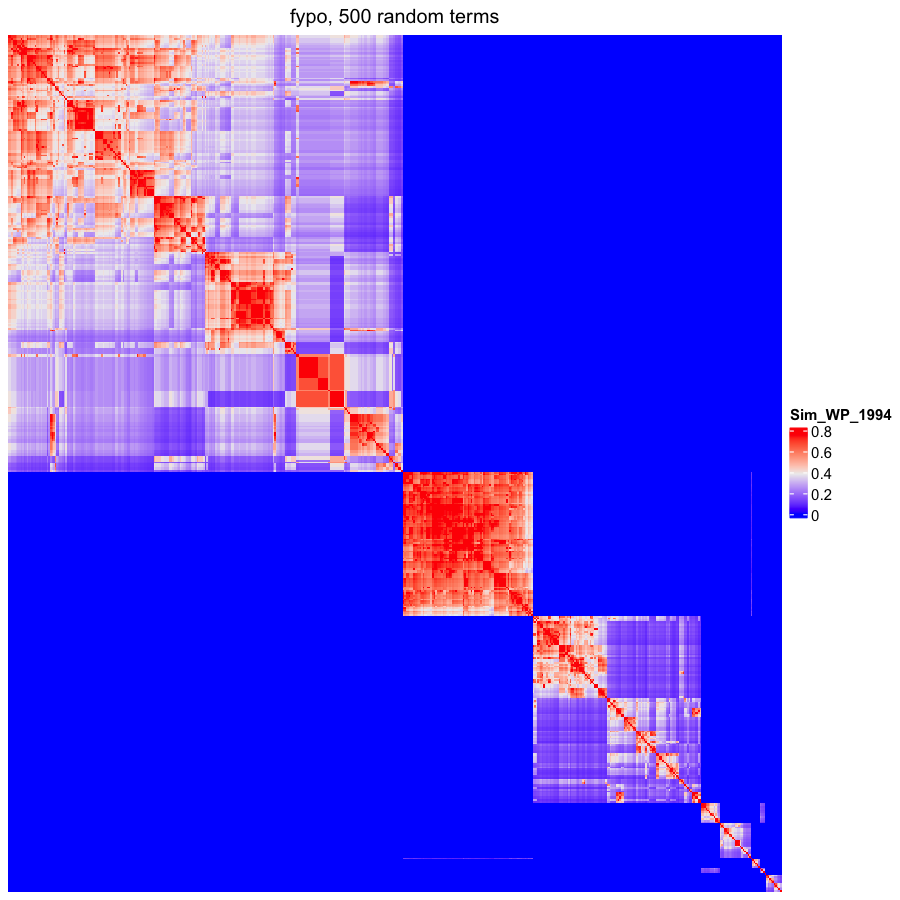

Supplement: Supplementary file 6 — Supplementary Material 6. OBO Foundry gallery [file 12864_2024_10759_MOESM6_ESM.zip › suppl6_OBOFoundry_gallery/image/OBOFoundry_fypo_heatmap.png]

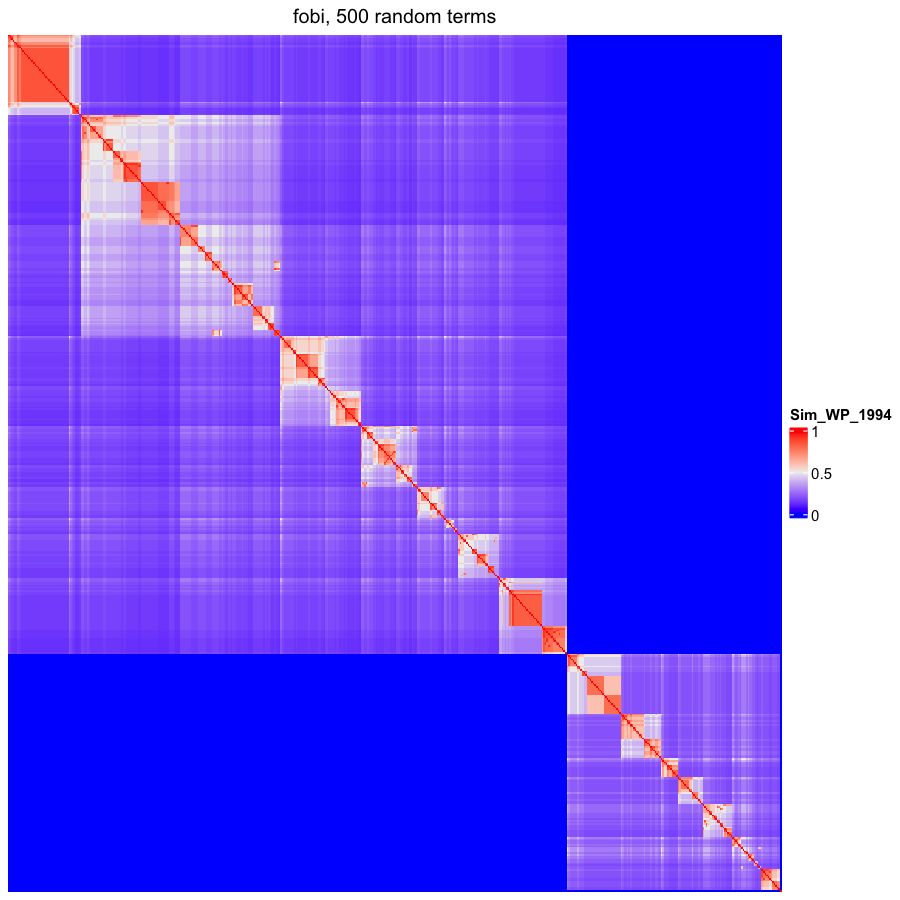

Supplement: Supplementary file 6 — Supplementary Material 6. OBO Foundry gallery [file 12864_2024_10759_MOESM6_ESM.zip › suppl6_OBOFoundry_gallery/image/OBOFoundry_fobi_heatmap.png]

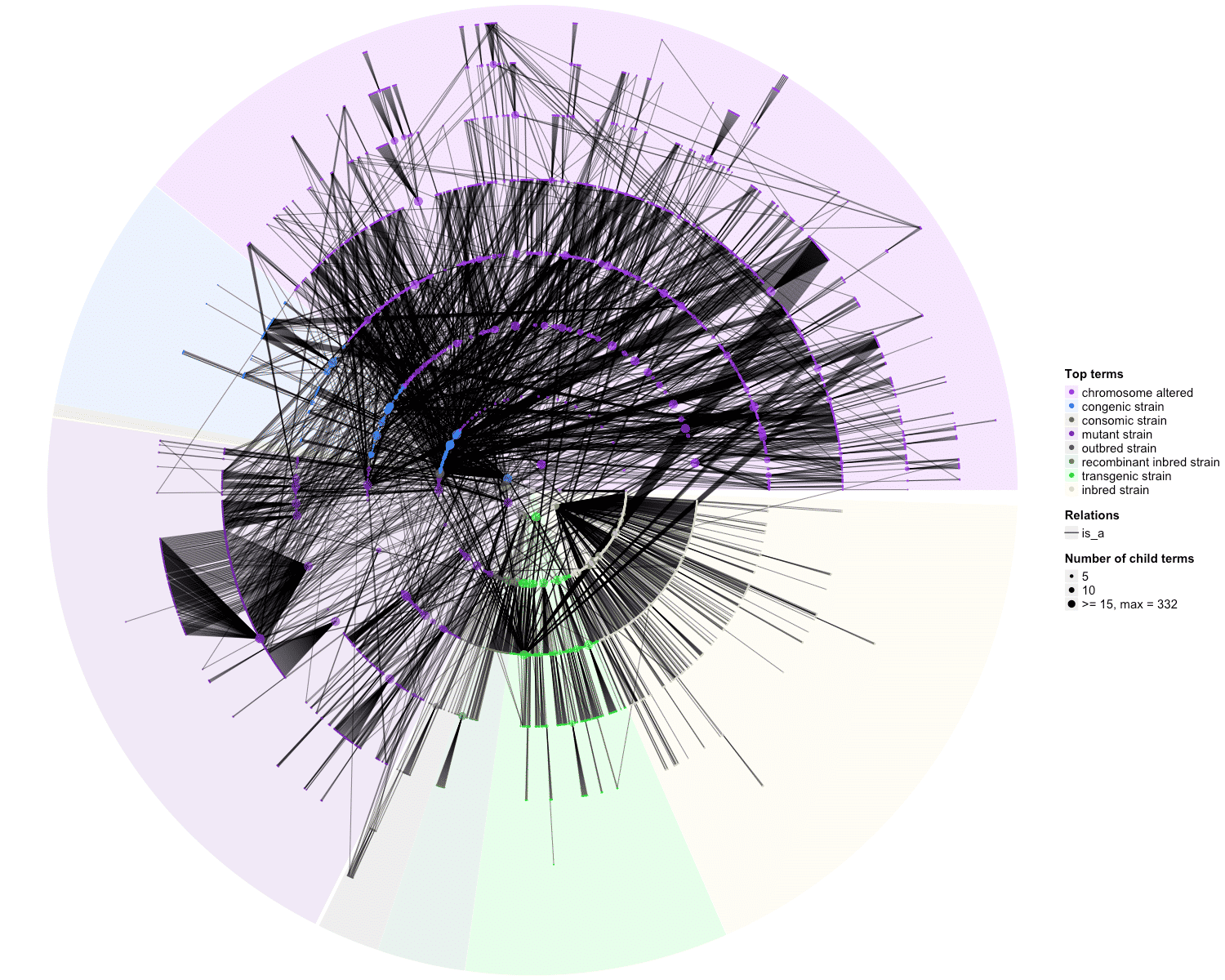

Supplement: Supplementary file 6 — Supplementary Material 6. OBO Foundry gallery [file 12864_2024_10759_MOESM6_ESM.zip › suppl6_OBOFoundry_gallery/image/OBOFoundry_rs.png]

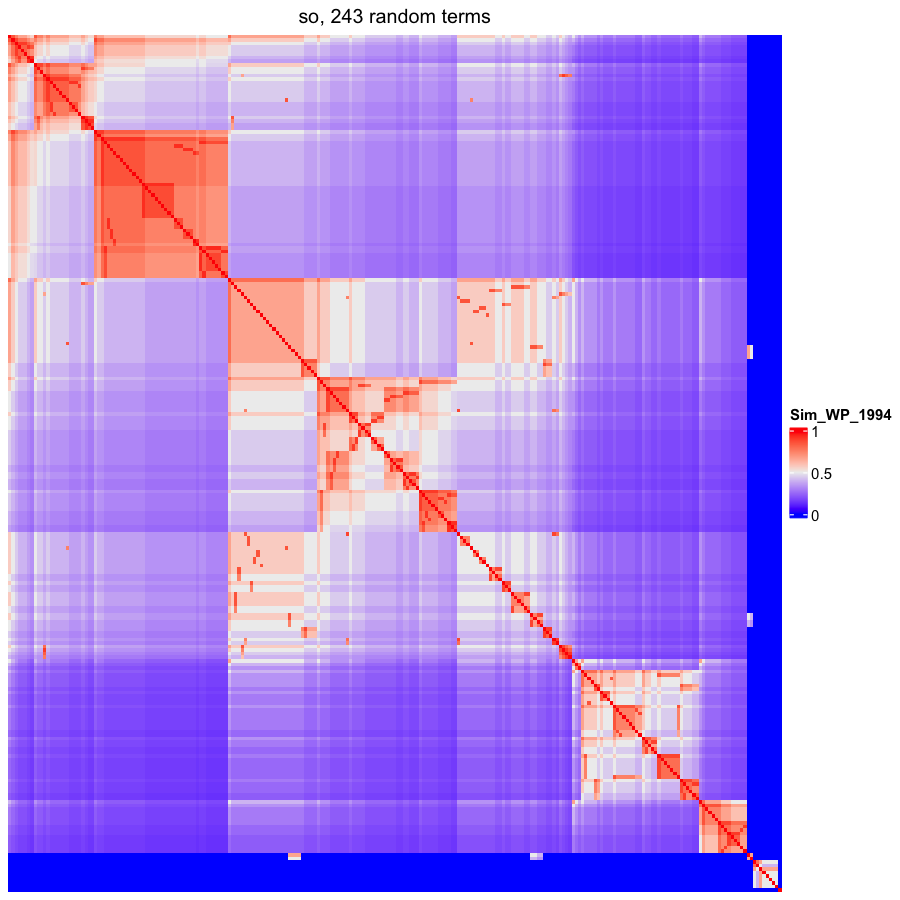

Supplement: Supplementary file 6 — Supplementary Material 6. OBO Foundry gallery [file 12864_2024_10759_MOESM6_ESM.zip › suppl6_OBOFoundry_gallery/image/OBOFoundry_so_heatmap.png]

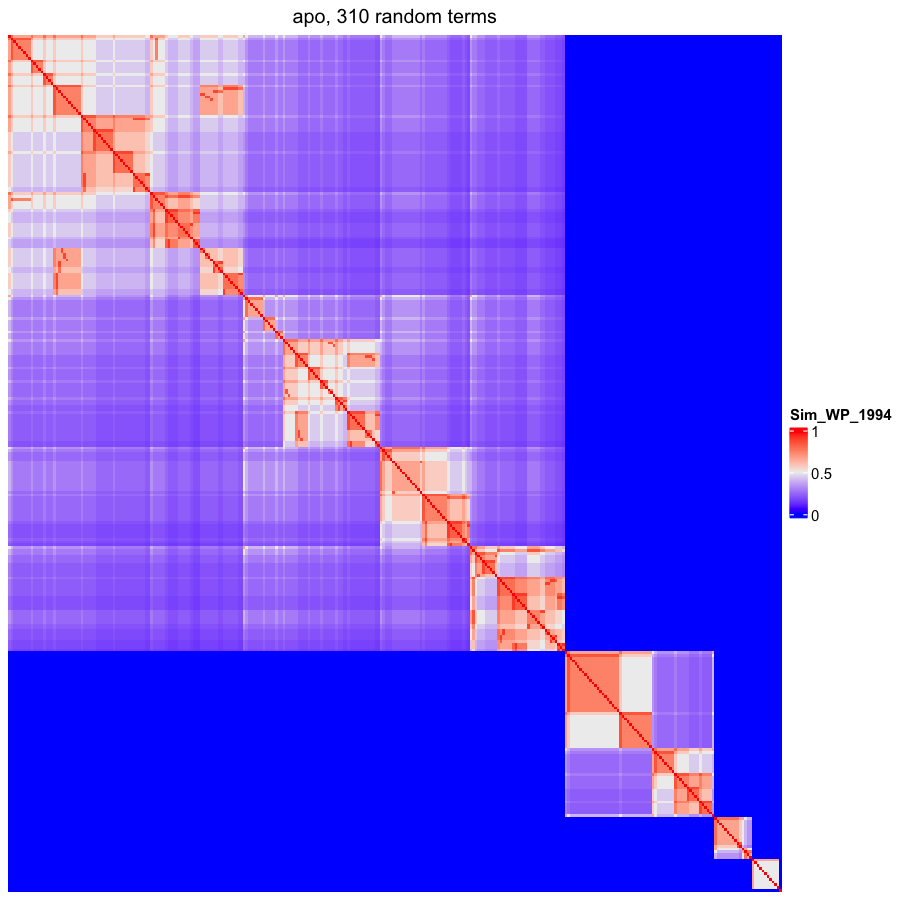

Supplement: Supplementary file 6 — Supplementary Material 6. OBO Foundry gallery [file 12864_2024_10759_MOESM6_ESM.zip › suppl6_OBOFoundry_gallery/image/OBOFoundry_apo_heatmap.png]

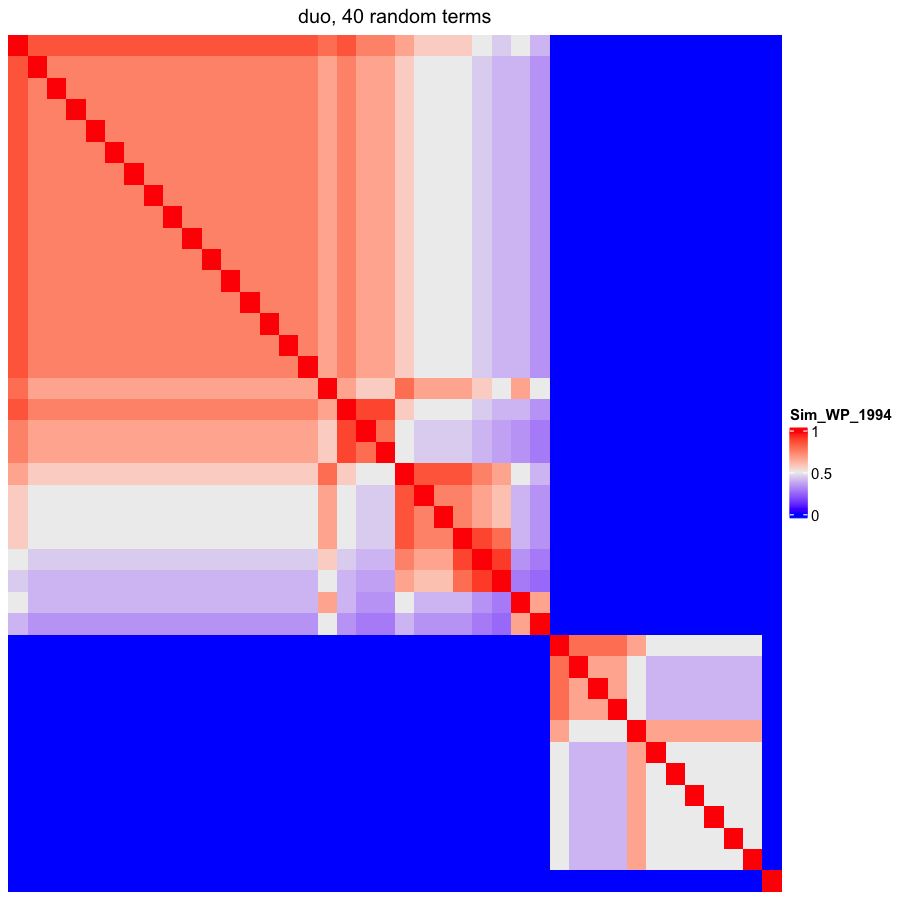

Supplement: Supplementary file 6 — Supplementary Material 6. OBO Foundry gallery [file 12864_2024_10759_MOESM6_ESM.zip › suppl6_OBOFoundry_gallery/image/OBOFoundry_duo_heatmap.png]

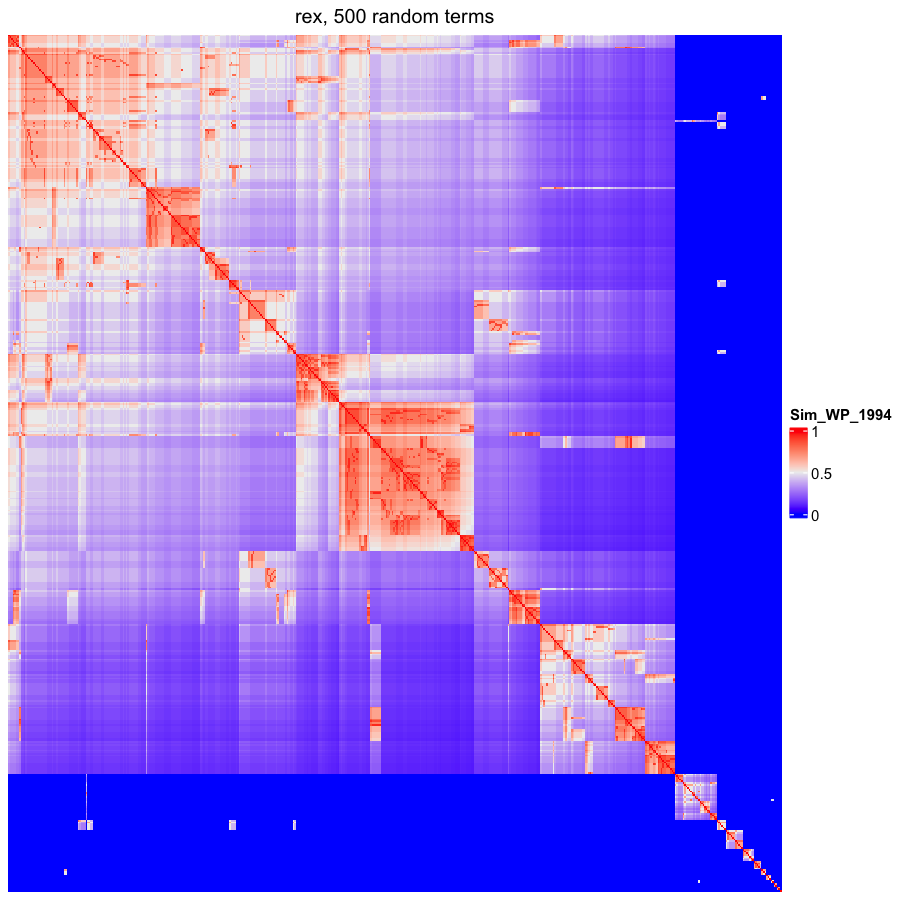

Supplement: Supplementary file 6 — Supplementary Material 6. OBO Foundry gallery [file 12864_2024_10759_MOESM6_ESM.zip › suppl6_OBOFoundry_gallery/image/OBOFoundry_rex_heatmap.png]

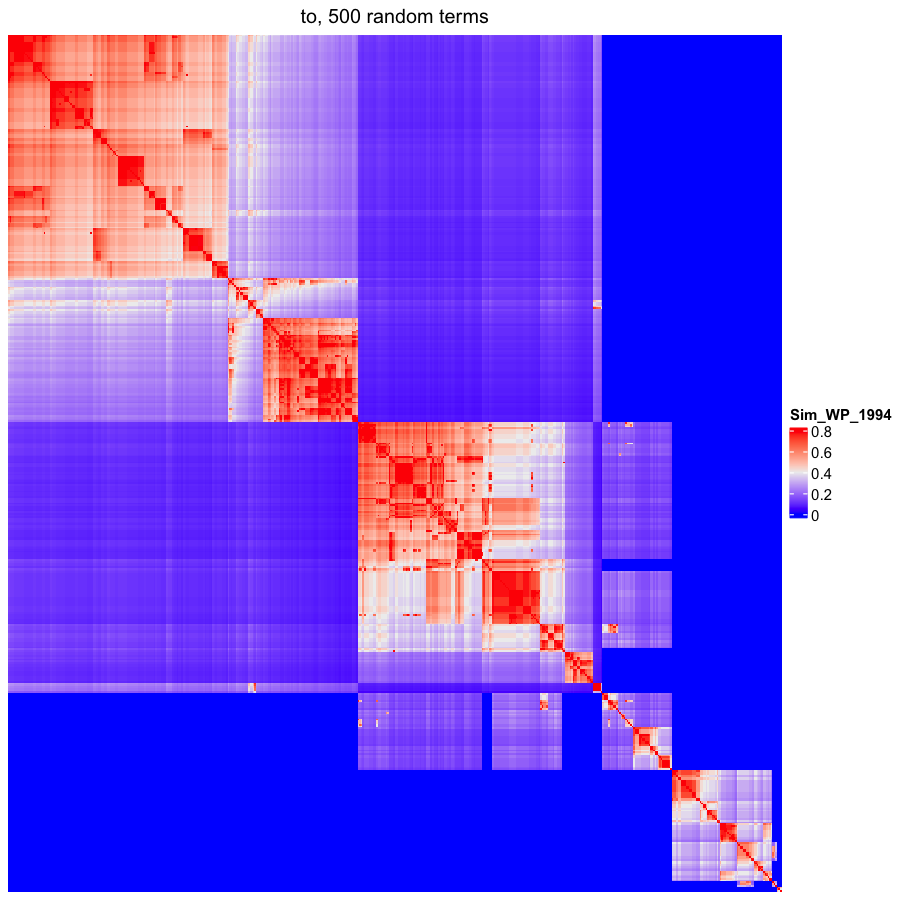

Supplement: Supplementary file 6 — Supplementary Material 6. OBO Foundry gallery [file 12864_2024_10759_MOESM6_ESM.zip › suppl6_OBOFoundry_gallery/image/OBOFoundry_to_heatmap.png]

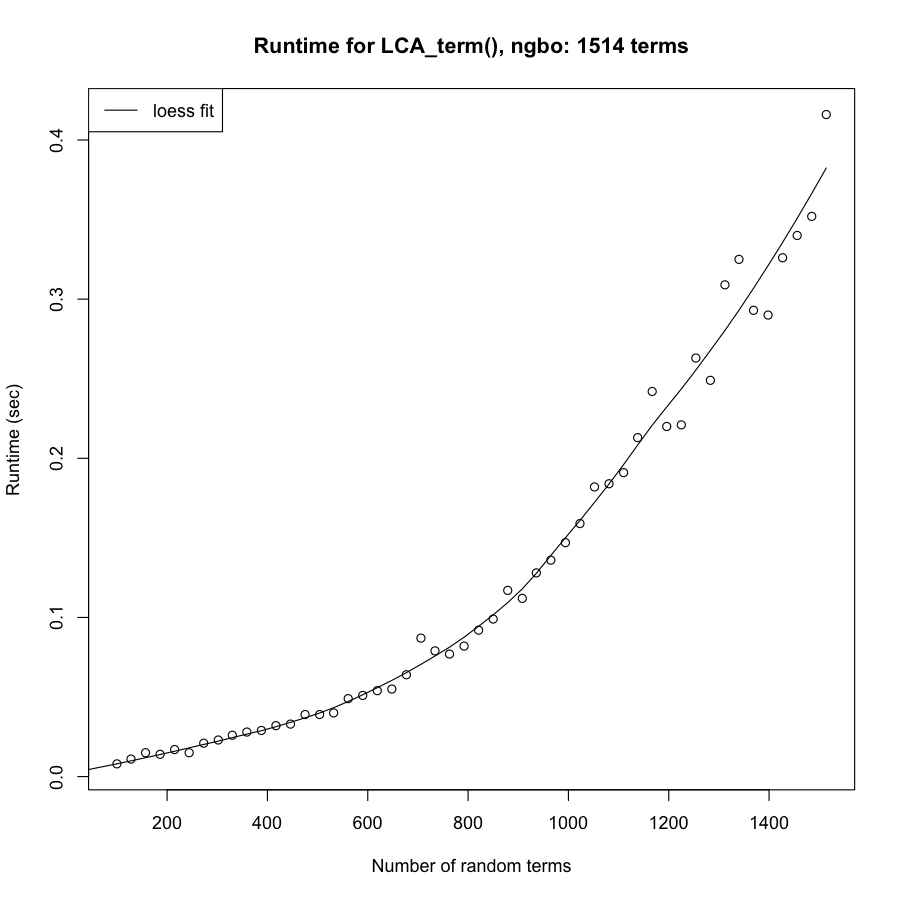

Supplement: Supplementary file 6 — Supplementary Material 6. OBO Foundry gallery [file 12864_2024_10759_MOESM6_ESM.zip › suppl6_OBOFoundry_gallery/image/OBOFoundry_ngbo_runtime.png]

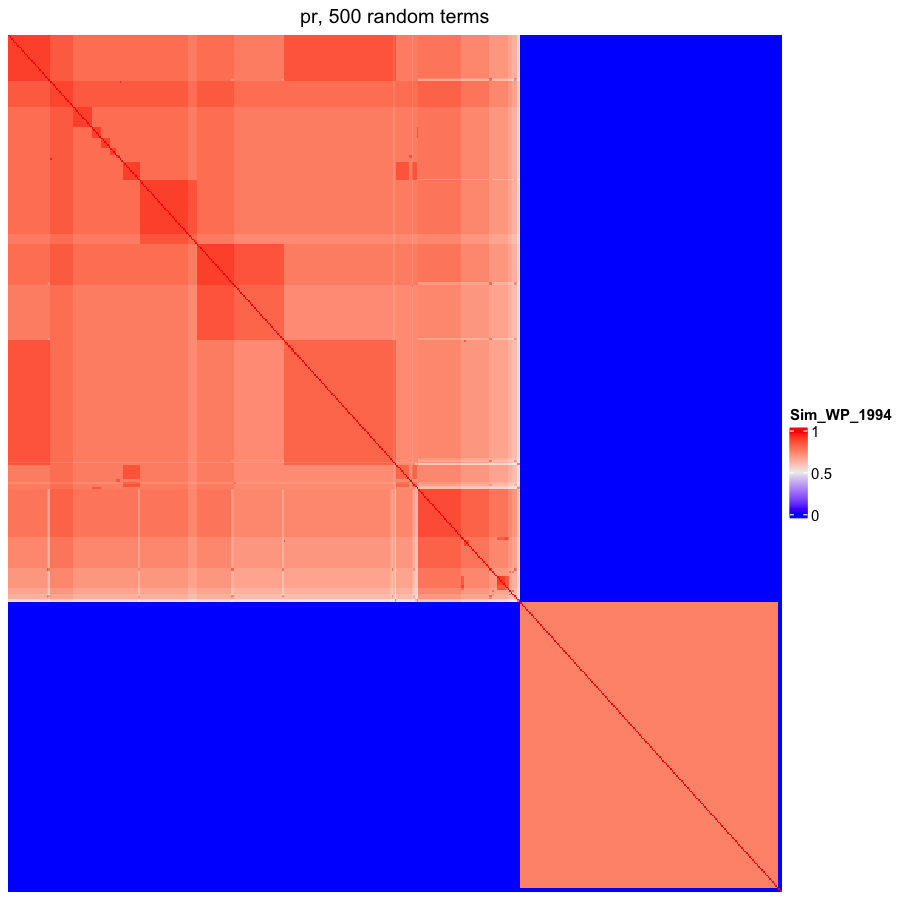

Supplement: Supplementary file 6 — Supplementary Material 6. OBO Foundry gallery [file 12864_2024_10759_MOESM6_ESM.zip › suppl6_OBOFoundry_gallery/image/OBOFoundry_pr_heatmap.png]

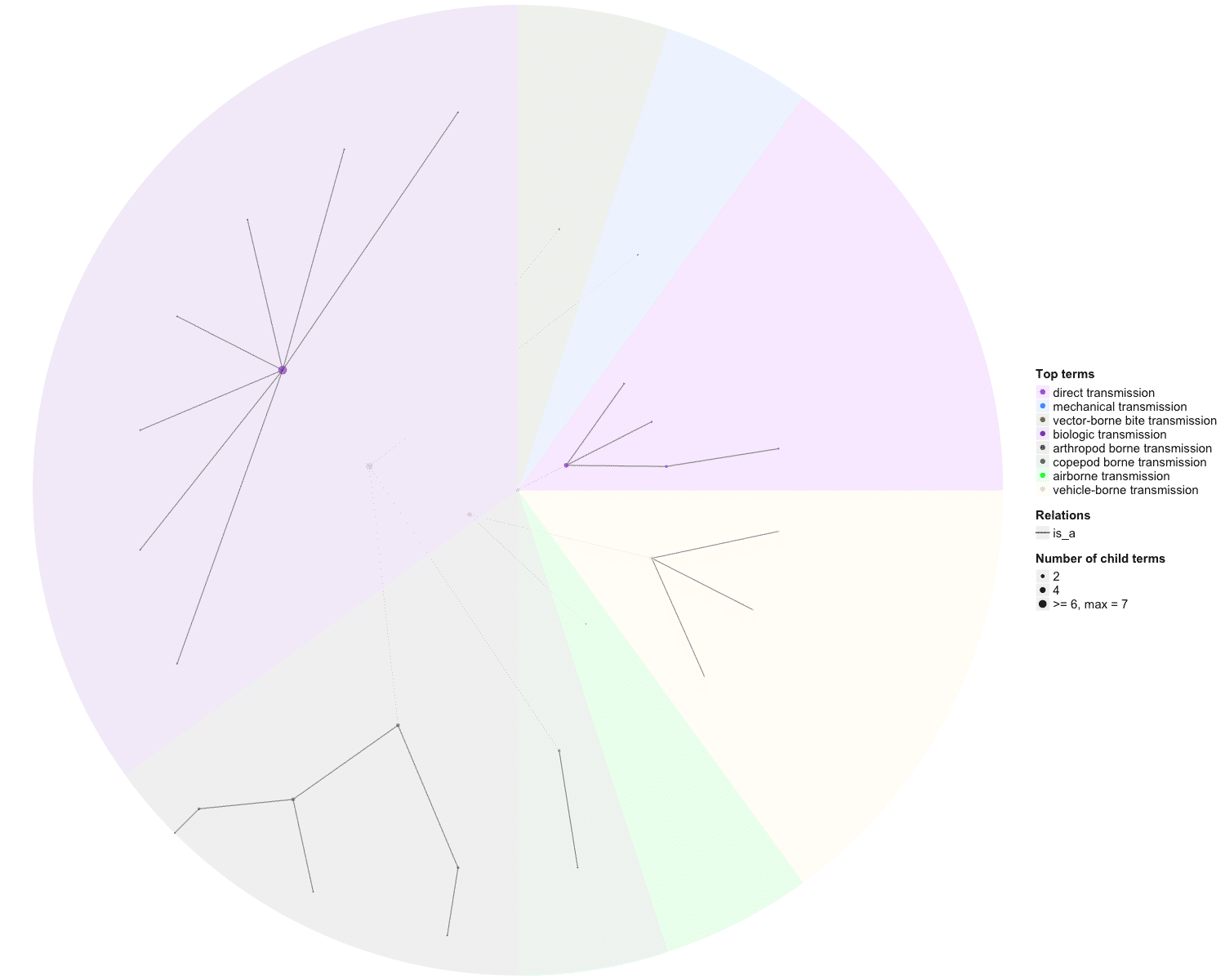

Supplement: Supplementary file 6 — Supplementary Material 6. OBO Foundry gallery [file 12864_2024_10759_MOESM6_ESM.zip › suppl6_OBOFoundry_gallery/image/OBOFoundry_trans.png]

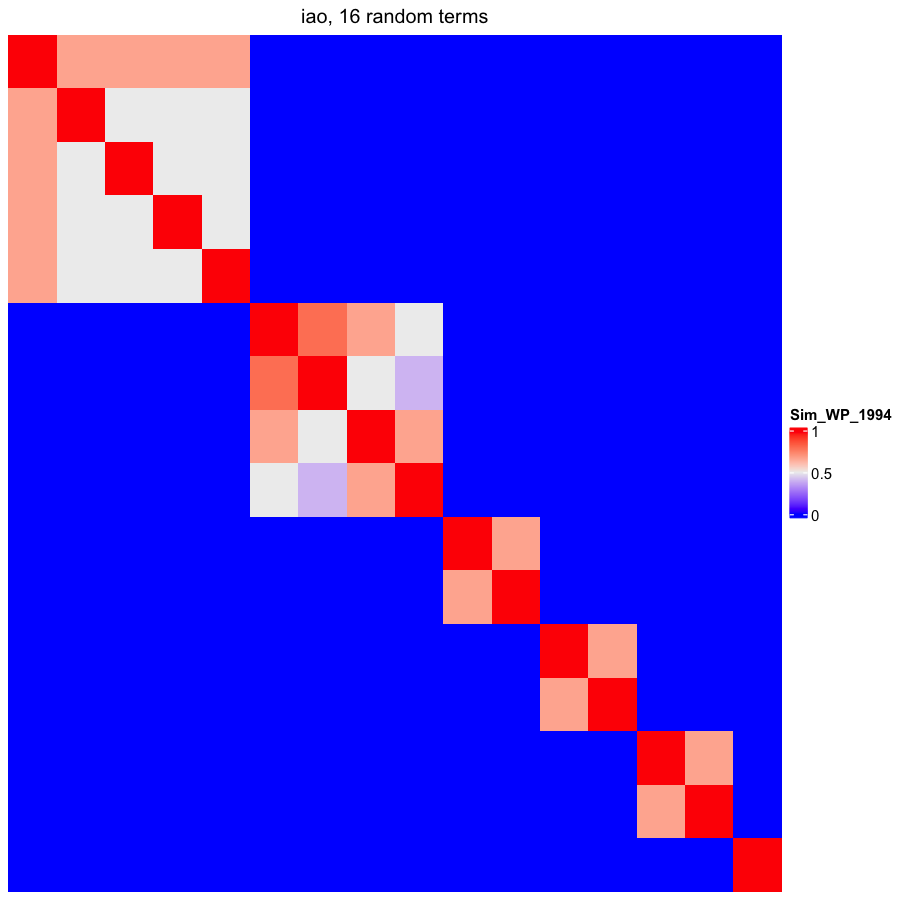

Supplement: Supplementary file 6 — Supplementary Material 6. OBO Foundry gallery [file 12864_2024_10759_MOESM6_ESM.zip › suppl6_OBOFoundry_gallery/image/OBOFoundry_iao_heatmap.png]

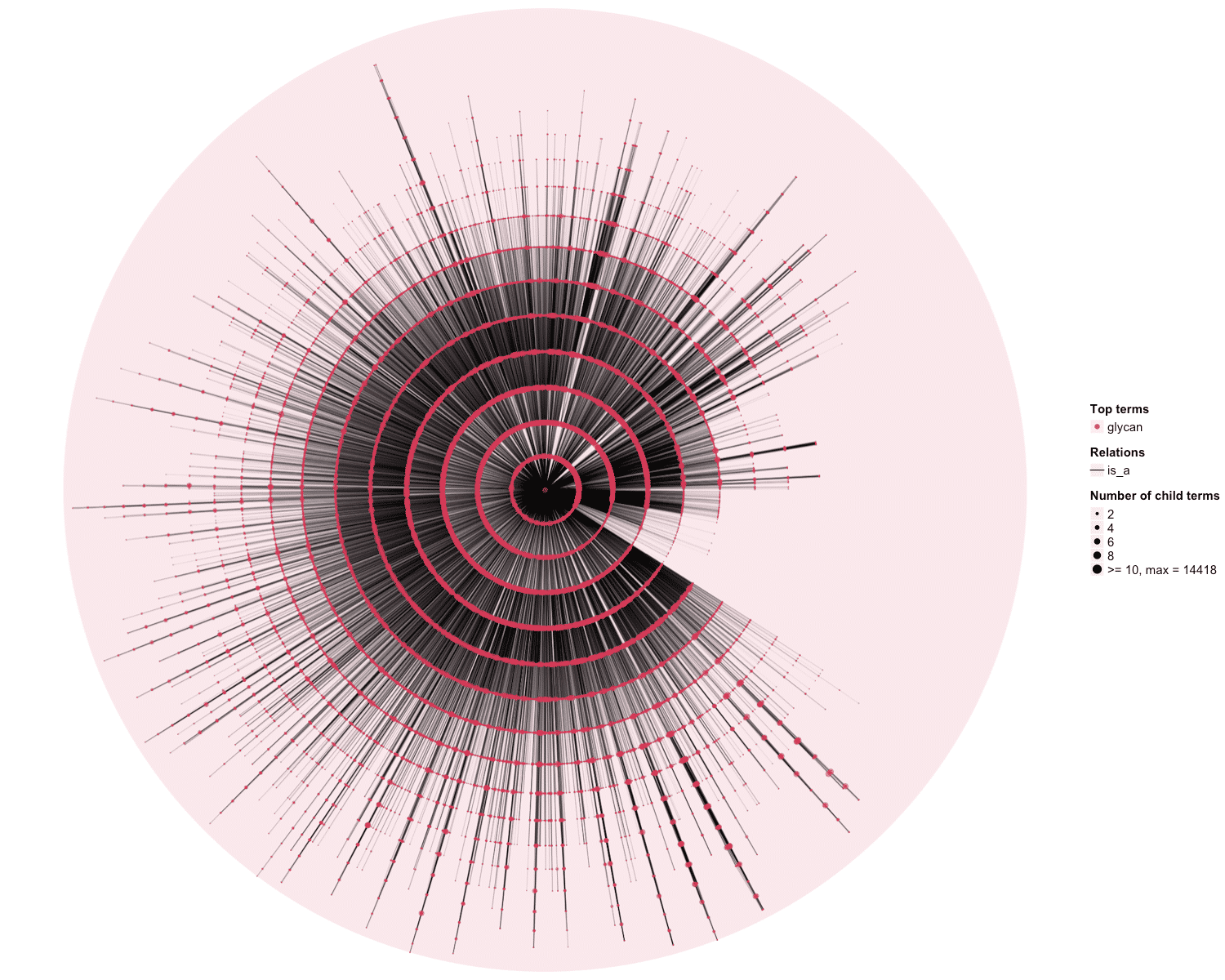

Supplement: Supplementary file 6 — Supplementary Material 6. OBO Foundry gallery [file 12864_2024_10759_MOESM6_ESM.zip › suppl6_OBOFoundry_gallery/image/OBOFoundry_gno.png]

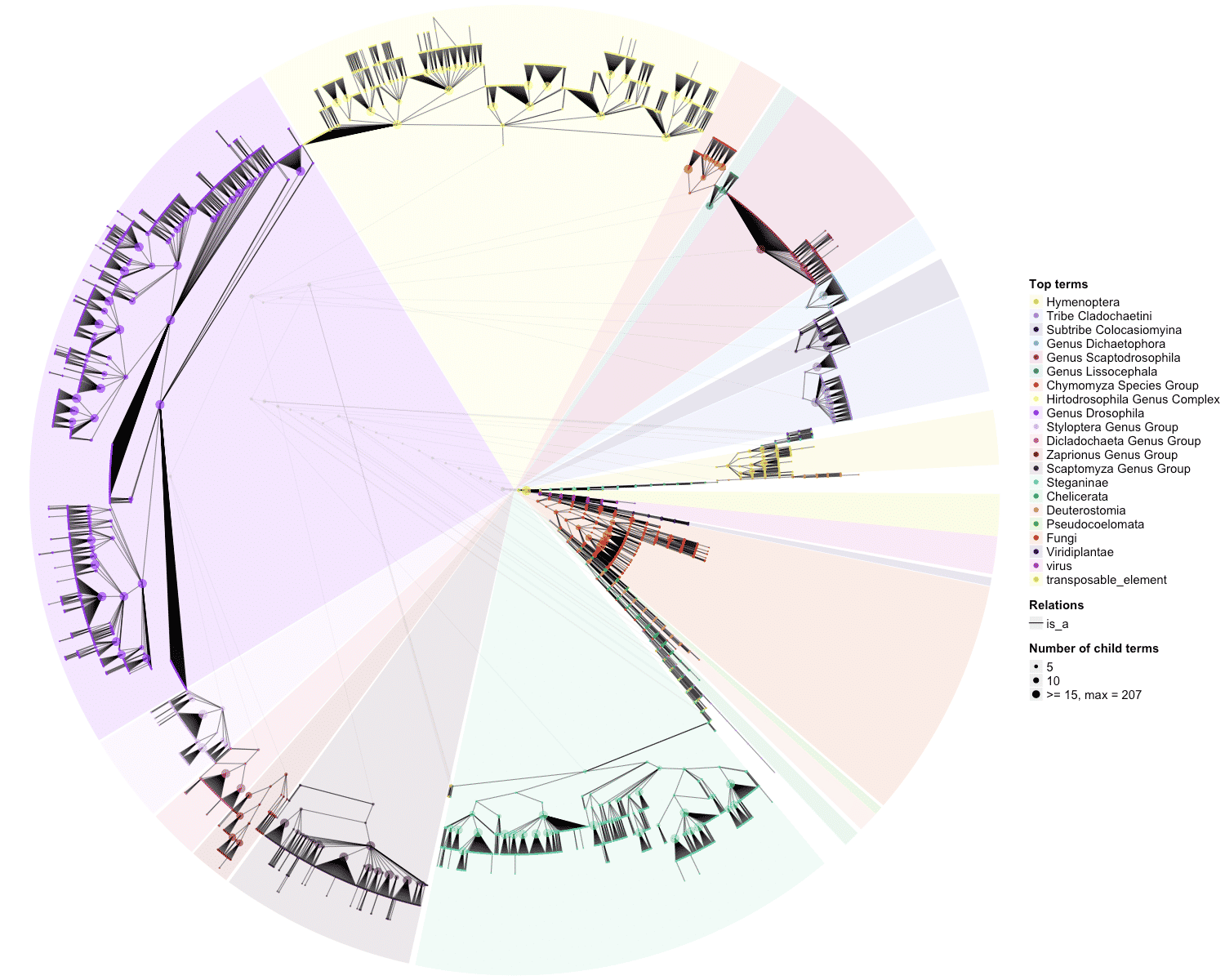

Supplement: Supplementary file 6 — Supplementary Material 6. OBO Foundry gallery [file 12864_2024_10759_MOESM6_ESM.zip › suppl6_OBOFoundry_gallery/image/OBOFoundry_fbsp.png]

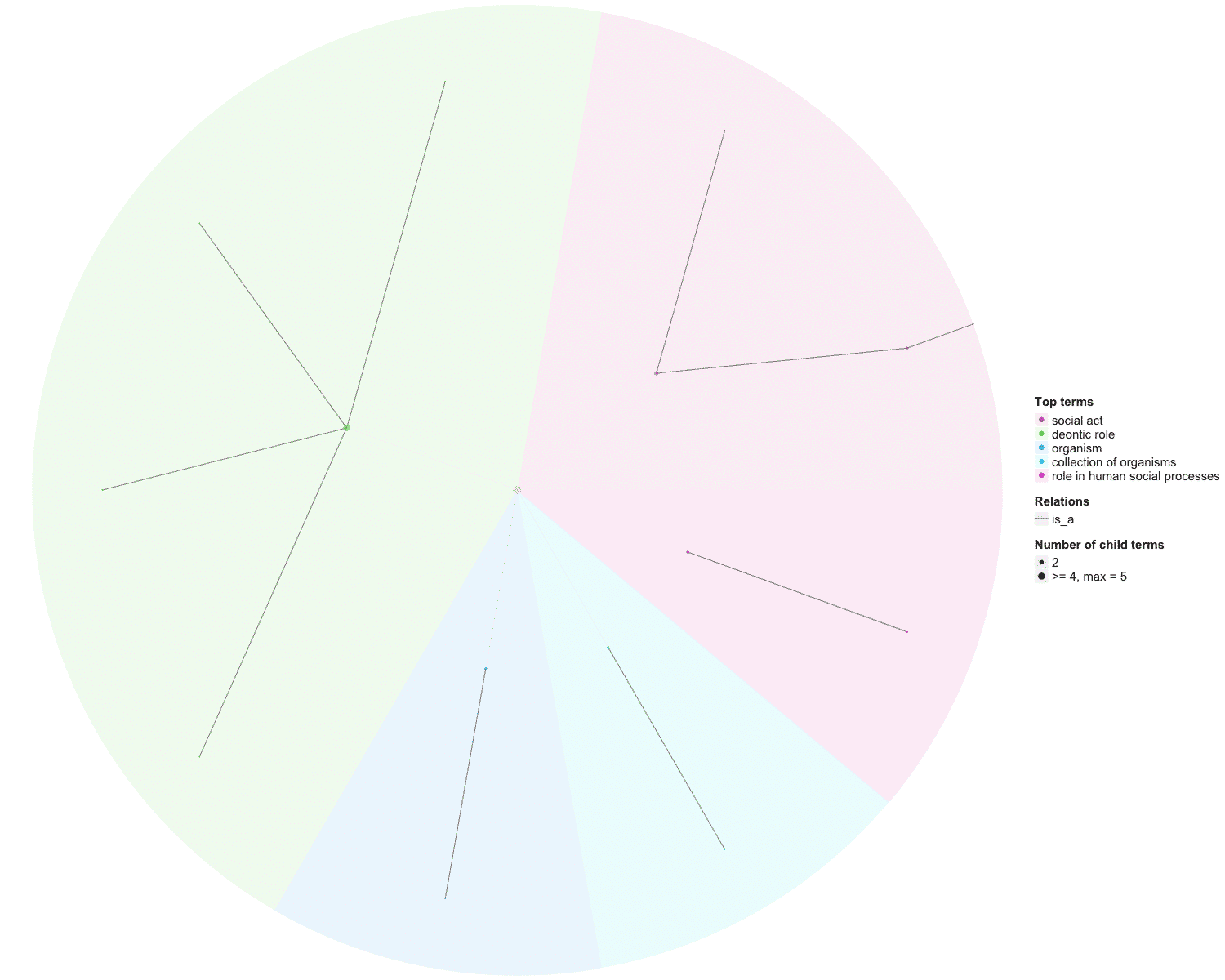

Supplement: Supplementary file 6 — Supplementary Material 6. OBO Foundry gallery [file 12864_2024_10759_MOESM6_ESM.zip › suppl6_OBOFoundry_gallery/image/OBOFoundry_iao.png]

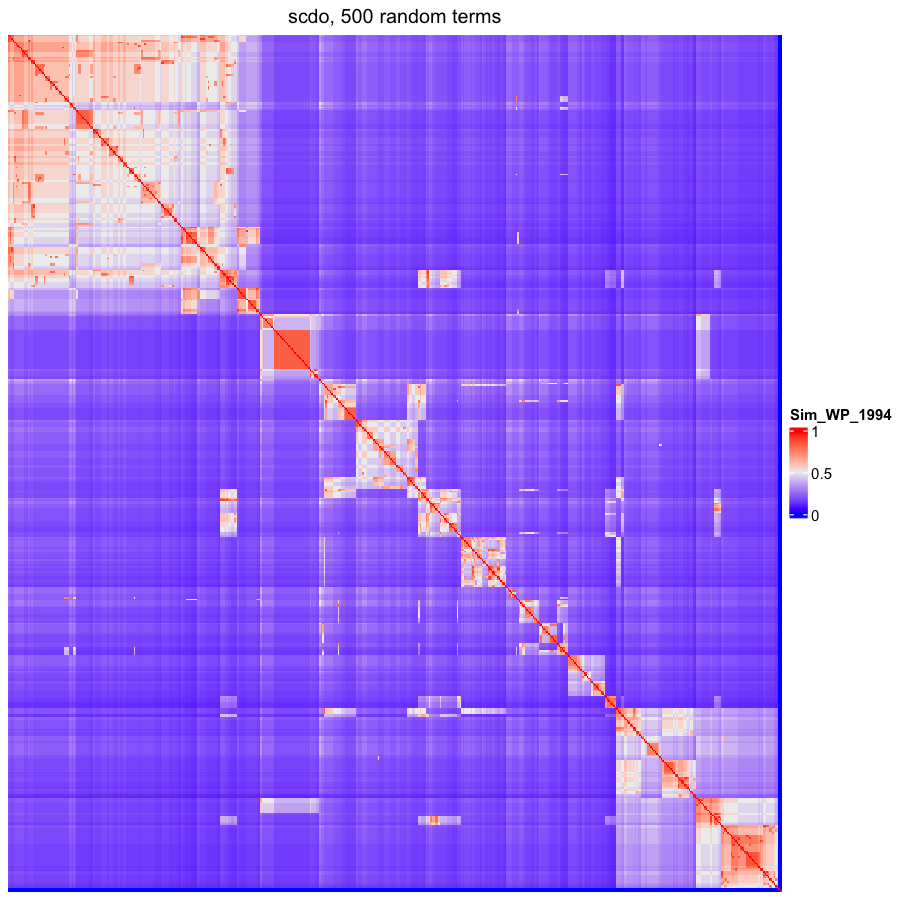

Supplement: Supplementary file 6 — Supplementary Material 6. OBO Foundry gallery [file 12864_2024_10759_MOESM6_ESM.zip › suppl6_OBOFoundry_gallery/image/OBOFoundry_scdo_heatmap.png]

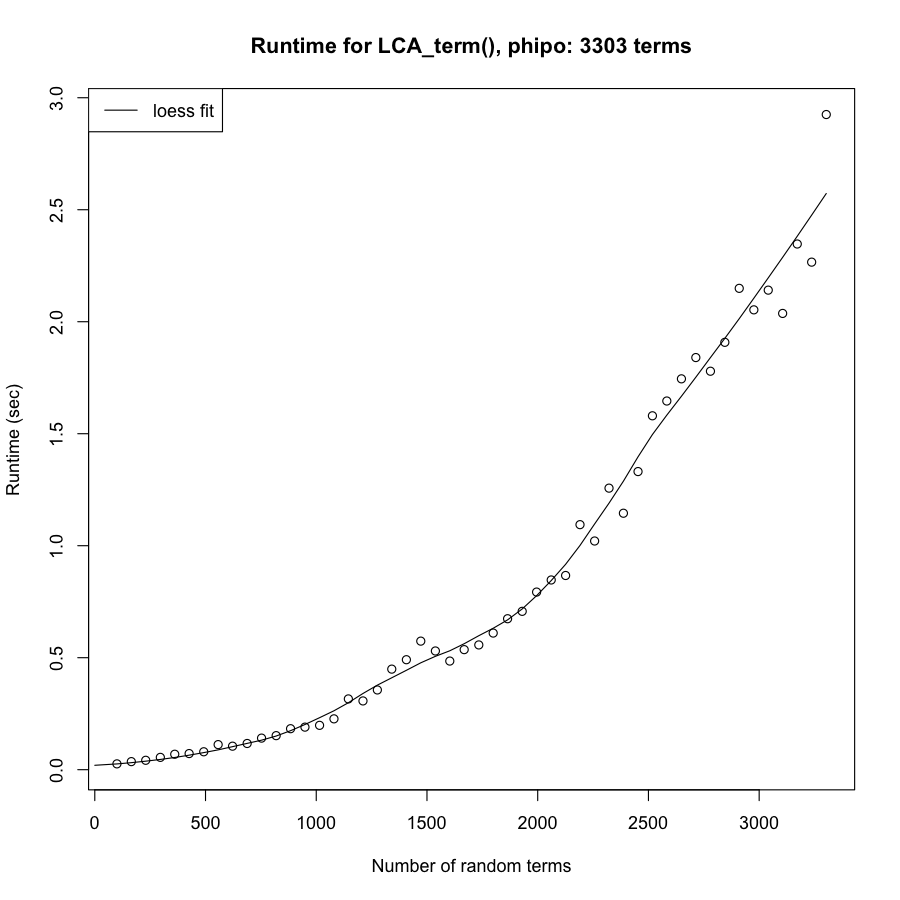

Supplement: Supplementary file 6 — Supplementary Material 6. OBO Foundry gallery [file 12864_2024_10759_MOESM6_ESM.zip › suppl6_OBOFoundry_gallery/image/OBOFoundry_phipo_runtime.png]

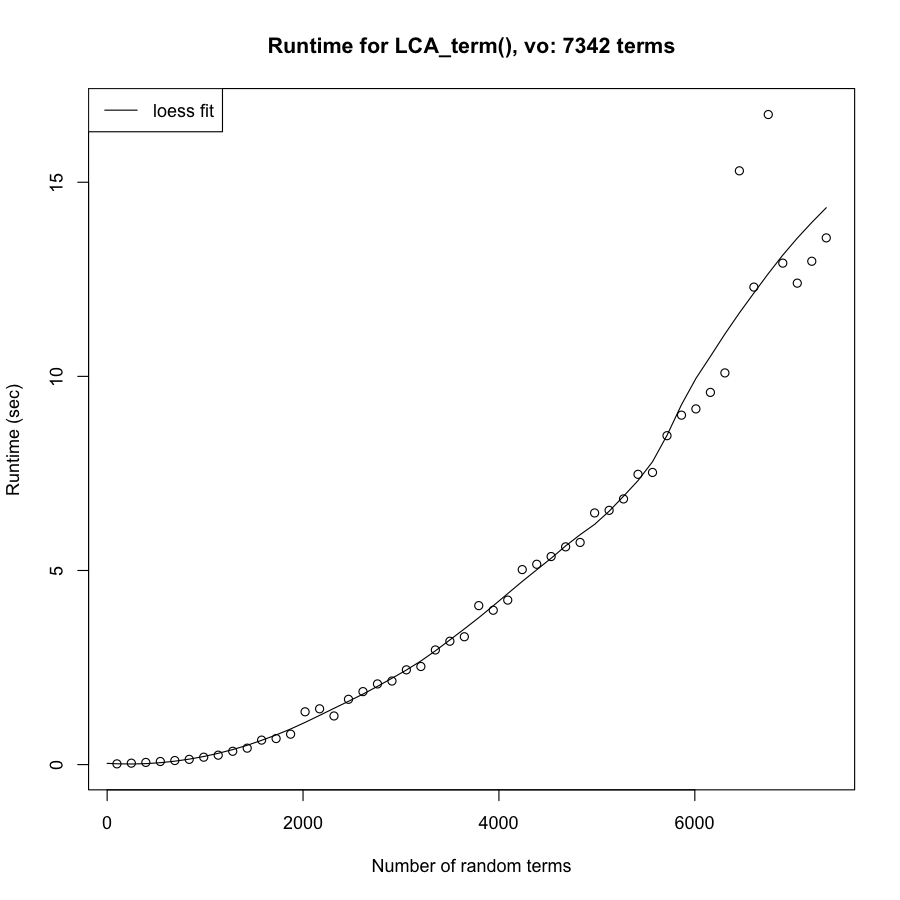

Supplement: Supplementary file 6 — Supplementary Material 6. OBO Foundry gallery [file 12864_2024_10759_MOESM6_ESM.zip › suppl6_OBOFoundry_gallery/image/OBOFoundry_vo_runtime.png]

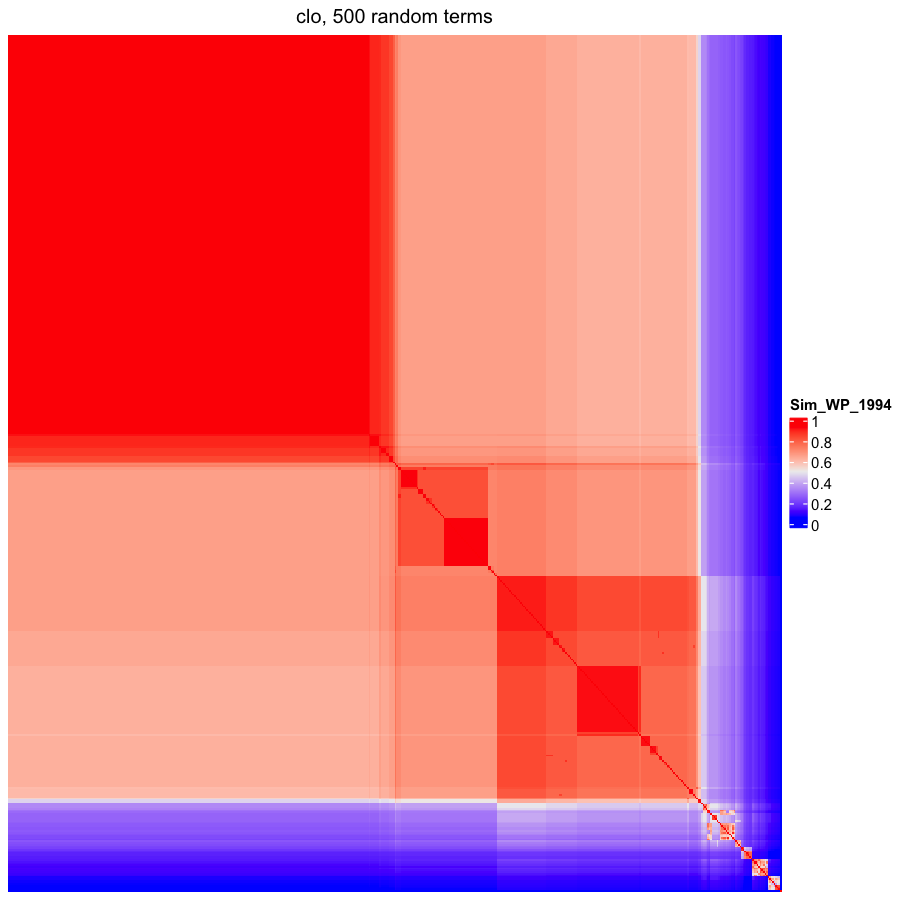

Supplement: Supplementary file 6 — Supplementary Material 6. OBO Foundry gallery [file 12864_2024_10759_MOESM6_ESM.zip › suppl6_OBOFoundry_gallery/image/OBOFoundry_clo_heatmap.png]

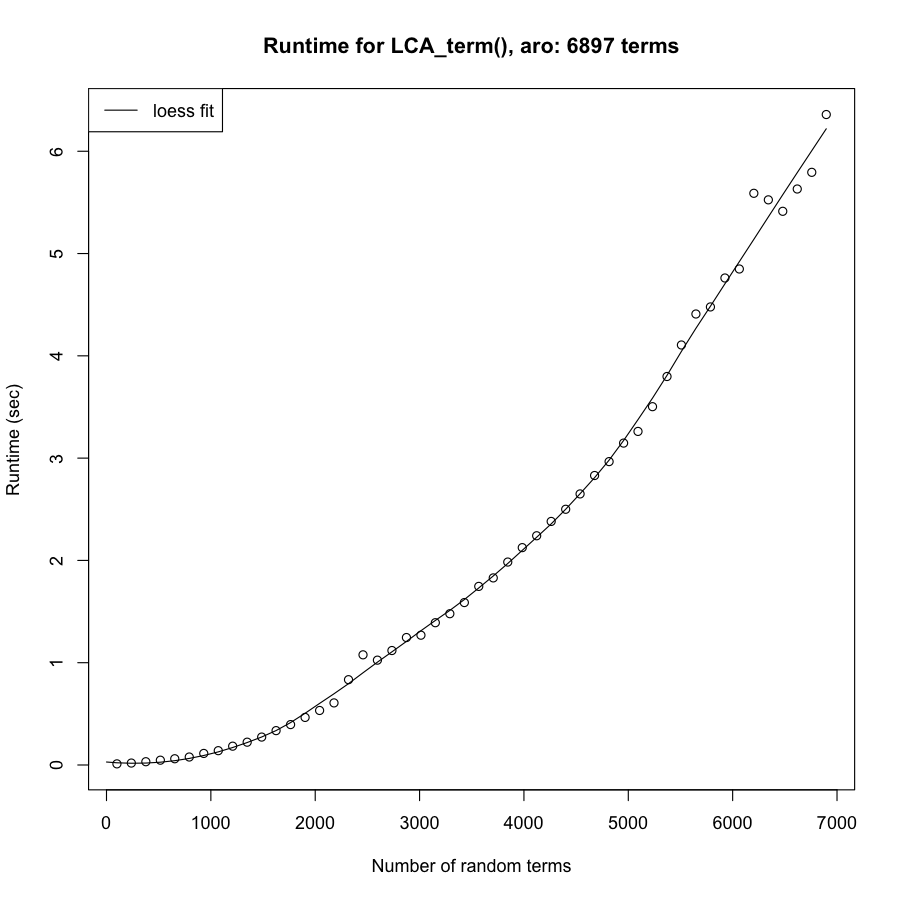

Supplement: Supplementary file 6 — Supplementary Material 6. OBO Foundry gallery [file 12864_2024_10759_MOESM6_ESM.zip › suppl6_OBOFoundry_gallery/image/OBOFoundry_aro_runtime.png]

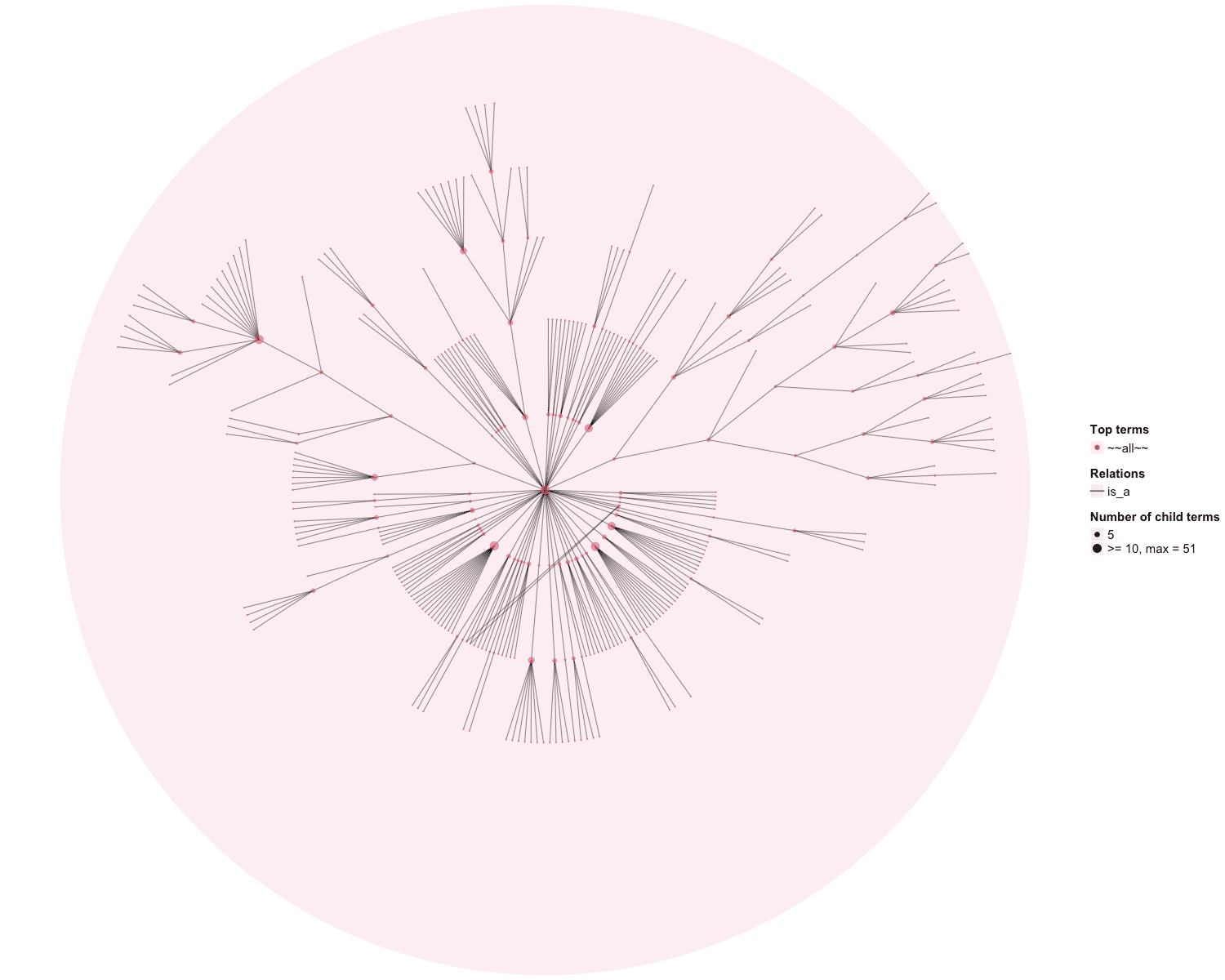

Supplement: Supplementary file 6 — Supplementary Material 6. OBO Foundry gallery [file 12864_2024_10759_MOESM6_ESM.zip › suppl6_OBOFoundry_gallery/image/OBOFoundry_ontoneo.png]

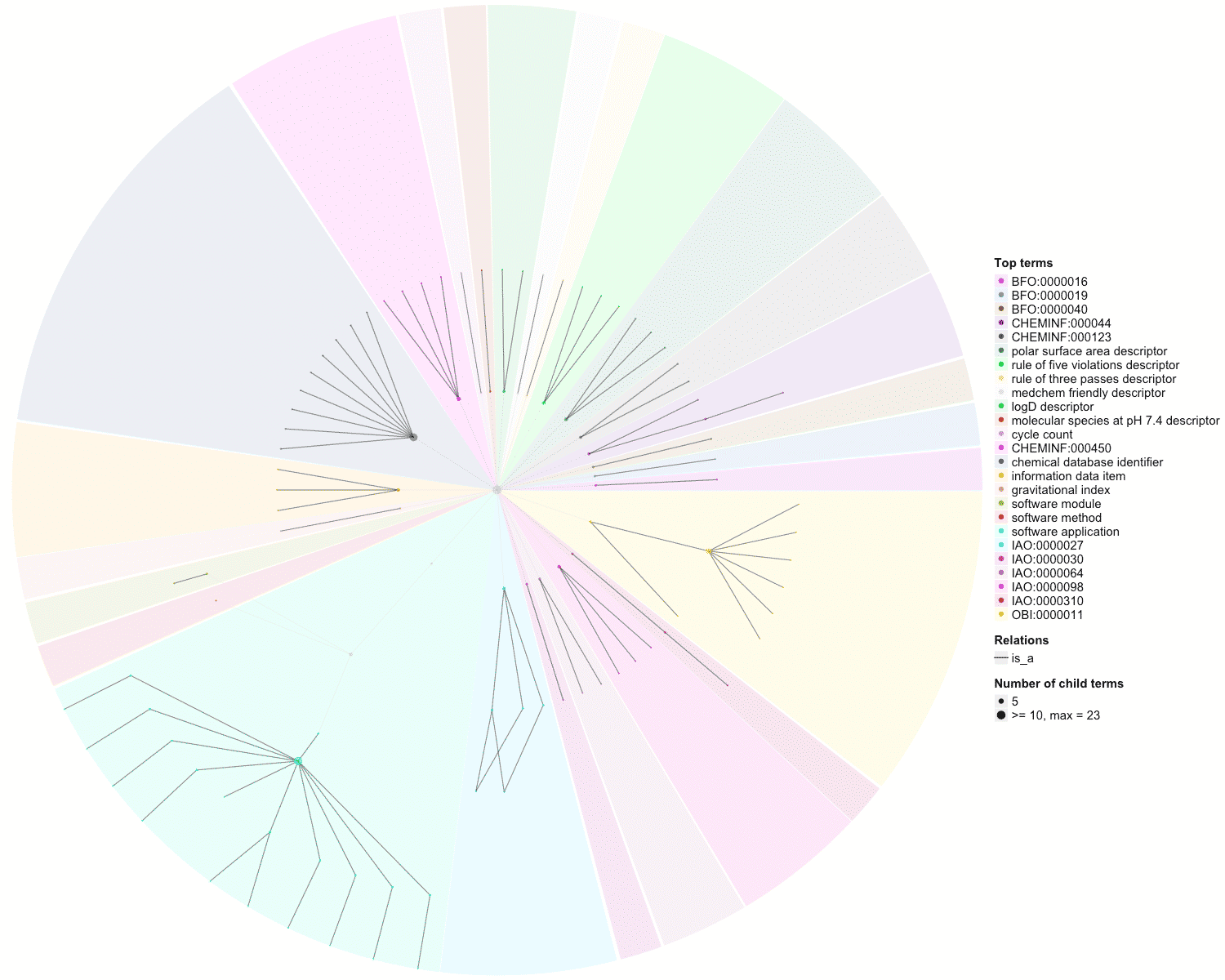

Supplement: Supplementary file 6 — Supplementary Material 6. OBO Foundry gallery [file 12864_2024_10759_MOESM6_ESM.zip › suppl6_OBOFoundry_gallery/image/OBOFoundry_cheminf.png]

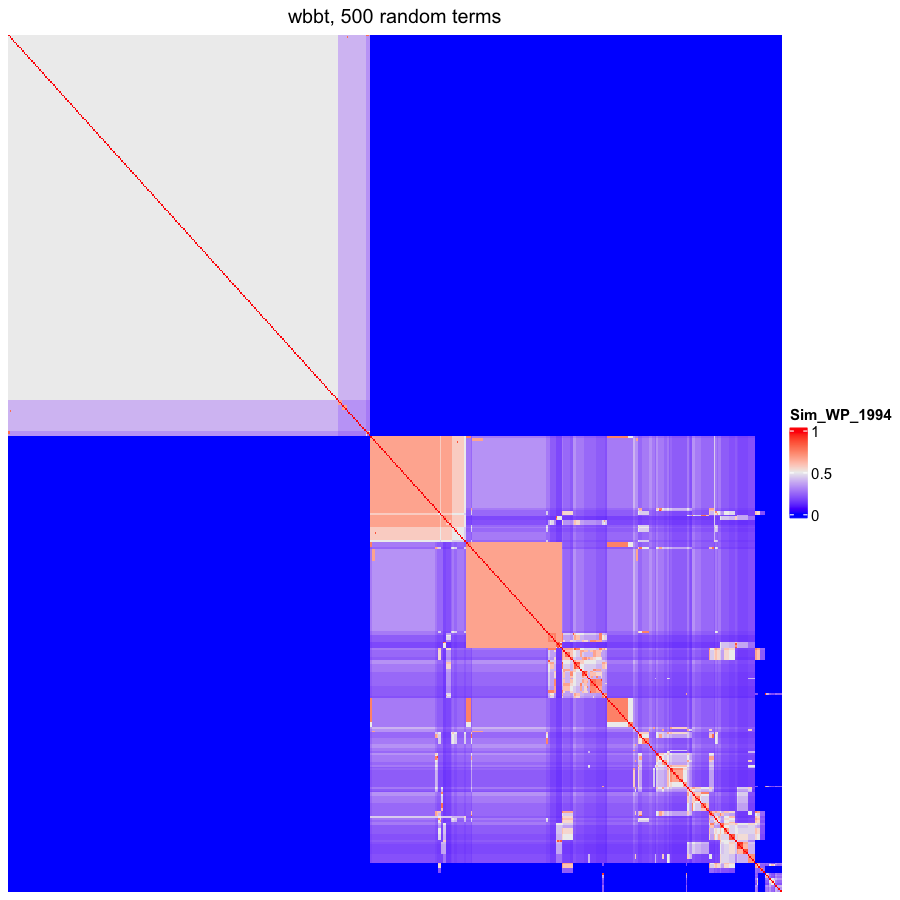

Supplement: Supplementary file 6 — Supplementary Material 6. OBO Foundry gallery [file 12864_2024_10759_MOESM6_ESM.zip › suppl6_OBOFoundry_gallery/image/OBOFoundry_wbbt_heatmap.png]

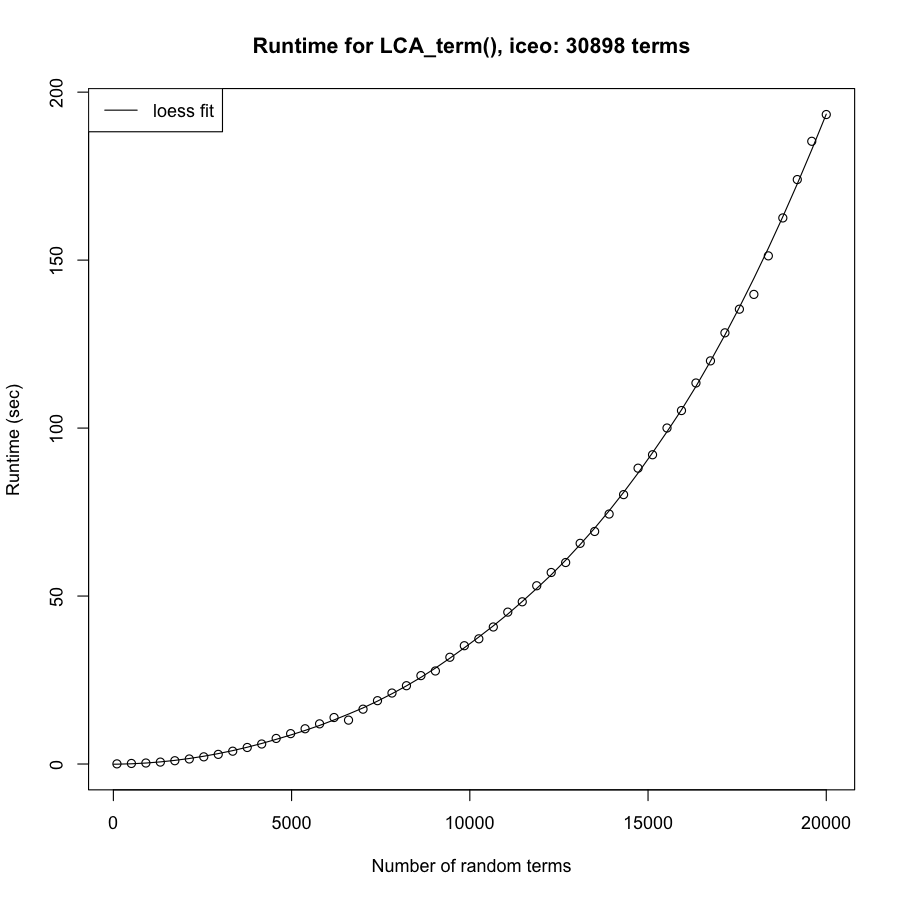

Supplement: Supplementary file 6 — Supplementary Material 6. OBO Foundry gallery [file 12864_2024_10759_MOESM6_ESM.zip › suppl6_OBOFoundry_gallery/image/OBOFoundry_iceo_runtime.png]

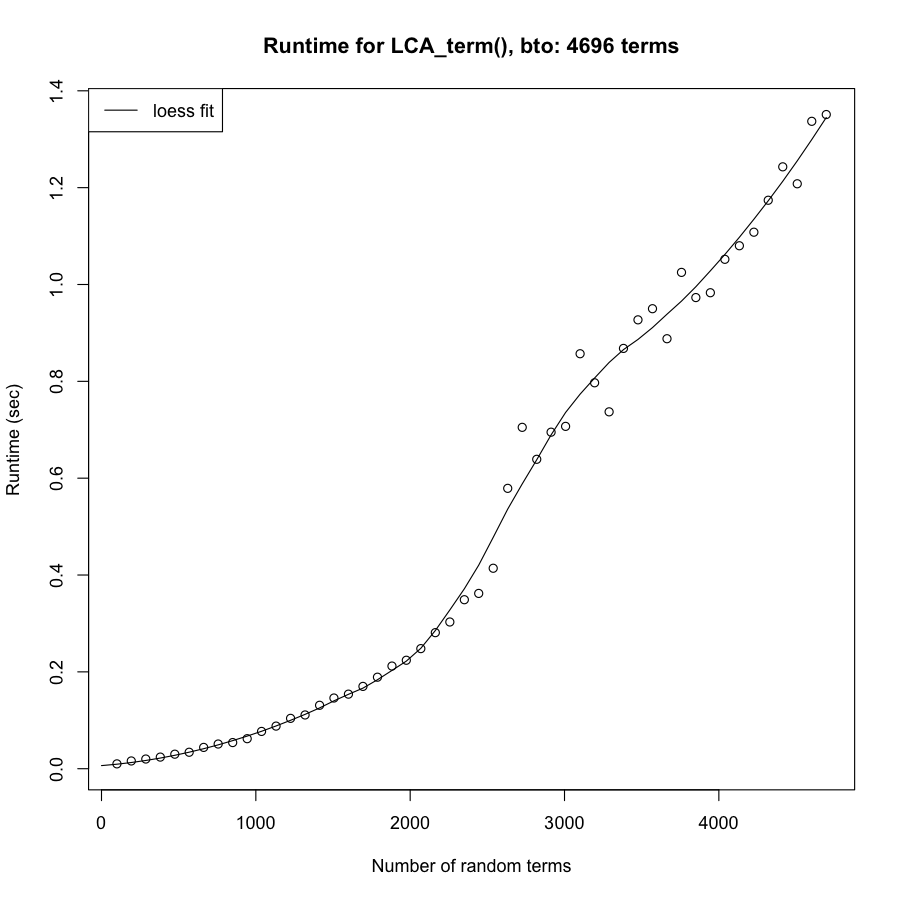

Supplement: Supplementary file 6 — Supplementary Material 6. OBO Foundry gallery [file 12864_2024_10759_MOESM6_ESM.zip › suppl6_OBOFoundry_gallery/image/OBOFoundry_bto_runtime.png]

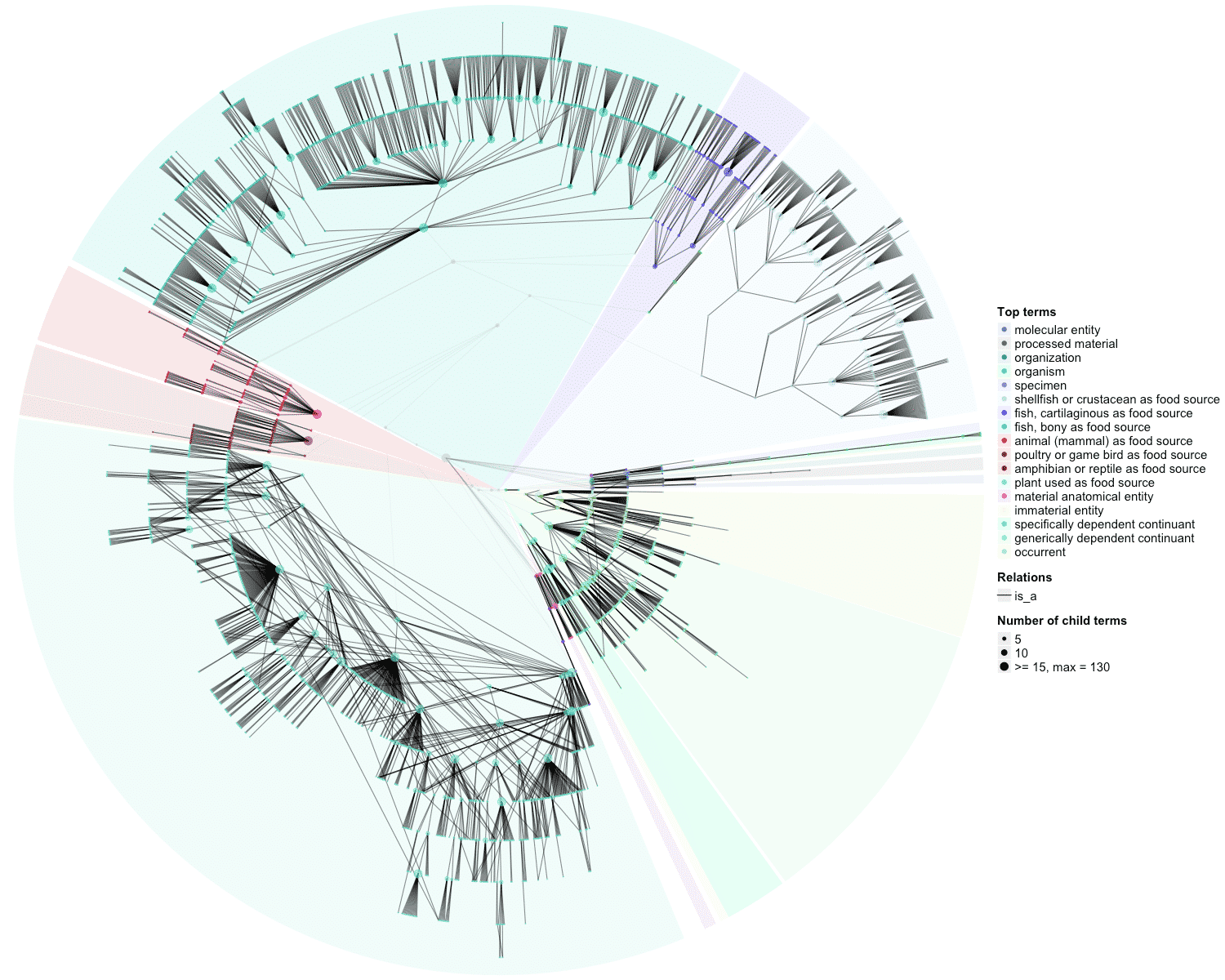

Supplement: Supplementary file 6 — Supplementary Material 6. OBO Foundry gallery [file 12864_2024_10759_MOESM6_ESM.zip › suppl6_OBOFoundry_gallery/image/OBOFoundry_ons.png]

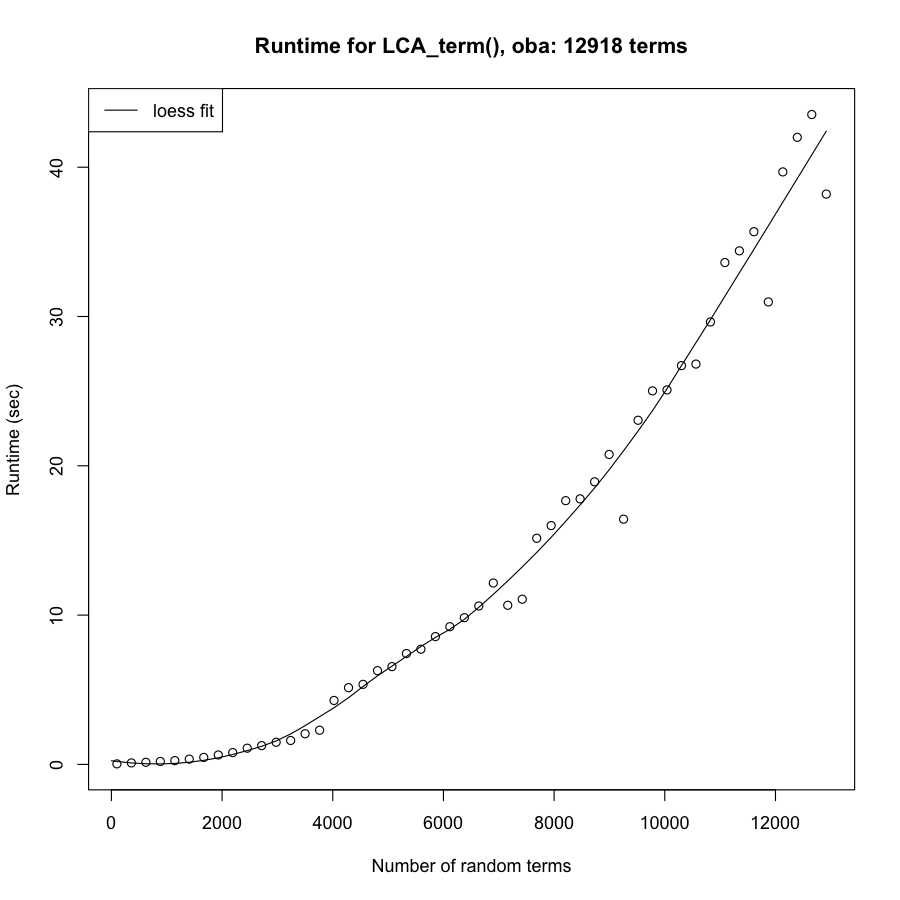

Supplement: Supplementary file 6 — Supplementary Material 6. OBO Foundry gallery [file 12864_2024_10759_MOESM6_ESM.zip › suppl6_OBOFoundry_gallery/image/OBOFoundry_oba_runtime.png]

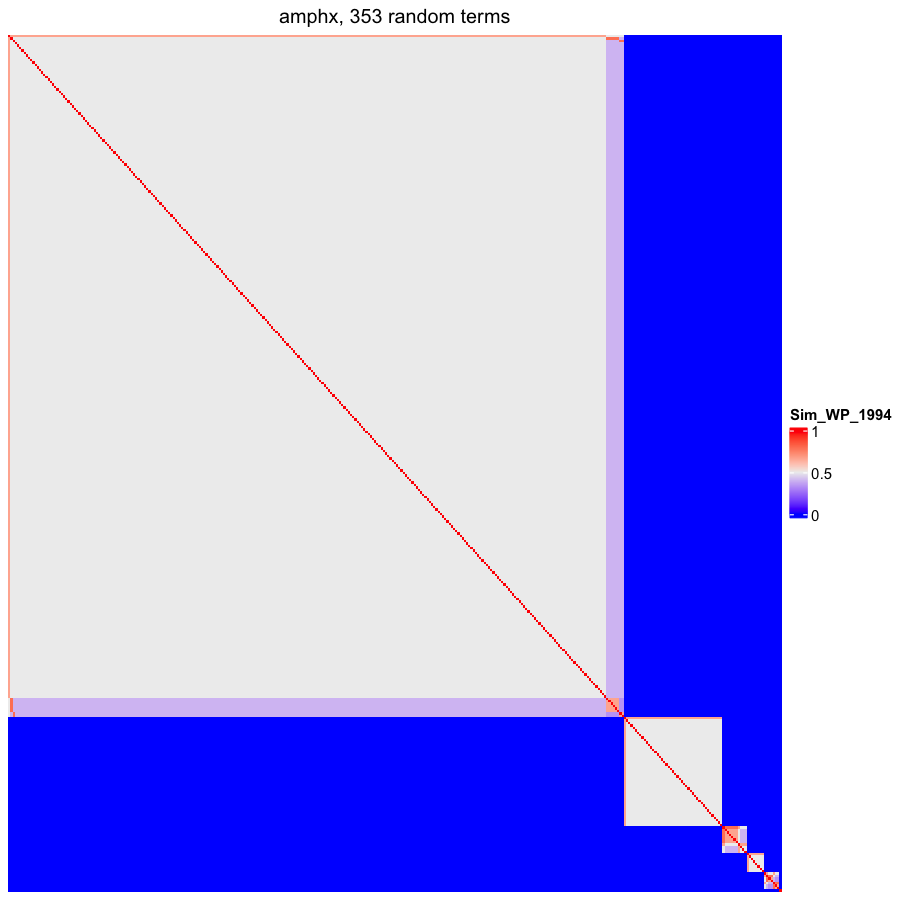

Supplement: Supplementary file 6 — Supplementary Material 6. OBO Foundry gallery [file 12864_2024_10759_MOESM6_ESM.zip › suppl6_OBOFoundry_gallery/image/OBOFoundry_amphx_heatmap.png]

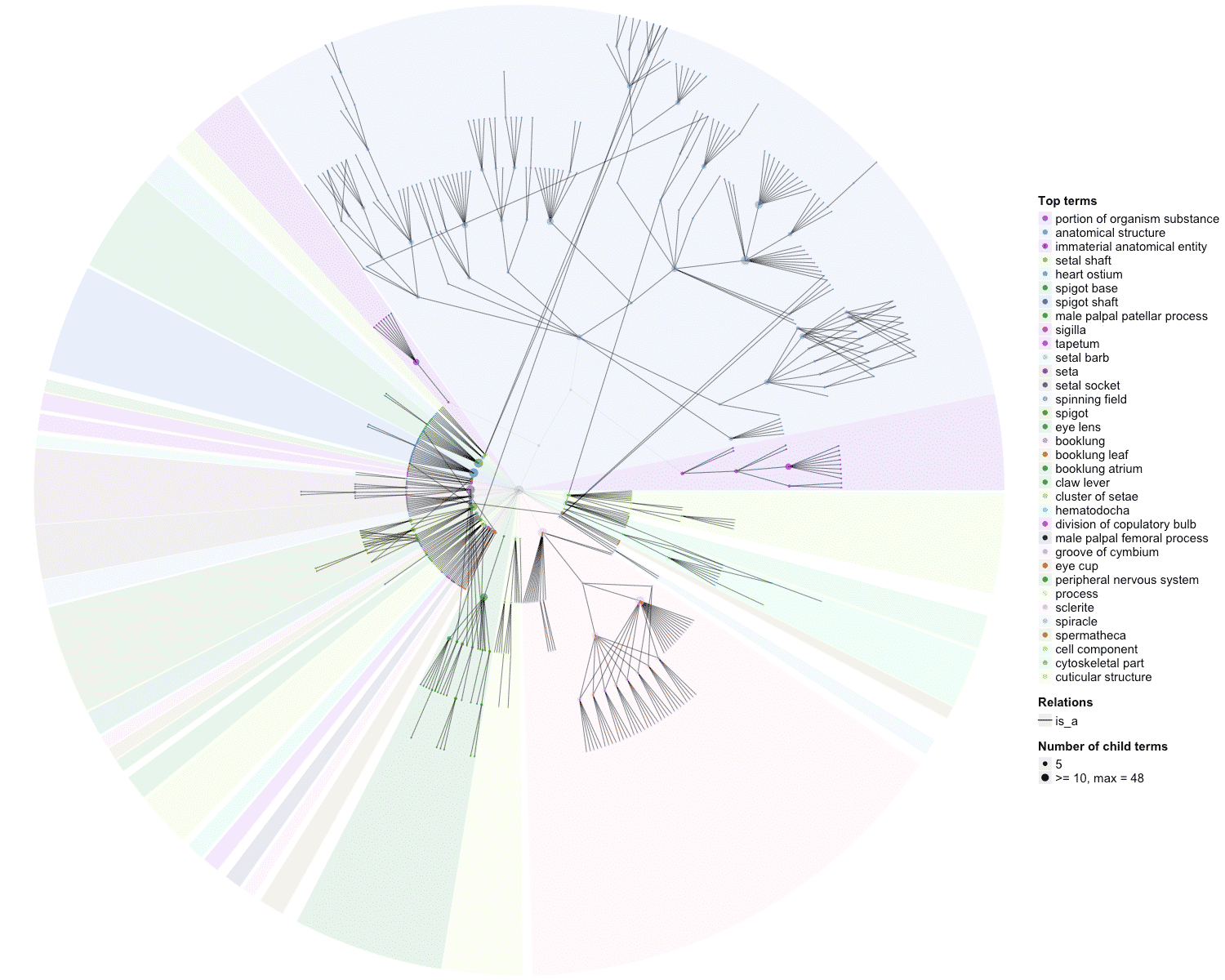

Supplement: Supplementary file 6 — Supplementary Material 6. OBO Foundry gallery [file 12864_2024_10759_MOESM6_ESM.zip › suppl6_OBOFoundry_gallery/image/OBOFoundry_spd.png]

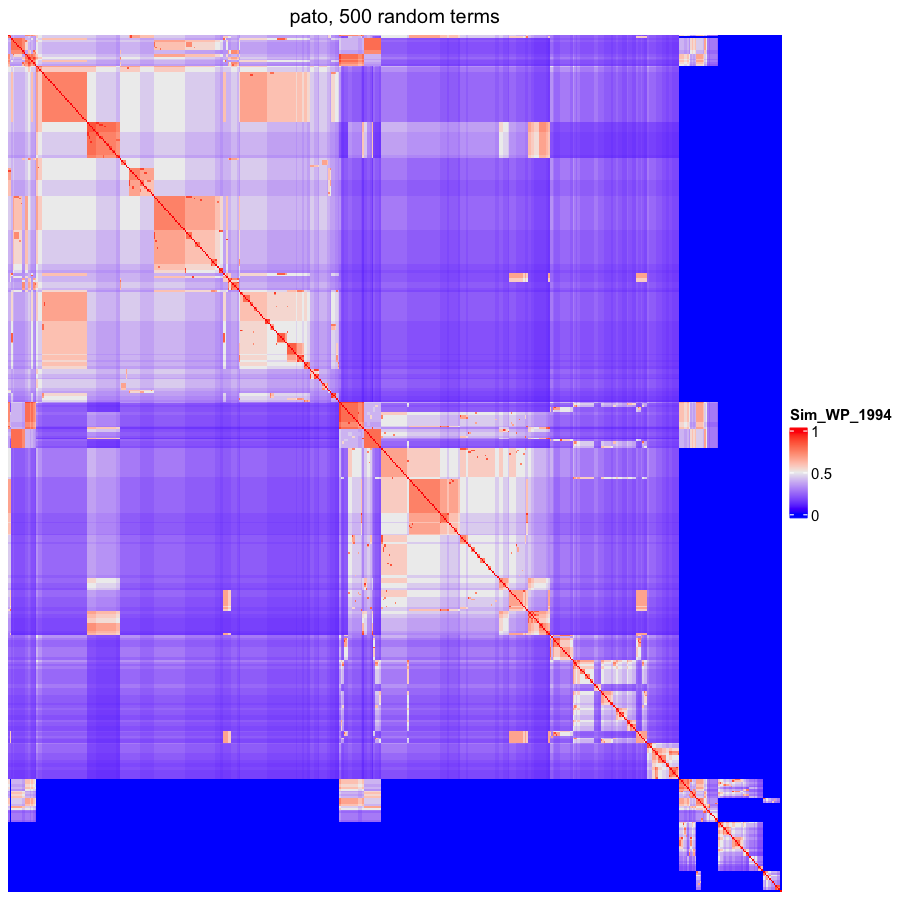

Supplement: Supplementary file 6 — Supplementary Material 6. OBO Foundry gallery [file 12864_2024_10759_MOESM6_ESM.zip › suppl6_OBOFoundry_gallery/image/OBOFoundry_pato_heatmap.png]

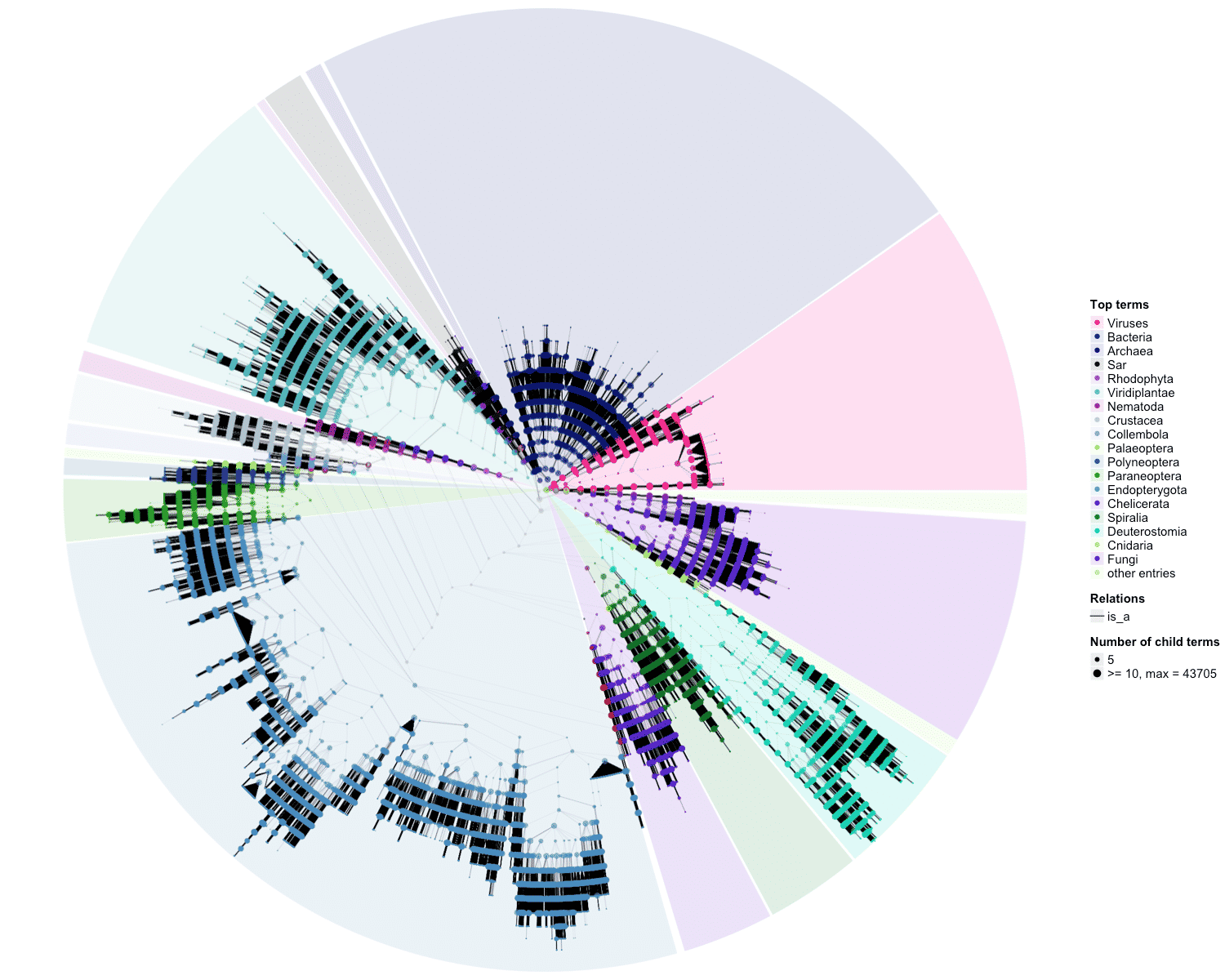

Supplement: Supplementary file 6 — Supplementary Material 6. OBO Foundry gallery [file 12864_2024_10759_MOESM6_ESM.zip › suppl6_OBOFoundry_gallery/image/OBOFoundry_ncbitaxon.png]

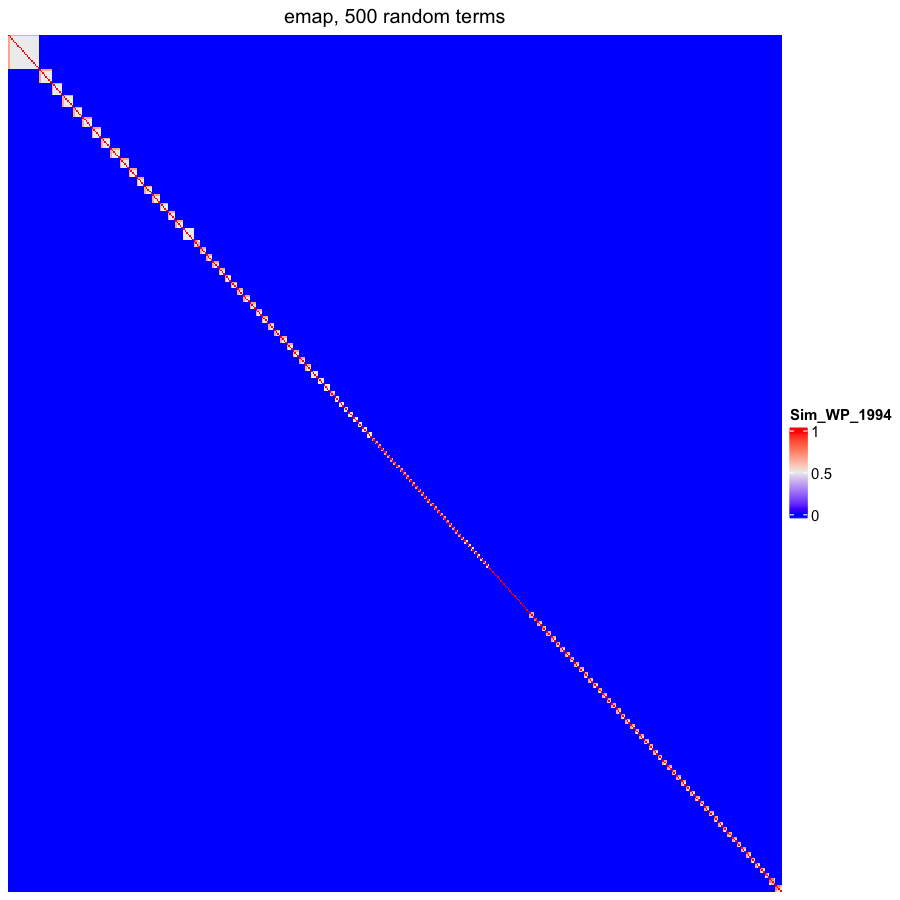

Supplement: Supplementary file 6 — Supplementary Material 6. OBO Foundry gallery [file 12864_2024_10759_MOESM6_ESM.zip › suppl6_OBOFoundry_gallery/image/OBOFoundry_emap_heatmap.png]

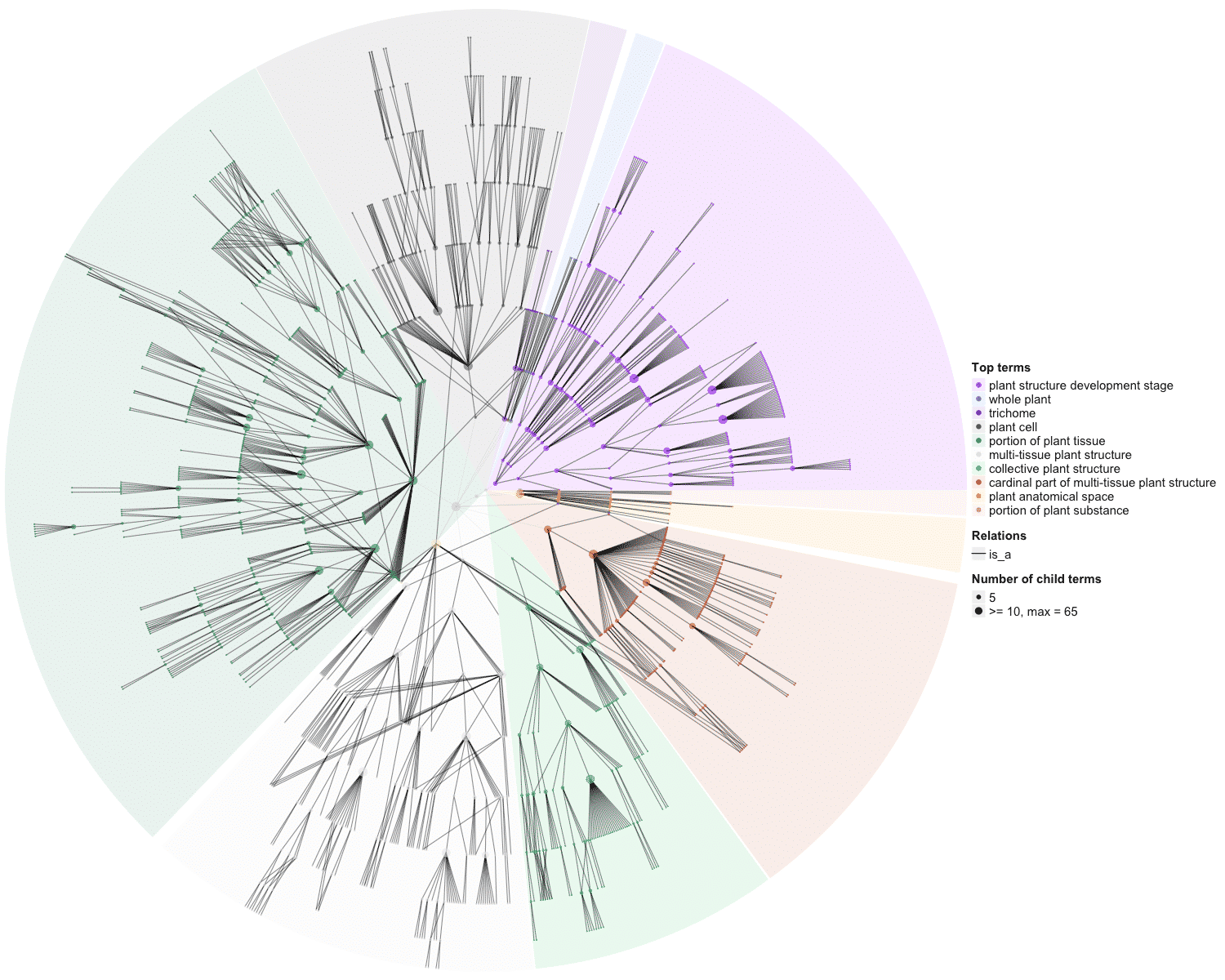

Supplement: Supplementary file 6 — Supplementary Material 6. OBO Foundry gallery [file 12864_2024_10759_MOESM6_ESM.zip › suppl6_OBOFoundry_gallery/image/OBOFoundry_po.png]

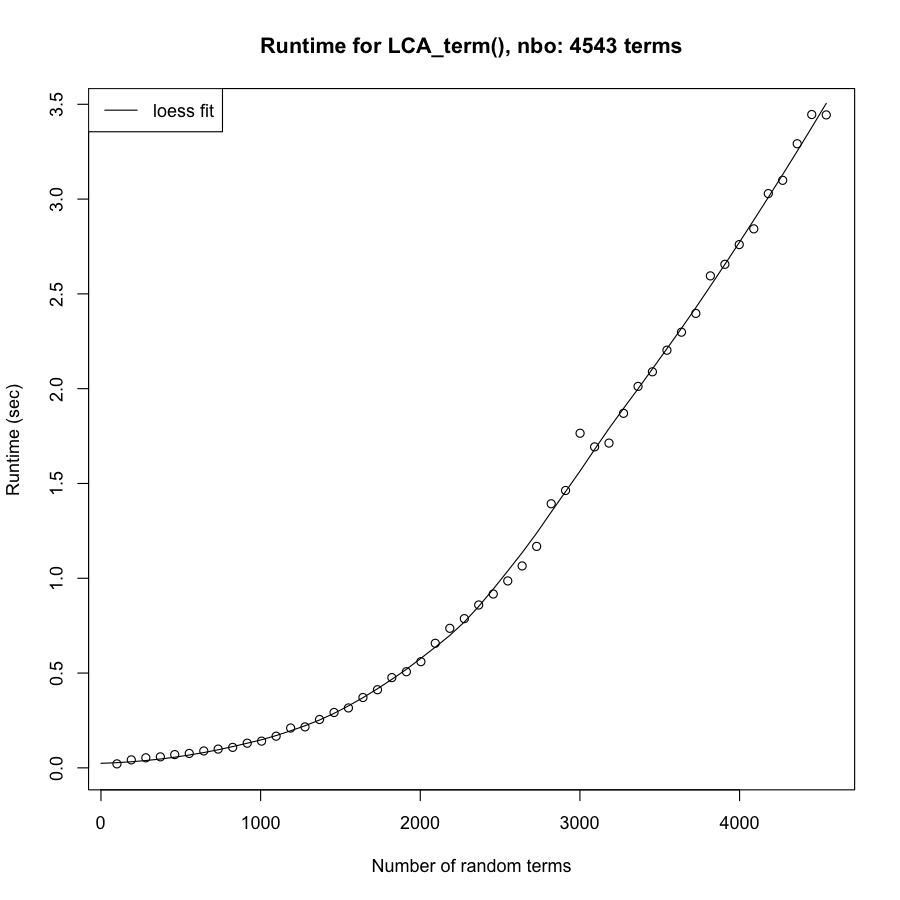

Supplement: Supplementary file 6 — Supplementary Material 6. OBO Foundry gallery [file 12864_2024_10759_MOESM6_ESM.zip › suppl6_OBOFoundry_gallery/image/OBOFoundry_nbo_runtime.png]

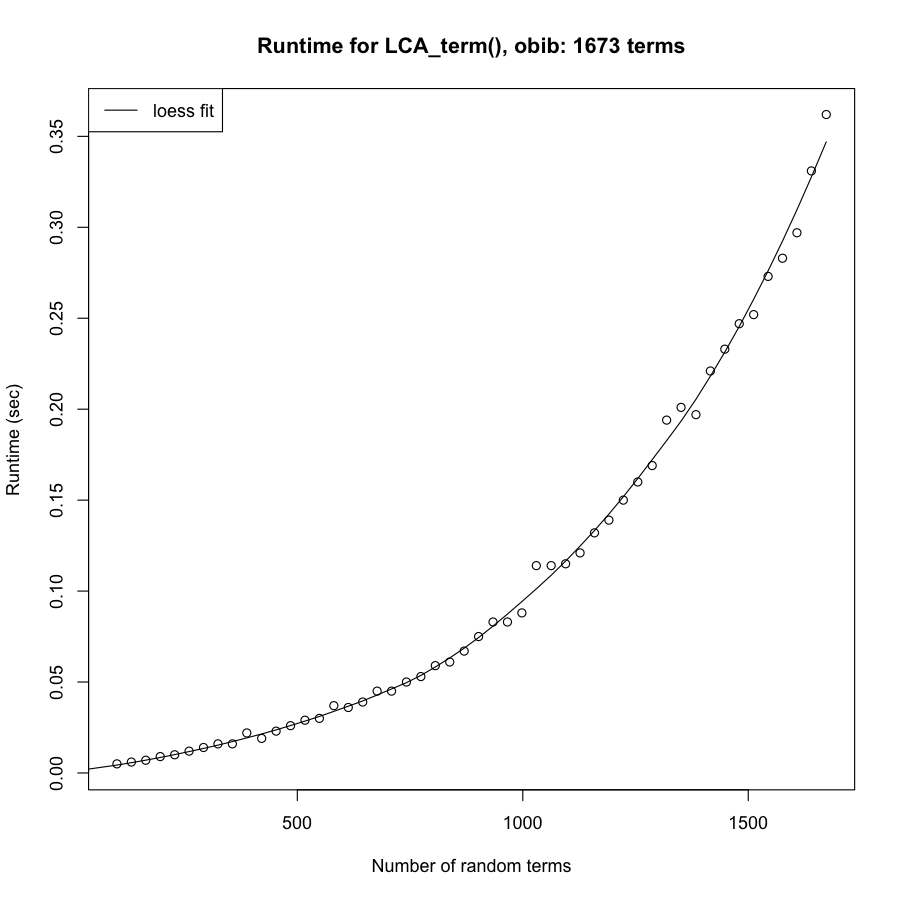

Supplement: Supplementary file 6 — Supplementary Material 6. OBO Foundry gallery [file 12864_2024_10759_MOESM6_ESM.zip › suppl6_OBOFoundry_gallery/image/OBOFoundry_obib_runtime.png]

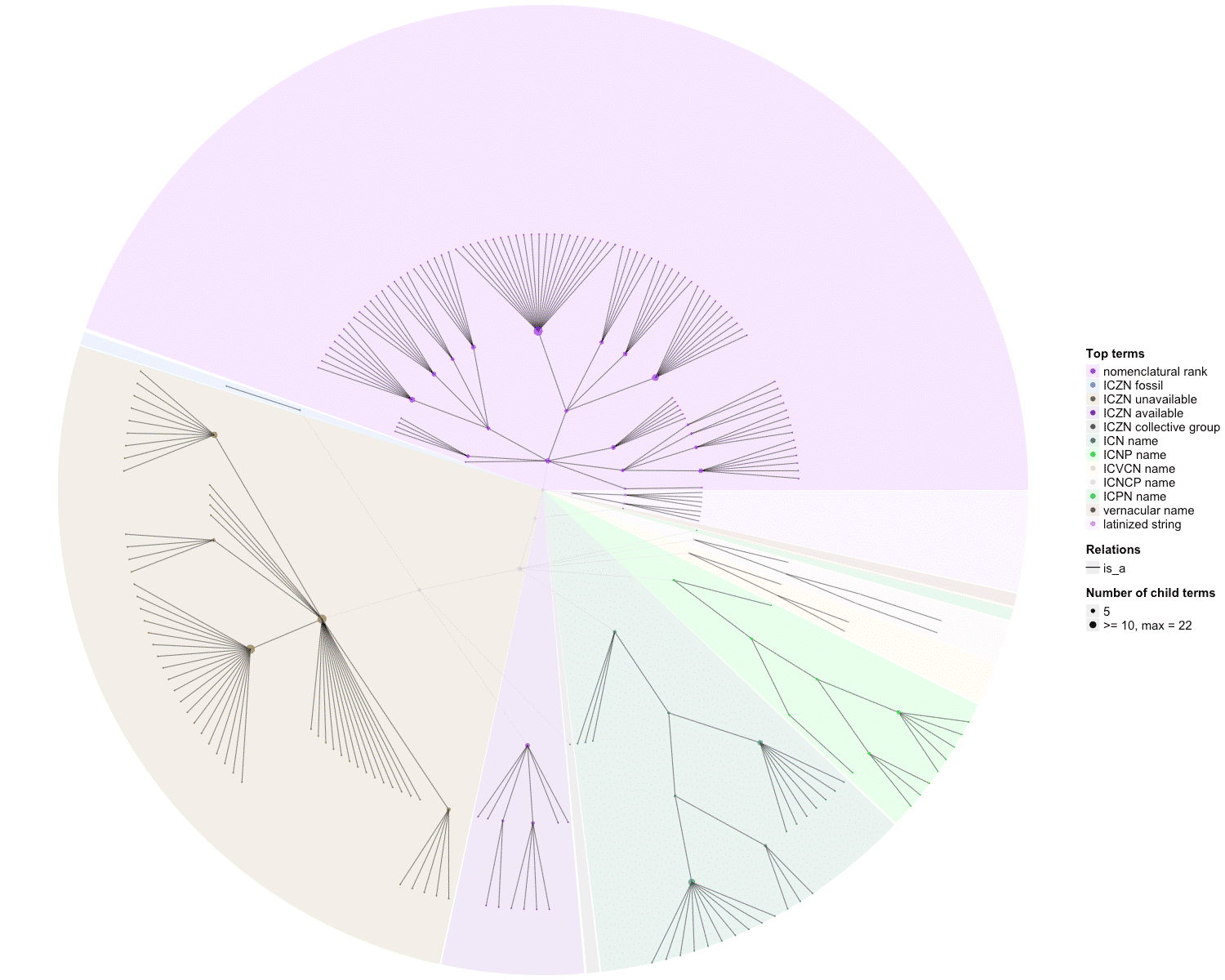

Supplement: Supplementary file 6 — Supplementary Material 6. OBO Foundry gallery [file 12864_2024_10759_MOESM6_ESM.zip › suppl6_OBOFoundry_gallery/image/OBOFoundry_nomen.png]

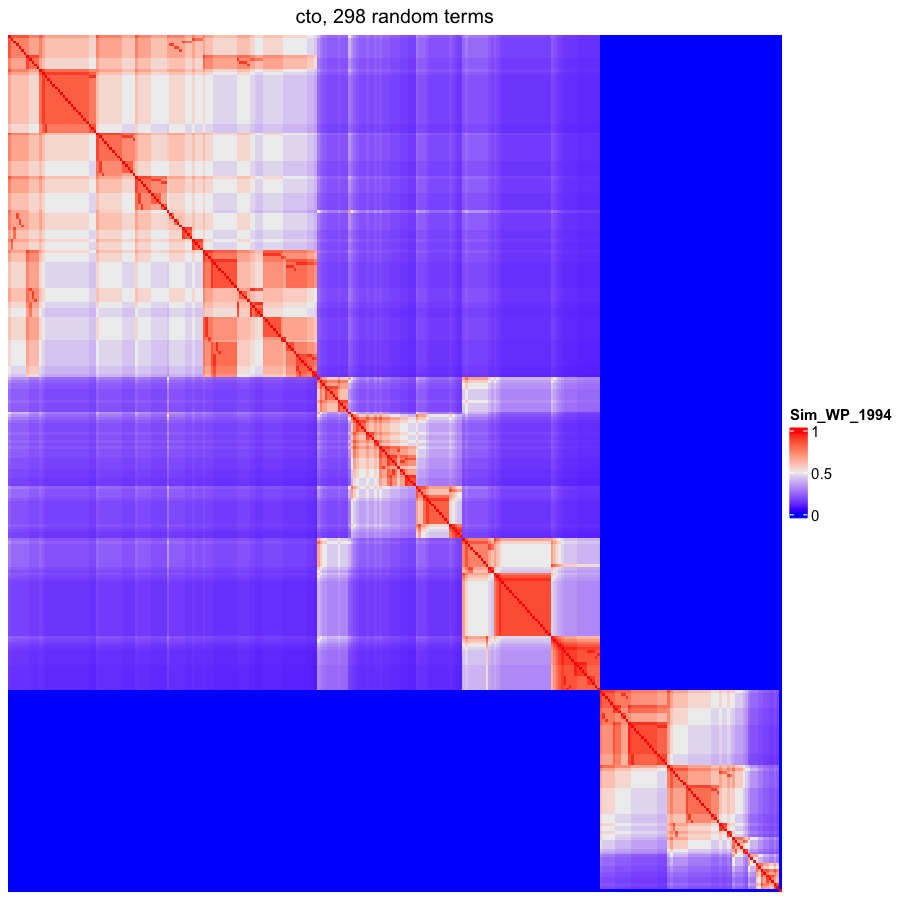

Supplement: Supplementary file 6 — Supplementary Material 6. OBO Foundry gallery [file 12864_2024_10759_MOESM6_ESM.zip › suppl6_OBOFoundry_gallery/image/OBOFoundry_cto_heatmap.png]

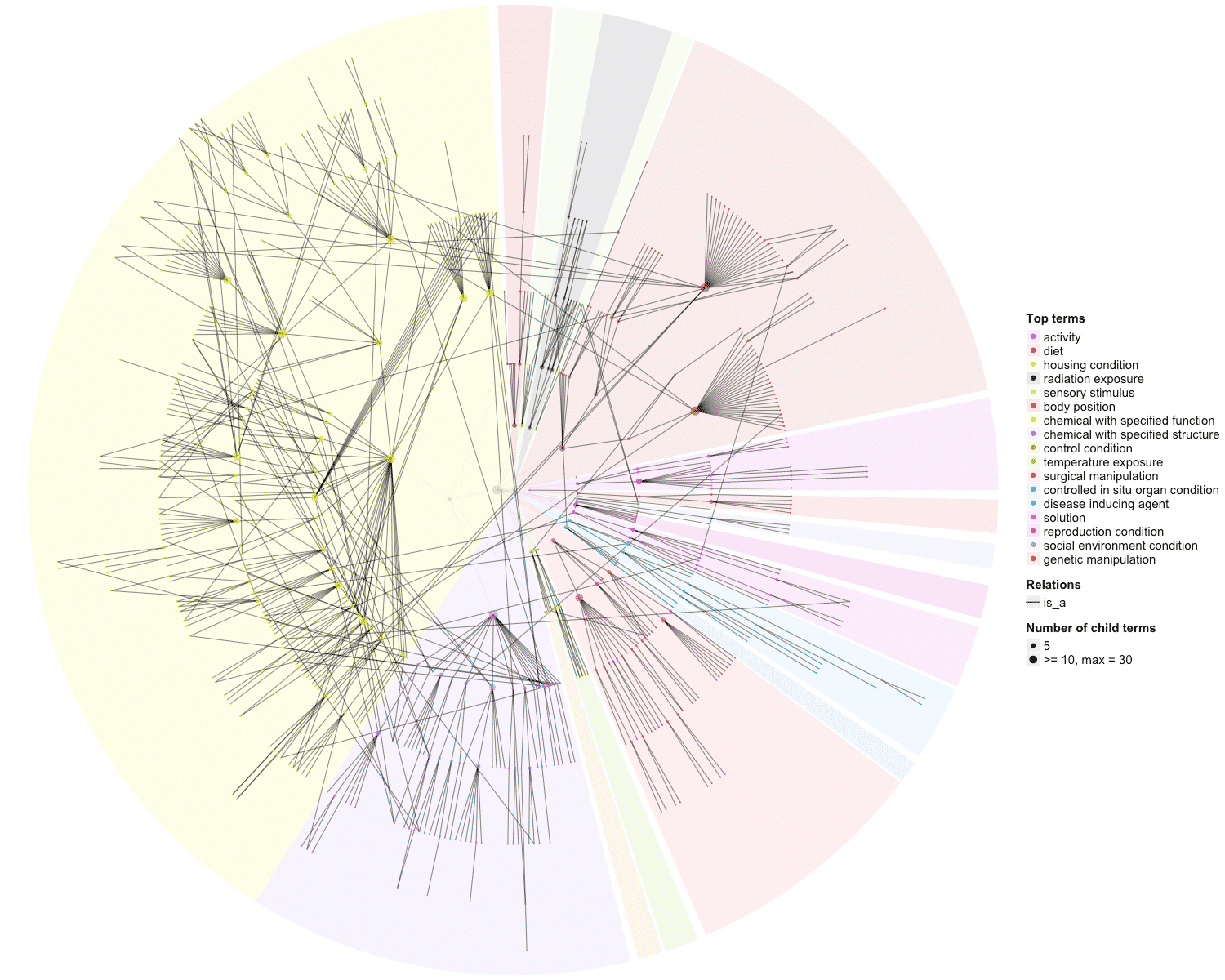

Supplement: Supplementary file 6 — Supplementary Material 6. OBO Foundry gallery [file 12864_2024_10759_MOESM6_ESM.zip › suppl6_OBOFoundry_gallery/image/OBOFoundry_xco.png]

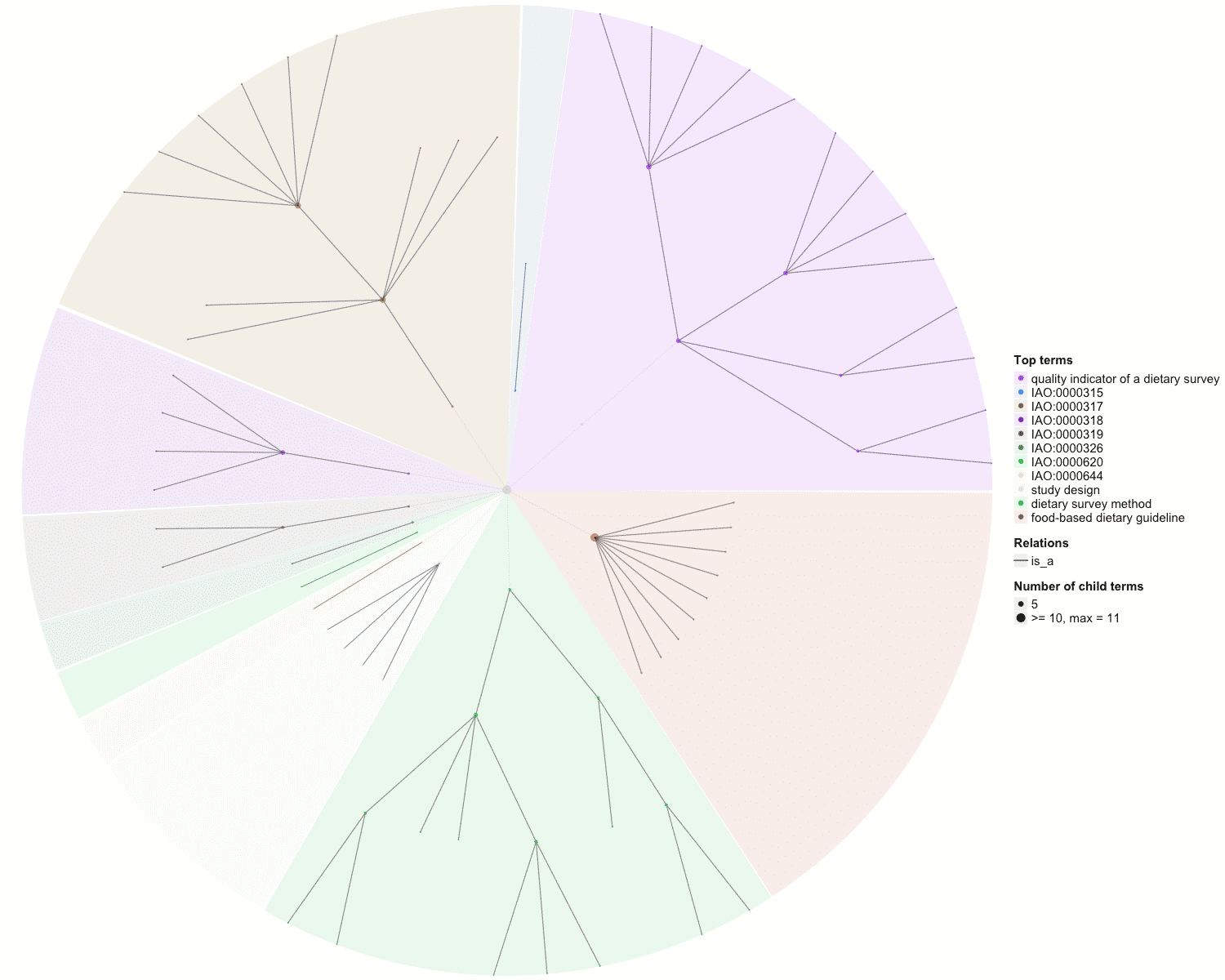

Supplement: Supplementary file 6 — Supplementary Material 6. OBO Foundry gallery [file 12864_2024_10759_MOESM6_ESM.zip › suppl6_OBOFoundry_gallery/image/OBOFoundry_one.png]

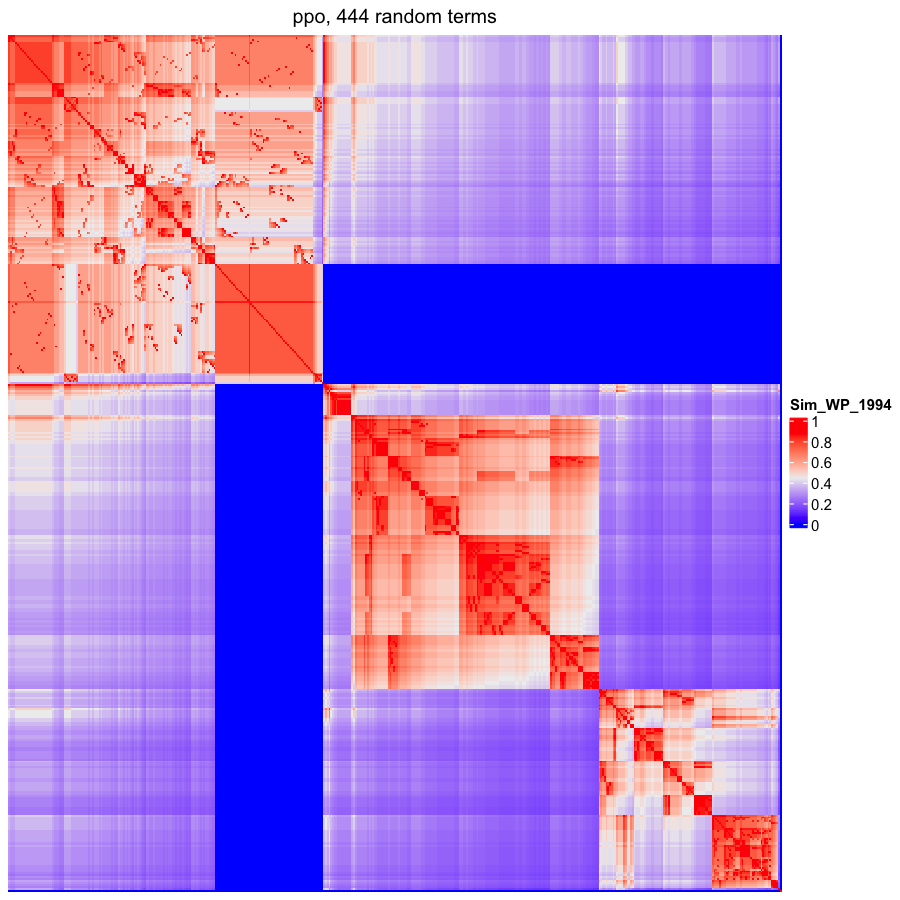

Supplement: Supplementary file 6 — Supplementary Material 6. OBO Foundry gallery [file 12864_2024_10759_MOESM6_ESM.zip › suppl6_OBOFoundry_gallery/image/OBOFoundry_ppo_heatmap.png]

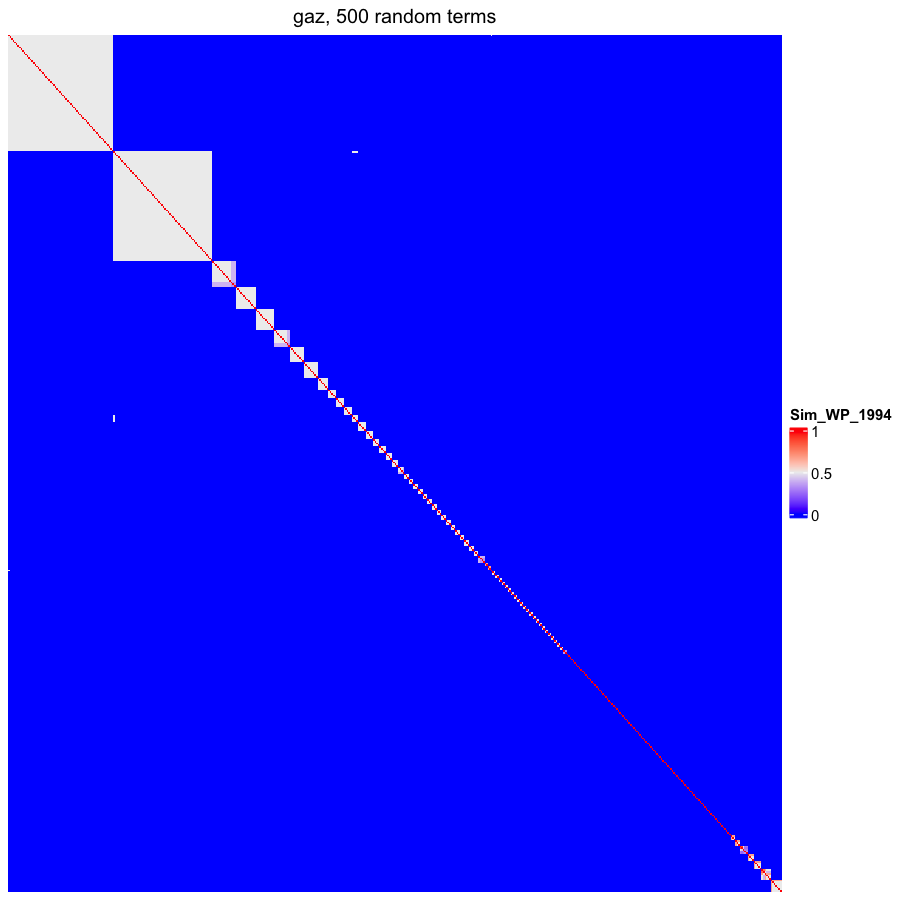

Supplement: Supplementary file 6 — Supplementary Material 6. OBO Foundry gallery [file 12864_2024_10759_MOESM6_ESM.zip › suppl6_OBOFoundry_gallery/image/OBOFoundry_gaz_heatmap.png]

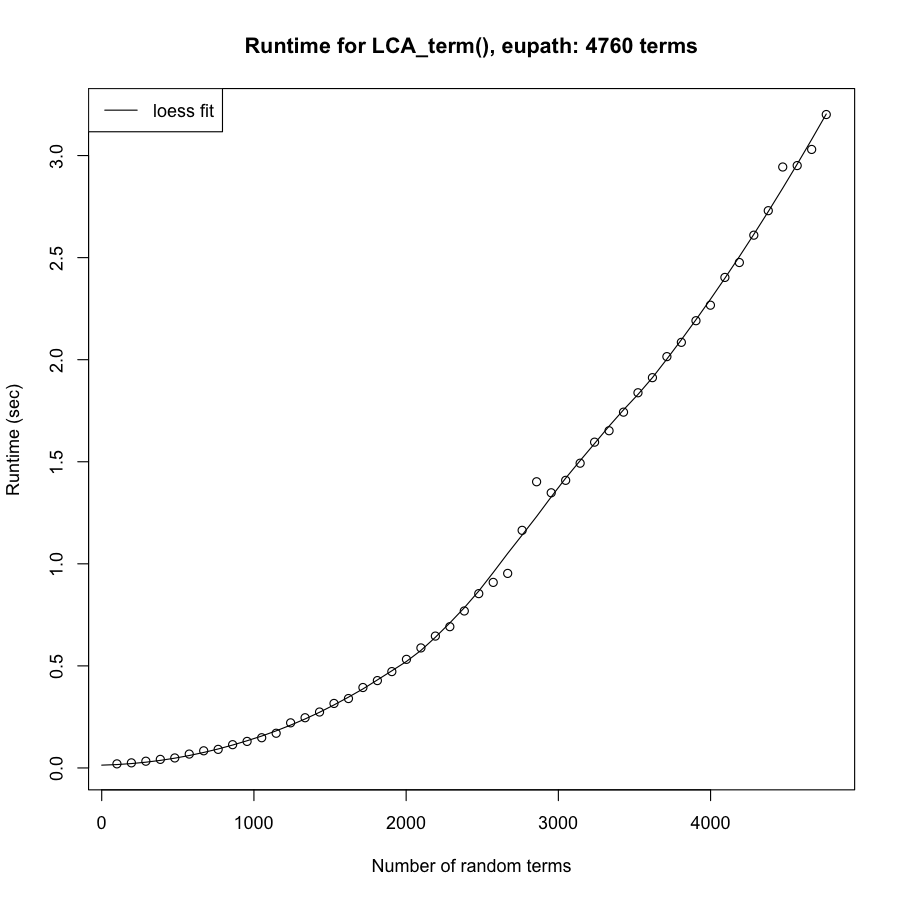

Supplement: Supplementary file 6 — Supplementary Material 6. OBO Foundry gallery [file 12864_2024_10759_MOESM6_ESM.zip › suppl6_OBOFoundry_gallery/image/OBOFoundry_eupath_runtime.png]

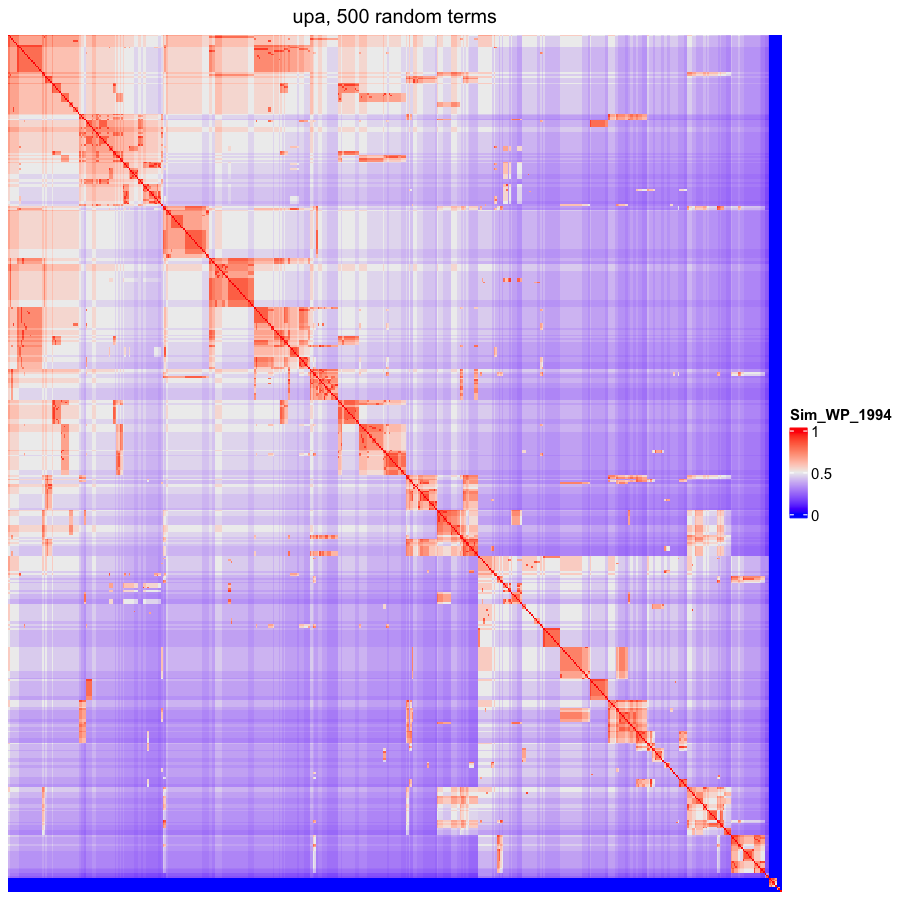

Supplement: Supplementary file 6 — Supplementary Material 6. OBO Foundry gallery [file 12864_2024_10759_MOESM6_ESM.zip › suppl6_OBOFoundry_gallery/image/OBOFoundry_upa_heatmap.png]

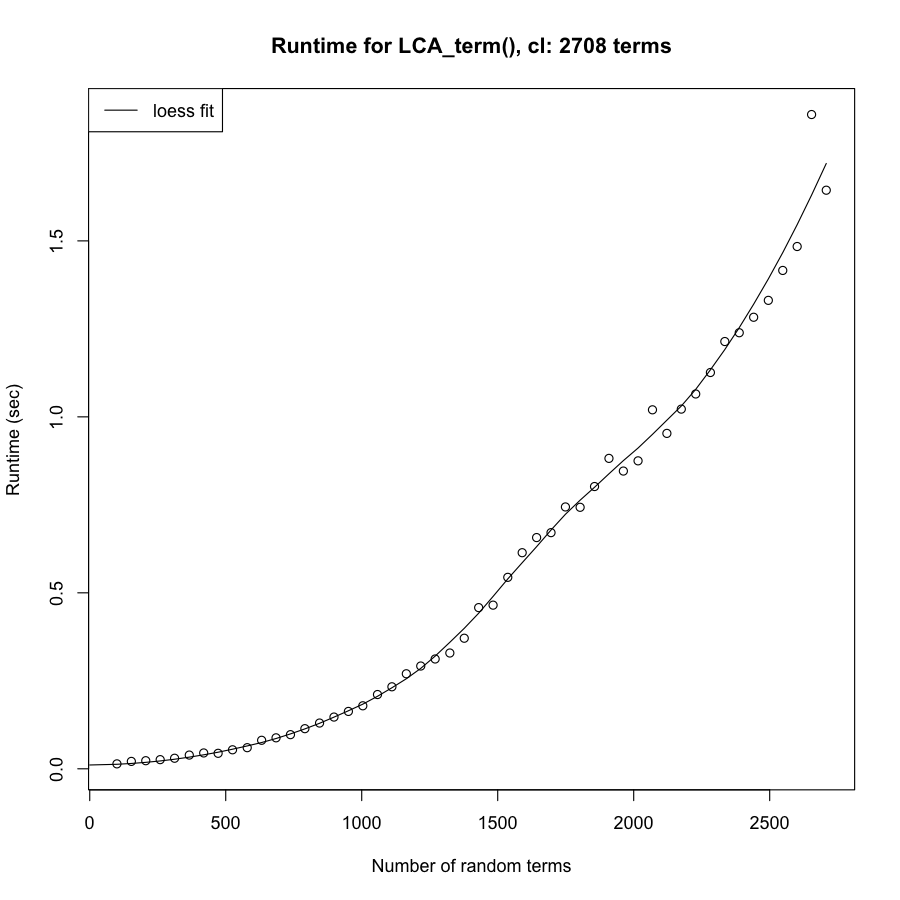

Supplement: Supplementary file 6 — Supplementary Material 6. OBO Foundry gallery [file 12864_2024_10759_MOESM6_ESM.zip › suppl6_OBOFoundry_gallery/image/OBOFoundry_cl_runtime.png]

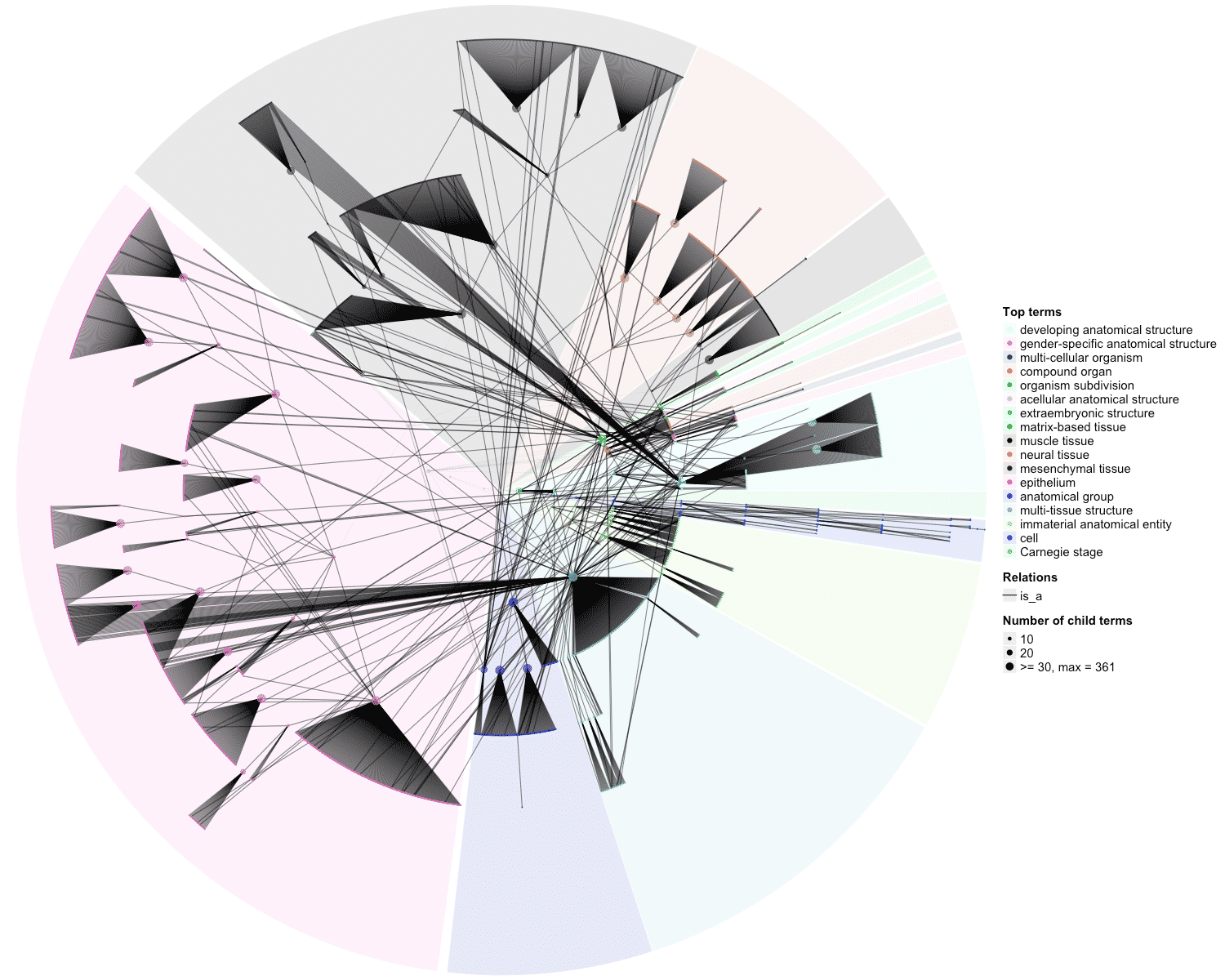

Supplement: Supplementary file 6 — Supplementary Material 6. OBO Foundry gallery [file 12864_2024_10759_MOESM6_ESM.zip › suppl6_OBOFoundry_gallery/image/OBOFoundry_ehdaa2.png]

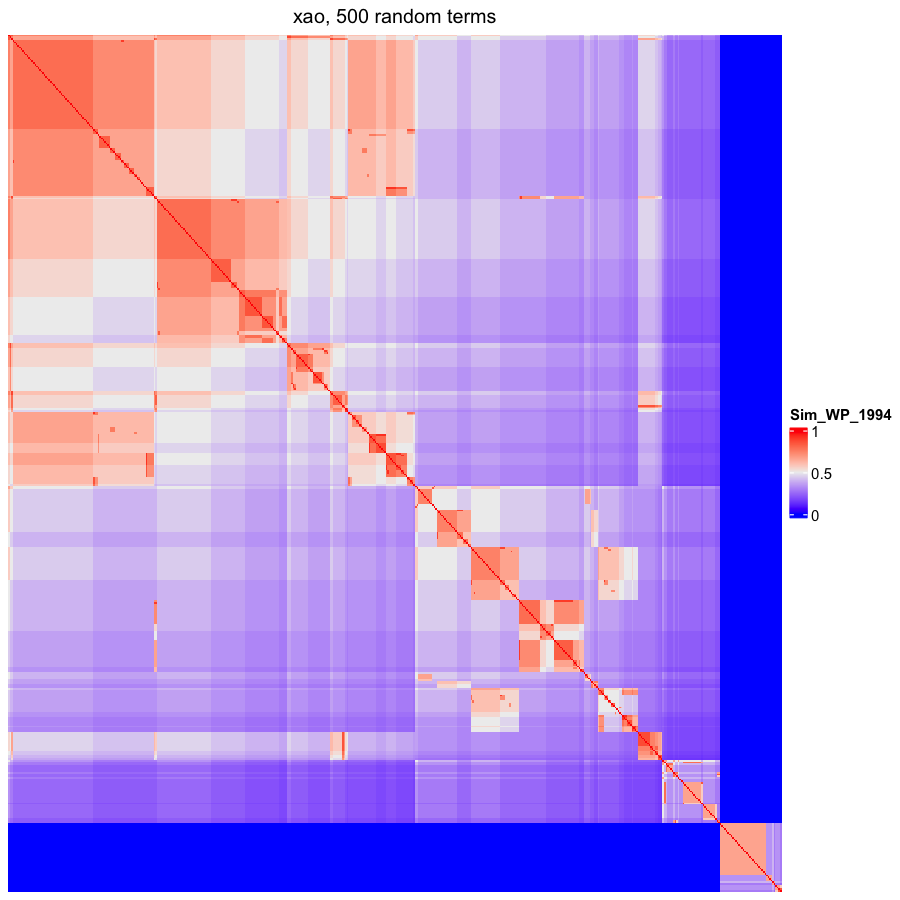

Supplement: Supplementary file 6 — Supplementary Material 6. OBO Foundry gallery [file 12864_2024_10759_MOESM6_ESM.zip › suppl6_OBOFoundry_gallery/image/OBOFoundry_xao_heatmap.png]

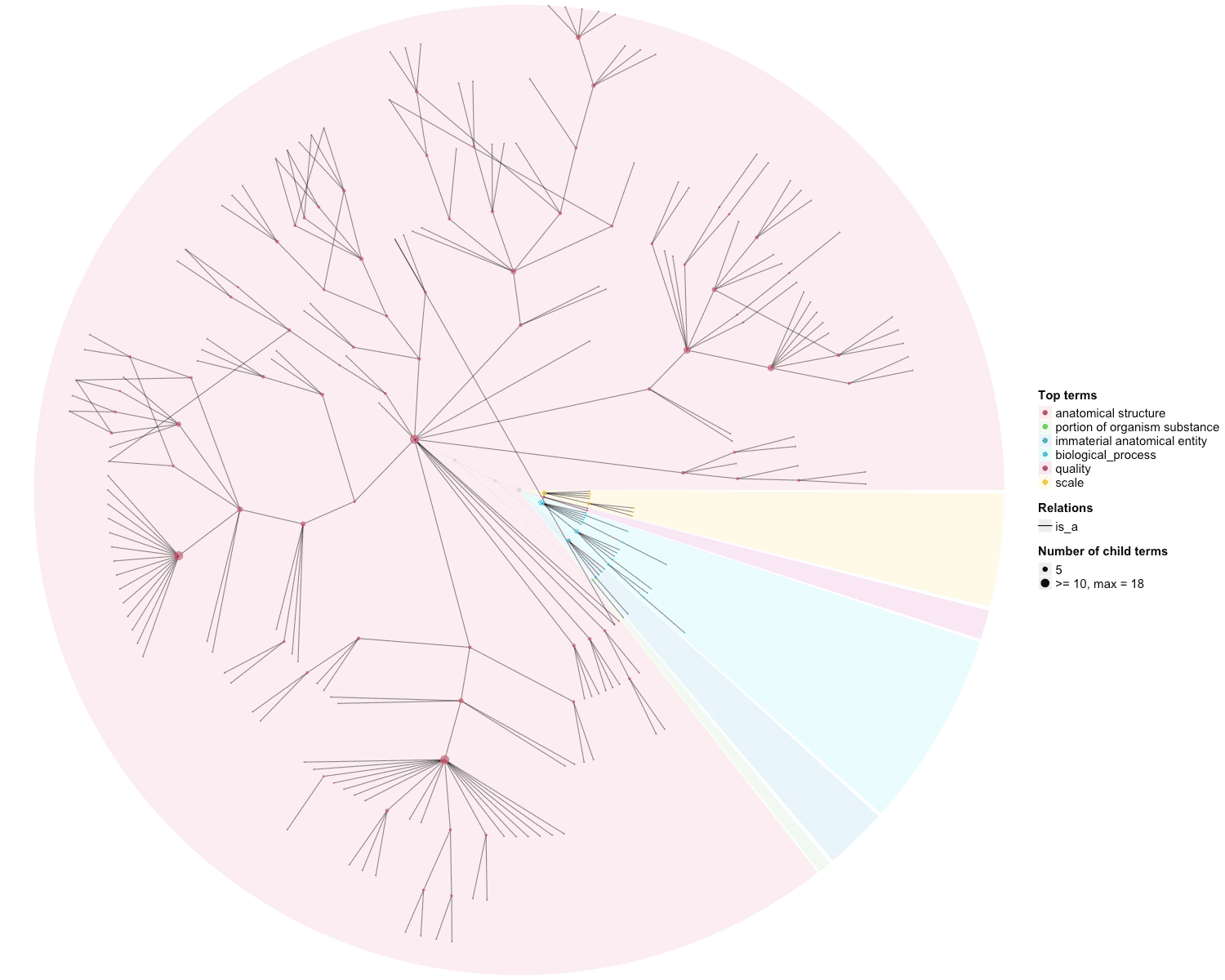

Supplement: Supplementary file 6 — Supplementary Material 6. OBO Foundry gallery [file 12864_2024_10759_MOESM6_ESM.zip › suppl6_OBOFoundry_gallery/image/OBOFoundry_vsao.png]
